# Bioinspired Collective Synthesis of Gymnothelignans I, F, K, and L

**DOI:** 10.1021/acs.joc.5c03261

**Published:** 2026-04-07

**Authors:** Nannaphat Chumsri, Kamonchanok Chualong, Arthit Chairoungdua, Chutima Kuhakarn, Pawaret Leowanawat, Vichai Reutrakul, Darunee Soorukram

**Affiliations:** a Department of Chemistry, Faculty of Science, 26685 Mahidol University, Rama VI Road, Bangkok 10400, Thailand; b Department of Physiology, Faculty of Science, 26685 Mahidol University, Rama VI Road, Bangkok 10400, Thailand; c Center of Excellence for Innovation in Chemistry (PERCH−CIC), Faculty of Science, 26685 Mahidol University, Rama VI Road, Bangkok 10400, Thailand

## Abstract

We report a collective stereoselective synthesis of gymnothelignan members, i.e., gymnothelignans I, F, K, and L, based on their proposed biosynthetic pathways. A chiral eupomatilone skeleton was employed as a key common intermediate. Reduction of a carbonyl moiety of eupomatilone readily gave gymnothelignan I, which was smoothly converted to the corresponding oxocarbenium ion upon treatment with an acid. Subsequent intermolecular and intramolecular nucleophilic additions of respective nucleophiles to the oxocarbenium ion provided gymnothelignans F, K, and L. This work provides information to support the plausible biosynthetic pathway of these structurally unique lignans.

## Graphical abstract

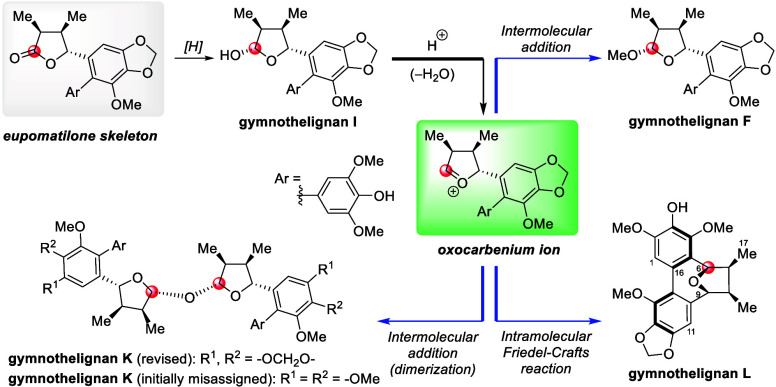

## Introduction

Gymnothelignans are a structurally unique subclass of lignans^1^ bearing a dimethyl-substituted tetrahydrofuran (THF) ring with variable conformations and stereochemistries across the THF ring. The first isolation of gymnothelignans A–O was revealed in 2012 by Xu et al.^2^ Based on their chemical structures, gymnothelignans A–O belong to three unusual and potentially related skeletons, namely, eupodienone,^3^ eupomatilone,^4^ and dibenzocyclooctadiene skeletons^5^ (Figure 1). Up to now, a series of new members of these three skeletons were isolated and identified. Recently, two new distinct scaffolds, namely, acyclic^6^ and spirocyclic THF-type^7^ skeletons, were included as new gymnothelignan skeletons. Due to their interesting structural frameworks, gymnothelignans have served as significant synthetic targets for the synthetic community, and during the past decade, considerable achievements in their total syntheses have been achieved.^8^ Driven by our interest in developing the asymmetric synthesis of gymnothelignans,^9^ we report herein a biomimetic synthesis of gymnothelignans I (**1**), F (**2**), K (revised structure) (**3**), and L (**4**) (Figure 1). Notably, the synthesis of the initially misassigned structure of gymnothelignan K is also reported.

**Figure 1:**
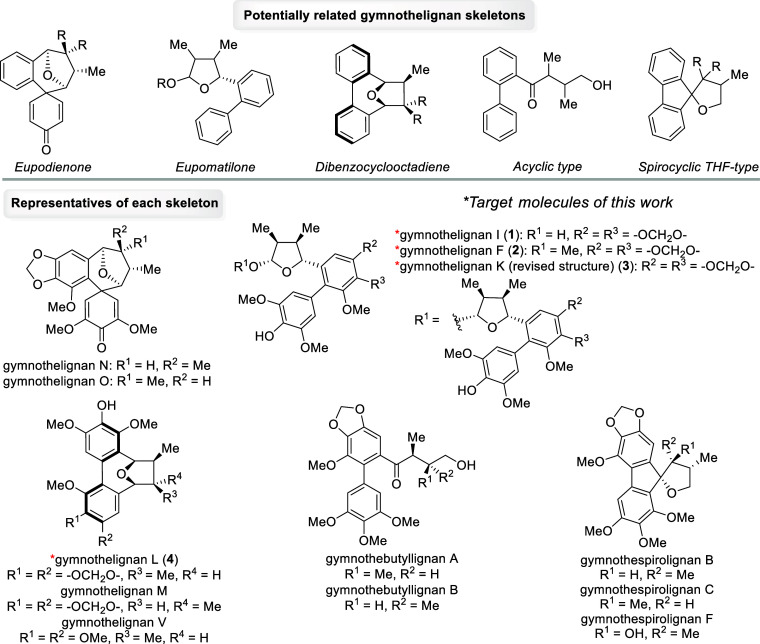
Potentially related gymnothelignan skeletons, representative members, and target molecules of this work.

The biosynthesis of gymnothelignans via spirohemiketal **I** ^4a^ was first proposed by Xu et al.^2,4a^ An alternative biosynthetic route starting from 2,5-diaryltetrahydrofurans was proposed by She et al.^8a^ We speculated^9b^ that all five skeletons of gymnothelignans (Figure 1) may be biogenetically generated from spirohemiketal **I** (Scheme 1). While dehydroxylation of spirohemiketal **I** gives eupodienone skeleton **II** (gymnothelignans N and O), rearrangement of spirohemiketal **I** provides eupomatilone skeleton **III** (eupomatilones 1–6), which could further undergo reduction to provide the corresponding gymnothelignans **IV** (gymnothelignans I and J). Structures **III** and **IV** serve as the key intermediates, leading to several gymnothelignan members. Dibenzocyclooctadiene skeleton **V** (gymnothelignans L and M) was proposed to be achieved via intramolecular Friedel–Crafts reaction through an oxocarbenium cation intermediate derived from **IV**. On the other hand, intermolecular nucleophilic addition should deliver gymnothelignan **VI** with diverse substituents and contiguous stereochemistries across the THF ring (gymnothelignans A–H and K). Newly reported acyclic skeleton **VII** and spirocyclic THF-type **VIII** were proposed to be derived from eupomatilone **III**. Lactone ring-opening followed by oxidation and reduction (vice versa) should provide **VII** (gymnothebutyllignans A and B), which could further undergo double cyclization to provide **VIII** (gymnothespirolignans A–F).

**Scheme 1:**
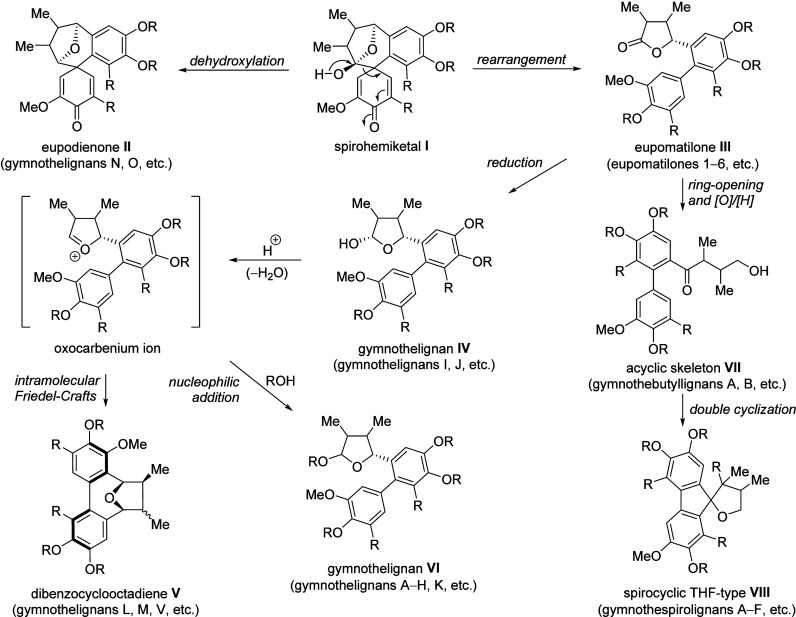
Proposed Biosynthetic Pathway to Gymnothelignans

Based on the above-mentioned plausible biosynthetic pathway, the synthetic plan to gymnothelignans I (**1**), F (**2**), K (**3**), and L (**4**) was proposed as depicted in Scheme 2 using eupomatilone **6** as a key synthetic intermediate. Lignan **1** should be obtained via a stereoselective reduction of **6**. Next, the formation of ether-linkage of **2** and **3** was planned to be constructed via intermolecular nucleophilic addition of an appropriate nucleophile to an oxocarbenium ion intermediate **5**, which should be readily generated upon treatment of **1** with an acid.^10^ The intramolecular Friedel–Crafts reaction of **5** should give **4**. Eupomatilone **6** should be readily synthesized via lactonization of acyclic skeleton **7**, which, in turn, was planned to be prepared through the reaction of organolithium species, derived from biaryl bromide **8**, and chiral aldehyde **9**.

**Scheme 2:**
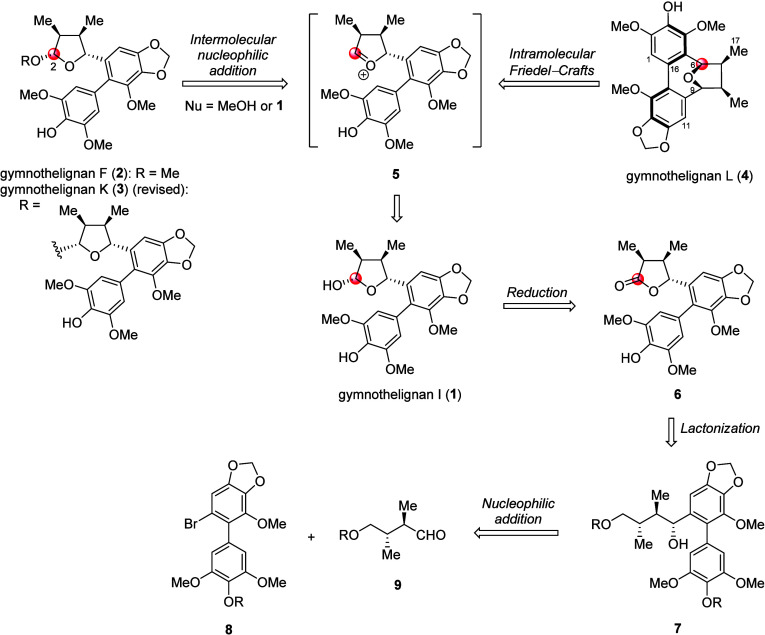
Synthetic Plan

## Results and Discussion

### Synthesis of Eupomatilone Skeletons **6**

The synthesis of **6** commenced with the preparation of biaryl bromide **8a** (Scheme 3A). Following the reported literature, aryl bromide **10** (36% yield) was synthesized in 3 steps starting from *o*-vanillin.^11^ After extensive optimization (see the Supporting Information), the regioselective iodination at C-5 of **10** was achieved upon treatment of **10** with molecular iodine and silver trifluoroacetate in CHCl_3_ at room temperature (35 °C) for 20 h, providing **11a** in 60% isolated yield. The structure of **11a** was confirmed based on NOESY experiments. Next, **8a** was prepared via Suzuki–Miyaura coupling between [4-(benzyloxy)-3,5-dimethoxyphenyl]­boronic acid (**12**) and **11a**, bearing both aryl bromide and aryl iodide moieties. After several attempts (see the Supporting Information), chemoselective Suzuki–Miyaura coupling of **12** with the C–I bond of **11a** was achieved by using Pd­(PPh_3_)_4_ (20 mol %) and K_3_PO_4_ (7 equiv) in DME/H_2_O (10:1, v/v) at refluxing temperature for 24 h to yield **8a** in 94% yield.^12^ Meanwhile, chiral aldehyde **9** was readily prepared via the oxidative cleavage of the alkene moiety of **13** (dr = 93:7) (Scheme 3B).^9^ Subsequent reaction of **9** with organolithium species, freshly generated from the reaction of **8a** and *n*-BuLi, in THF at −78 °C for 5 h provided the corresponding alcohols **7a** and **7b** along with the other two minor isomers (dr = 44:43:8:5, ^1^H NMR analysis) as an inseparable mixture (38% combined yield from **13**). Using organocerium species, prepared from transmetalation of the lithiated derivative of **8a** using CeCl_3_ solution (0.5 M in THF), to react with **9** gave inferior results. Alcohols **7a** and **7b** along with other two minor isomers (dr = 34:55:9:2, ^1^H NMR analysis) were obtained in 10% combined yield. Desilylation of this mixture followed by oxidative lactonization of the corresponding diols using a combination of DIB/TEMPO in CH_2_Cl_2_ at room temperature (35 °C) provided **6a** ^8n^ (34% yield) and **6b** (43% yield) each as a single diastereomer. The relative stereochemistry of compounds **6a** and **6b** was confirmed by NOESY experiments. Alternatively, **6a** can be readily prepared in 3 steps from a mixture of **7a** and **7b** (dr = 51:49) (Scheme 3C). After oxidation using PDC, ketone **14** was obtained in 70% yield. Substrate-controlled (Felkin–Ahn model)^13^ stereoselective reduction of the carbonyl moiety of **14** (NaBH_4_, CeCl_3_·7H_2_O, MeOH) gave **7a** as a major diastereomer (91% yield, dr = 95:5, ^1^H NMR analysis). Desilylation (TBAF) of **7a** followed by oxidative lactonization (DIB/TEMPO) gave **6a** as a single diastereomer in 89% yield (2 steps). Noteworthily, chiral ketones of types **14** are new members of gymnothelignan scaffolds (Figure 1 and Scheme 1), and they were recently employed as the key substrates for the synthesis of gymnothespirolignans.^8p^

**Scheme 3:**
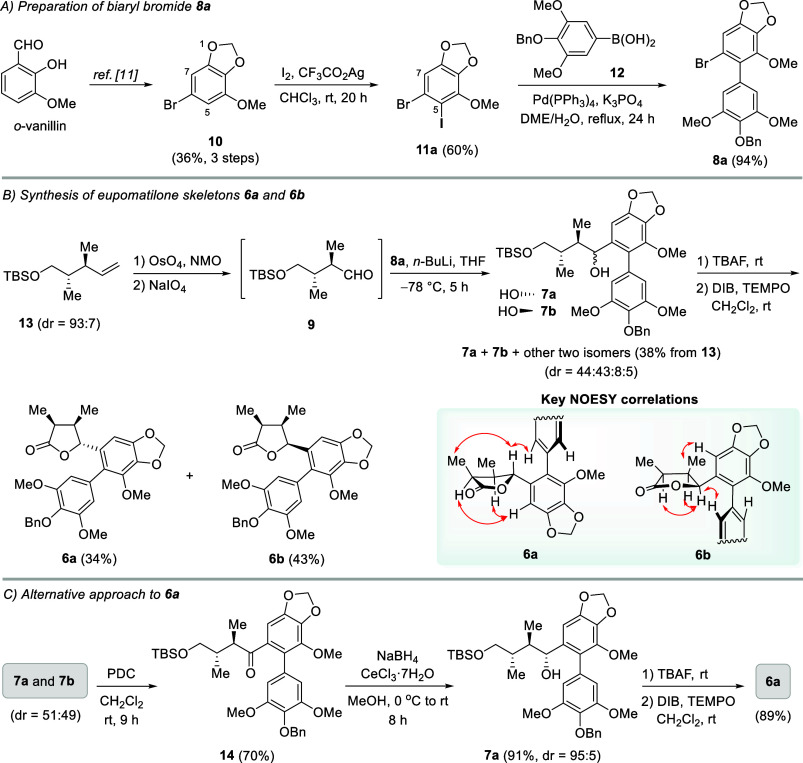
(A–C) Synthesis of Eupomatilone Skeletons **6**

### Synthesis of Gymnothelignans I (**1**) and F (**2**) from **6a**

After having **6a** in hand, the synthesis of gymnothelignan I (**1**) was carried out (Scheme 4). Compound **6a** (single isomer) was subjected to debenzylation (H_2_, Pd/C, EtOAc, room temperature (rt), 30 min). After filtration through a Celite pad and concentration, the corresponding phenol product (99%, ^1^H NMR analysis) was obtained and used in the next step without further chromatographic purification. Subsequent reduction of a keto group using DIBAL-H in CH_2_Cl_2_ at −78 °C for 1.5 h yielded **1** and its C-2 epimer (dr = 86:14, ^1^H NMR analysis) in 99% combined yield. Attempts to separate **1** and its C-2 epimer by means of chromatographic methods were not successful. We thus decided to use a mixture of **1** and its C-2 epimer in the synthesis of gymnothelignan F (**2**). Thus, treatment of the mixture of **1** and its C-2 epimer with a catalytic amount of *p*-TsOH·H_2_O in the presence of trimethyl orthoformate in dry MeOH at room temperature (35 °C) overnight (16 h) provided, after chromatography, **2** and 2-*epi*-gymnothelignan F (**15**) in 58% and 11% yields (3 steps from **6a**), respectively. Each of the products was obtained as a single diastereomer. The relative stereochemistry of **2** and **15** was established on the basis of NOESY experiments (see the Supporting Information).

**Scheme 4:**
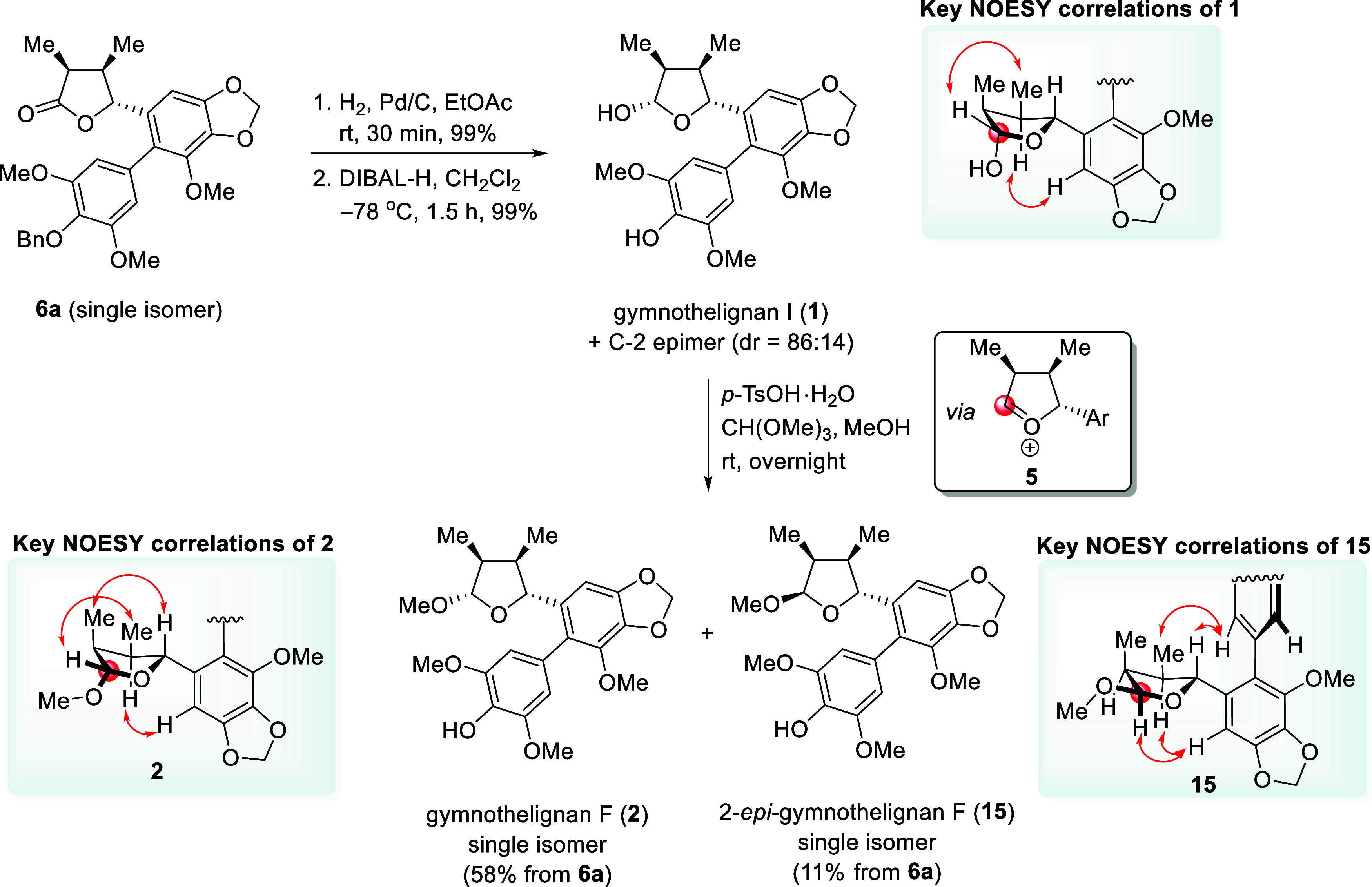
Synthesis of Gymnothelignans I (**1**) and F (**2**) from **6a**

### Synthesis of Gymnothelignans K (**3**) (Revised) and L (**4**) from **1**

Noteworthily, the condensation of **1**, via the generation of **5** followed by the intermolecular nucleophilic addition of **1** to **5** (Scheme 2), to form gymnothelignan K (**3**) (revised) presents a greater challenge since intramolecular Friedel–Crafts reaction of **5** leading to gymnothelignan L (**4**) is usually a major competitive reaction. To find the suitable conditions, a more readily available substrate, gymnothelignan J (**16**),^9b^ was employed as a model substrate to synthesize **17**. The results were summarized in Table 1. Notably, **17** was originally proposed as the initially misassigned structure of gymnothelignan K,^2^ of which the structure was revised later to be **3** based on the synthesis reported by Lee and co-workers.^8s^

**Table 1:**
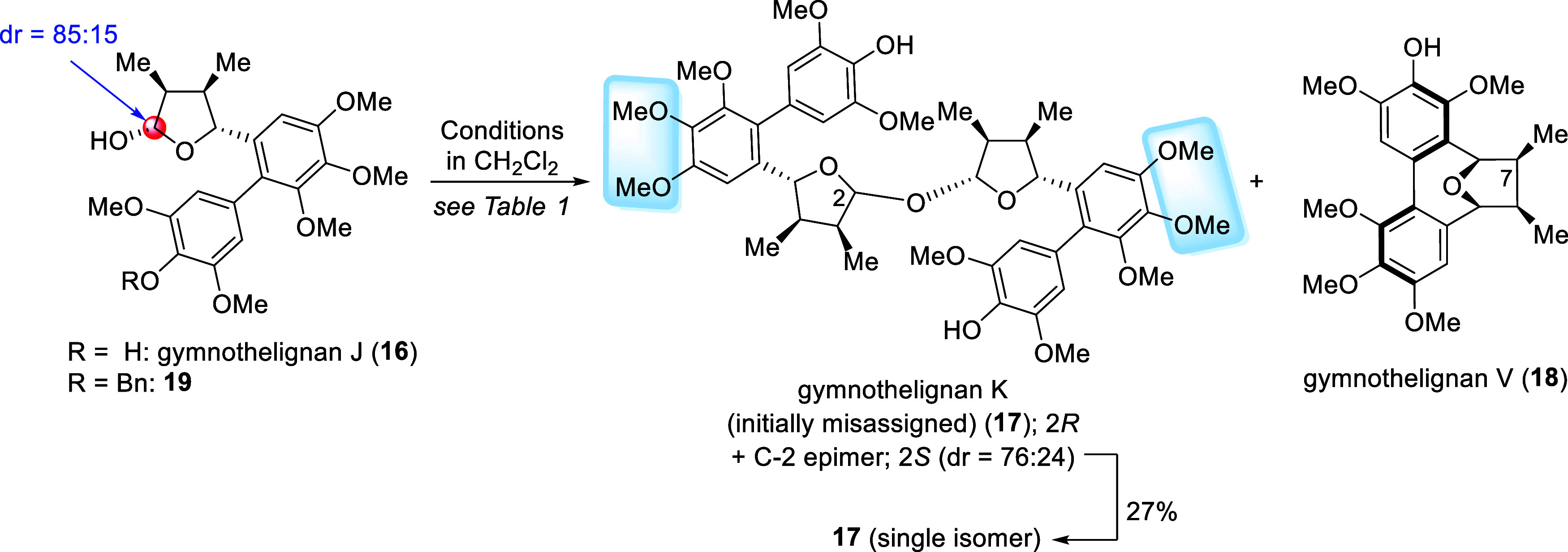
Optimization of the Dimerization of Gymnothelignan J (**16**) and Synthesis of the Initially Misassigned Gymnothelignan K (**17**)

entry	conditions^a^	^1^H NMR ratio^b^ of **16**:**17**:**18**
1	*p*-TsOH·H_2_O (0.5 equiv), 4 Å MS, 0 °C to rt, 3 days	50:46:4
2	*p*-TsOH·H_2_O (2 equiv), CuSO_4_·5H_2_O (3 equiv), 4 Å MS, 0 °C to rt, 48 h	79:17:4
3	*p*-TsOH·H_2_O (10 equiv), CuSO_4_·5H_2_O (6 equiv), 4 Å MS, 0 °C to rt, 18 h	10:1:89
4	*p*-TsOH·H_2_O (0.5 equiv), CuSO_4_·5H_2_O (3 equiv), 0 °C to rt, 8 h	29:38:33
5^c^	*p*-TsOH·H_2_O (1 equiv), CuSO_4_·5H_2_O (3 equiv), 0 °C, 12 h	33:53:14
6^d^	*p*-TsOH·H_2_O (1 equiv), CuSO_4_·5H_2_O (3 equiv), 0 °C, 12 h	17:51:4
7^e^	(1) Ac_2_O, pyridine, 4 Å MS	0:0:100
	(2) H_2_, Pd/C, EtOAc	
	(3) **16** (1.1 equiv), SnCl_4_ (1 equiv), 4Å MS, CH_2_Cl_2_, –78 °C (2 h)/0 °C (1 h)	

a Unless stated otherwise, **16** was used as a substrate.

b The ratio of **16**:**17**:**18** (obtained each as a mixture of diastereomers) was determined by ^1^H NMR analysis of a crude mixture, unless stated otherwise.

c **17** was isolated in 56% yield (dr = 76:24).

d Dimethyl sulfone was used as an internal standard, and the NMR yield was reported.

e Using **19** as a substrate. **18** was isolated in 52% yield.

Our attempts focused on the use of *p*-TsOH·H_2_O as a pivotal acid protomer for the dimerization without employing an additional chiral catalyst.^8s^ Based on our previous report, gymnothelignan V (**18**) was obtained as a sole product when **16** was treated with only *p*-TsOH·H_2_O in CH_2_Cl_2_.^9b^ Using slightly modified Lee’s conditions^8s^ (*p*-TsOH·H_2_O, 4 Å MS, CH_2_Cl_2_, 0 °C to rt, 3 days), the formation of **17** was observed (46% conversion, ^1^H NMR analysis) (Table 1, entry 1). The use of CuSO_4_ to promote the dimerization was next evaluated according to the reports by Cubero et al. and Barker et al.^14^ However, using a combination between *p*-TsOH·H_2_O and CuSO_4_·5H_2_O in the presence of 4 Å MS gave inferior results (Table 1, entries 2 and 3). Among these cases, **18** was a major product (89% conversion) when a large excess of *p*-TsOH·H_2_O (10 equiv) was employed (Table 1, entry 3). After an extensive screening, we were delighted to find that using a combination of *p*-TsOH·H_2_O and CuSO_4_·5H_2_O in the absence of 4 Å MS led to the formation of **17** in an acceptable ratio within a reasonable reaction time (Table 1, entries 4 and 5). Under these conditions, maintaining the reaction temperature at 0 °C is important to minimize the competitive formation of **18**. Thus, treatment of **16** with *p*-TsOH·H_2_O (1 equiv) and CuSO_4_·5H_2_O (3 equiv) in CH_2_Cl_2_ at 0 °C for 12 h gave a mixture of **16**:**17**:**18** (obtained each as a mixture of diastereomers) in a 33:53:14 ratio as determined by ^1^H NMR analysis (Table 1, entry 5); **17** (dr = 76:24) could be readily isolated in 56% yield. An analytically pure **17** could be obtained in 27% yield by column chromatography. Quantitative NMR analysis under these conditions, using dimethyl sulfone as an internal standard, gave results consistent with the reported NMR ratios for the formation of product **17** (Table 1, entry 6). Notably, increasing the stoichiometry of reagents and prolonged reaction time led to the competitive formation of **18** in a higher ratio. Using the reaction conditions reported by Li et al. was also unsuccessful (Table 1, entry 7);^15^ **18** and its C-7 epimer were obtained as the sole products. We therefore decided to choose the reaction conditions in entry 5 (Table 1) as the optimal conditions for the synthesis of **17** and **3**.

The synthesis of **3** and **4** could be readily accomplished in three steps starting from **6a** (single isomer) (Scheme 5). After debenzylation and reduction of a lactone moiety, the obtained **1** and its C-2 epimer (dr = 83:17) were then treated with *p*-TsOH·H_2_O (1 equiv) and CuSO_4_·5H_2_O (3 equiv) under the optimized reaction conditions (entry 5, Table 1) to give **3** and its C-2 epimer (dr = 91:9) in 61% yield and **4** (single isomer) in 8% yield (Scheme 5A), respectively. Analytically pure **3** was obtained in 24% yield by column chromatography. The equilibration between the **3**/C-2 epimer and **1** was examined; under the optimized dimerization conditions, the conversion of the **3**/C-2 epimer (dr = 85:15) into **1** (30% conversion) was detected by ^1^H NMR analysis (Scheme 5B). These results support our observation that attempting to drive the reaction to the complete consumption of **1** (or **16**) usually led to the competitive formation of **4** (or **18**) as the major product.

**Scheme 5:**
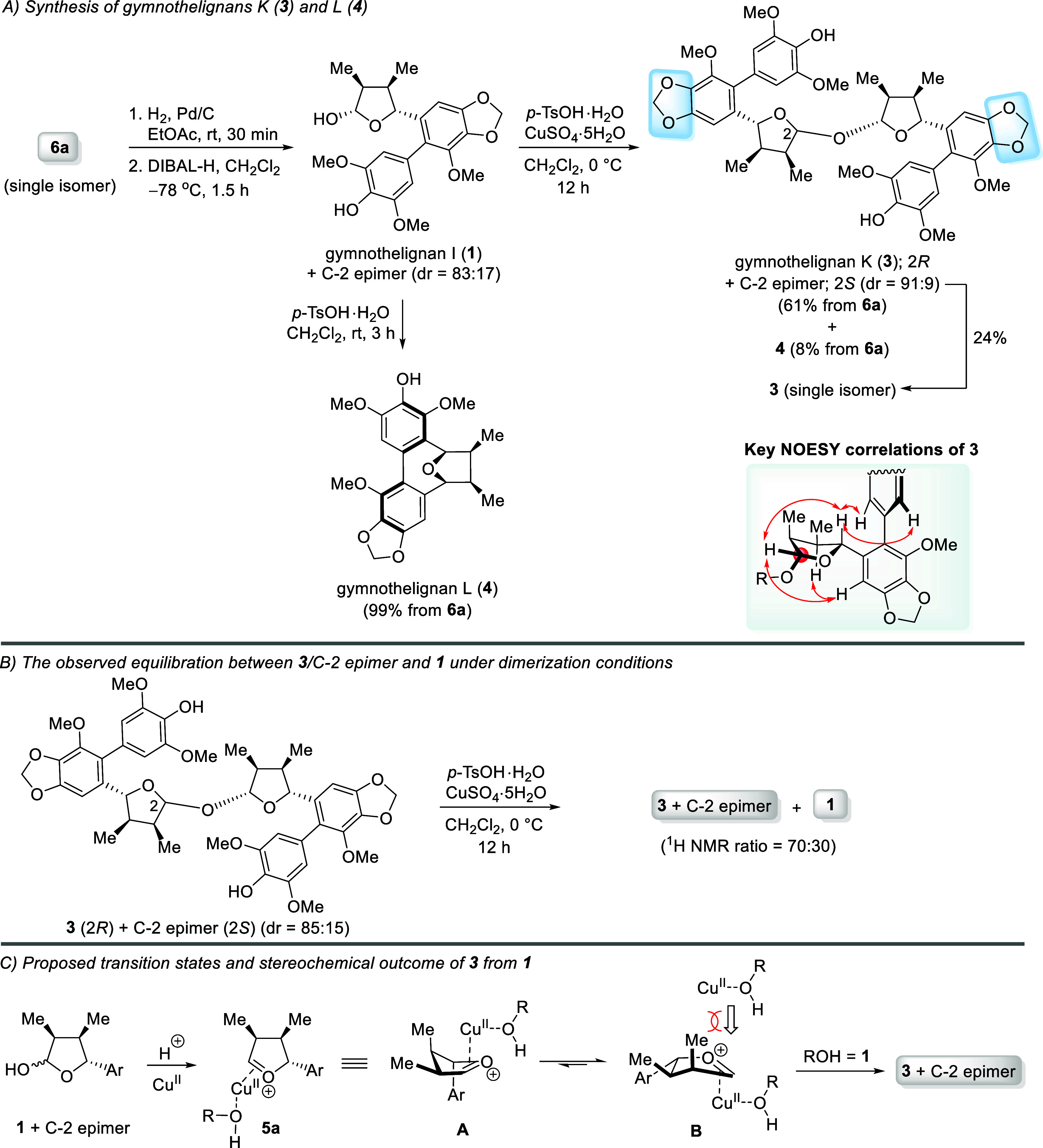
(A–C) Synthesis of Gymnothelignans K (**3**) and L (**4**)

A plausible rationalization for the formation of **3** as a major product is proposed (Scheme 5C). Together with a Bro̷nsted acid (*p*-TsOH), CuSO_4_ acts as a Lewis acid to initially coordinate with the hydroxyl group of **1**, thus activating the formation of an oxocarbenium ion complex **5a**. It was proposed that the coordination between the copper­(II)-complex and the double bond of the oxocarbenium ion depicted in **5a** is important to minimize the competitive intramolecular Friedel–Crafts reaction to form **4**. Finally, the attack of the nucleophile from the less sterically hindered axial face of more favorable conformer **B** (**A** vs **B**)^16^ led to **3** as a major product.

On the other hand, the efficient synthesis of **4** was accomplished via intramolecular Friedel–Crafts reaction of the oxocarbenium ion derived from **1**/C-2 epimer.^8n^ In this work, after debenzylation of **6a** followed by reduction of a lactone moiety, treatment of the obtained **1**/C-2 epimer (dr = 83:17) with a catalytic amount of *p*-TsOH·H_2_O in CH_2_Cl_2_ (rt, 3 h) gave **4** in 99% yield (3 steps) (Scheme 5A).

The synthesis of previously unreported diastereomers of gymnothelignan members, i.e., compounds **20**, **21**, and **22**, was also demonstrated by following the synthetic sequences described for **1**, **2**, and **3** (Scheme 6). Compounds **20** (dr = 73:27) and **21** (dr = 71:29) were obtained each as an inseparable mixture along with their C-2 epimers in 99% and 68% combined yields, respectively, starting from **6b** (single isomer). Finally, dimerization of the **20**/C-2 epimer (dr = 73:27) gave **22**, which was isolated as a single isomer in 21% yield.

**Scheme 6:**
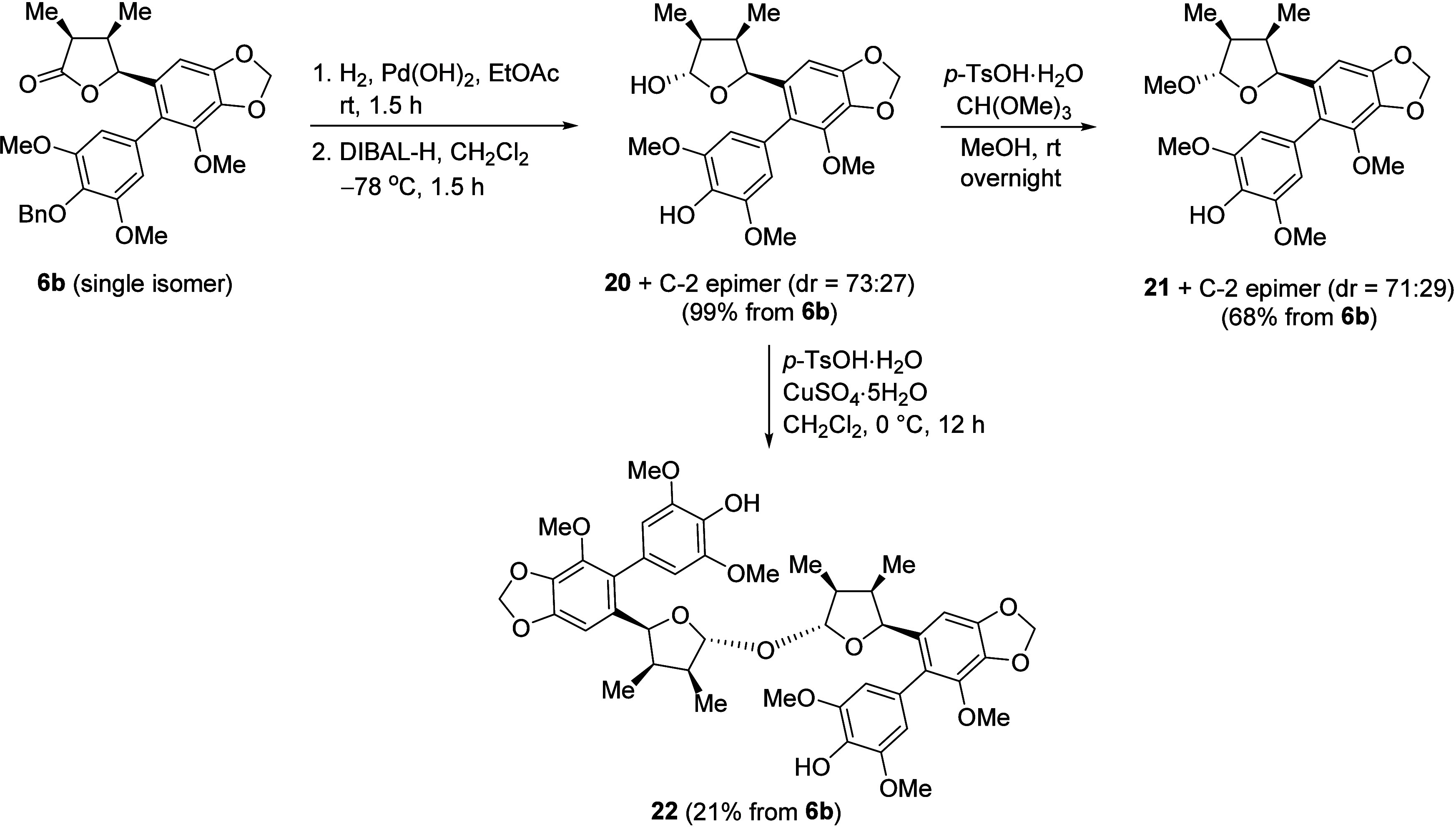
Synthesis of Previously Unreported Diastereomers of Gymnothelignan Members

The synthetic natural compounds (**1**, **2**, **3**, **4**, and **18**), along with previously unreported diastereomers (**15** and **20**) and key synthetic intermediates (**6a**, **6b**, **7a**, and **14**), were tested for their cytotoxic effects on cholangiocarcinoma (CCA) cell lines (see the Supporting Information). In the initial screening, **7a** significantly decreased cell viability in both CCA cell lines, KKU-M213 and KKU-M055. It also showed dose-dependent cytotoxicity against CCA cells (KKU-055), with an IC_50_ of 17.91 ± 0.74 μM. Conversely, **7a** had lower cytotoxicity in human cholangiocyte cells (MMNK-1), with an IC_50_ of 34.13 ± 0.54 μM (Figure 2 and Table 2). 5-Fluorouracil (5-FU) was used as a positive control, exhibiting lower IC_50_ values than **7a**.

**Figure 2:**
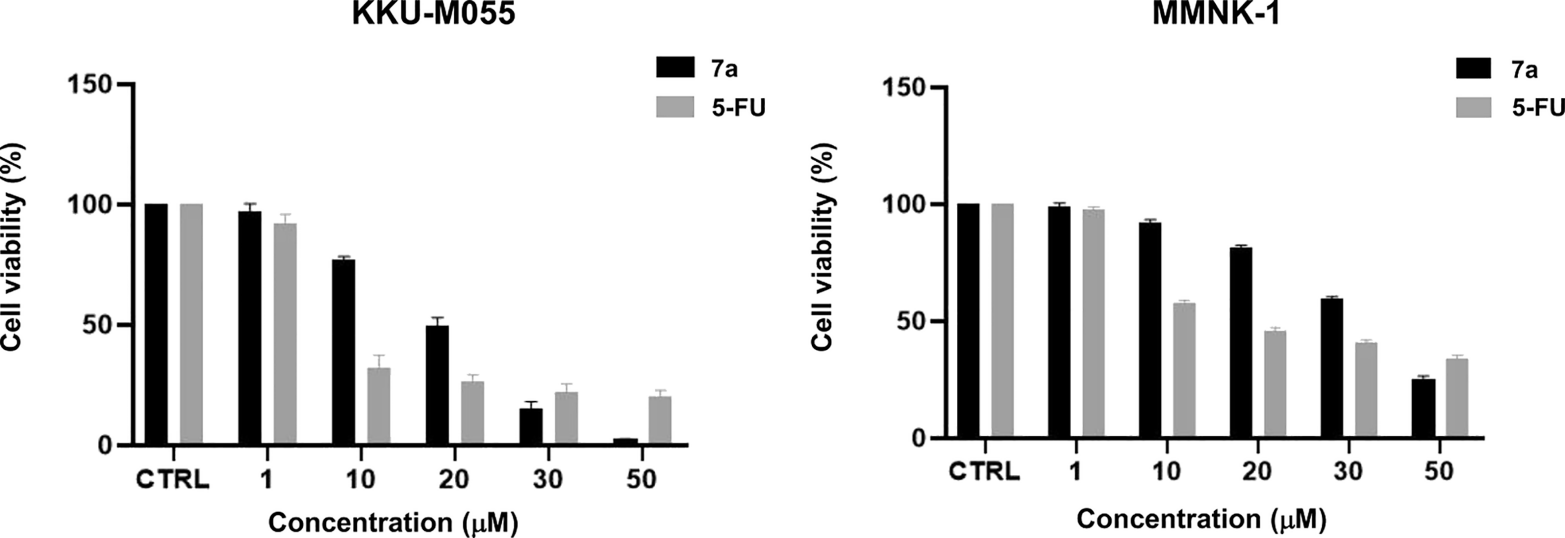
Dose-dependent cytotoxic effect of **7a** on cholangiocarcinoma (KKU-M055) and MMNK-1 (human cholangiocyte) cell lines at 72 h. 5-Fluorouracil (5-FU) is used as a positive control. Data are presented as the percentage of cell viability and are expressed as mean ± SEM.

**Table 2:** IC_50_ Values of Compound **7a** and 5-FU against Human Cholangiocarcinoma (KKU-M055) and Human Cholangiocyte (MMNK-1) Cells

	cell lines (IC_50_, μM)^a^	
compounds	KKU-M055	MMNK-1
**7a**	17.91 ± 0.74	34.13 ± 0.54
5-fluorouracil	7.65 ± 0.59	18.35 ± 0.62

a IC_50_ values are expressed as mean ± SEM (*n* = 3). 5-Fluorouracil (5-FU) was used as a positive control.

## Conclusions

In conclusion, a collective stereoselective synthesis of structurally related gymnothelignan members, i.e., gymnothelignans I, F, K (revised), and L, was accomplished based on their proposed biosynthetic pathways. Starting from the chiral eupomatilone skeleton, a key common intermediate, reduction of a carbonyl group readily gave gymnothelignan I. Further conversion of gymnothelignan I to the corresponding oxocarbenium ion by simple treatment with an acid, followed by either inter- or intramolecular nucleophilic additions provided gymnothelignans F, K (revised), and L. This work thus provides information to support the plausible biosynthetic pathway of these structurally unique lignans. Furthermore, based on similar synthetic approaches, the initially misassigned structure of gymnothelignan K and previously unreported diastereomers of gymnothelignan members were also synthesized. The cytotoxicity evaluation of the compounds synthesized in the present work revealed that the acyclic skeleton (**7a**) of the gymnothelignan family exhibited notable cytotoxicity against cholangiocarcinoma cell lines compared with 5-fluorouracil (5-FU). Although the toxicity of **7a** is less than that of the clinical drug 5-FU, further investigation into the structure–activity relationship (SAR) of this skeleton and its derivatives could benefit the organic synthesis community and drug discovery research.

## Supplementary Material



## Data Availability

The data underlying this study are available in the published article and its Supporting Information.

## Notes

Experimental procedures and characterization data; copies of ^1^H, ^13^C­{^1^H}, 2D NMR, and optical rotation spectra; and cytotoxicity data on cholangiocarcinoma cell lines (PDF)
