# Supplementary material for: Bioinspired Collective Synthesis of Gymnothelignans I, F, K, and L
Source: J Org Chem. 2026 Apr 7;91(15):5334–43. doi: 10.1021/acs.joc.5c03261 (PMC13097257; doi:10.1021/acs.joc.5c03261)
Supplement: Supplementary file 1 [file jo5c03261_si_001.pdf]

Supporting information

Bioinspired Collective Synthesis of Gymnothelignans I, F, K, and L

Nannaphat Chumsri,[a] Kamonchanok Chualong,[b] Arthit Chairoungdua,[b,c] Chutima Kuhakarn,[a,c] Pawaret Leowanawat,[a,c] Vichai Reutrakul,[a,c] and Darunee Soorukram*,[a,c]

[a] Department of Chemistry, Faculty of Science, Mahidol University, Rama VI Road, Bangkok 10400, Thailand
[b] Department of Physiology, Faculty of Science, Mahidol University, Rama VI Road, Bangkok 10400, Thailand
[c] Center of Excellence for Innovation in Chemistry (PERCH-CIC), Faculty of Science, Mahidol University, Rama VI Road, Bangkok 10400, Thailand
E-mail: darunee.soo@mahidol.ac.th. Tel: (+)-66-2-2015158. Fax: (+)-66-2-3547151

Table of Contents

	Page
General information	SI-2
Experimental procedures and characterization data	SI-3
References	SI-17
Copies (PDF files) of ^{1}H, ^{13}C{^{1}H}, 2D NMR and optical rotation spectra	SI-18
Cytotoxic effects on cholangiocarcinoma (CCA) cell lines	SI-89

General information

^{1}H NMR spectra were recorded on a 400 or 500 spectrometer in $CDCl_3$ or acetone-d_6 using residual non-deuterated solvent peaks as an internal standard and are reported in ppm. NMR data are reported as follows: s = singlet, d = doublet, t = triplet, dd = doublet of doublets, m = multiplet, br = broad, ABq = AB quartets. Proton-decoupled $^{13}C\{^{1}H\}$ NMR spectra were recorded on a 100 or 125 MHz spectrometer in $CDCl_3$ or acetone-d_6 using residual non-deuterated solvent peaks as an internal standard and are reported in ppm. Structural assignments were made with additional information from 2D NMR (COSY, HSQC, HMBC) and NOESY experiments. The IR spectra were recorded with a Bruker FT-IR spectrometer (ALPHA). The high-resolution mass spectra were recorded with a HR-TOF-MS Micromass model VQ-TOF2 mass spectrometer and a JEOL model JMS-T100LP AccuTOF LC-plus 4G. The mass spectra were recorded with a Thermo Finnigan Polaris Q mass spectrometer. Melting points were recorded with a Buchi 510 melting Point Apparatus and uncorrected. The specific rotation values were recorded with a Jasco P-2000 polarimeter. Tetrahydrofuran (THF) was distilled from sodium-benzophenone ketyl. Dichloromethane (CH_2Cl_2) and ethyl acetate (EtOAc) were distilled over calcium hydride and stored over activated molecular sieves (4 Å). Methanol (MeOH) was distilled over Mg powder. Hexanes were distilled over magnesium sulfate. Other common solvents (CH_2Cl_2, hexanes, and EtOAc) were distilled before use. All glassware including needles and syringes were oven-dried and kept in a desiccator before use. Reactions were monitored by thin-layer chromatography (TLC) and visualized by UV and a solution of $KMnO_4$. Purification of the products was carried out by column chromatography or preparative thin-layer chromatography on Merck silica gel.

Experimental procedures and characterization data

Synthesis of 6-bromo-4-methoxybenzo[*d*][1,3]dioxole (10)[1]

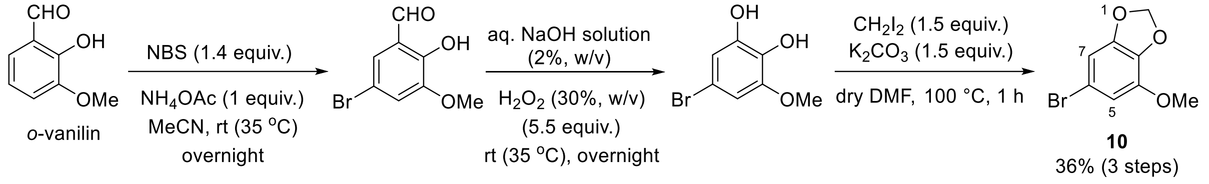

A round bottom flask, equipped with a magnetic stirring bar, was charged with NH_4OAc (1.54 g, 20 mmol) and MeCN (100 mL) then *o*-vanillin (3.04 g, 20 mmol) and NBS (4.99 g, 28 mmol) were consecutively added to the reaction mixture. After stirring at room temperature (35 °C) for overnight (16 h), the reaction mixture was quenched with a saturated aqueous $Na_2S_2O_3$ solution (30 mL) and extracted with EtOAc (3 × 30 mL). The combined organic phase was washed with brine (30 mL), dried over anhydrous Na_2SO_4, filtered, and evaporated in *vacuo*. The brominated crude product, obtained as a dark brown oil, was used in the next step without further purification. The obtained crude product was then treated with an aqueous solution of NaOH (0.86 g) in water (43 mL) followed by the addition of additional water (17 mL). An aqueous solution of H_2O_2 (30% w/v, 13.4 mL) was slowly added to the reaction mixture. The reaction was stirred at room temperature for overnight (16 h). After that, the reaction mixture was carefully acidified with an aqueous HCl solution (15 mL) and extracted with EtOAc (3 × 50 mL). The combined organic phase was washed with brine (50 mL), dried over anhydrous Na_2SO_4, and evaporated in *vacuo* to give the dark brown crude product which was used in the next step without further purification. A flame-dried round bottom flask, equipped with a magnetic stirring bar, an argon inlet, and a reflux condenser, was charged with the above obtained bromocatechol, K_2CO_3 (3.06 g, 22.1 mmol), and dry DMF (100 mL). To the reaction mixture, CH_2I_2 (1.8 mL) was added and the resulting mixture was heated at 100 °C (oil bath) for 1 h under argon atmosphere. After cooling to room temperature, the reaction mixture was quenched with a saturated aqueous $Na_2S_2O_3$ solution (30 mL) and extracted with EtOAc (3 × 100 mL). The combined organic phase was washed with brine (50 mL), dried over anhydrous Na_2SO_4, filtered, and evaporated in *vacuo* to give the crude product. Purification by column chromatography (1:4 v/v EtOAc:hexanes) gave the desired product **10** (1.67 g, 36% yield for 3 steps) as a pale pink solid. R_f 0.65 (1:4 v/v EtOAc:hexanes). ^1H NMR (400 MHz, $CDCl_3$): δ 6.68–6.66 (m, 2H), 5.97 (s, 2H), 3.88 (s, 3H) ppm. $^{13}C\{^1H\}$ NMR (100 MHz, $CDCl_3$): δ 149.6, 144.3, 135.0, 113.4, 111.1, 106.3, 102.1, 56.9 ppm.

Synthesis of 6-bromo-5-iodo-4-methoxybenzo[*d*][1,3]dioxole (11a), 5-bromo-4-iodo-7-methoxybenzo[*d*][1,3]dioxole (11b), and 5-bromo-4,6-diiodo-7-methoxybenzo[*d*][1,3]dioxole (11c)

Table S1. Optimization of the reaction conditions for regioselective iodination of **10**

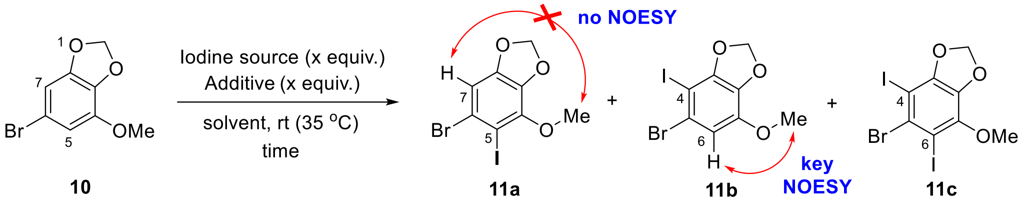

Entry	Iodine source (equiv.)	Additive (equiv.)	Solvent	Time (h)	**11a**:**11b**:**11c**:**10**[a]
1[b,c]	NIS (1.5)	*p*-TsOH (0.5)	MeCN	24	45:14:2:39
2[c]	I_2 (1.5)	CF_3CO_2Ag (1.5)	$CHCl_3$	96	25:19:3:53
3[d]	I_2 (1.5)	$AgNO_3$ (1.5)	$CHCl_3$	24	49:11:3:37
4[e]	I_2 (1.5/1/1)	$AgNO_3$ (1.5/1/1)	$CHCl_3$	15/5/2	60:24:16:0
5[f]	I_2 (3/1)	$AgNO_3$ (3/1)	$CHCl_3$	48	0:0:100:0
**6[d]**	**I_2 (1.5)**	**CF_3CO_2Ag (1.5)**	**$CHCl_3$**	**20**	**65:20:14:1**
7[c]	I_2 (1.1)	CF_3CO_2Ag (1.1)	$CHCl_3$	20	52:15:4:29
8[c]	I_2 (1.3)	CF_3CO_2Ag (1.3)	$CHCl_3$	20	54:20:5:21
9[g]	I_2 (1.5)	CF_3CO_2Ag (1.5)	$CHCl_3$	20	64:21:11:4

[a] The ratio of the products was determined by ^1H NMR analysis (CH_2 peak) of a crude mixture. [b] Using the conditions reported in ref. [2]. [c] Using 1 mmol of **10**. [d] Using 2 mmol of **10**. [e] Using 1 mmol of **10** and adding the reagents portion-wise as the time indicated. The reaction was monitored by TLC. [f] Using 4 mmol of **10** and adding the reagents portion-wise as the time indicated. The reaction was monitored by TLC. [g] Using 3 mmol of **10**.

The optimized reaction conditions (entry 6, Table S1): A flame-dried round bottom flask, equipped with a magnetic stirring bar, an argon inlet, and a rubber septum was charged with **10** (464 mg, 2 mmol) and $CHCl_3$ (20 mL). Then, silver trifluoroacetate (CF_3CO_2Ag) (665 mg, 3 mmol) and molecular I_2 (765 mg, 3 mmol) were added at room temperature. After stirring for 20 h, the solvent was removed in *vacuo* to give a crude product which was filtered through a Celite pad. The filtrate was collected and the residue was washed with CH_2Cl_2. The combined filtrate was washed with a saturated aqueous $Na_2S_2O_3$ solution (10 mL) and extracted with CH_2Cl_2 (3 × 10 mL). The combined organic phase was dried over anhydrous Na_2SO_4, filtered, and concentrated in *vacuo*. Careful purification by column chromatography (1:19 v/v CH_2Cl_2:hexanes) afforded **11a** (430 g, 60% yield), **11b** (109 mg, 15% yield), and **11c** (73 mg, 8% yield).

11a: a white solid, mp 116–119 °C (1:19 v/v CH_2Cl_2:hexanes). R_f 0.60 (1:19 v/v CH_2Cl_2:hexanes, triple run). ^1H NMR (400 MHz, $CDCl_3$): δ 6.92 (s, 1H, Ar*H*), 5.99 (s, 2H, C*H*$_2$), 4.01 (s, 3H, OC*H*$_3$) ppm. $^{13}C\{^1H\}$ NMR (100 MHz, $CDCl_3$): δ 150.7 (C), 143.9 (C), 135.7 (C), 121.4 (C), 107.9 (CH), 102.2 (CH_2), 88.7 (C) 60.3 (OCH_3) ppm. IR (ATR): ν_{max} 3094, 3020, 1596, 1463, 1087, 1033 cm^{-1}. MS: *m*/*z* (%) relative intensity 232 [(M^+ – I), 97], 230 (100). HRMS (DART)

m/z: $[M]^+$ Calcd for $C_8H_6{}^{79}BrIO_3$ 355.8545; Found 355.8544, Calcd for $C_8H_6{}^{81}BrIO_3$ 357.8525; Found 357.8526.

11b: a white solid, mp 101–103 °C (1:19 v/v CH_2Cl_2:hexanes). R_f 0.40 (1:19 v/v CH_2Cl_2:hexanes, triple run). ^{1}H NMR (400 MHz, $CDCl_3$): δ 6.90 (s, 1H, Ar*H*), 6.05 (s, 2H, C*H*$_2$), 3.88 (s, 3H, OC*H*$_3$) ppm. ^{13}C{^{1}H} NMR (100 MHz, $CDCl_3$): δ 151.5 (C), 144.5 (C), 133.7 (C), 120.5 (C), 112.3 (CH), 101.8 (CH_2), 70.2 (C) 57.1 (OCH_3) ppm. IR (ATR): ν_{max} 2960, 2904, 1628, 1469, 1433, 1415, 1102, 1033 cm^{-1}. MS: *m/z* (%) relative intensity 230 [$(M - I + H)^+$, 13], 157 (100). HRMS (APCI) *m/z*: $[M + H]^+$ Calcd for $C_8H_7{}^{79}BrIO_3$ 356.8623; Found 356.8625, Calcd for $C_8H_7{}^{81}BrIO_3$ 358.8603; Found 358.8606.

11c: a white solid, mp 172–175 °C (1:19 v/v CH_2Cl_2:hexanes). R_f 0.51 (1:19 v/v CH_2Cl_2:hexanes, triple run). ^{1}H NMR (400 MHz, $CDCl_3$): δ 6.07 (s, 2H, C*H*$_2$), 4.00 (s, 3H, OC*H*$_3$) ppm. ^{13}C{^{1}H} NMR (100 MHz, $CDCl_3$): δ 153.0 (C), 144.2 (C), 133.6 (C), 127.6 (C), 101.7 (CH_2), 88.4 (C), 70.1 (C), 60.5 (OCH_3) ppm. IR (ATR): ν_{max} 2938, 2917, 1776, 1446, 1030 cm^{-1}. MS: *m/z* (%) relative intensity 229 [$(M^+ - 2I)$, 27], 227 (100), 91 (98). HRMS (ESI-TOF) *m/z*: $[M + Na]^+$ Calcd for $C_8H_5{}^{79}BrI_2NaO_3$ 504.7409; Found 504.7405, Calcd for $C_8H_5{}^{81}BrI_2NaO_3$ 506.7389; Found 506.7387.

Synthesis of 5-[4-(benzyloxy)-3,5-dimethoxyphenyl]-6-bromo-4-methoxybenzo[*d*][1,3]dioxole (8a) and 5,6-bis[4-(benzyloxy)-3,5-dimethoxyphenyl]-4-methoxybenzo[*d*][1,3]dioxole (8b)

Table S2. Optimization of the reaction conditions for Suzuki−Miyaura cross-coupling to synthesize **8a**[a]

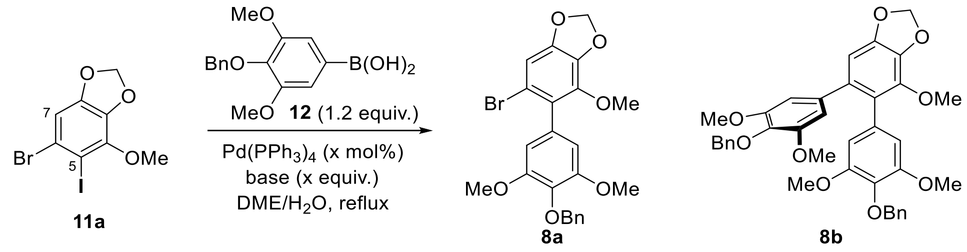

Entry	Pd (mol%)	Base (equiv.)	Time (h)	**8a**[b]	**8b**[b]	Recovery of **11a**[b]
1[3]	5	Na_2CO_3 (3.5)	10	25	-	61
2[3],c	5/5	Na_2CO_3 (3.5/3.5)	10/20	32	-	51
3[3],c	10/10	Na_2CO_3 (3.5/3.5)	10/20	28	-	61
4[3]	100	Na_2CO_3 (3.5)	12	38	-	0
5[4],c	10/10	K_3PO_4 (3.5/3.5)	10/20	49	15	0
6[4]	10	K_3PO_4 (3.5)	24	77	-	20
**7[4]**	**20**	**K_3PO_4 (7)**	**24**	**94**	**4**	**0**

[a] The optimization was carried out using 1 mmol of **11a**. [b] Isolated yield. [c] The reagents were added portion-wise as the time indicated.

The optimized reaction conditions (entry 7, Table S2): A round-bottom flask equipped with a magnetic stirring bar, an argon inlet, and a reflux condenser was charged with **11a** (234 mg, 0.66 mmol), [4-(benzyloxy)-3,5-dimethoxyphenyl]boronic acid (227 mg, 0.79 mmol), K_3PO_4 (974 mg, 4.59 mmol), $Pd(PPh_3)_4$ (151 mg, 20 mol%), DME (14 mL), and H_2O (1.4 mL). The resulting mixture was heated to reflux (oil bath) for 24 h. After cooling to room temperature, the mixture was quenched with water (20 mL) and extracted with EtOAc (3 × 20 mL). The combined organic layer was washed with water, brine, and dried over anhydrous Na_2SO_4. After filtration, the solvents were removed in *vacuo*. Purification by column chromatography (1:4 v/v EtOAc:hexanes) afforded **8a** (291 mg, 94% yield) and **8b** (16.8 mg, 4% yield).

8a: a pale orange solid, mp 147–150 °C (1:4 v/v EtOAc:hexanes). R_f 0.32 (1:4 v/v EtOAc:hexanes). ^{1}H NMR (400 MHz, $CDCl_3$): δ 7 .54–7.48 (m, 2H, 2 × Ar*H*), 7.36–7.27 (m, 3H, 3 × Ar*H*), 6.88 (s, 1H, Ar*H*), 6.43 (s, 2H, 2 × Ar*H*), 6.00 (s, 2H, CH_2), 5.08 (s, 2H, CH_2), 3.81 (s, 6H, 2 × OCH_3), 3.79 (s, 3H, OCH_3) ppm. ^{13}C{^{1}H} NMR (100 MHz, $CDCl_3$): δ 153.2 (2 × C), 149.0 (C), 142.1 (C), 138.1 (C), 137.3 (C), 136.4 (C), 132.9 (C), 129.5 (C), 128.6 (2 × CH), 128.2 (2 × CH), 127.9 (CH), 115.2 (C), 107.8 (2 × CH), 107.7 (CH), 101.9 (CH_2), 75.1 (CH_2), 60.3 (OCH_3), 56.3 (2 × OCH_3) ppm. IR (ATR): ν_{max} 2934, 1583, 1464, 1235, 1125 cm^{-1}. MS: *m*/*z* (%) relative intensity 404 [(M + Na)$^+$ – Bn, 100], 91 (27). HRMS (ESI-TOF) *m*/*z*: [M + Na]$^+$ Calcd for $C_{23}H_{21}{}^{81}BrNaO_6$ 497.0399; Found 497.0399, Calcd for $C_{23}H_{21}{}^{79}BrNaO_6$ 495.0419; Found 495.0416.

8b: a burnt orange oil. R_f 0.22 (1:4 v/v EtOAc:hexanes, double run). ^{1}H NMR (400 MHz, $CDCl_3$): δ 7.51–7.43 (m, 4H, 4 × Ar*H*), 7.36–7.32 (m, 4H, 4 × Ar*H*), 7.30–7.27 (m, 2H, 2 × Ar*H*), 6.72 (s, 1H, Ar*H*), 6.33 (s, 2H, 2 × Ar*H*), 6.27 (s, 2H, 2 × Ar*H*), 6.03 (s, 2H, CH_2), 4.96 (s, 2H, CH_2), 4.92 (s, 2H, CH_2), 3.85 (s, 3H, OCH_3), 3.62 (s, 6H, 2 × OCH_3), 3.59 (s, 6H, 2 × OCH_3) ppm. ^{13}C{^{1}H} NMR (100 MHz, $CDCl_3$): δ 153.0 (4 × C), 148.5 (C), 141.3 (C), 138.1 (C), 138.0 (C), 137.1 (C), 137.0 (C), 136.1 (C), 136.0 (C), 135.8 (C), 132.4 (C), 128.6 (2 × CH), 128.5 (2 × CH), 128.3 (2 × CH), 128.0 (2 × CH), 127.9 (2 × CH), 127.3 (C), 109.1 (2 × CH), 107.4 (2 × CH), 105.0 (CH), 101.5 (CH_2), 75.3 (2 × CH_2), 60.2 (OCH_3), 56.3 (2 × OCH_3), 56.2 (2 × OCH_3) ppm. IR (ATR): ν_{max} 2934, 1580, 1468, 1235, 1121 cm^{-1}. MS: *m/z* (%) relative intensity 635 [(M–H)$^+$, 36], 455 (63), 423 (100), 181 (60). HRMS (ESI-TOF) *m*/*z*: [M + Na]$^+$ Calcd for $C_{38}H_{36}NaO_9$ 659.2257; Found 659.2258.

Synthesis of *tert*-butyl{[(2*S*,3*S*)-2,3-dimethylpent-4-en-1-yl]oxy}dimethylsilane (13)

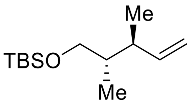

13 (dr = 93:7)

According to our previous reports,[5] a flame-dried round bottom flask, equipped with a magnetic stirring bar, was charged with (*S*)-3-[(2*S*,3*S*)-2,3-dimethylpent-4-enoyl]-4-phenyloxazolidin-2-one (dr = 93:7, 610 mg, 2.23 mmol) in mixture solvent of THF and MeOH with ratio 2:1 (22:11 mL). Then the solution of $NaBH_4$ (845 mg, 22.3 mmol) with H_2O (11 mL) was added dropwise. The reaction mixture was stirring at room temperature for 2 h. After that the reaction was quenched with H_2O (15 mL) and extracted with EtOAc (3 × 15 mL). The combined organic phase was washed with brine and dried over

anhydrous Na_2SO_4. Then removal of solvent in *vacuo*, the crude product was purified by flash column chromatography (1:19 v/v EtOAc:CH_2Cl_2) to afford the corresponding alcohol as a colorless liquid and the recovered chiral oxazolidinone as a white solid. A flame-dried round bottom flask, equipped with a magnetic stirring bar, an argon inlet, and a rubber septum was charged with the obtained crude alcohol, imidazole (304 mg, 4.46 mmol), and dry CH_2Cl_2 (5 mL). To the solution was added a solution of TBSCl (673 mg, 4.46 mmol) in dry hexanes (2.5 mL) at room temperature. The reaction mixture was allowed to stir at room temperature overnight and then quenched with a saturated aqueous $NaHCO_3$ solution (10 mL). The organic phase was collected and the aqueous phase was extracted with CH_2Cl_2 (3 × 10 mL). The combined organic phase was washed with brine, dried over anhydrous Na_2SO_4, and concentrated in *vacuo*. Purification by column chromatography (1:19 v/v EtOAc:CH_2Cl_2) gave **13** (303 mg, 59% yield, 2 steps) as a colorless liquid with a 93:7 diastereomeric ratio as determined by ^{1}H NMR (400 MHz) analysis. R_f 0.82 (1:19 v/v EtOAc in CH_2Cl_2). $[\alpha]_D^{24}$ −7.3 (c 1.0, $CHCl_3$). ^{1}H NMR (400 MHz, $CDCl_3$, interpreted only a major isomer): δ 5.75–5.66 (m, 1H, C*H*), 4.99–4.93 (m, 2H, C*H₂*), 3.49 (dd, *J* = 9.8, 6.3 Hz, 1H, C*H*H), 3.39 (dd, *J* = 9.8, 6.6 Hz, 1H, CH*H*), 2.35–2.26 (m, 1H, C*H*), 1.60–1.53 (m, 1H, C*H*), 1.00 (d, *J* = 7.0 Hz, 3H, C*H*$_3$), 0.89 (s, 9H, 3 × C*H*$_3$), 0.81 (d, *J* = 6.9 Hz, 3H, C*H*$_3$), 0.03 (s, 6H, 2 × C*H*$_3$) ppm. ^{13}C{^{1}H} NMR (100 MHz, $CDCl_3$, interpreted only a major isomer): δ 142.0 (CH), 114.1 (CH_2), 66.5 (CH_2), 40.7 (CH), 39.0 (CH), 26.1 (3 × CH_3), 18.5 (C), 18.0 (CH_3), 13.1 (CH_3), −5.2 (2 × CH_3) ppm. IR (ATR): ν_{max} 2925, 1468, 1373, 1086, 1034 cm^{-1}. MS: *m*/*z* (%) relative intensity 251 [(M + Na)$^+$, 40], 229 (24), 97 (19). HRMS (ESI-TOF) *m*/*z*: [M + Na]$^+$ Calcd for $C_{13}H_{28}NaOSi$ 251.1807; Found 251.1796.

Synthesis of (2*R*,3*S*)-1-{6-[4-(benzyloxy)-3,5-dimethoxyphenyl]-7-methoxybenzo[*d*][1,3]-dioxol-5-yl}-4-[(*tert*-butyldimethylsilyl)oxy]-2,3-dimethylbutan-1-one (14)

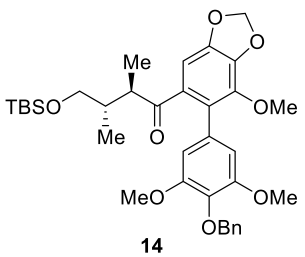

14

To a suspension of **13** (dr = 93:7) (480 mg, 2.1 mmol) and *N*-methylmorpholine-*N*-oxide (738 mg, 6.3 mmol) in CH_2Cl_2 (40 mL) were added OsO_4 (2.5% w/v in *t*-butanol, 0.1 mL, 0.1 mmol) and water (0.1 mL). ***Caution!*** *OsO_4 (2.5% w/v in t-butanol) is considered hazardous, flammable liquids (Category 2), acute oral toxicity (Category 4), acute dermal toxicity (Category 2), acute inhalation toxicity - vapors (Category 3), skin corrosion/irritation (Category 2), serious eye damage/eye irritation (Category 2), respiratory sensitization (Category 1), specific target organ toxicity (single exposure) (Category 3); target organs - respiratory system, central nervous system (CNS).* After stirring at room temperature for 14 h, $NaIO_4$ (898 mg, 4.2 mmol) was added. The stirring was continued for 1.5 h then the mixture was quenched with a saturated aqueous $Na_2S_2O_3$ solution (20 mL) and extracted with CH_2Cl_2 (3 × 20 mL). The combined organic phase was washed with brine (20 mL), dried over anhydrous Na_2SO_4, and evaporated under reduced pressure to provide aldehyde **9** which was used in the next step without purification. A flame-dried round bottom flask equipped with a magnetic stirring bar, an argon inlet, and a rubber septum was charged with **8a** (0.99 g, 2.1 mmol) and dry THF (5 mL). The solution was cooled at −78 °C then

a solution of *n*-BuLi (1.6 M in hexanes, 1.3 mL, 2.1 mmol) was added dropwise. ***Caution!*** *n-BuLi (1.6 M solution in hexanes) is considered hazardous, flammable liquids (Category 2), substances/mixtures which, in contact with water, emit flammable gases (Category 1), pyrophoric liquids (Category 1), skin corrosion/irritation (Category 1 B), serious eye damage/eye irritation (Category 1), reproductive toxicity (Category 2), specific target organ toxicity (single exposure) (Category 3); target organs - respiratory system, central nervous system (CNS).* After stirring for 10 min, a solution of **9** in dry THF (5 mL) was added dropwise. After stirring at −78 °C for 5 h, the reaction mixture was quenched with a saturated aqueous $NaHCO_3$ (20 mL) and extracted with EtOAc (3 × 15 mL). The combined organic phase was washed with brine (10 mL) and dried over anhydrous Na_2SO_4. Purification by column chromatography (1:9 v/v EtOAc:hexanes) afforded a diastereomeric mixture of **7a** and **7b** along with other minor isomers (499 mg, 38% yield for 3 steps) with a 44:43:8:5 diastereomeric ratio as determined by ^{1}H NMR (400 MHz) analysis as a pale yellow oil. A flame-dried round bottom flask, equipped with a magnetic stirring bar, an argon inlet, and a rubber septum was charged with PDC (79 mg, 0.21 mmol) and dry CH_2Cl_2 (0.8 mL). A solution of **7a** and **7b** (dr = 51:49, 41 mg, 0.07 mmol) in dry CH_2Cl_2 (1.2 mL) was added at room temperature. After stirring for 9 h, the reaction mixture was quenched with a saturated aqueous $NaHCO_3$ (10 mL) and extracted with CH_2Cl_2 (3 × 15 mL). The combined organic phase was washed with brine, dried over anhydrous Na_2SO_4, and concentrated in *vacuo*. Purification by column chromatography (1:4 v/v EtOAc:hexanes) gave **14** (30.7 mg, 70% yield) as a colorless oil. R_f 0.66 (1:4 v/v EtOAc:hexanes, double run). $[\alpha]_D^{23}$ −19.4 (c 1.1, acetone). ^{1}H NMR (400 MHz, acetone-*d*$_6$): δ 7.55−7.50 (m, 2H, 2 × Ar*H*), 7.38−7.33 (m, 2H, 2 × Ar*H*), 7.33−7.27 (m, 1H, Ar*H*), 6.67 (s, 1H, Ar*H*), 6.65−6.51 (br, 1H, Ar*H*), 6.50−6.33 (br, 1H, Ar*H*), 6.12 (s, 2H, CH_2), 5.01 (s, 2H, CH_2), 3.83 (s, 3H, OCH_3), 3.81 (br s, 6H, 2 × OCH_3), 3.39 (dd, *J* = 10.0, 5.2 Hz, 1H, C*H*H), 3.29 (dd, *J* = 10.0, 6.8 Hz, 1H, CH*H*), 2.29−2.22 (m, 1H, C*H*), 1.77−1.67 (m, 1H, C*H*), 0.85 (s, 9H, 3 × CH_3), 0.74 (d, *J* = 7.0 Hz, 3H, CH_3), 0.70 (d, *J* = 6.9 Hz, 3H, CH_3), 0.00 (s, 3H, CH_3), −0.01 (s, 3H, CH_3) ppm. ^{13}C{^{1}H} NMR (100 MHz, acetone-*d*$_6$): δ 208.7 (CO), 154.3 (2 × C), 149.6 (C), 141.5 (C), 139.9 (C), 139.4 (C), 137.8 (C), 137.7 (C), 132.5 (C), 129.0 (4 × CH), 128.5 (CH), 128.1 (C), 109.2 (2 × CH), 104.0 (CH), 103.0 (CH_2), 75.3 (CH_2), 66.0 (CH_2), 60.3 (OCH_3), 56.6 (2 × OCH_3), 48.3 (CH), 38.7 (CH), 26.4 (3 × CH_3), 18.9 (C), 16.0 (CH_3), 13.2 (CH_3), −5.2 (CH_3), −5.3 (CH_3) ppm. IR (ATR): ν_{max} 2929, 1680, 1453, 1236, 1125 cm^{-1}. MS: *m/z* (%) relative intensity 645 [(M + Na)$^+$, 55], 554 (69), 91 (100). HRMS (ESI-TOF) *m/z*: [M + Na]$^+$ Calcd for $C_{35}H_{46}NaO_8Si$ 645.2860; Found 645.2837.

Synthesis of (1*R*,2*R*,3*S*)-1-{6-[4-(benzyloxy)-3,5-dimethoxyphenyl]-7-methoxybenzo[*d*][1,3]-dioxol-5-yl}-4-[(*tert*-butyldimethylsilyl)oxy]-2,3-dimethylbutan-1-ol (7a)

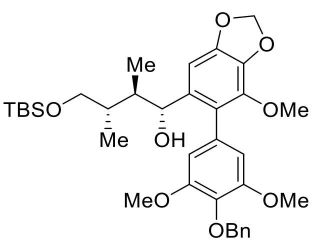

7a (dr = 95:5)

A flame-dried round bottom flask equipped with a magnetic stirring bar, and an argon inlet was charged with **14** (31.6 mg, 0.051 mmol) and $CeCl_3 \cdot 7H_2O$ (189 mg, 0.51 mmol). A solution of $NaBH_4$ (19.2 mg, 0.51

mmol) in dry MeOH (2 mL) was added at 0 °C. The reaction mixture was warmed up to room temperature and stirred for 8 h. The reaction mixture was quenched with a saturated aqueous $NaHCO_3$ (10 mL) and extracted with EtOAc (3 × 15 mL). The combined organic phase was washed with brine, dried over anhydrous Na_2SO_4, and concentrated in *vacuo*. Purification by column chromatography (1:4 v/v EtOAc:hexanes) gave **7a** (28.9 mg, 91% yield, dr = 95:5, ^{1}H NMR analysis) as a pale yellow oil. R_f 0.43 (1:4 v/v EtOAc:hexanes, double run). $[\alpha]_D^{26}$ −30.5 (c 1.9, acetone). ^{1}H NMR (400 MHz, acetone-d_6): δ 7.57−7.53 (m, 2H, 2 × Ar*H*), 7.41−7.35 (m, 2H, 2 × Ar*H*), 7.34−7.28 (m, 1H, Ar*H*), 6.79 (s, 1H, Ar*H*), 6.58 (d, *J* = 1.7 Hz, 1H, Ar*H*), 6.45 (d, *J* = 1.7 Hz, 1H, Ar*H*), 6.03, 6.02 (ABq, *J* = 2.9 Hz, 2H, C*HH*), 5.02 (s, 2H, C*H₂*), 4.44 (dd, *J* = 9.0, 4.2 Hz, 1H, C*H*), 4.07 (d, *J* = 4.2 Hz, 1H, O*H*), 3.82 (s, 3H, OCH_3), 3.81 (s, 3H, OCH_3), 3.76 (s, 3H, OCH_3), 3.56 (dd, *J* = 9.9, 5.4 Hz, 1H, CH*H*), 3.38 (dd, *J* = 9.9, 7.1 Hz, 1H, C*H*H), 2.13−2.07 (m, 1H, C*H*), 1.84−1.75 (m, 1H, C*H*), 0.87 (s, 9H, 3 × CH_3), 0.85 (d, *J* = 7.1 Hz, 3H, CH_3), 0.54 (d, *J* = 7.2 Hz, 3H, CH_3), 0.01 (s, 6H, 2 × CH_3) ppm. ^{13}C{^{1}H} NMR (100 MHz, acetone-d_6): δ 154.2 (C), 154.0 (C), 149.7 (C), 141.4 (C), 139.7 (C), 139.2 (C), 137.1 (C), 136.8 (C), 133.5 (C), 129.0 (C), 128.9 (2 × CH), 128.8 (2 × CH), 128.4 (CH), 109.9 (CH), 108.7 (CH), 102.1 (CH_2), 101.9 (CH), 75.3 (CH_2), 72.3 (CH), 66.2 (CH_2), 60.1 (OCH_3), 56.6 (2 × OCH_3), 44.4 (CH), 37.0 (CH), 26.4 (3 × CH_3), 18.9 (C), 15.7 (CH_3), 13.2 (CH_3), −5.2 (2 × CH_3) ppm. IR (ATR): ν_{max} 3378, 2929, 1503, 1124 cm^{-1}. MS: *m/z* (%) relative intensity 556 [(M – Bn + Na)$^+$, 100], 541 (54). HRMS (ESI-TOF) *m/z*: [M + Na]$^+$ Calcd for $C_{35}H_{48}NaO_8Si$ 647.3016; Found 647.3002.

Synthesis of (3*S*,4*R*,5*R*) and (3*S*,4*R*,5*S*)-5-{6-[4-(benzyloxy)-3,5-dimethoxyphenyl]-7-methoxybenzo[*d*][1,3]dioxol-5-yl}-3,4-dimethyldihydrofuran-2(3*H*)-ones (6a and 6b)

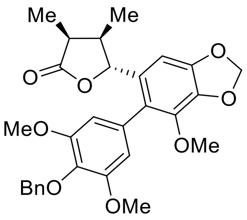

6a

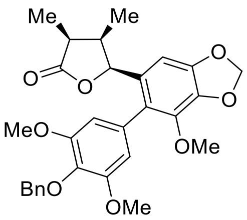

6b

A flame-dried round bottom flask, equipped with a magnetic stirring bar, and an argon inlet was charged with a mixture of **7a** and **7b** (dr = 54:43:3, 169 mg, 0.27 mmol) and dry THF (7 mL). A solution of TBAF (103 mg, 0.33 mmol) in dry THF (16 mL) was added at room temperature. After stirring for 2 h, the reaction mixture was quenched with a saturated aqueous $NaHCO_3$ (15 mL) and extracted with EtOAc (3 × 20 mL). The combined organic phase was washed with brine, dried over anhydrous Na_2SO_4, and concentrated in *vacuo*. The obtained crude product (173 mg) was dissolved in dry CH_2Cl_2 (11 mL) under an argon atmosphere. To the resulting solution were added sequentially (diacetoxyiodo)benzene (DIB, 328 mg, 1.02 mmol) and (2,2,6,6-tetramethylpiperidin-1-yl)oxyl (TEMPO, 10.7 mg, 0.07 mmol) at room temperature. After stirring for 3.5 h, the resulting suspension was quenched with a saturated aqueous $Na_2S_2O_3$ solution (10 mL) and extracted with CH_2Cl_2 (3 × 10 mL). The combined organic phase was washed with a saturated aqueous $NaHCO_3$, brine, dried over anhydrous Na_2SO_4, and concentrated in *vacuo*. Purification by column chromatography (2:3 v/v EtOAc:hexanes) gave **6a** (47.2 mg, 34% yield) and **6b** (59.4 mg, 43% yield) for two steps.

6a: a colorless oil. R_f 0.50 (2:3 v/v EtOAc:hexanes, double run). $[\alpha]_D^{23}$ −23.1 (c 0.5, acetone). ^{1}H NMR (400 MHz, $CDCl_3$): δ 7.53−7.48 (m, 2H, 2 × Ar*H*), 7.35−7.28 (m, 3H, 3 × Ar*H*), 6.59 (s, 1H, Ar*H*), 6.42 (d, *J* = 1.7 Hz, 1H, Ar*H*), 6.35 (d, *J* = 1.7 Hz, 1H, Ar*H*), 5.99, 5.98 (ABq, *J* = 2.9 Hz, 2H, C*HH*), 5.09 (s, 2H, C*H₂*), 4.89 (d, *J* = 4.9 Hz, 1H, C*H*), 3.82 (s, 3H, OC*H₃*), 3.81 (s, 3H, OC*H₃*), 3.79 (s, 3H, OC*H₃*), 2.79−2.71 (m, 1H, C*H*), 2.41−2.32 (m, 1H, C*H*), 1.04 (d, *J* = 7.5 Hz, 3H, C*H₃*), 0.61 (d, *J* = 7.1 Hz, 3H, C*H₃*) ppm. ^{13}C{^{1}H} NMR (100 MHz, $CDCl_3$): δ 179.9 (CO), 153.7 (C), 153.5 (C), 149.1 (C), 141.3 (C), 137.9 (C), 136.9 (C), 136.1 (C), 131.5 (C), 131.1 (C), 128.7 (2 × CH), 128.3 (2 × CH), 128.0 (CH), 127.8 (C), 108.4 (CH), 107.0 (CH), 101.6 (CH_2), 99.9 (CH), 83.1 (CH), 75.0 (CH_2), 60.2 (OCH_3), 56.4 (OCH_3), 56.3 (OCH_3), 41.8 (CH), 37.4 (CH), 13.4 (CH_3), 10.1 (CH_3) ppm. IR (ATR): ν_{max} 2920, 1775, 1460, 1126 cm^{-1}. MS: *m/z* (%) relative intensity 529 [(M + Na)$^+$, 61], 438 (100), 394 (34). HRMS (ESI-TOF) *m/z*: [M + Na]$^+$ Calcd for $C_{29}H_{30}NaO_8$ 529.1838; Found 529.1839.

6b: a colorless oil. R_f 0.60 (2:3 v/v EtOAc:hexanes, double run). $[\alpha]_D^{23}$ −20.3 (c 1.2, acetone). ^{1}H NMR (400 MHz, $CDCl_3$): δ 7.52−7.47 (m, 2H, 2 × Ar*H*), 7.35−7.28 (m, 3H, 3 × Ar*H*), 6.75 (s, 1H, Ar*H*), 6.40 (d, *J* = 1.6 Hz, 1H, Ar*H*), 6.27 (d, *J* = 1.6 Hz, 1H, Ar*H*), 5.99 (s, 2H, C*H₂*), 5.23 (d, *J* = 5.0 Hz, 1H, C*H*), 5.11 (s, 2H, C*H₂*), 3.82 (s, 3H, OC*H₃*), 3.80 (s, 3H, OC*H₃*), 3.78 (s, 3H, OC*H₃*), 2.67−2.60 (m, 1H, C*H*), 2.08−2.00 (m, 1H, C*H*), 1.09 (d, *J* = 7.2 Hz, 3H, C*H₃*), 0.55 (d, *J* = 7.4 Hz, 3H, C*H₃*) ppm. ^{13}C{^{1}H} NMR (100 MHz, $CDCl_3$): δ 178.7 (CO), 153.7 (C), 153.5 (C), 148.8 (C), 141.1 (C), 137.9 (C), 136.5 (C), 136.0 (C), 131.3 (C), 129.1 (C), 128.6 (2 × CH), 128.2 (2 × CH), 127.9 (CH), 126.4 (C), 107.8 (CH), 106.3 (CH), 101.5 (CH_2), 101.2 (CH), 80.6 (CH), 74.9 (CH_2), 60.2 (OCH_3), 56.4 (OCH_3), 56.3 (OCH_3), 40.8 (CH), 39.1 (CH), 10.0 (CH_3), 9.9 (CH_3) ppm. IR (ATR): ν_{max} 2935, 1771, 1453, 1232 cm^{-1}. MS: *m/z* (%) relative intensity 529 [(M + Na)$^+$, 51], 438 (100), 300 (49), 254 (71), 91 (54). HRMS (ESI-TOF) *m/z*: [M + Na]$^+$ Calcd for $C_{29}H_{30}NaO_8$ 529.1838; Found 529.1828.

Synthesis of gymnothelignan I (1)

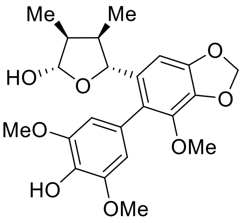

1 + C-2 epimer
(dr = 86:14)

A flame-dried round bottom flask equipped with a magnetic stirring bar, an argon inlet, and a rubber septum was charged with **6a** (38.3 mg, 0.08 mmol), Pd/C (10% w/w, 8.5 mg, 0.08 mmol), and dry EtOAc (3 mL). The argon inlet was replaced by a hydrogen balloon, and the reaction mixture was stirred at room temperature for 30 min. ***Caution!*** *Hydrogen is classified as a GHS Flammable Gas (Category 1).* The resulting mixture was filtered through a Celite pad then the residue was eluted with EtOAc (10 mL) and evaporated under reduced pressure to provide the lactone crude product which was used in the next step without chromatographic purification. A flame-dried round bottom flask, equipped with a magnetic stirring bar, an argon inlet and a rubber septum was charged with the lactone crude product (37.3 mg, 0.09 mmol) and dry CH_2Cl_2 (1 mL) and the resulting solution was cooled at −78 °C. A solution of DIBAL–H (1 M in hexanes) (0.45 mL, 0.45 mmol) was added dropwise. ***Caution!*** *DIBAL-H (1M solution in hexanes) is considered hazardous, flammable liquids*

(Category 2), substances/mixtures which, in contact with water, emit flammable gases (Category 1), pyrophoric liquids (Category 1), skin corrosion/irritation (Category 1 A), serious eye damage/eye irritation (Category 1), reproductive toxicity (Category 2), specific target organ toxicity (single exposure) (Category 3); target organs - respiratory system, central nervous system (CNS). The reaction was allowed to stir at −78 °C for 1.5 h then it was quenched with MeOH at −78 °C, allowed to warm to room temperature, and extracted with EtOAc (3 ×10 mL). The combined organic layer was washed with water, brine, and dried over anhydrous Na_2SO_4. Purification by column chromatography (3:2 v/v EtOAc:hexanes) gave **1** and its C-2 epimer (37.5 mg, 99% yield, dr = 86:14, ^{1}H NMR analysis) as a light yellow gum. R_f 0.49 (3:2 v/v EtOAc:hexanes, double run). $[\alpha]_D^{25}$ −4.4 (c 0.1, acetone). ^{1}H NMR (400 MHz, acetone-d_6, integrated equally for both diastereomers, **1** marked*): δ 7.20* (s, 1H, O*H*), 7.16* (s, 1H, Ar*H*), 6.68 (s, 1H, Ar*H*), 6.46 (d, *J* = 1.6 Hz, 1H, Ar*H*), 6.44* (d, *J* = 1.6 Hz, 1H, Ar*H*), 6.42* (d, *J* = 1.6 Hz, 1H, Ar*H*), 6.00* (s, 2H, C*H$_2$*), 5.52 (dd, *J* = 4.5, 4.3 Hz, 1H, C*H*), 5.41* (d, *J* = 4.2 Hz, 1H, O*H*), 5.04* (dd, *J* = 4.1, 1.9 Hz, 1H, C*H*), 4.94 (d, *J* = 4.3 Hz, 1H, O*H*), 4.69 (d, *J* = 4.0 Hz, 1H, C*H*), 4.48* (d, *J* = 8.0 Hz, 1H, C*H*), 3.82 (s, 3H, OC*H$_3$*), 3.81 (s, 3H, OC*H$_3$*), 3.81* (s, 6H, 2 × OC*H$_3$*), 3.74 (s, 3H, OC*H$_3$*), 3.73* (s, 3H, OC*H$_3$*), 2.40−2.31* (m, 1H, C*H*), 2.31−2.25 (m, 1H, C*H*), 2.12−2.08* (m, 1H, C*H*), 2.01−1.96 (m, 1H, C*H*), 0.89 (d, *J* = 7.2 Hz, 3H, C*H$_3$*), 0.77* (d, *J* = 7.3 Hz, 3H, C*H$_3$*), 0.72 (d, *J* = 7.2 Hz, 3H, C*H$_3$*), 0.69* (d, *J* = 7.0 Hz, 3H, C*H$_3$*) ppm. Some proton peaks of minor isomer overlap with **1**. ^{13}C{^{1}H} NMR (100 MHz, acetone-d_6, **1** marked*): δ 149.4* (C), 148.4* (C), 148.3* (C), 141.4* (C), 137.2* (C), 137.1* (C), 136.0* (C), 129.6* (C), 127.6* (C), 110.2* (CH), 110.1 (CH), 108.5* (CH), 108.4 (CH), 104.4* (CH), 103.4* (CH), 102.0* (CH_2), 101.5 (CH), 101.4 (CH), 84.8 (CH), 83.8* (CH), 60.1* (OCH_3), 56.8* (2 × OCH_3), 45.2* (CH), 44.9 (CH), 44.2* (CH), 40.6 (CH), 15.5 (CH_3), 12.3* (CH_3), 11.3* (CH_3), 9.4 (CH_3) ppm. Some carbon peaks of minor isomer were not interpreted due to low intensity. IR (ATR): ν_{max} 3407, 2926, 1612, 1475, 1112 cm^{-1}. MS: *m/z* (%) relative intensity 441 [(M + Na)$^+$, 21], 437 (27), 315 (100), 102 (40). HRMS (ESI-TOF) *m/z*: [M + Na]$^+$ Calcd for $C_{22}H_{26}NaO_8$ 441.1525; Found 441.1526.

Synthesis of gymnothelignan F (2) and 2-*epi*-gymnothelignan F (15)

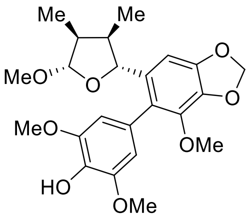

2

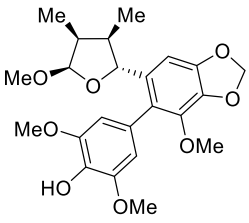

15

Following the synthetic sequences for the synthesis of **1**, debenzylation of **6a** (23.5 mg, 0.046 mmol) using H_2, Pd/C (10% w/w, 4.9 mg, 0.046 mmol), and dry EtOAc (1.9 mL) gave the lactone crude product, which was subjected to the reduction using DIBAL−H (1 M in hexanes) (0.25 mL, 0.25 mmol) at −78 °C for 1.5 h to provide the crude mixture of **1** and its C-2 epimer, which was used in the next step without further purification. A flame-dried round bottom flask equipped with a magnetic stirring bar, an argon inlet, and a rubber septum was charged with a mixture of **1** and its C-2 epimer (dr = 86:14) and *p*-TsOH·H_2O (10 mg, 0.05 mmol) in dry MeOH (2 mL) followed by the addition of a solution of $CH(OMe)_3$ (0.16 mL). After stirring at room

temperature overnight (16 h), the reaction mixture was quenched with H_2O (5 mL) and extracted with EtOAc (3 × 10 mL). The combined organic layer was washed with brine and dried over anhydrous Na_2SO_4. Purification by column chromatography (2:3 v/v EtOAc:hexanes) gave **2** (11.5 mg, 58% yield) and **15** (2.2 mg, 11% yield) from **6a**.

2: a pale yellow oil. R_f 0.53 (2:3 v/v EtOAc:hexanes). $[\alpha]_D^{24}$ −1.9 (c 0.2, acetone). ^{1}H NMR (400 MHz, acetone-d_6): δ 7.23 (s, 1H, O*H*), 6.86 (s, 1H, Ar*H*), 6.44 (d, *J* = 1.6 Hz, 1H, Ar*H*), 6.42 (d, *J* = 1.6 Hz, 1H, Ar*H*), 6.02 (s, 2H, CH_2), 4.55 (s, 1H, C*H*), 4.52 (d, *J* = 8.9 Hz, 1H, C*H*), 3.82 (s, 3H, OCH_3), 3.80 (s, 3H, OCH_3), 3.74 (s, 3H, OCH_3), 3.38 (s, 3H, OCH_3), 2.40−2.31 (m, 1H, C*H*), 2.18−2.11 (m, 1H, C*H*), 0.76 (d, *J* = 7.4 Hz, 3H, CH_3), 0.72 (d, *J* = 7.0 Hz, 3H, CH_3) ppm. ^{13}C{^{1}H} NMR (100 MHz, acetone-d_6): δ 149.6 (C), 148.3 (C), 148.2 (C), 141.5 (C), 137.2 (C), 136.4 (C), 136.0 (C), 130.1 (C), 127.5 (C), 111.6 (CH), 110.1 (CH), 108.4 (CH), 102.8 (CH), 102.1 (CH_2), 84.2 (CH), 60.1 (OCH_3), 56.8 (OCH_3), 56.7 (OCH_3), 55.1 (OCH_3), 44.5 (CH), 44.1 (CH), 11.9 (CH_3), 11.4 (CH_3) ppm. IR (ATR): ν_{max} 3419, 2957, 2927, 1611, 1474, 1111 cm^{-1}. MS: *m/z* (%) relative intensity 455 [$(M + Na)^+$, 74], 315 (72), 257 (100), 254 (47). HRMS (ESI-TOF) *m/z*: $[M + Na]^+$ Calcd for $C_{23}H_{28}NaO_8$ 455.1682; Found 455.1658.

15: a pale yellow oil. R_f 0.61 (2:3 v/v EtOAc:hexanes). $[\alpha]_D^{24}$ −45.2 (c 0.22, acetone). ^{1}H NMR (400 MHz, acetone-d_6): δ 7.23 (s, 1H, O*H*), 6.70 (s, 1H, Ar*H*), 6.46 (d, *J* = 1.6 Hz, 1H, Ar*H*), 6.44 (d, *J* = 1.6 Hz, 1H, Ar*H*), 6.01, 6.00 (ABq, *J* = 4.2 Hz, 2H, C*HH*), 4.98 (d, *J* = 4.9 Hz, 1H, C*H*), 4.55 (d, *J* = 4.1 Hz, 1H, C*H*), 3.83 (s, 3H, OCH_3), 3.81 (s, 3H, OCH_3), 3.74 (s, 3H, OCH_3), 3.24 (s, 3H, OCH_3), 2.42−2.34 (m, 1H, C*H*), 2.02−1.94 (m, 1H, C*H*), 0.86 (d, *J* = 7.2 Hz, 3H, CH_3), 0.65 (d, *J* = 7.2 Hz, 3H, CH_3) ppm. ^{13}C{^{1}H} NMR (100 MHz, acetone-d_6): δ 149.4 (C), 148.6 (C), 148.5 (C), 142.0 (C), 137.9 (C), 137.0 (C), 136.1 (C), 128.6 (C), 127.6 (C), 110.1 (CH), 108.3 (CH), 108.2 (CH), 102.1 (CH_2), 101.4 (CH), 85.1 (CH), 60.1 (OCH_3), 56.8 (2 × OCH_3), 54.9 (OCH_3), 44.4 (CH), 40.4 (CH), 15.4 (CH_3), 9.2 (CH_3) ppm. IR (ATR): ν_{max} 3450, 2929, 1611, 1519, 1113 cm^{-1}. MS: *m/z* (%) relative intensity 455 [$(M + Na)^+$, 100], 353 (14). HRMS (ESI-TOF) *m/z*: $[M + Na]^+$ Calcd for $C_{23}H_{28}NaO_8$ 455.1682; Found 455.1679.

Synthesis of initially misassigned gymnothelignan K (17)

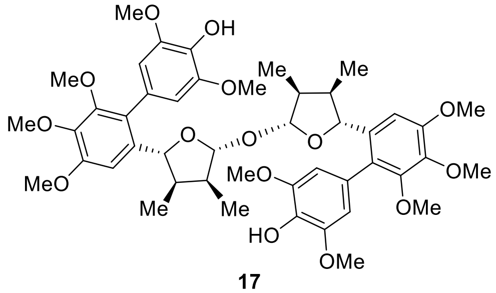

17

A flame-dried round bottom flask equipped with a magnetic stirring bar, an argon inlet, and a rubber septum was charged with a mixture of **16** and its C-2 epimer (dr = 85:15, 28.1 mg, 0.065 mmol), *p*-TsOH·H_2O (12.3 mg, 0.065 mmol), and $CuSO_4·5H_2O$ (48.5 mg, 0.19 mmol) in CH_2Cl_2 (1.7 mL) at 0 °C. After stirring for 12 h, the resulting mixture was quenched with saturated aqueous $NaHCO_3$ (10 mL) and extracted with CH_2Cl_2 (3 × 10 mL). The combined organic layers were washed with a saturated aqueous NaCl solution (10 mL), dried over anhydrous Na_2SO_4, and concentrated in *vacuo*. Purification by column chromatography (3:2 v/v EtOAc:hexanes) gave a

mixture of **17** and its C-2 epimer (9.4 mg, 56% yield) (dr = 76:24, ^{1}H NMR analysis) as a pale yellow gum.

17 (single isomer) could be partially separated (4.5 mg, 27% yield) by column chromatography (3:7 v/v EtOAc:hexanes): a pale yellow powder. R$_f$ 0.54 (3:2 v/v EtOAc:hexanes, double run). $[\alpha]_D^{24}$ +33.3 (c 0.14, acetone). ^{1}H NMR (400 MHz, acetone-d_6): δ 7.22 (s, 2H, 2 × O*H*), 7.06 (s, 2H, 2 × Ar*H*), 6.47 (br d, *J* = 1.9 Hz, 4H, 4 × Ar*H*), 5.15 (s, 2H, 2 × C*H*), 4.62 (d, *J* = 9.0 Hz, 2H, 2 × C*H*), 3.89 (s, 6H, 2 × OC*H*$_3$), 3.83 (s, 6H, 2 × OC*H*$_3$), 3.82 (s, 12H, 4 × OC*H*$_3$), 3.59 (s, 6H, 2 × OC*H*$_3$), 2.57–2.48 (m, 2H, 2 × C*H*), 2.43–2.36 (m, 2H, 2 × C*H*), 0.86 (d, *J* = 7.3 Hz, 6H, 2 × C*H*$_3$), 0.77 (d, *J* = 6.9 Hz, 6H, 2 × C*H*$_3$) ppm. ^{13}C{^{1}H} NMR (100 MHz, acetone-d_6): δ 154.0 (2 × C), 151.9 (2 × C), 148.3 (2 × C), 148.2 (2 × C), 142.7 (2 × C), 136.9 (2 × C), 136.0 (2 × C), 131.0 (2 × C), 127.5 (2 × C), 110.0 (2 × CH), 108.5 (2 × CH), 107.7 (2 × CH), 106.3 (2 × CH), 84.0 (2 × CH), 61.3 (2 × OCH$_3$), 60.9 (2 × OCH$_3$), 56.8 (4 × OCH$_3$), 56.4 (2 × OCH$_3$), 44.8 (2 × CH), 44.1 (2 × CH), 11.7 (2 × CH$_3$), 11.5 (2 × CH$_3$) ppm. IR (ATR): ν_{max} 3428, 2925, 1461, 1102 cm^{-1}. MS: *m/z* (%) relative intensity 873 [(M + Na)$^+$, 79], 457 (100). HRMS (ESI-TOF) *m/z*: [M + Na]$^+$ Calcd for $C_{46}H_{58}NaO_{15}$ 873.3673; Found 873.3677.

Synthesis of gymnothelignan K (3) and gymnothelignan L (4)

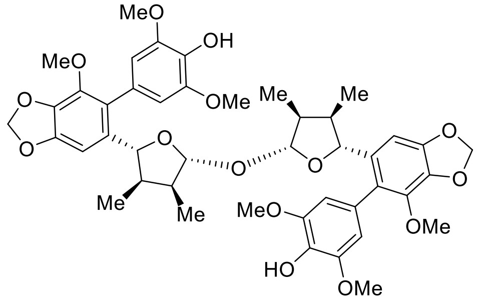

gymnothelignan K (revised) (**3**)

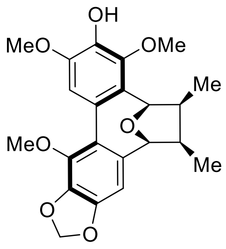

gymnothelignan L (**4**)

Following the synthetic sequences for the synthesis of **1**, debenzylation of **6a** (38 mg, 0.075 mmol) using H_2, Pd/C (10% w/w, 8 mg, 0.075 mmol), and dry EtOAc (2.9 mL) gave the lactone crude product, which was subjected to the reduction using DIBAL–H (1 M in hexanes) (0.45 mL, 0.45 mmol) at −78 °C for 1.5 h to provide the crude mixture of **1** and its C-2 epimer, which was used in the next step without further purification. A flame-dried round bottom flask equipped with a magnetic stirring bar, an argon inlet, and a rubber septum was charged with a mixture of **1** and its C-2 epimer (dr = 86:14), *p*-TsOH·H_2O (18.5 mg, 0.097 mmol), $CuSO_4$·5H_2O (72.7 mg, 0.291 mmol) and dry CH_2Cl_2 (1.1 mL) at 0 °C. After stirring for 12 h, the resulting mixture was quenched with saturated aqueous $NaHCO_3$ (10 mL) and extracted with CH_2Cl_2 (3 × 10 mL). The combined organic layers were washed with brine and dried over anhydrous Na_2SO_4. Purification by column chromatography (3:2 v/v EtOAc:hexanes) gave **3** (19 mg, 61% yield) (dr = 91:9, ^{1}H NMR analysis) and **4** (2.3 mg, 8% yield) from **6a**.

3 (single isomer) could be partially separated (7.5 mg, 24% yield) by using column chromatography (3:7 v/v EtOAc:hexanes): a white solid. R$_f$ 0.26 (3:7 v/v EtOAc: hexanes, triple run). $[\alpha]_D^{21}$ −10.9 (c 0.09, MeOH). ^{1}H NMR (400 MHz, acetone-d_6): δ 7.21 (s, 2H, 2 × O*H*), 6.90 (s, 2H, 2 × Ar*H*), 6.44 (s, 4H, 4 × Ar*H*), 6.03, 6.01 (ABq, *J* = 0.9 Hz, 4H, 2 × C*HH*), 5.07 (s, 2H,

2 × C*H*), 4.55 (d, *J* = 8.8 Hz, 2H, 2 × C*H*), 3.82 (s, 6H, 2 × OCH_3), 3.81 (s, 6H, 2 × OCH_3), 3.74 (s, 6H, 2 × OCH_3), 2.52–2.43 (m, 2H, 2 × C*H*), 2.40–2.32 (m, 2H, 2 × C*H*), 0.85 (d, *J* = 7.4 Hz, 6H, 2 × CH_3), 0.80 (d, *J* = 6.9 Hz, 6H, 2 × CH_3) ppm. $^{13}C\{^1H\}$ NMR (100 MHz, acetone-d_6): δ 149.6 (2 × C), 148.4 (2 × C), 148.2 (2 × C), 141.5 (2 × C), 137.3 (2 × C), 136.2 (2 × C), 136.0 (2 × C), 130.3 (2 × C), 127.4 (2 × C), 110.0 (2 × CH), 108.5 (2 × CH), 106.0 (2 × CH), 102.8 (2 × CH), 102.2 (2 × CH_2), 84.3 (2 × CH), 60.1 (2 × OCH_3), 56.8 (4 × OCH_3), 44.7 (2 × CH), 44.1 (2 × CH), 11.9 (2 × CH_3), 11.5 (2 × CH_3) ppm. IR (ATR): ν_{max} 2961, 1781, 1475, 1217, 1112 cm^{-1}. MS: *m/z* (%) relative intensity 841 [$(M + Na)^+$, 26], 401 (23), 315 (100). HRMS (ESI-TOF) *m/z*: $[M + Na]^+$ Calcd for $C_{44}H_{50}NaO_{15}$ 841.3047; Found 841.3046.

4: a pale yellow oil. R_f 0.54 (3:7 v/v EtOAc:hexanes). $[\alpha]_D^{23}$ −1.9 (c 0.2, acetone). ^1H NMR (400 MHz, acetone-d_6): δ 7.64 (s, 1H, O*H*), 7.27 (s, 1H, Ar*H*), 6.53 (s, 1H, Ar*H*), 6.05 (d, *J* = 1.0 Hz, 1H, C*H*H), 5.99 (d, *J* = 1.0 Hz, 1H, CH*H*), 5.11 (d, *J* = 1.8 Hz, 1H, C*H*), 4.49 (d, *J* = 5.5 Hz, 1H, C*H*), 3.88 (s, 3H, OCH_3), 3.84 (s, 3H, OCH_3), 3.71 (s, 3H, OCH_3), 2.41−2.32 (m, 1H, C*H*), 2.29−2.22 (m, 1H, C*H*), 1.06 (d, *J* = 5.1 Hz, 3H, CH_3), 1.05 (d, *J* = 5.1 Hz, 3H, CH_3) ppm. $^{13}C\{^1H\}$ NMR (100 MHz, acetone-d_6): δ 147.8 (C), 146.7 (C), 144.6 (C), 144.0 (C), 141.1 (C), 139.6 (C), 139.0 (C), 130.9 (C), 124.9 (C), 124.1 (C), 113.4 (CH), 103.9 (CH), 102.4 (CH_2), 90.8 (CH), 85.4 (CH), 60.5 (OCH_3), 60.4 (OCH_3), 56.5 (OCH_3), 49.7 (CH), 42.7 (CH), 14.1 (CH_3), 13.9 (CH_3) ppm. IR (ATR): ν_{max} 3355, 2930, 1616, 1506, 1101 cm^{-1}. MS: *m/z* (%) relative intensity 423 [$(M + Na)^+$, 68], 331 (87), 315 (98), 311 (69), 123 (95). HRMS (ESI-TOF) *m/z*: $[M + Na]^+$ Calcd for $C_{22}H_{24}NaO_7$ 423.1420; Found 423.1428.

Synthesis of 20

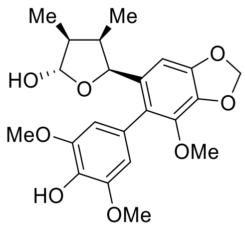

20 + C-2 epimer
(dr = 73:27)

Following the synthetic sequences for the synthesis of **1**, debenzylation of **6b** (59 mg, 0.116 mmol) using H_2, $Pd(OH)_2$ (20% wt, 16.8 mg, 0.12 mmol), and dry EtOAc (4.7 mL) gave the lactone crude product, which was subjected to the reduction using DIBAL–H (1 M in hexanes) (0.6 mL, 0.6 mmol) at −78 °C for 1.5 h to provide the crude mixture of **20** and its C-2 epimer. Purification by column chromatography (3:2 v/v EtOAc:hexanes) gave a mixture of **20** and its C-2 epimer (48 mg, 99% yield, dr = 73:27, ^1H NMR analysis) as a colorless oil. R_f 0.54 (3:2 v/v EtOAc:hexanes). $[\alpha]_D^{22}$ −10.1 (c 0.3, acetone). ^1H NMR (400 MHz, acetone-d_6, integrated equally for both diastereomers, **20** marked*): δ 7.25* (s, 1H, O*H*), 7.23 (s, 1H, O*H*), 7.02 (s, 1H, Ar*H*), 6.81* (s, 1H, Ar*H*), 6.45* (d, *J* = 1.6 Hz, 1H, Ar*H*), 6.44 (d, *J* = 1.6 Hz, 1H, Ar*H*), 6.36* (d, *J* = 1.6 Hz, 1H, Ar*H*), 6.35 (d, *J* = 1.6 Hz, 1H, Ar*H*), 6.01*, 6.00* (ABq, *J* = 1.0 Hz, 2H, C*HH*), 5.99 (d, *J* = 1.0 Hz, 1H, CH*H*), 5.25−5.21 (m, 2H, C*H* and OH), 5.10−5.08* (m, 3H, 2 × C*H* and O*H*), 4.85 (d, *J* = 6.1 Hz, 1H, C*H*), 3.83* (s, 3H, OCH_3), 3.82 (s, 3H, OCH_3), 3.79* (s, 3H, OCH_3), 3.74* (s, 3H, OCH_3), 2.18−2.12 (m, 1H, C*H*), 2.02−1.99* (m, 1H, C*H*), 1.93−1.83* (m, 1H, C*H*), 0.95 (d, *J* = 7.1 Hz, 3H, CH_3), 0.94* (d, *J* = 7.1 Hz, 3H, CH_3), 0.69 (d, *J* = 7.5 Hz, 3H, CH_3), 0.51* (d, *J* = 7.4 Hz, 3H, CH_3) ppm. Some proton peaks of minor isomer overlap with **20**. $^{13}C\{^1H\}$ NMR (100 MHz, acetone-d_6, **20** marked*): δ 149.0* (C), 148.9 (C), 148.6* (C), 148.4* (C), 148.3 (C), 142.1* (C), 136.8* (C), 136.1* (C),

136.0 (C), 135.7 (C), 134.3* (C), 127.9* (C), 127.8 (2 × C), 127.7* (C), 109.3* (CH), 109.1 (CH), 107.9* (CH), 107.7 (CH), 104.1* (CH), 103.7 (CH), 102.5* (CH), 102.0* (CH_2), 101.9 (CH_2), 99.9 (CH), 83.2 (CH), 81.0* (CH), 60.2* (OCH_3), 56.8* (2 × OCH_3), 56.7 (2 × OCH_3), 46.7* (CH), 43.0 (CH), 41.9* (CH), 40.5 (CH), 12.3* (CH_3), 12.2 (CH_3), 10.4* (CH_3), 10.3 (CH_3) ppm. Some carbon peaks of minor isomer were not interpreted due to low intensity. IR (ATR): ν_{max} 3442, 2924, 1772, 1610, 1451, 1053 cm^{-1}. MS: *m/z* (%) relative intensity 441 [$(M + Na)^+$, 100], 315 (42), 257 (14). HRMS (ESI-TOF) *m/z*: $[M + Na]^+$ Calcd for $C_{22}H_{26}NaO_8$ 441.1525; Found 441.1520.

Synthesis of 21

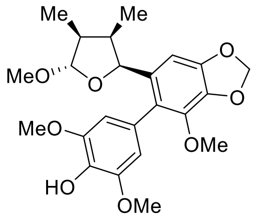

21 + C-2 epimer
(dr = 71:29)

Following the synthetic sequences for the synthesis of **1**, debenzylation of **6b** (59.6 mg, 0.118 mmol) using H_2, $Pd(OH)_2$ (20% wt, 16.6 mg, 0.118 mmol), and dry EtOAc (4.7 mL) gave the lactone crude product, which was subjected to the reduction using DIBAL–H (1 M in hexanes) (0.7 mL, 0.7 mmol) at −78 °C for 1.5 h to provide the crude mixture of **20** and its diastereomer, which was used in the next step without further purification. A flame-dried round bottom flask equipped with a magnetic stirring bar, an argon inlet, and a rubber septum was charged with a mixture of **20** and its diastereomer (dr = 73:27) and *p*-TsOH·H_2O (25 mg, 0.13 mmol) in dry MeOH (4.4 mL) followed by the addition of a solution of $CH(OMe)_3$ (0.44 mL). After stirring at room temperature overnight (16 h), the reaction mixture was quenched with H_2O (5 mL) and extracted with EtOAc (3 × 10 mL). The combined organic layer was washed with brine and dried over anhydrous Na_2SO_4. Purification by column chromatography (3:2 v/v EtOAc:hexanes) gave a mixture of **21** and its C-2 epimer (34.6 mg, 68% yield, dr = 71:29, ^1H NMR analysis) as a light brown gum. R_f 0.66 (3:2 v/v EtOAc:hexanes). $[\alpha]_D^{23}$ −5.7 (c 0.4, acetone). ^1H NMR (400 MHz, acetone-d_6, integrated equally for both diastereomers, **21** marked*): δ 7.23* (s, 1H, O*H*), 7.22 (s, 1H, O*H*), 6.83* (s, 1H, Ar*H*), 6.81 (s, 1H, Ar*H*), 6.46* (d, *J* = 1.4 Hz, 1H, Ar*H*), 6.45 (d, *J* = 1.6 Hz, 1H, Ar*H*), 6.36* (d, *J* = 1.4 Hz, 1H, Ar*H*), 6.34 (d, *J* = 1.6 Hz, 1H, Ar*H*), 6.01* (d, *J* = 0.9 Hz, 2H, CH_2), 4.98* (d, *J* = 4.7 Hz, 1H, C*H*), 4.91 (d, *J* = 5.9 Hz, 1H, C*H*), 4.71 (d, *J* = 5.4 Hz, 1H, C*H*), 4.68* (d, *J* = 5.3 Hz, 1H, C*H*), 3.82* (s, 3H, OCH_3), 3.78* (s, 3H, OCH_3), 3.75* (s, 3H, OCH_3), 3.44 (s, 3H, OCH_3), 3.31* (s, 3H, OCH_3), 2.27−2.19 (m, 1H, C*H*), 2.11−2.08* (m, 1H, C*H*), 1.94−1.84* (m, 1H, C*H*), 0.94* (d, *J* = 7.2 Hz, 3H, CH_3), 0.91 (d, *J* = 7.2 Hz, 3H, CH_3), 0.62 (d, *J* = 7.5 Hz, 3H, CH_3), 0.53* (d, *J* = 7.4 Hz, 3H, CH_3) ppm. Some proton peaks of minor isomer overlap with **21**. $^{13}C\{^1H\}$ NMR (100 MHz, acetone-d_6): δ 149.0* (C), 148.9 (C), 148.6* (C), 148.6 (C), 148.4* (C), 148.4 (C), 142.1* (C), 141.9 (C), 136.9* (C), 136.7 (C), 136.1* (C), 136.0 (C), 135.2 (C), 133.7* (C), 128.0* (C), 127.9 (C), 127.7 (C), 127.6* (C), 111.1* (CH), 109.2* (CH), 109.1 (CH), 107.8* (CH), 107.7 (CH), 107.3 (CH), 103.0 (CH), 102.6* (CH), 102.1* (CH_2), 102.0 (CH_2), 83.6 (CH), 81.4* (CH), 60.2* (OCH_3), 56.8* (2 × OCH_3), 56.7 (2 × OCH_3), 56.0* (OCH_3), 55.9 (OCH_3), 44.9* (CH), 42.7 (CH), 41.5* (CH), 40.1 (CH), 12.5* (CH_3), 12.0 (CH_3), 10.4* (CH_3), 10.0 (CH_3) ppm. Some carbon peaks of minor isomer overlap with **21**. IR (ATR): ν_{max} 3448, 2935, 1610, 1474, 1219, 1114 cm^{-1}.

MS: *m/z* (%) relative intensity 455 [(M + Na)$^+$, 100], 381 (28), 353 (37). HRMS (ESI-TOF) *m/z*: [M + Na]$^+$ Calcd for $C_{23}H_{28}NaO_8$ 455.1682; Found 455.1668.

Synthesis of 22

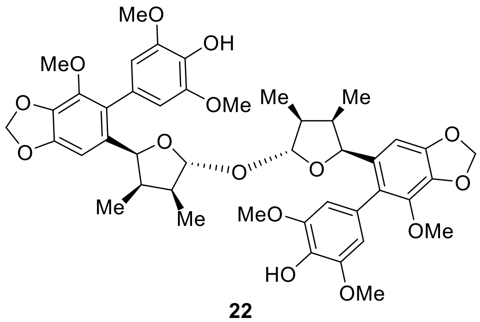

22

Following the synthetic sequences for the synthesis of **21**, treatment of **20** and its C-2 epimer (dr = 73:27, 16 mg, 0.038 mmol) with *p*-TsOH·H_2O (7.2 mg, 0.038 mmol) and $CuSO_4·5H_2O$ (28.7 mg, 0.11 mmol) in dry CH_2Cl_2 (1 mL) at 0 °C for 12 h gave a mixture of **22** and its diastereomers in 30% combined yield (4.7 mg) after column chromatography (2:3 v/v EtOAc:hexanes). Purification by preparative thin-layer chromatography (3:7 v/v EtOAc:hexanes) gave **22** in 21% yield (3.3 mg) as a brown oil. R_f 0.30 (2:3 v/v EtOAc:hexanes, triple run). $[\alpha]_D^{25}$ +34.2 (c 0.1, acetone). ^{1}H NMR (500 MHz, acetone-d_6): δ 7.21 (s, 2H, 2 × O*H*), 6.82 (s, 2H, 2 × Ar*H*), 6.43 (d, *J* = 1.6 Hz, 2H, 2 × Ar*H*), 6.35 (d, *J* = 1.6 Hz, 2H, 2 × Ar*H*), 6.03−6.00 (m, 4H, 2 × C*HH*), 5.07 (d, *J* = 5.0 Hz, 2H, 2 × C*H*), 4.98 (d, *J* = 5.0 Hz, 2H, 2 × C*H*), 3.80 (s, 6H, 2 × OC*H*$_3$), 3.78 (s, 6H, 2 × OC*H*$_3$), 3.74 (s, 6H, 2 × OC*H*$_3$), 2.09–2.07 (m, 2H, 2 × C*H*), 1.98–1.93 (m, 2H, 2 × C*H*), 0.88 (d, *J* = 7.2 Hz, 6H, 2 × C*H*$_3$), 0.53 (d, *J* = 7.4 Hz, 6H, 2 × C*H*$_3$) ppm. ^{13}C{^{1}H} NMR (125 MHz, acetone-d_6): δ 149.0 (2 × C), 148.6 (2 × C), 148.4 (2 × C), 142.1 (2 × C), 137.0 (2 × C), 136.1 (2 × C), 133.6 (2 × C), 128.1 (2 × C), 127.6 (2 × C), 109.2 (2 × CH), 107.7 (2 × CH), 106.1 (2 × CH), 102.6 (2 × CH), 102.1 (2 × CH_2), 81.4 (2 × CH), 60.2 (2 × OCH_3), 56.8 (4 × OCH_3), 44.9 (2 × CH), 41.1 (2 × CH), 12.4 (2 × CH_3), 10.6 (2 × CH_3) ppm. IR (ATR): ν_{max} 3428, 2924, 1462, 1083 cm^{-1}. MS: *m/z* (%) relative intensity 841 [(M+ Na)$^+$, 16], 455 (23), 381 (71), 353 (100). HRMS (ESI-TOF) *m/z*: [M + Na]$^+$ Calcd for $C_{44}H_{50}NaO_{15}$ 841.3047; Found 841.3050.

References

[1] Schmidt, B.; Riemer, M. Suzuki−Miyaura Coupling of Halophenols and Phenol Boronic Acids: Systematic Investigation of Positional Isomer Effects and Conclusions for the Synthesis of Phytoalexins from Pyrinae. *J. Org. Chem.* **2014**, *79*, 4104−4118.

[2] Bovonsombat, P.; Leykajarakul, J.; Khan, C.; Pla-on, K.; Krause, M. M.; Khanthapura, P.; Ali, R.; Doowa, N. Regioselective Iodination of Phenol and Analogues using *N*-Iodosuccinimide and *p*-Toluenesulfonic Acid. *Tetrahedron Lett.* **2009**, *50*, 2664−2667.

[3] (a) Moleele, S. S.; Michael J. P.; de Koning, C. B. Methodology for the Synthesis of 1,2-Disubstituted Arylnaphthalenes from α-Tetralones. *Tetrahedron* **2006**, *62*, 2831–2844. (b) Niphakis, M. J.; Georg, G. I. Synthesis of Tylocrebrine and Related Phenanthroindolizidines by VOF_3-Mediated Oxidative Aryl-Alkene Coupling. *Org. Lett.* **2011**, *13*, 196–199.

[4] Jana, A.; Ravichandiran, V.; Swain, S. P. Application of Organometallic Catalysts for the Synthesis of *o*-Tolyl Benzonitrile, A Key Starting Material for Sartans. *New J. Chem.* **2021**, *45*, 17753–17771.

[5] Yodwaree, S.; Soorukram, D.; Kuhakarn, C.; Tuchinda, P.; Reutrakul, V.; Pohmakotr, M. Formal Synthesis of (+)-3-*epi*-Eupomatilone-6 and the 3,5-bis-Epimer. *Org. Biomol. Chem.* **2014**, *12*, 6885–6894.

^{1}H NMR spectrum of **10** (400 MHz, $CDCl_3$)

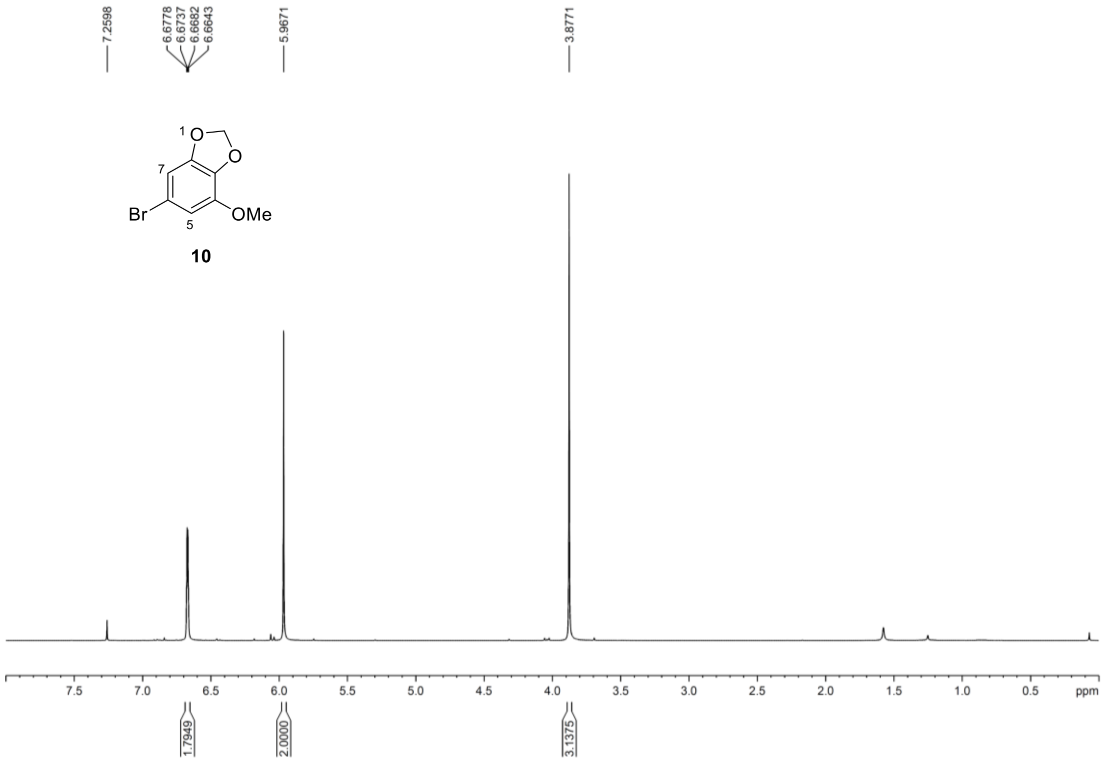

^{13}C{^{1}H} NMR spectrum of **10** (100 MHz, $CDCl_3$)

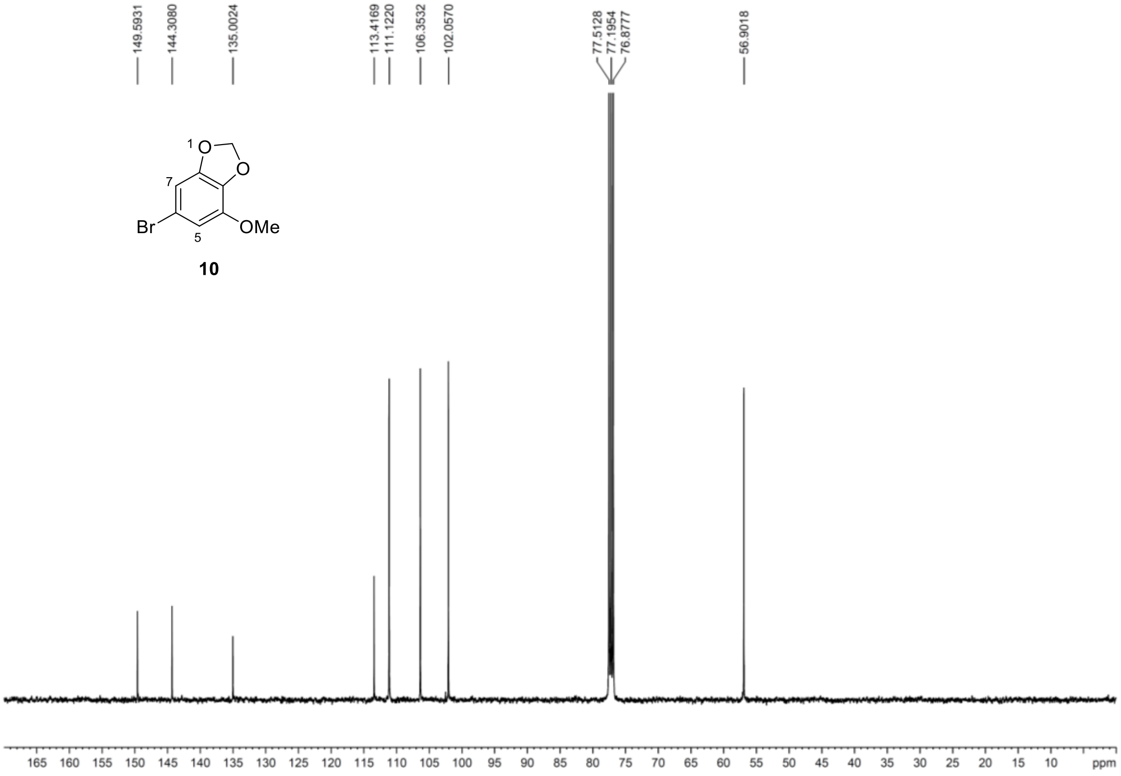

^{1}H NMR spectrum of **11a** (400 MHz, $CDCl_3$)

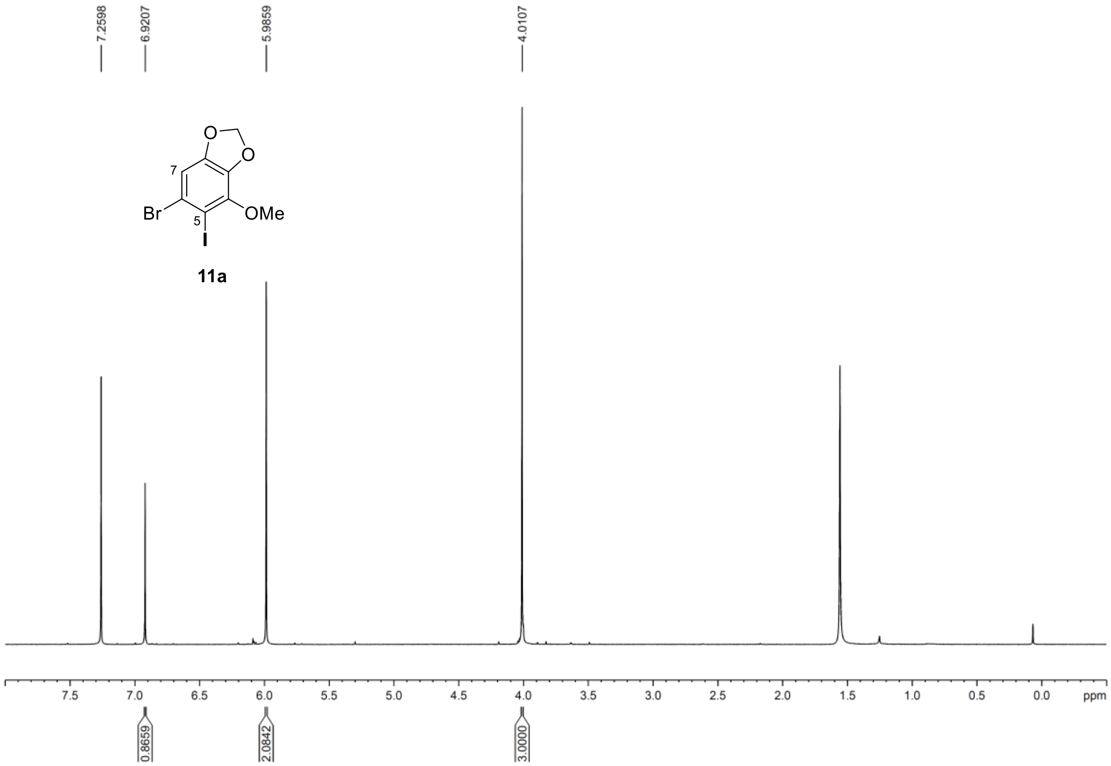

$^{13}C\{^{1}H\}$ NMR spectrum of **11a** (100 MHz, $CDCl_3$)

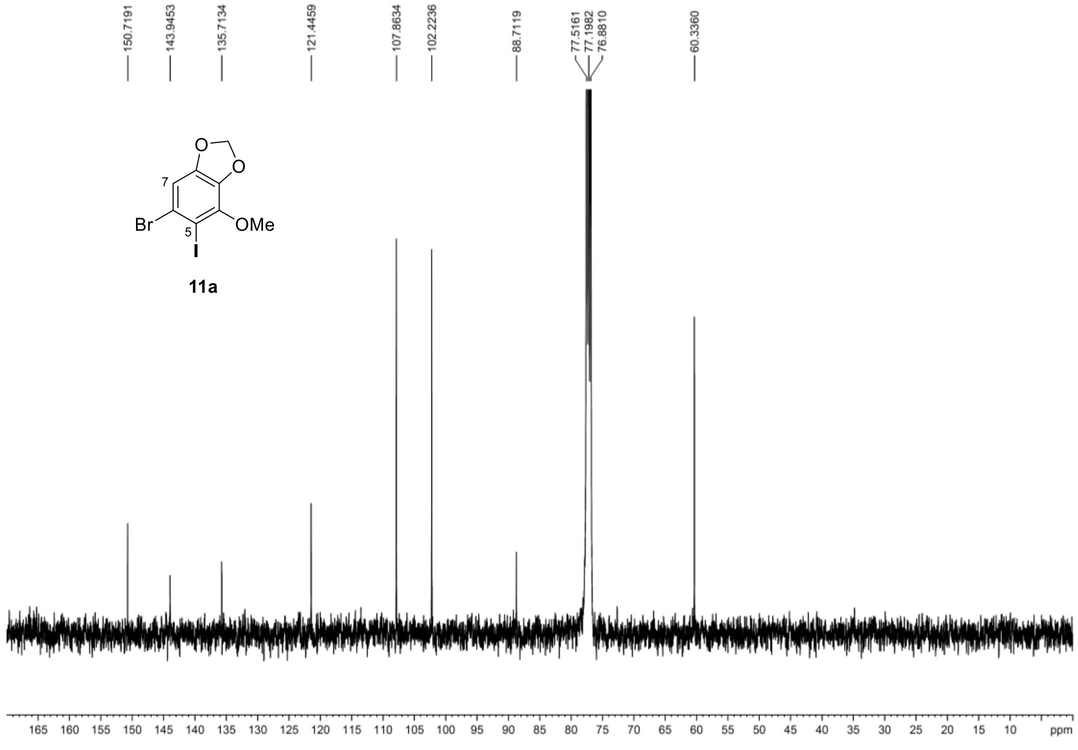

DEPT-135 NMR spectrum of **11a** (100 MHz, $CDCl_3$)

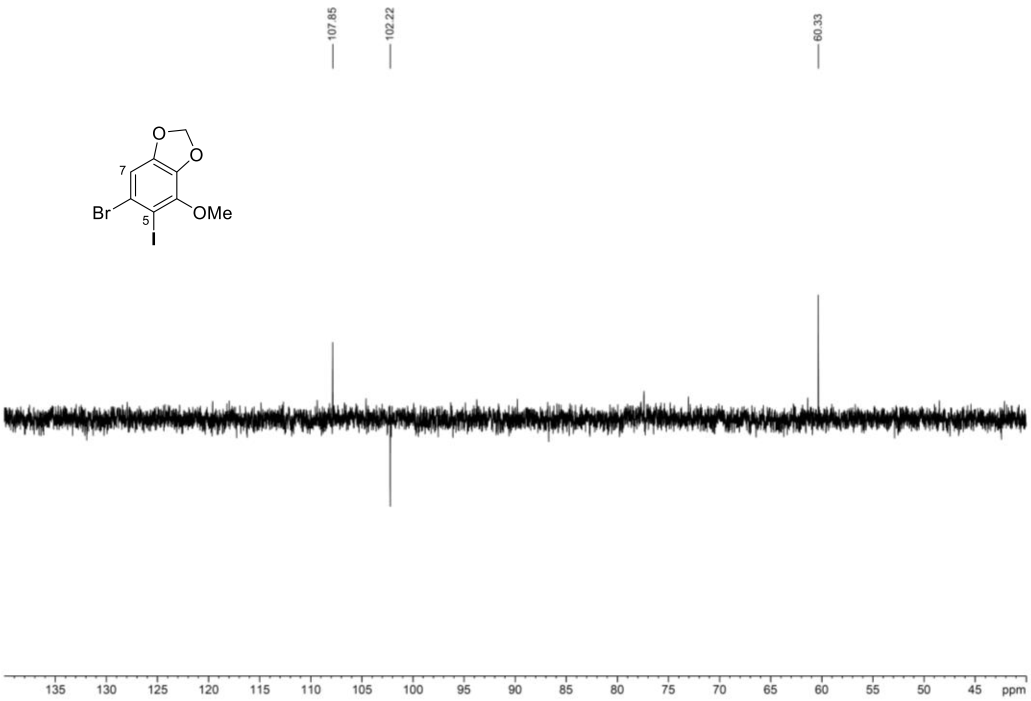

HSQC spectrum of **11a** (400 MHz for ^{1}H NMR and 100 MHz for ^{13}C{^{1}H} NMR, $CDCl_3$)

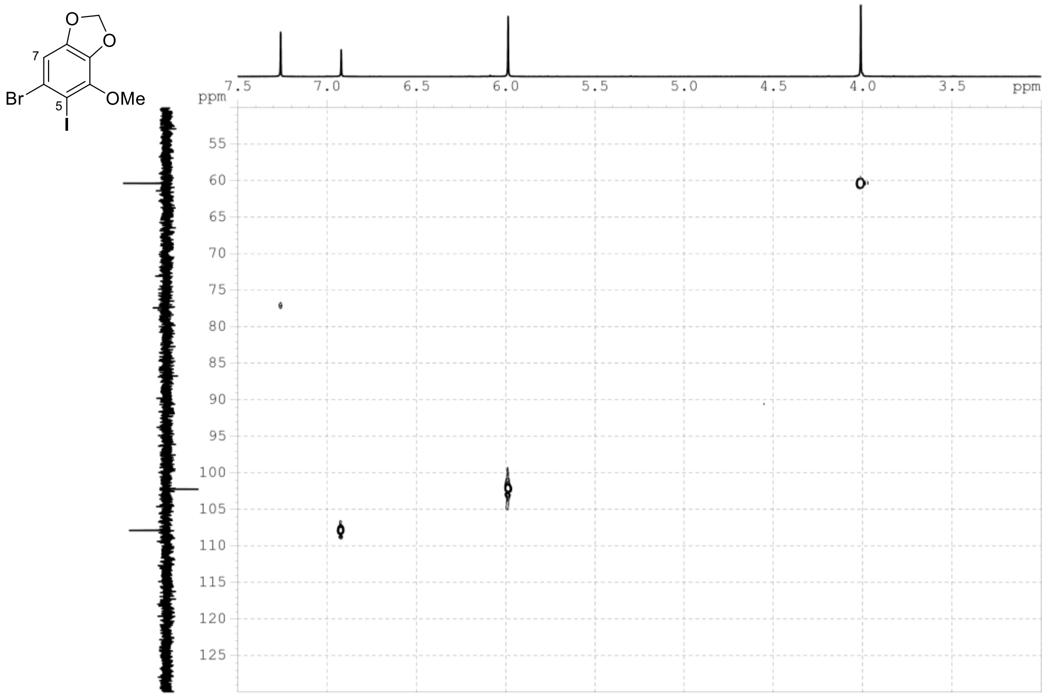

HMBC spectrum of **11a** (400 MHz for ^{1}H NMR and 100 MHz for $^{13}C\{^{1}H\}$ NMR, $CDCl_3$)

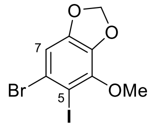

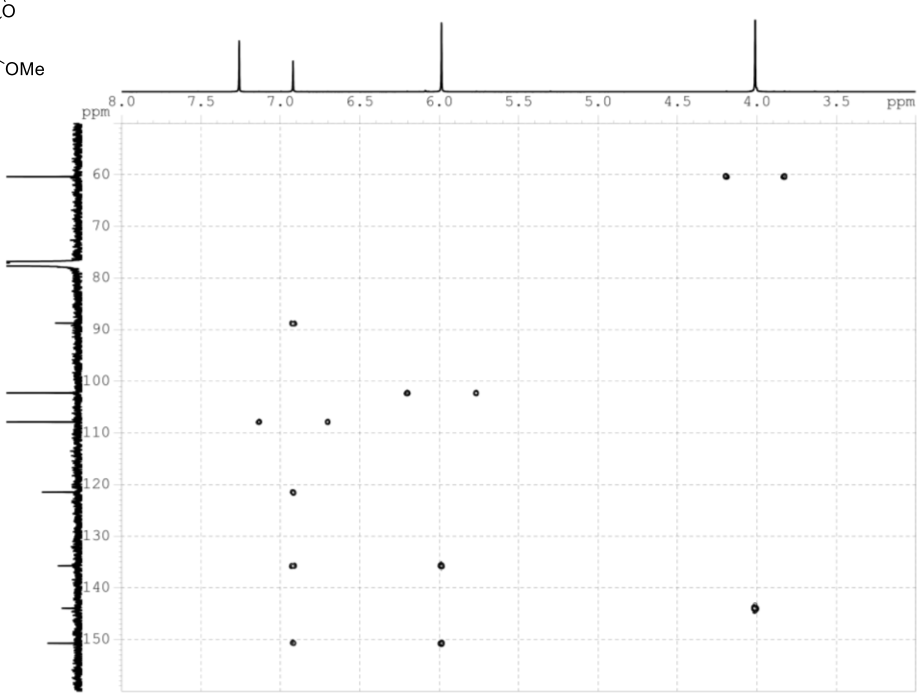

Key NOESY correlation of **11a** (400 MHz, $CDCl_3$)

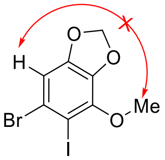

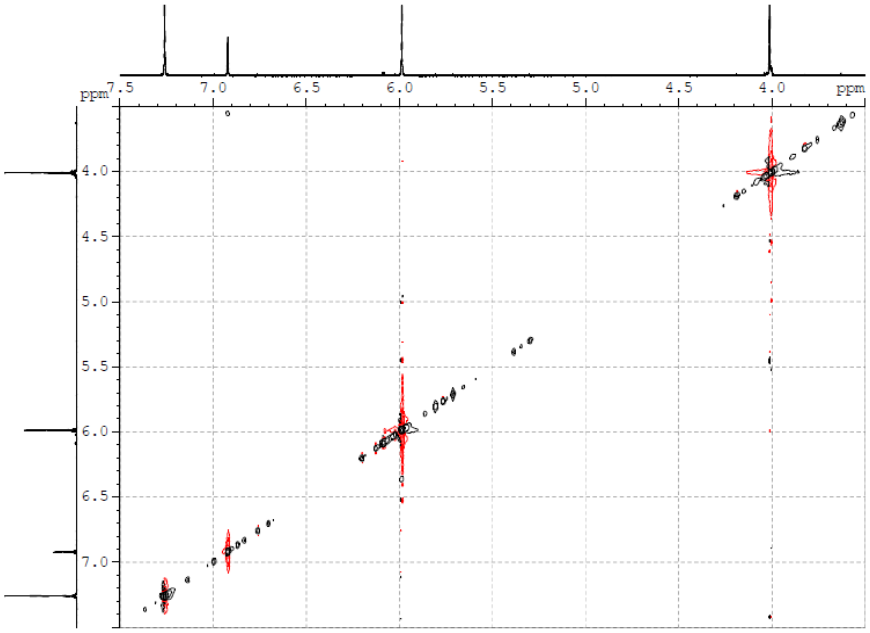

^{1}H NMR spectrum of **11b** (400 MHz, $CDCl_3$)

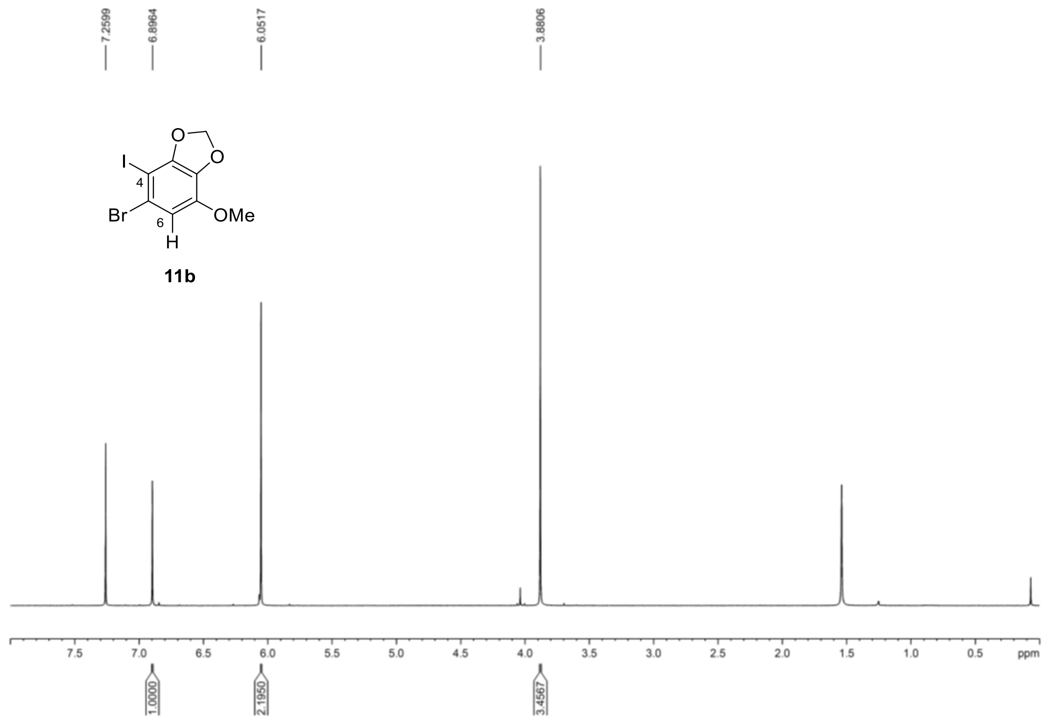

^{13}C{^{1}H} NMR spectrum of **11b** (100 MHz, $CDCl_3$)

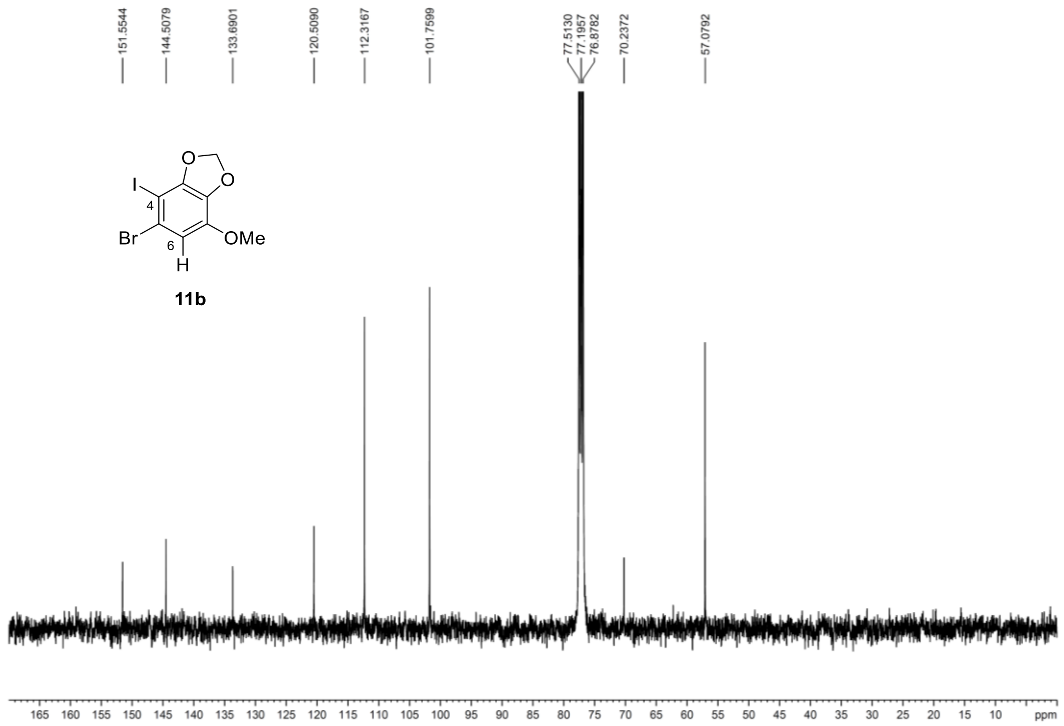

DEPT-135 NMR spectrum of **11b** (100 MHz, $CDCl_3$)

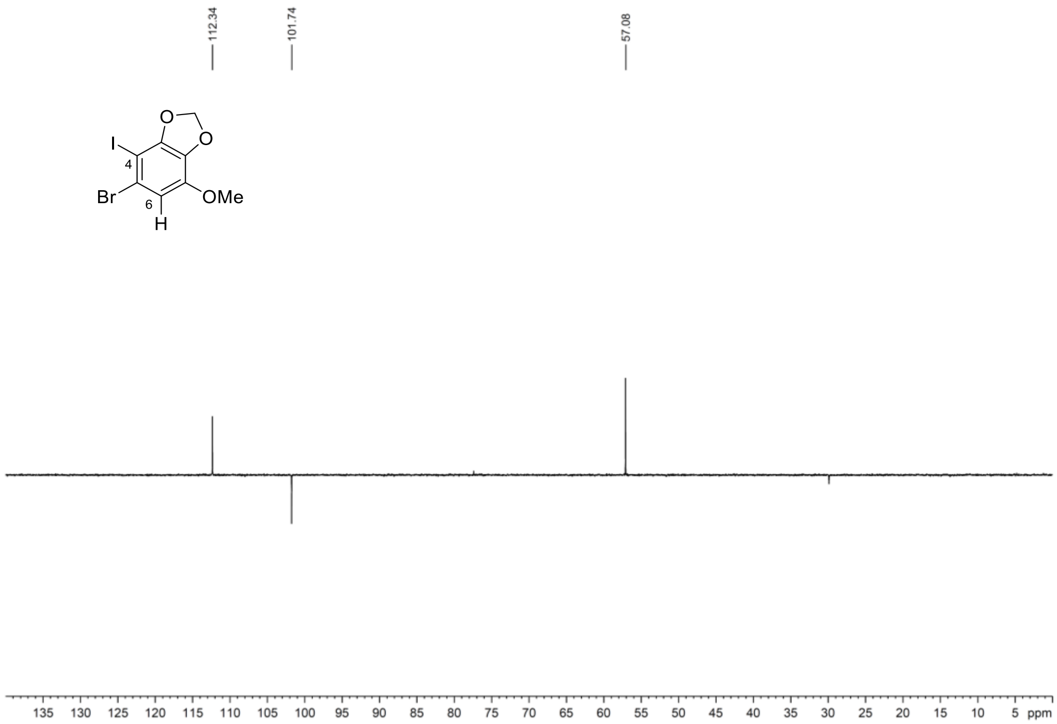

HSQC spectrum of **11b** (400 MHz for ^{1}H NMR and 100 MHz for $^{13}C\{^{1}H\}$ NMR, $CDCl_3$)

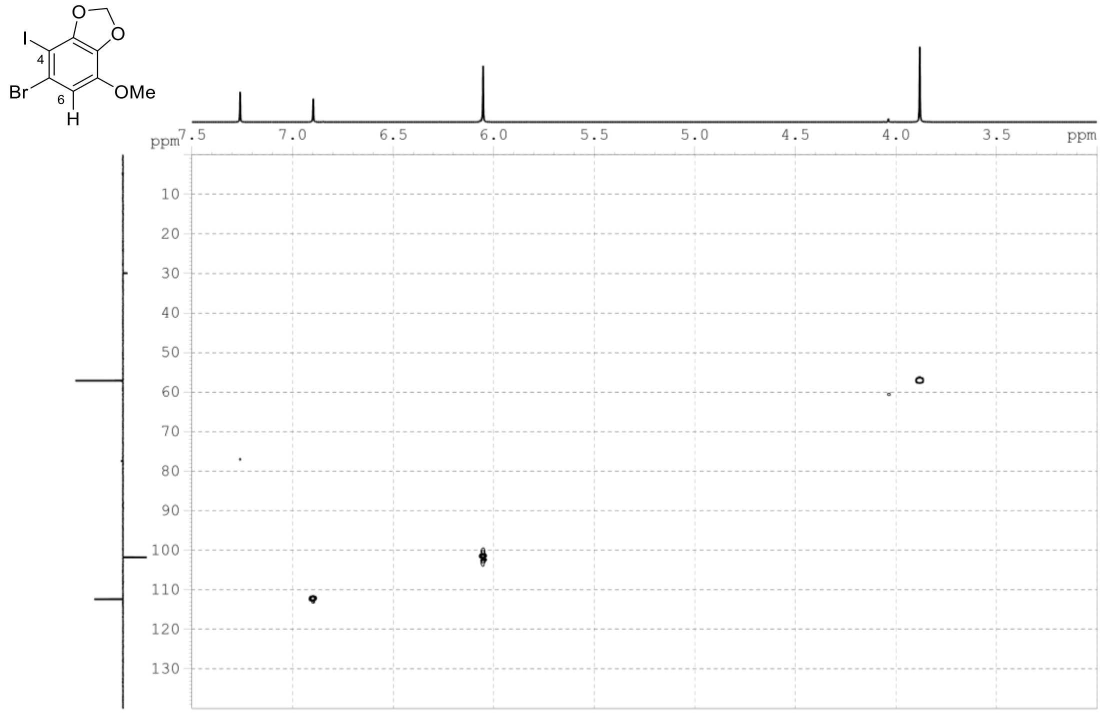

HMBC spectrum of **11b** (400 MHz for ^{1}H NMR and 100 MHz for $^{13}C\{^{1}H\}$ NMR, $CDCl_3$)

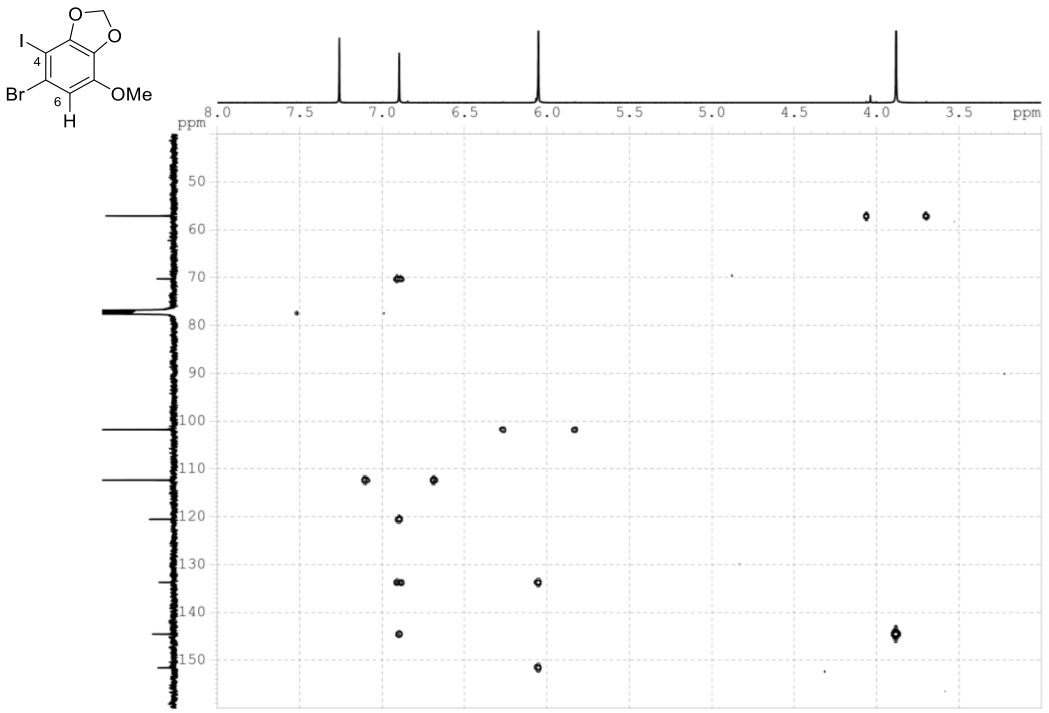

Key NOESY correlation of **11b** (400 MHz, $CDCl_3$)

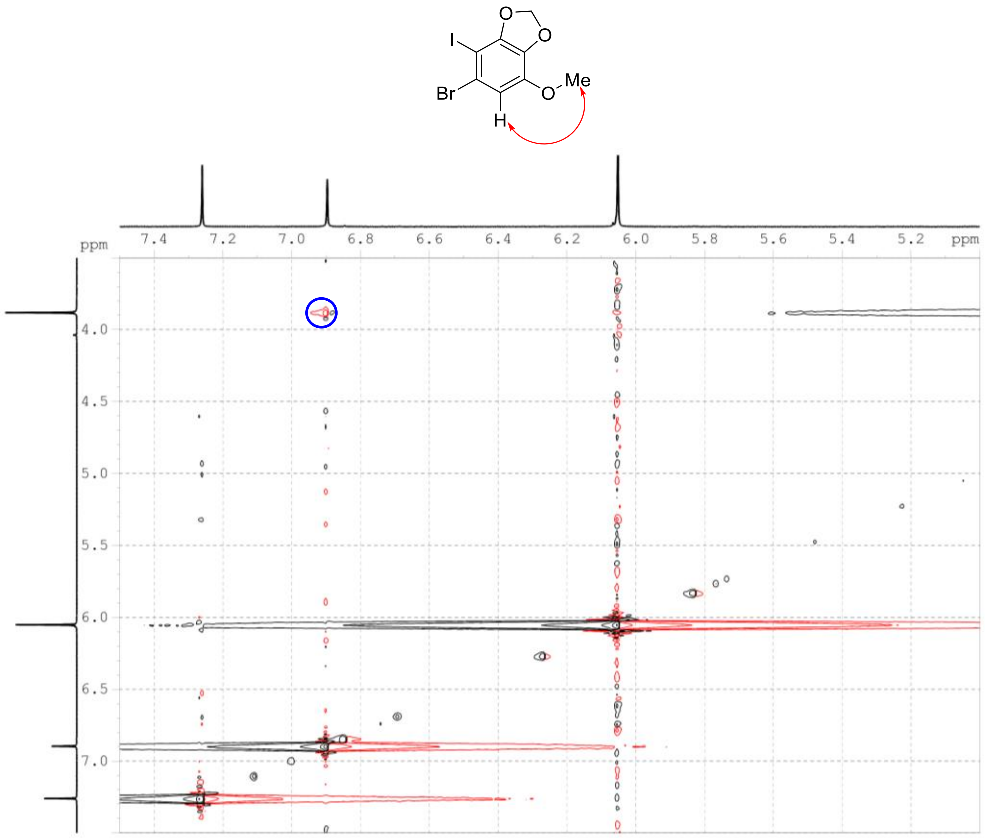

^{1}H NMR spectrum of **11c** (400 MHz, $CDCl_3$)

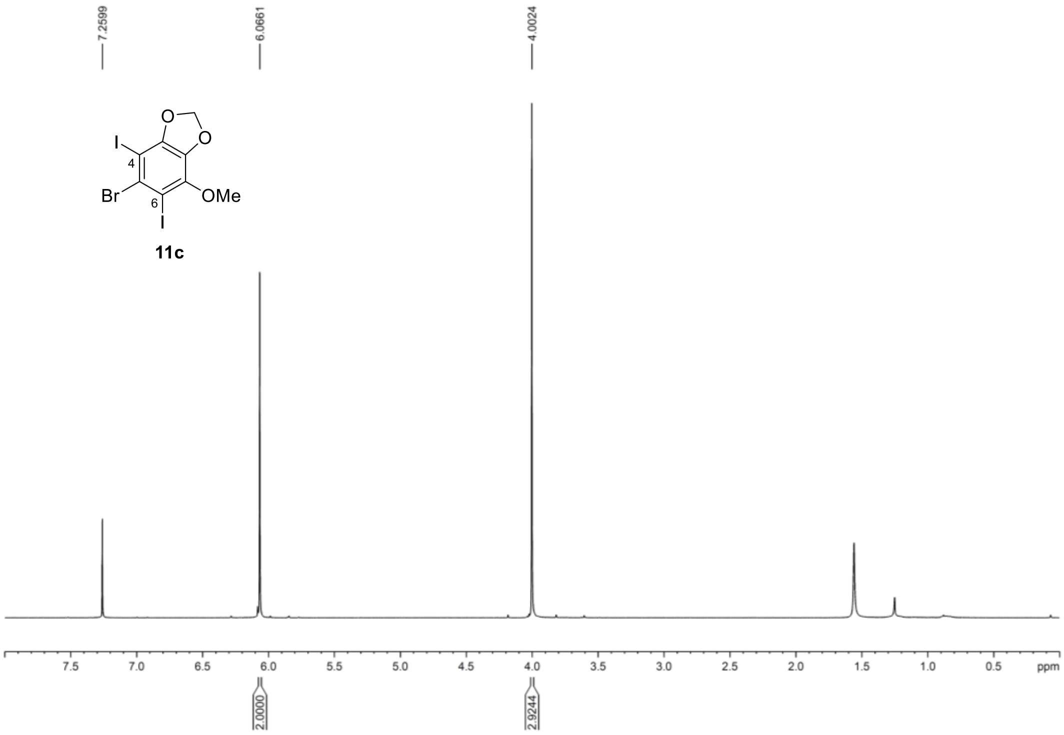

^{13}C{^{1}H} NMR spectrum of **11c** (100 MHz, $CDCl_3$)

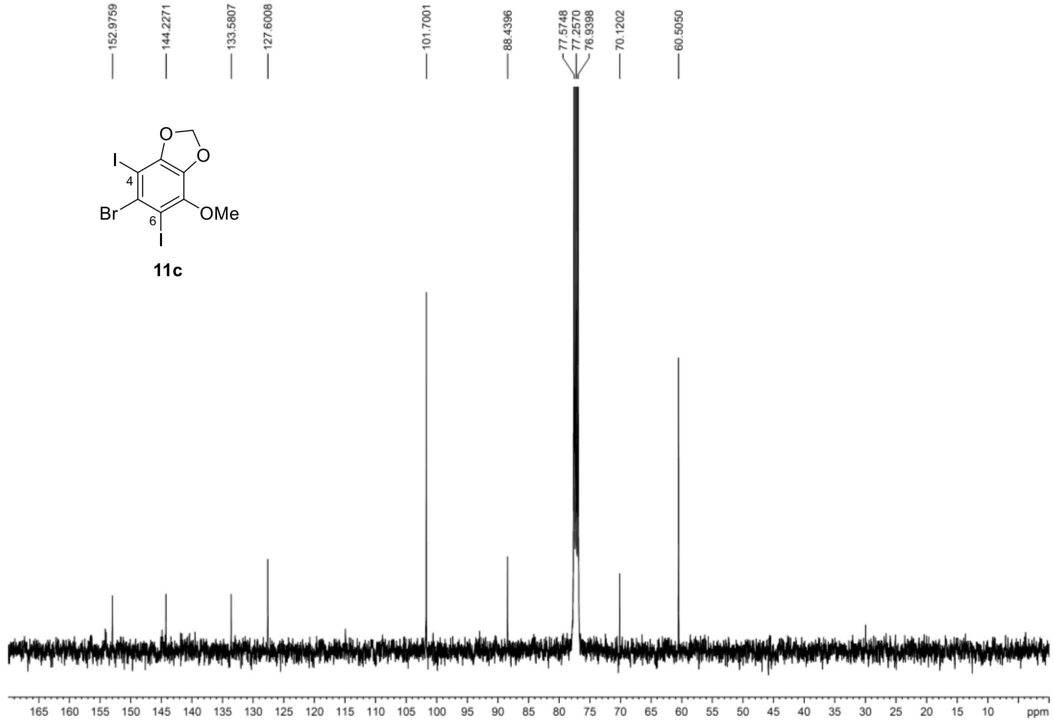

DEPT-135 NMR spectrum of **11c** (100 MHz, $CDCl_3$)

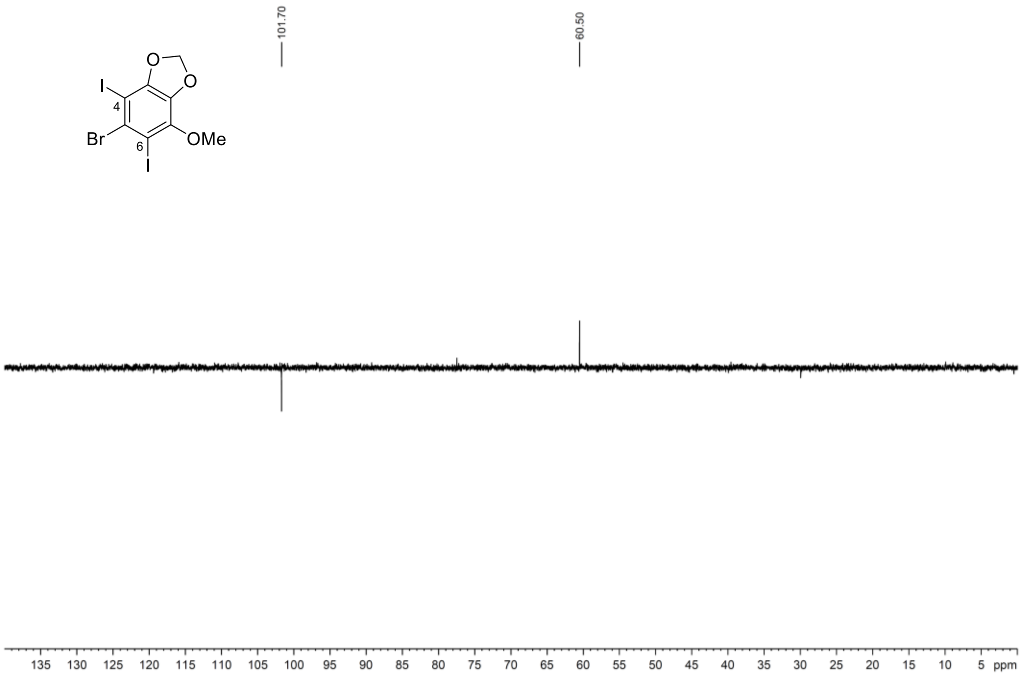

HSQC spectrum of **11c** (400 MHz for ^1H NMR and 100 MHz for $^{13}C\{^1H\}$ NMR, $CDCl_3$)

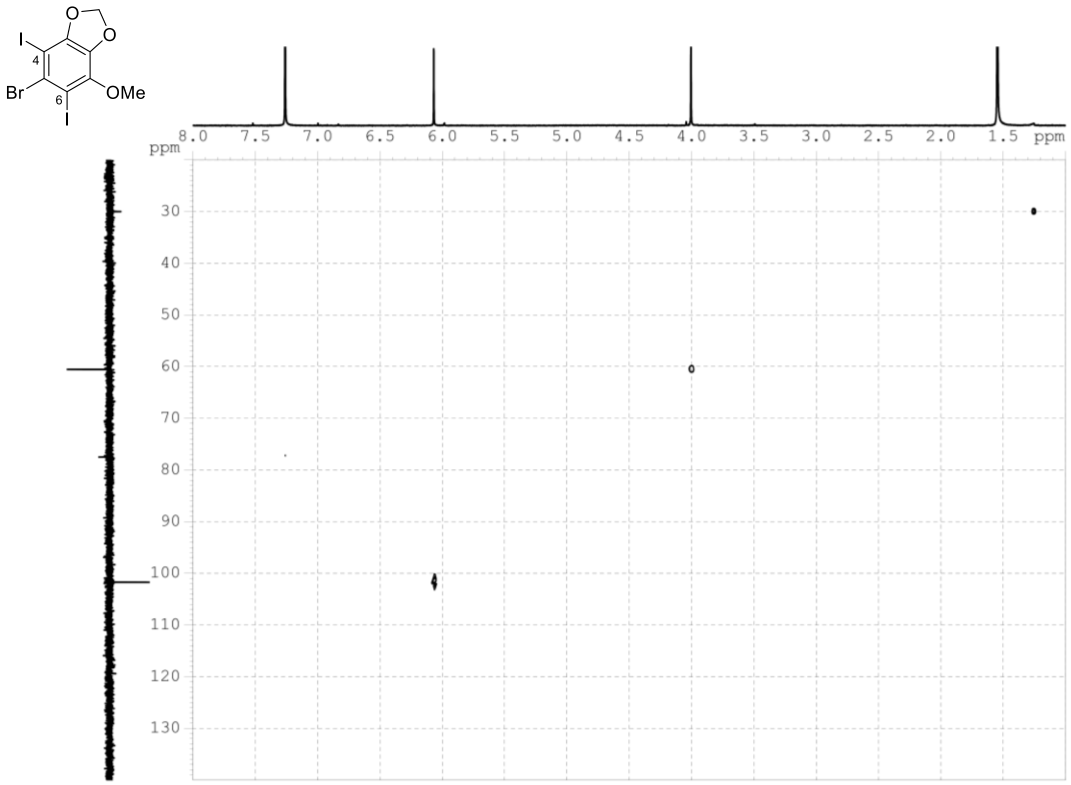

HMBC spectrum of **11c** (400 MHz for ^{1}H NMR and 100 MHz for $^{13}C\{^{1}H\}$ NMR, $CDCl_3$)

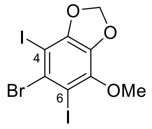

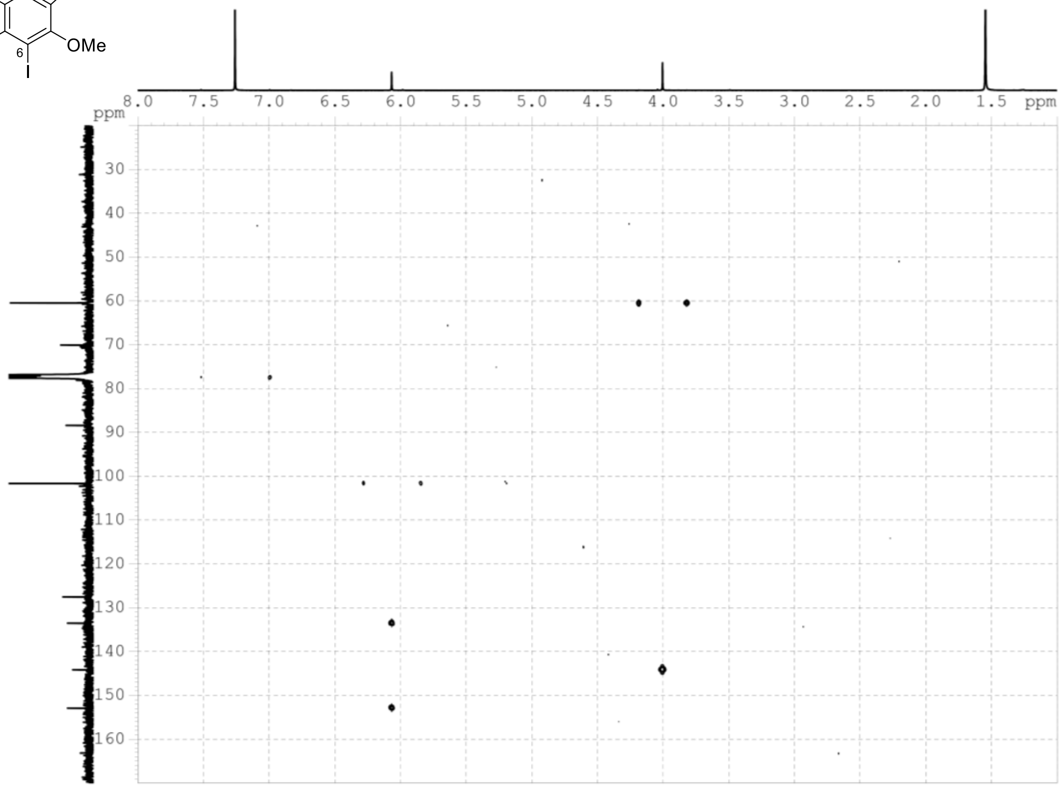

^{1}H NMR spectrum of **8a** (400 MHz, $CDCl_3$)

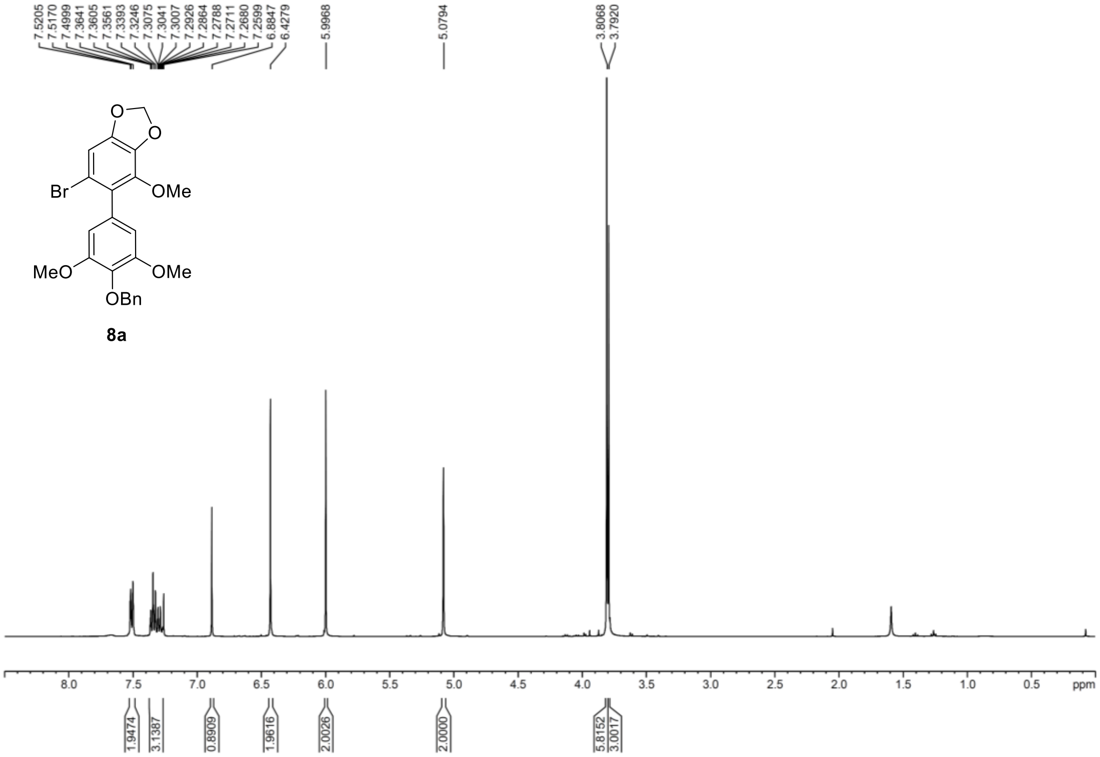

$^{13}C\{^{1}H\}$ NMR spectrum of **8a** (100 MHz, $CDCl_3$)

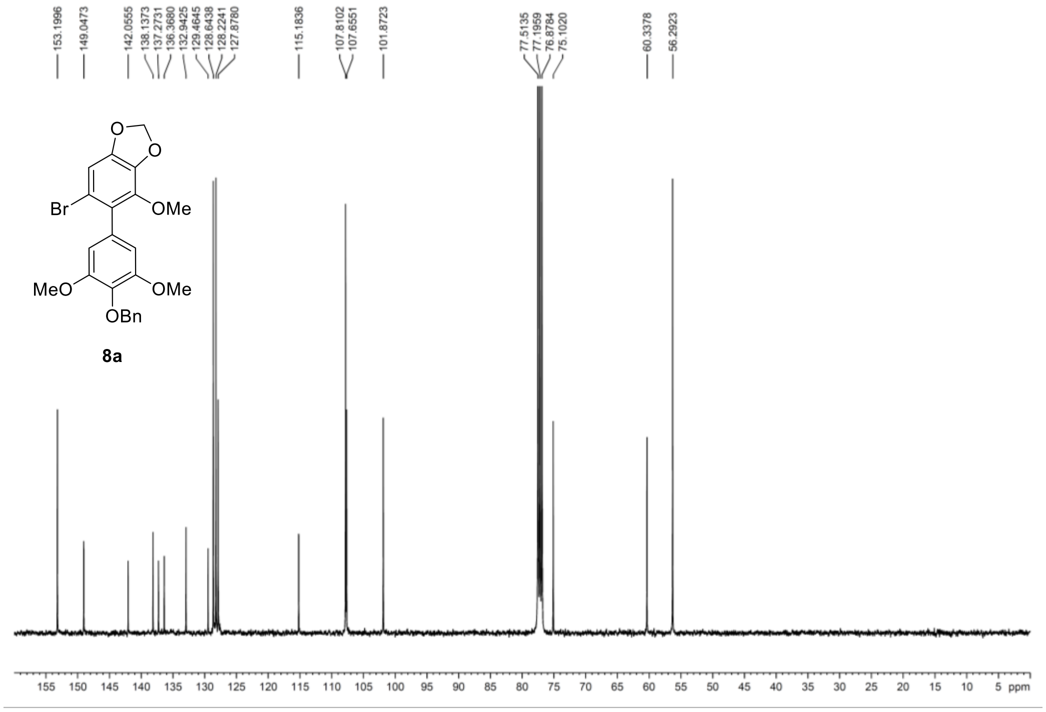

DEPT-135 NMR spectrum of **8a** (100 MHz, $CDCl_3$)

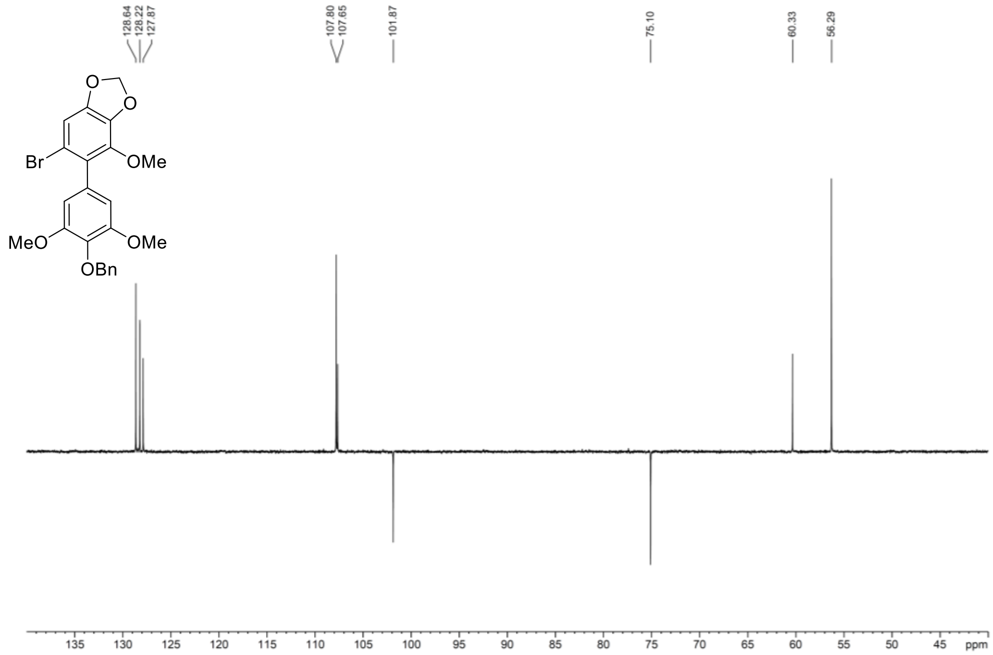

HSQC spectrum of **8a** (400 MHz for ^1H NMR and 100 MHz for $^{13}C\{^1H\}$ NMR, $CDCl_3$)

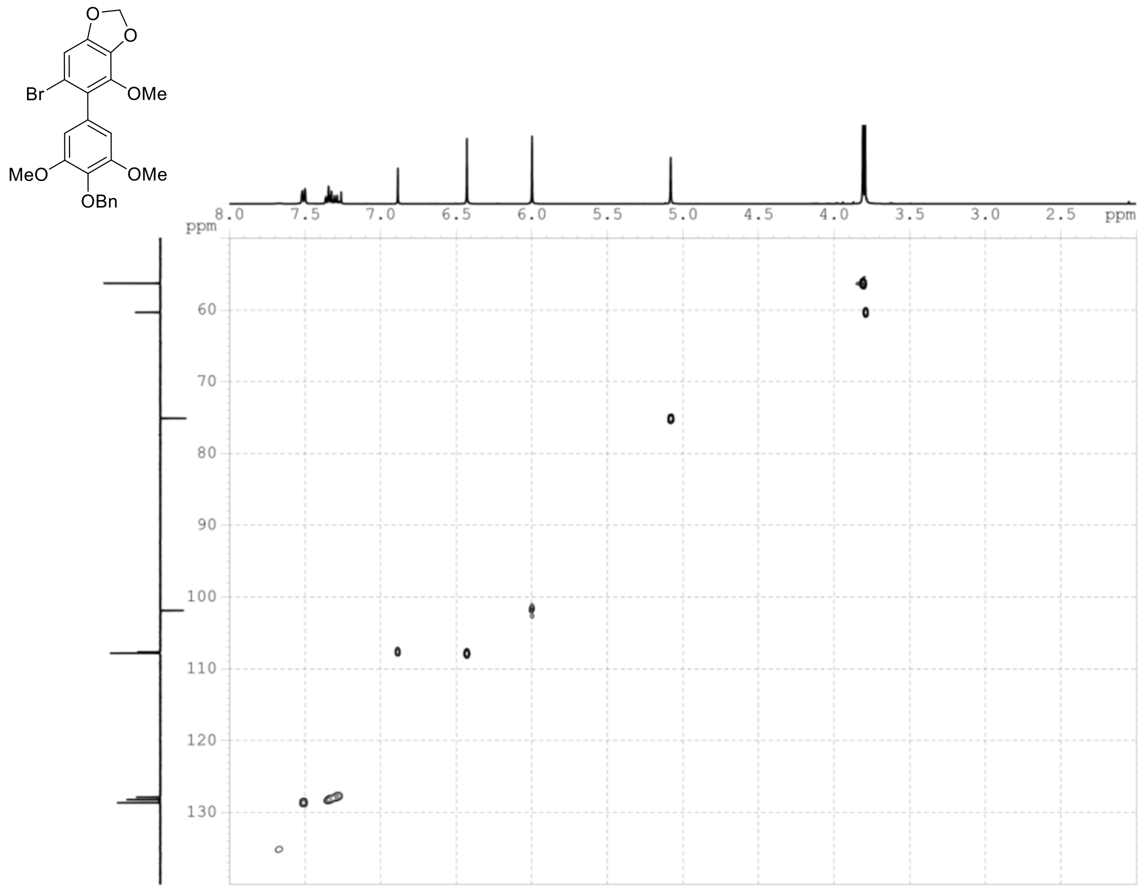

HMBC spectrum of **8a** (400 MHz for ^{1}H NMR and 100 MHz for ^{13}C{^{1}H} NMR, $CDCl_3$)

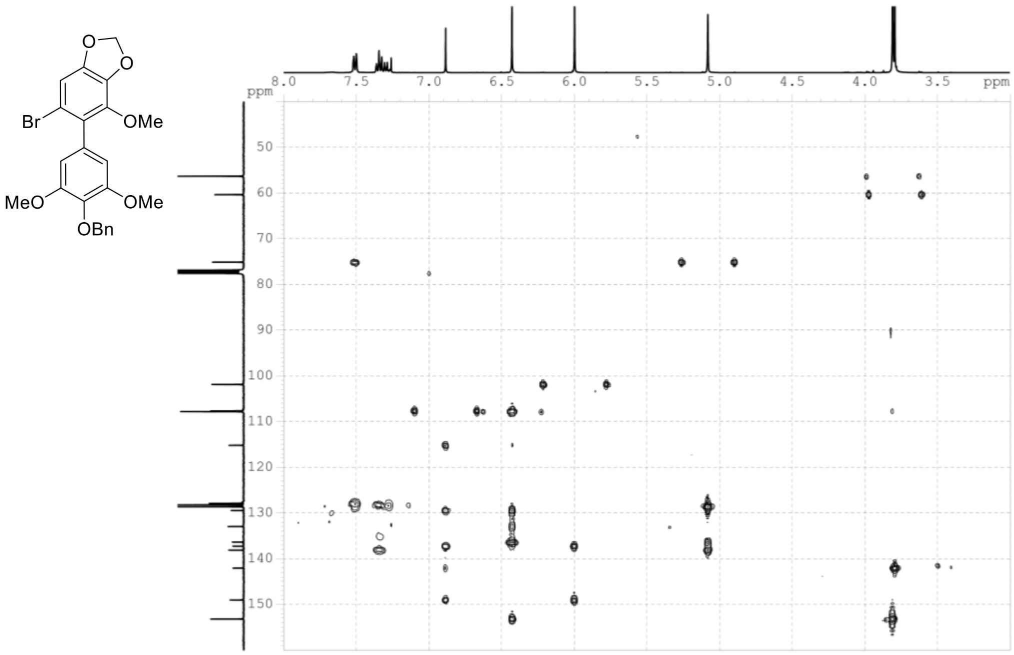

Key NOESY correlation of **8a** (400 MHz, $CDCl_3$)

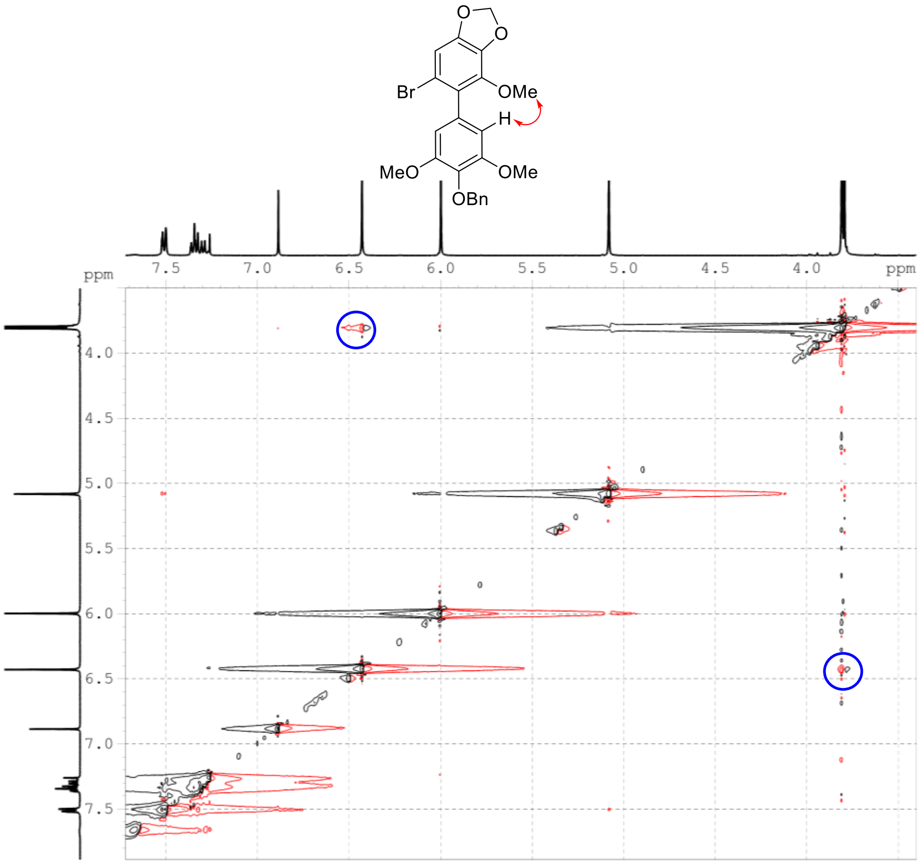

^{1}H NMR spectrum of **8b** (400 MHz, $CDCl_3$)

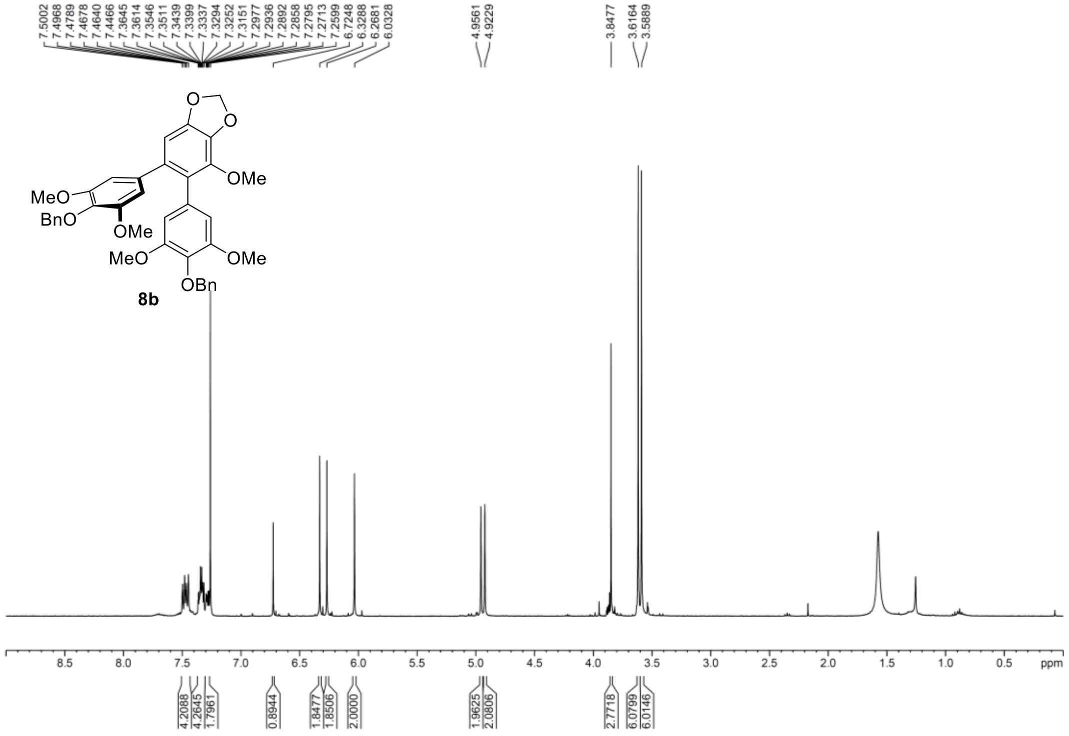

^{13}C{^{1}H} NMR spectrum of **8b** (100 MHz, $CDCl_3$)

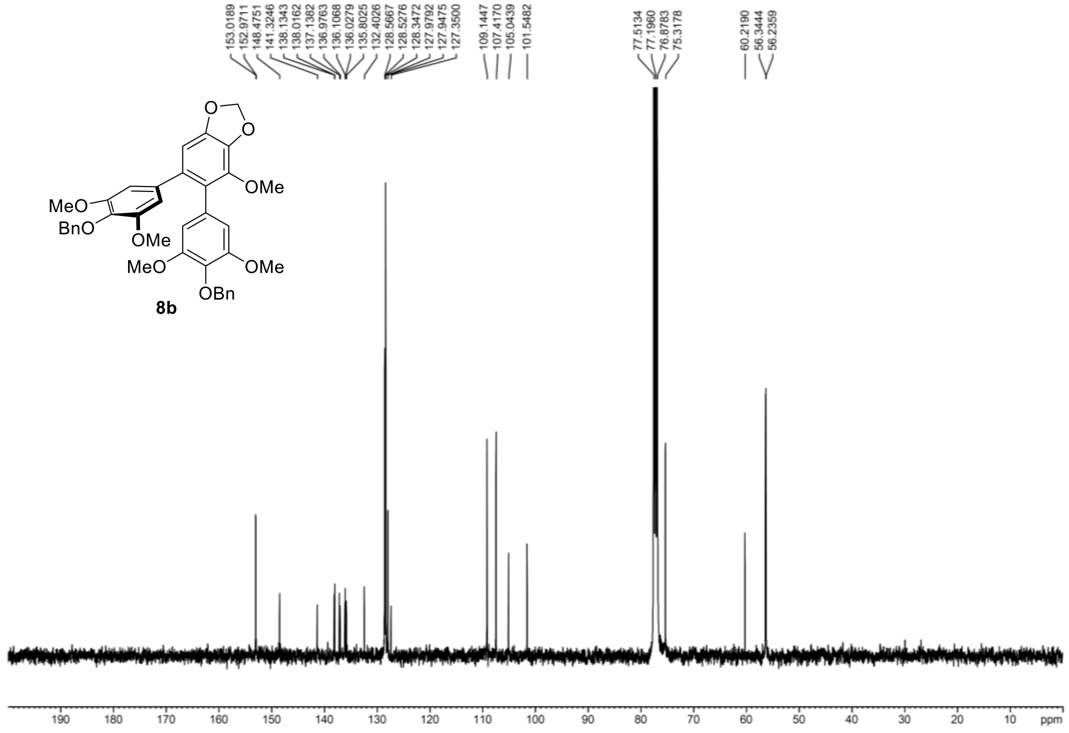

DEPT-135 NMR spectrum of **8b** (100 MHz, $CDCl_3$)

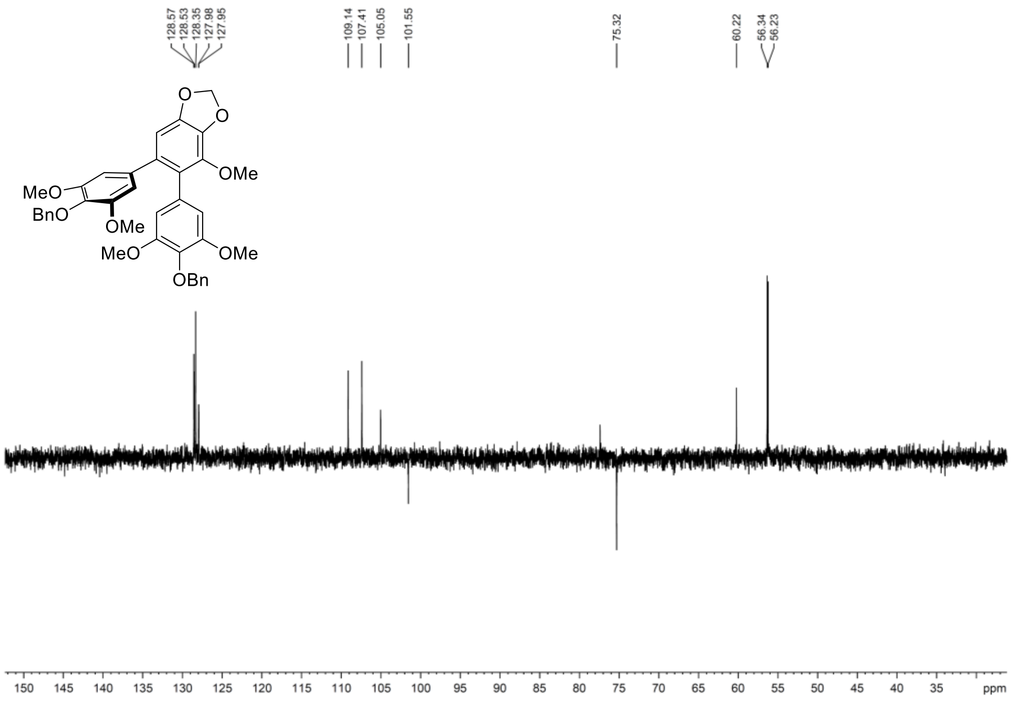

HSQC spectrum of **8b** (400 MHz for ^1H NMR and 100 MHz for $^{13}C\{^1H\}$ NMR, $CDCl_3$)

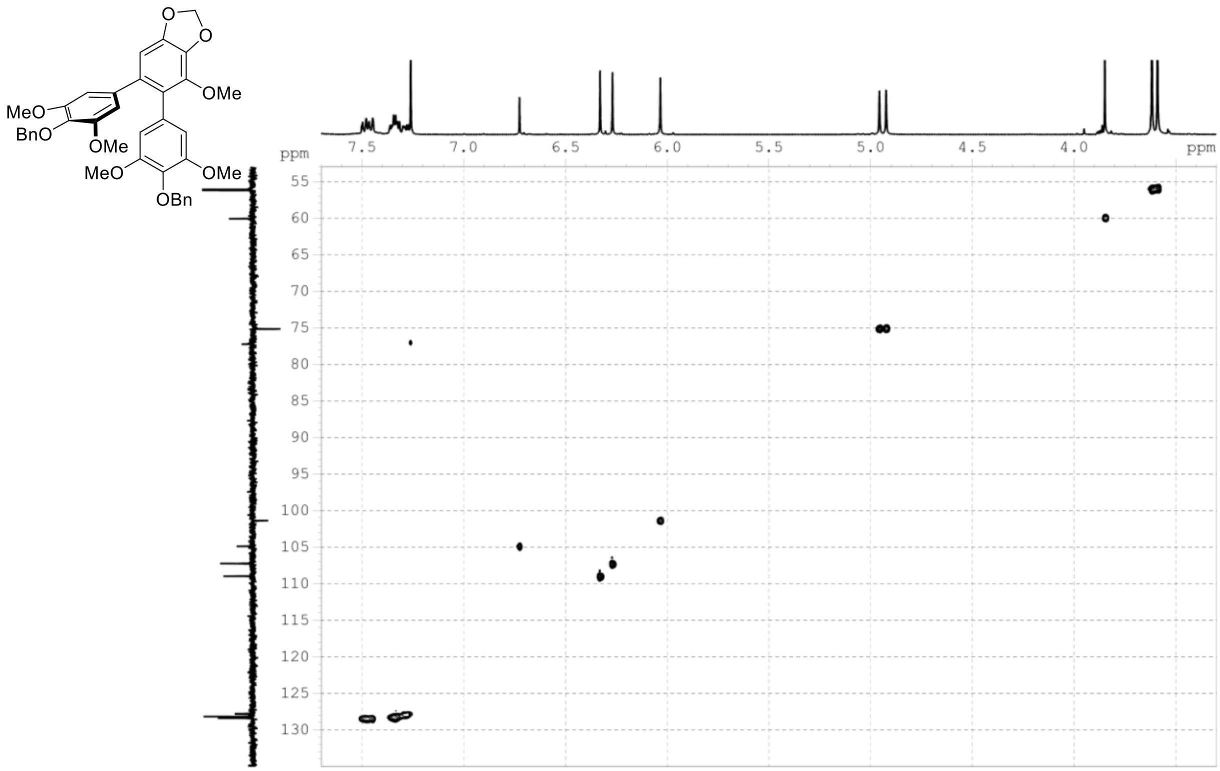

HMBC spectrum of **8b** (400 MHz for ^{1}H NMR and 100 MHz for ^{13}C{^{1}H} NMR, $CDCl_3$)

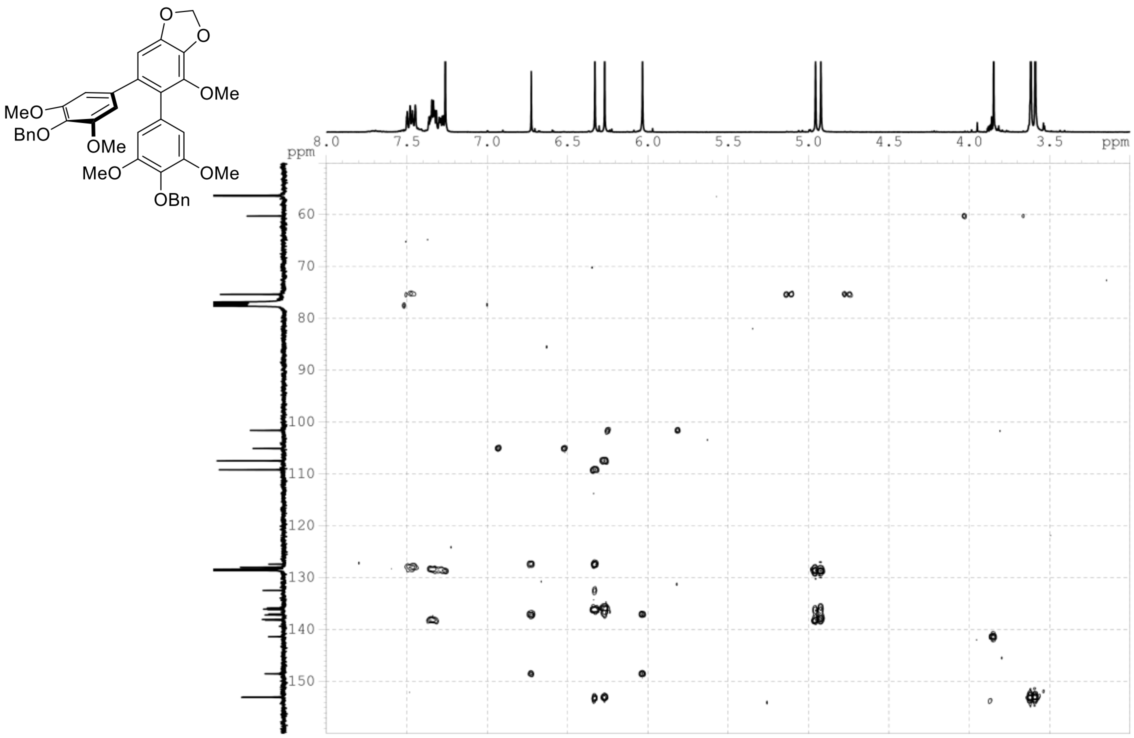

Key NOESY correlations of **8b** (400 MHz, $CDCl_3$)

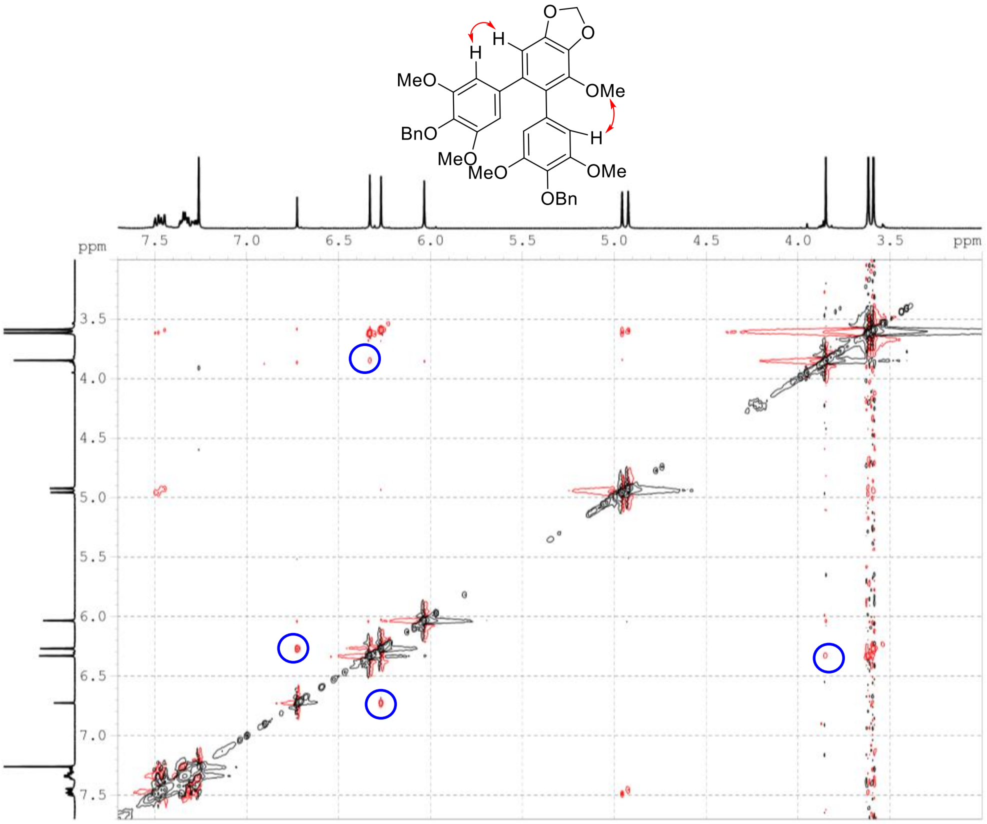

^{1}H NMR spectrum of **13** (400 MHz, $CDCl_3$)

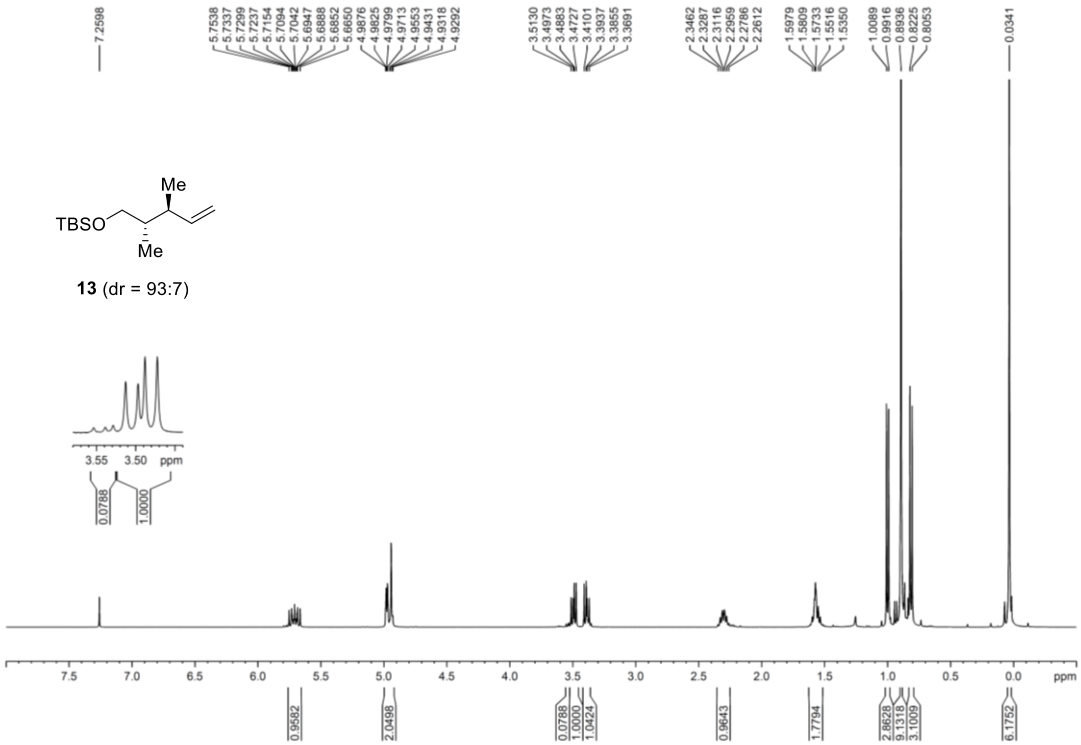

^{13}C{^{1}H} NMR spectrum of **13** (100 MHz, $CDCl_3$)

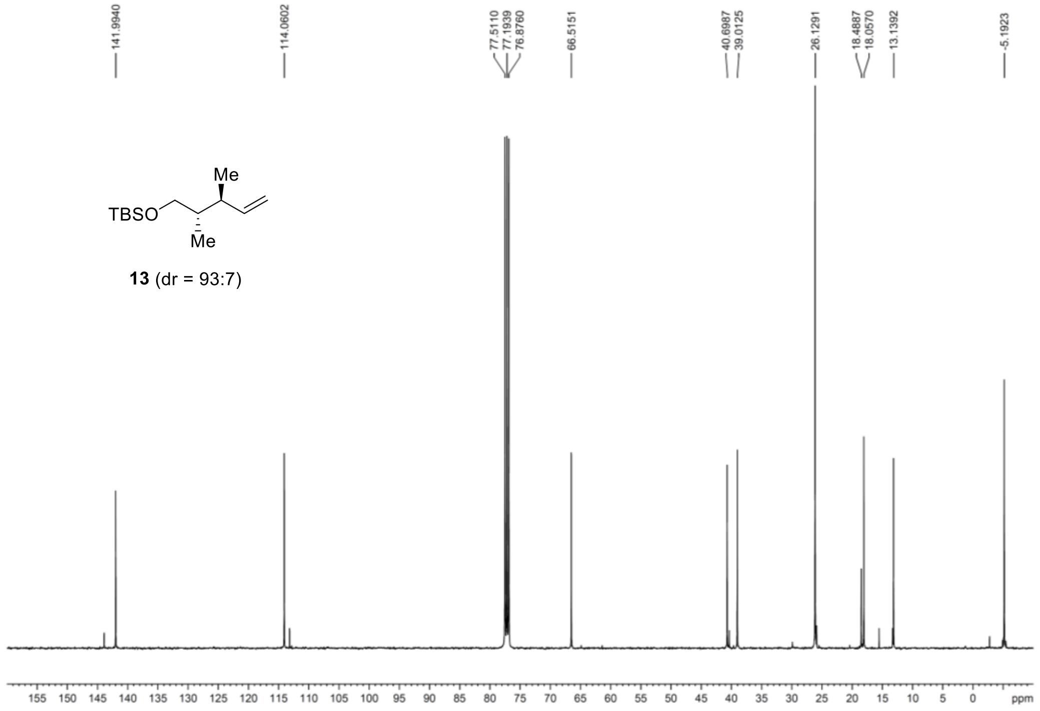

DEPT-135 NMR spectrum of **13** (100 MHz, $CDCl_3$)

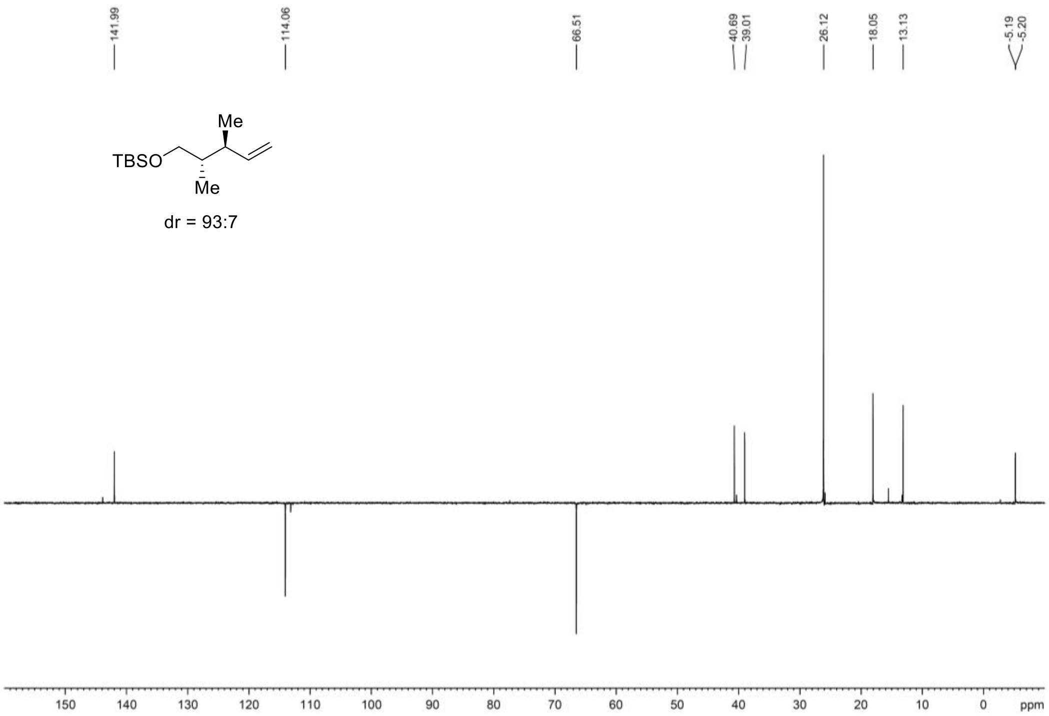

HSQC spectrum of **13** (400 MHz for ^1H NMR and 100 MHz for $^{13}C\{^1H\}$ NMR, $CDCl_3$)

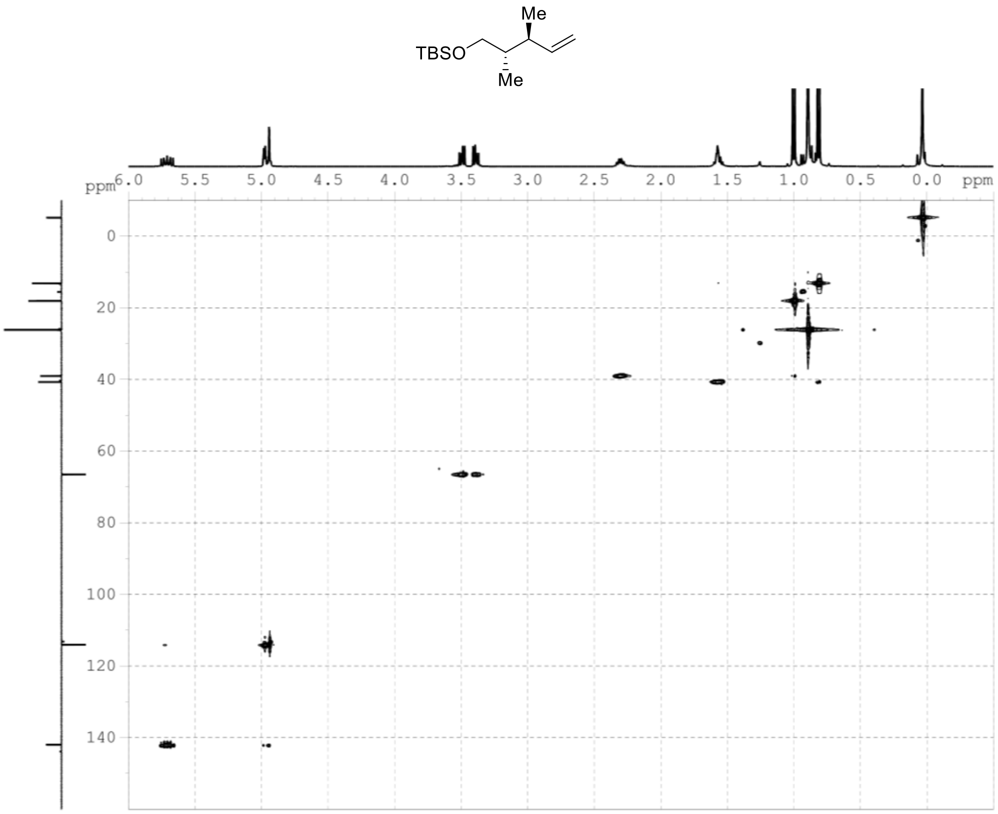

HMBC spectrum of **13** (400 MHz for ^{1}H NMR and 100 MHz for ^{13}C{^{1}H} NMR, $CDCl_3$)

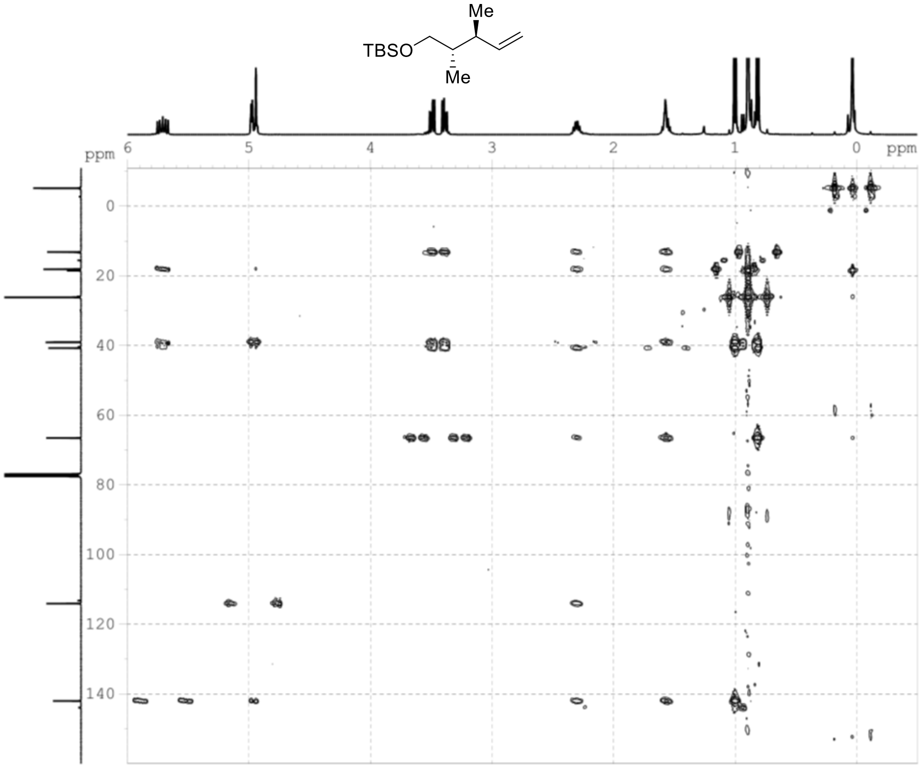

COSY spectrum of **13** (400 MHz, $CDCl_3$)

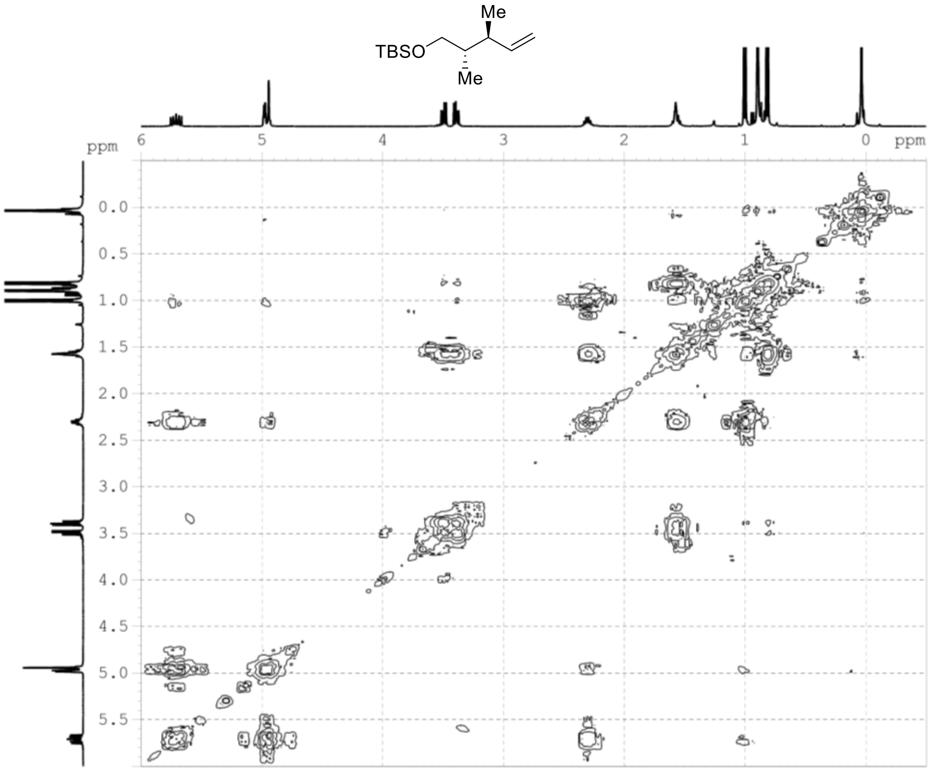

Optical rotation of **13**

Light Source	Na
Monitor wavelength	589 nm
D.I.T.	5 sec
No. of cycle	5
Cycle interval	1 sec
Temp. Monitor	Holder
Temp. Corr. Factor	0 at 25 C
Correct	Blank
Aperture(S)	8.0mm
Aperture(L)	Auto
Mode	Specific O.R.
Path Length	10 mm
Concentration	0.97 w/v%
Water content of sample	0 %
Factor	1

		Sample No.	Mode	Specific O.R.	Temperature(C)	Blank	Measurement Date	Comment
1	*	374F1-1	Specific O.R.	-6.9691	24.01	0.0004	8/8/2025 10:19 AM	
2	*	374F1-2	Specific O.R.	-7.4845	24.01	0.0004	8/8/2025 10:19 AM	
3	*	374F1-3	Specific O.R.	-7.4845	24.00	0.0004	8/8/2025 10:19 AM	
4	*	374F1-4	Specific O.R.	-7.2784	24.00	0.0004	8/8/2025 10:19 AM	
5	*	374F1-5	Specific O.R.	-7.0722	23.99	0.0004	8/8/2025 10:19 AM	
6	*	Avg.		-7.2577				
7		S.D		0.2351				
8		C.V		3.2391				

^{1}H NMR spectrum of **14** (400 MHz, acetone-d_6)

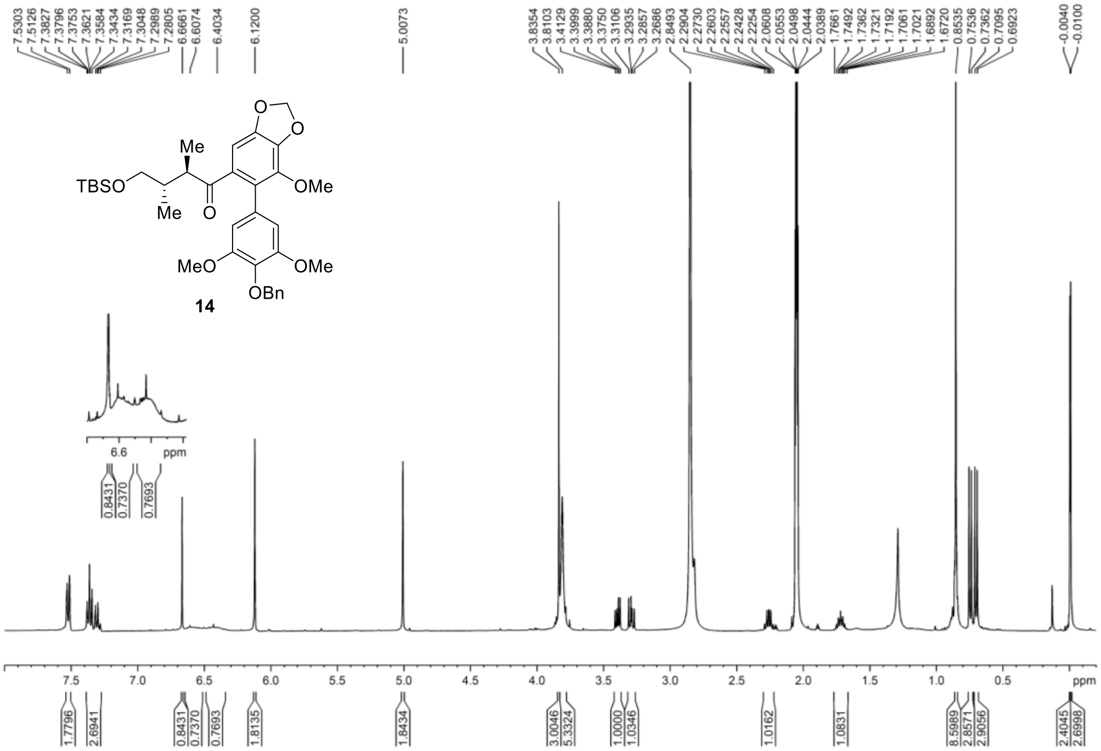

$^{13}C\{^{1}H\}$ NMR spectrum of **14** (100 MHz, acetone-d_6)

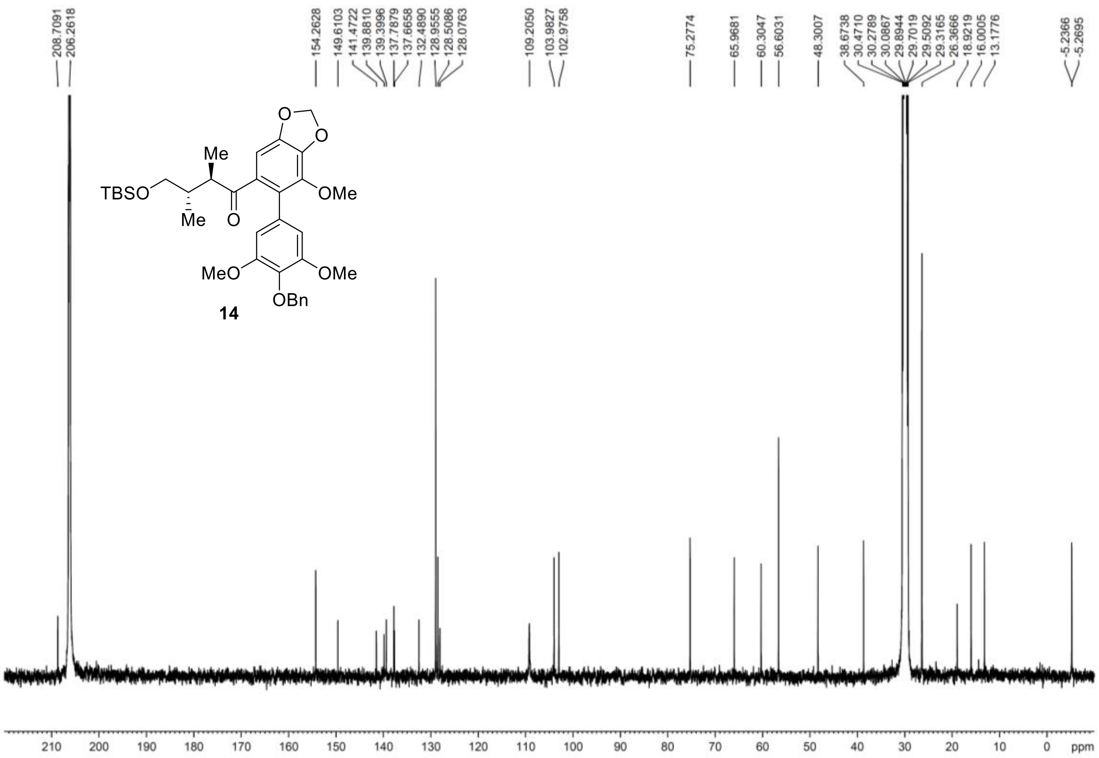

DEPT-135 NMR spectrum of **14** (100 MHz, acetone-d_6)

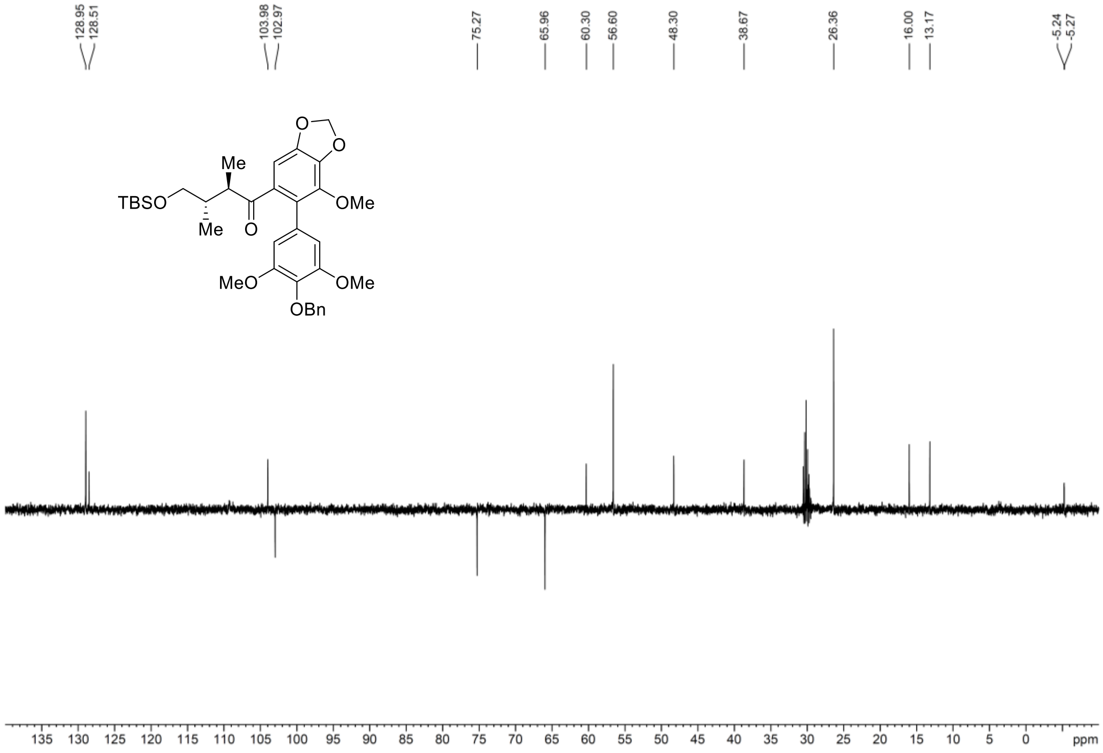

HSQC spectrum of **14** (400 MHz for ^{1}H NMR and 100 MHz for ^{13}C{^{1}H} NMR, acetone-d_6)

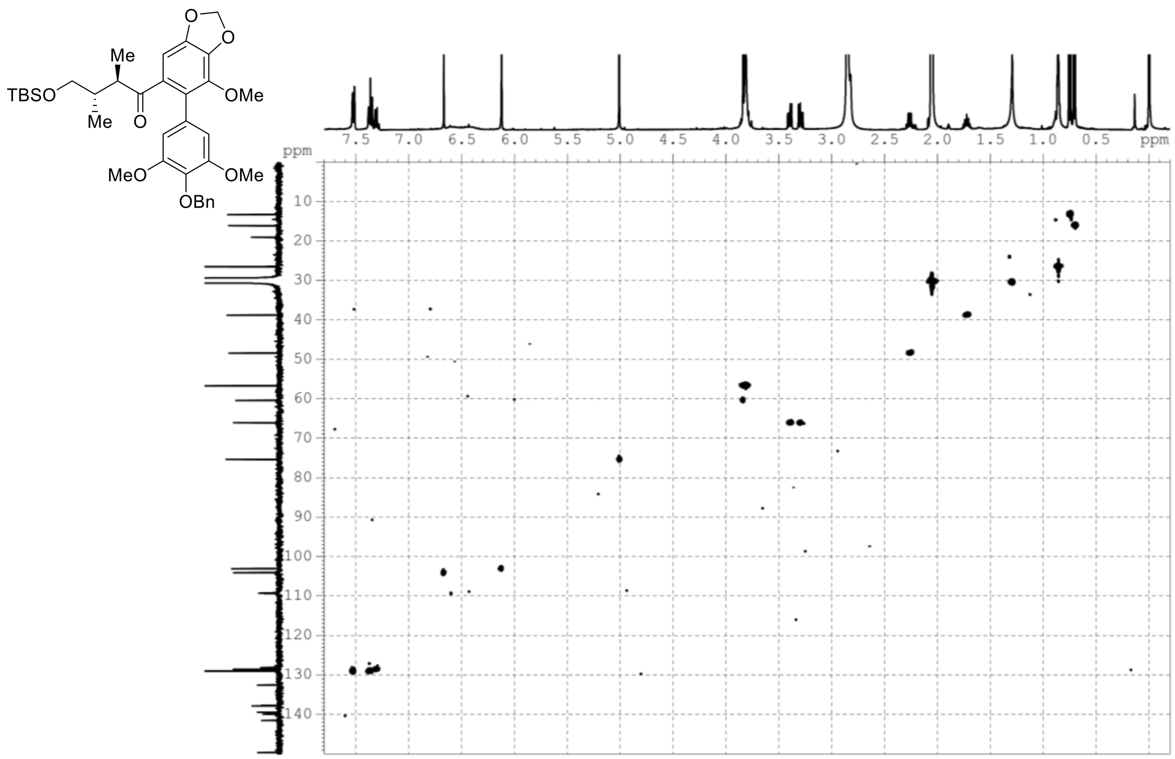

HMBC spectrum of **14** (400 MHz for ^{1}H NMR and 100 MHz for ^{13}C{^{1}H} NMR, acetone-d_6)

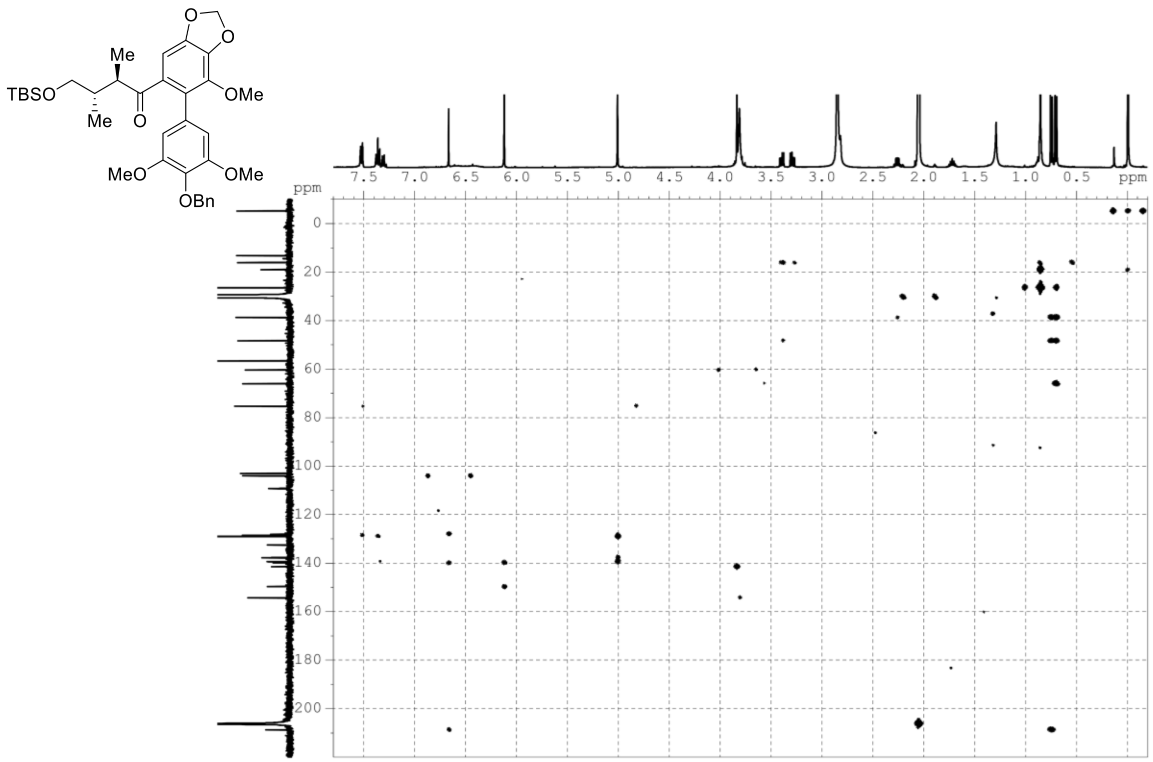

COSY spectrum of **14** (400 MHz, acetone-d_6)

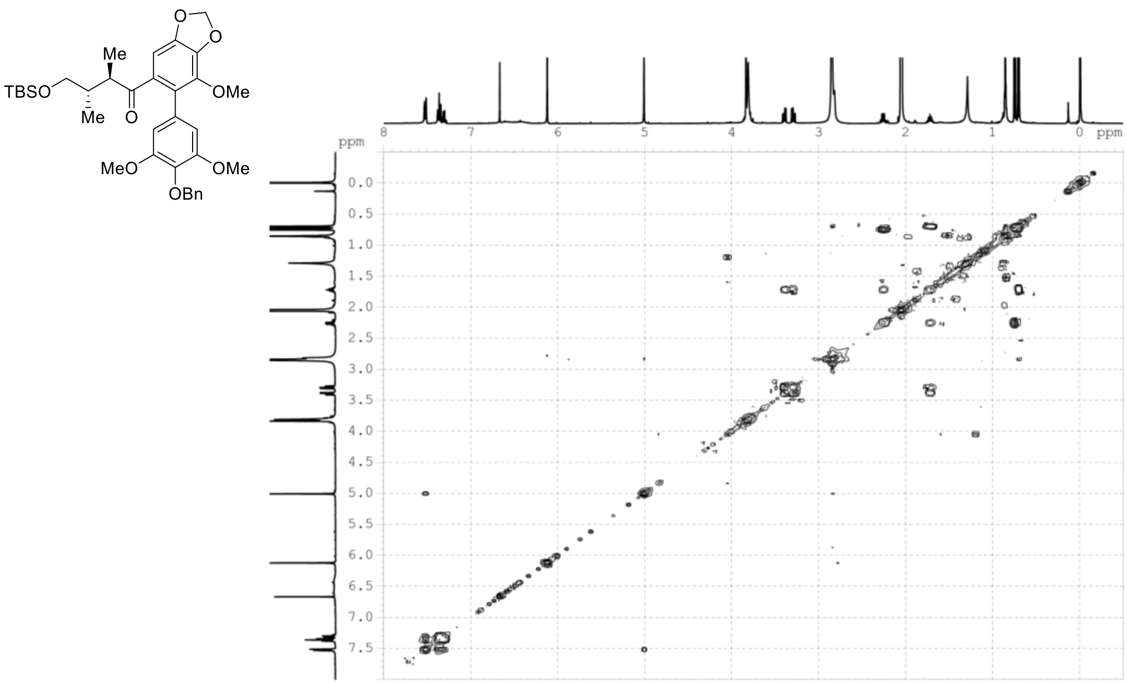

NOESY of **14** (400 MHz, acetone-d_6)

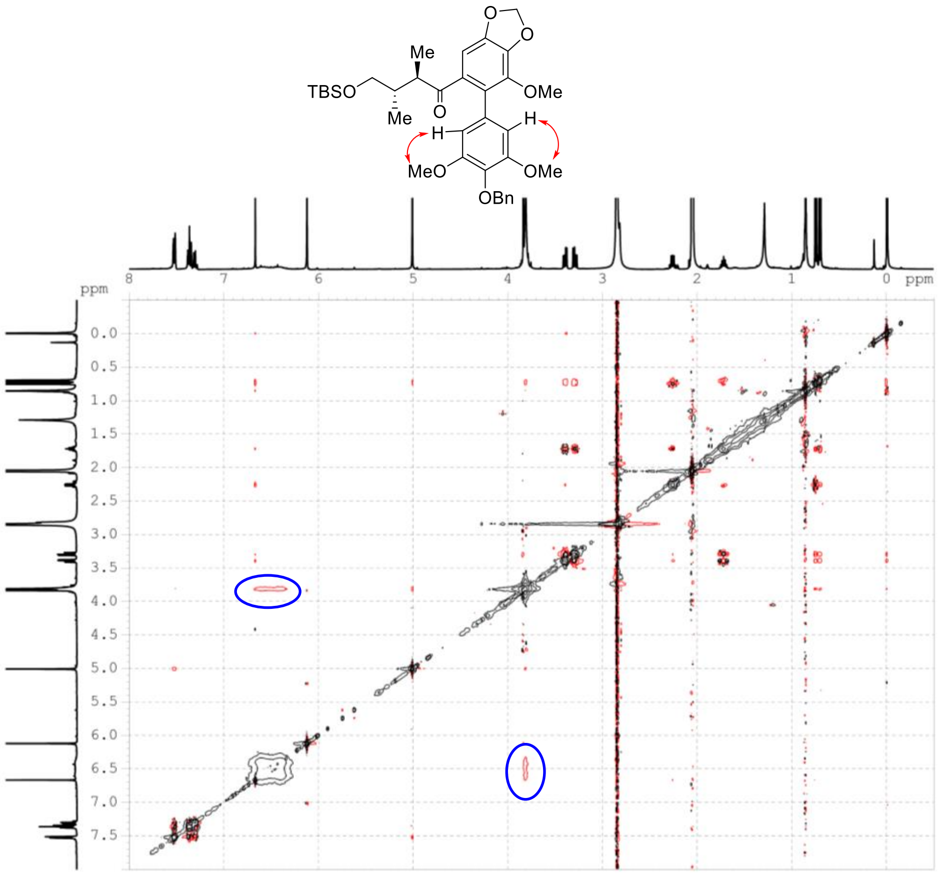

Optical rotation of **14**

Light Source	Na
Monitor wavelength	589 nm
D.I.T.	5 sec
No. of cycle	5
Cycle interval	1 sec
Temp. Monitor	Holder
Temp. Corr. Factor	0 at 25 C
Correct	Blank
Aperture(S)	8.0mm
Aperture(L)	Auto
Mode	Specific O.R.
Path Length	10 mm
Concentration	1.12 w/v%
Water content of sample	0 %
Factor	1

		Sample No.	Mode	Specific O.R.	Temperature(C)	Blank	Measurement Date	Comment
1	*	365F1-1	Specific O.R.	-18.9464	22.88	0.0006	6/13/2025 11:29 AM	
2	*	365F1-2	Specific O.R.	-19.7500	22.88	0.0006	6/13/2025 11:29 AM	
3	*	365F1-3	Specific O.R.	-19.3036	22.88	0.0006	6/13/2025 11:29 AM	
4	*	365F1-4	Specific O.R.	-19.5714	22.88	0.0006	6/13/2025 11:29 AM	
5	*	365F1-5	Specific O.R.	-19.3929	22.88	0.0006	6/13/2025 11:29 AM	
6	*	Avg.		-19.3929				
7		S.D		0.3028				
8		C.V		1.5613				

^{1}H NMR spectrum of **7a** (400 MHz, acetone-d_6)

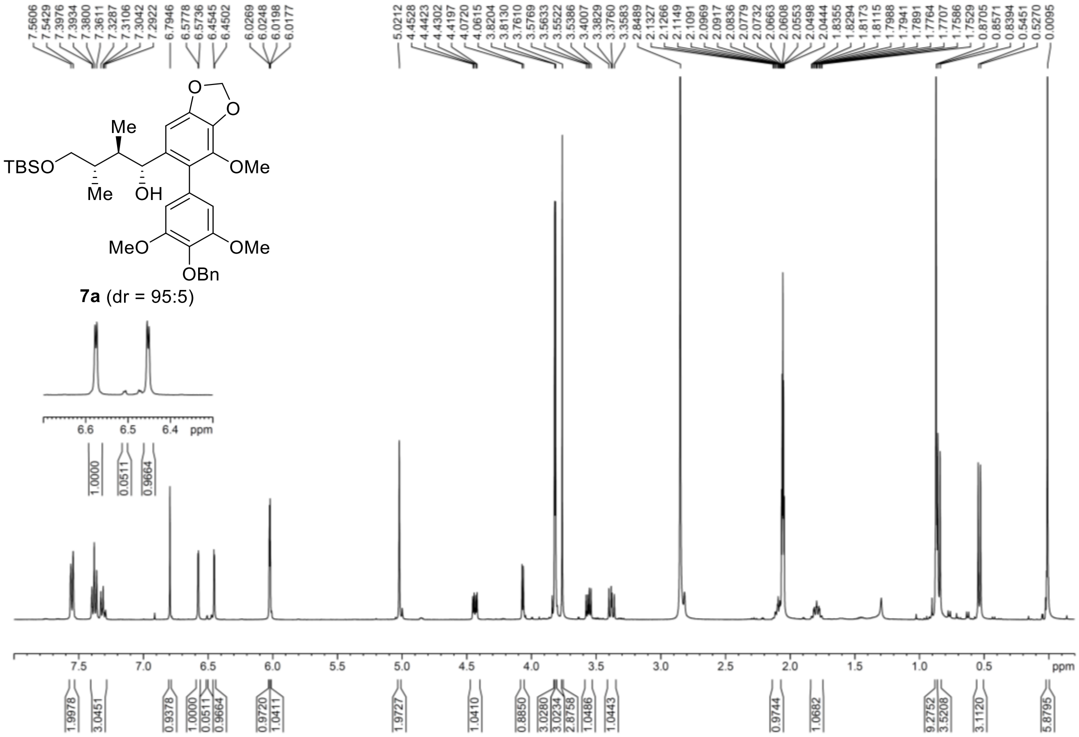

$^{13}C\{^{1}H\}$ NMR spectrum of **7a** (100 MHz, acetone-d_6)

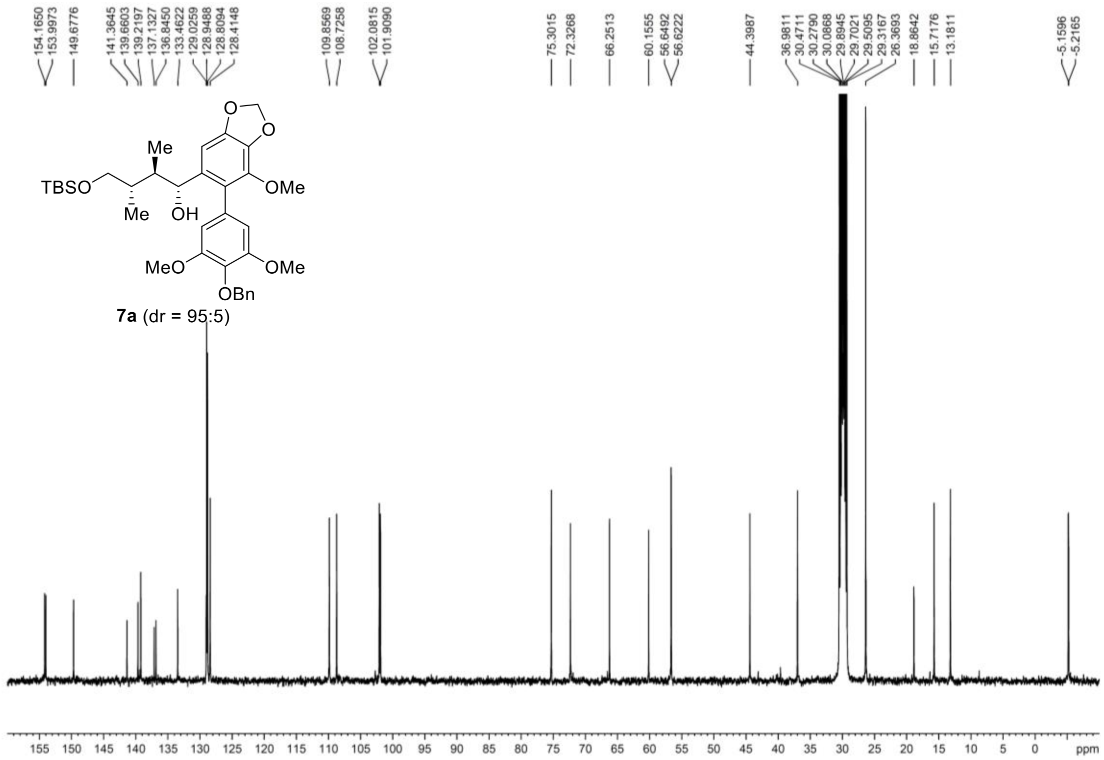

DEPT-135 NMR spectrum of **7a** (100 MHz, acetone-d_6)

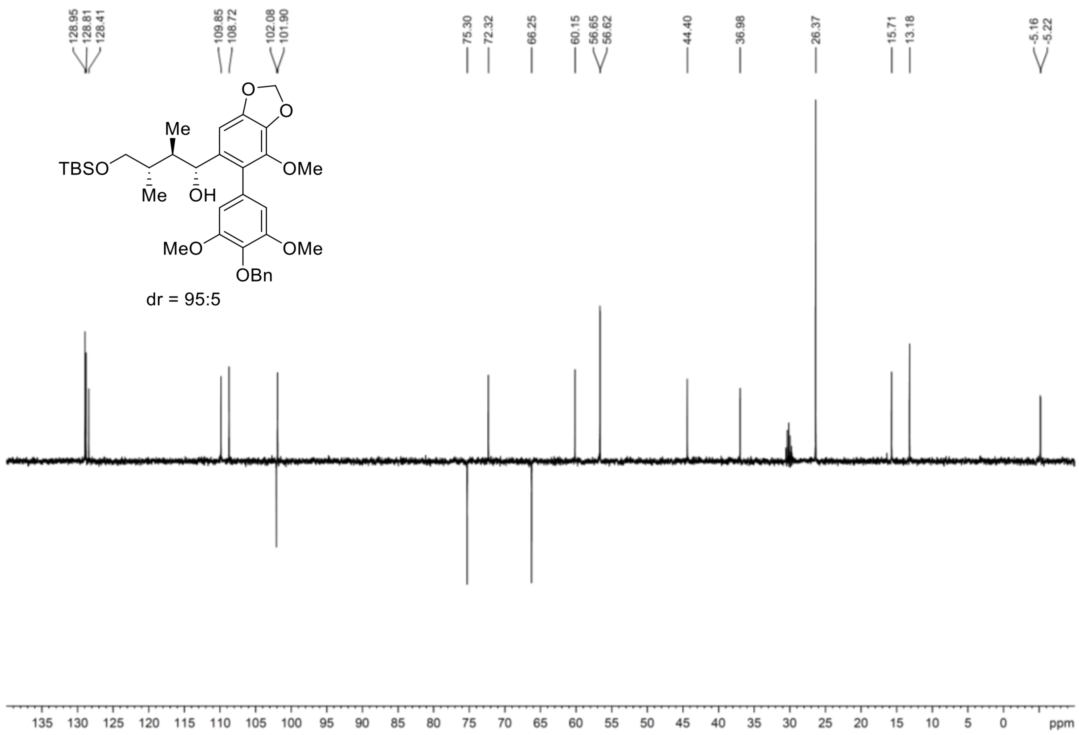

HSQC spectrum of **7a** (400 MHz for ^{1}H NMR and 100 MHz for $^{13}C\{^{1}H\}$ NMR, acetone-d_6)

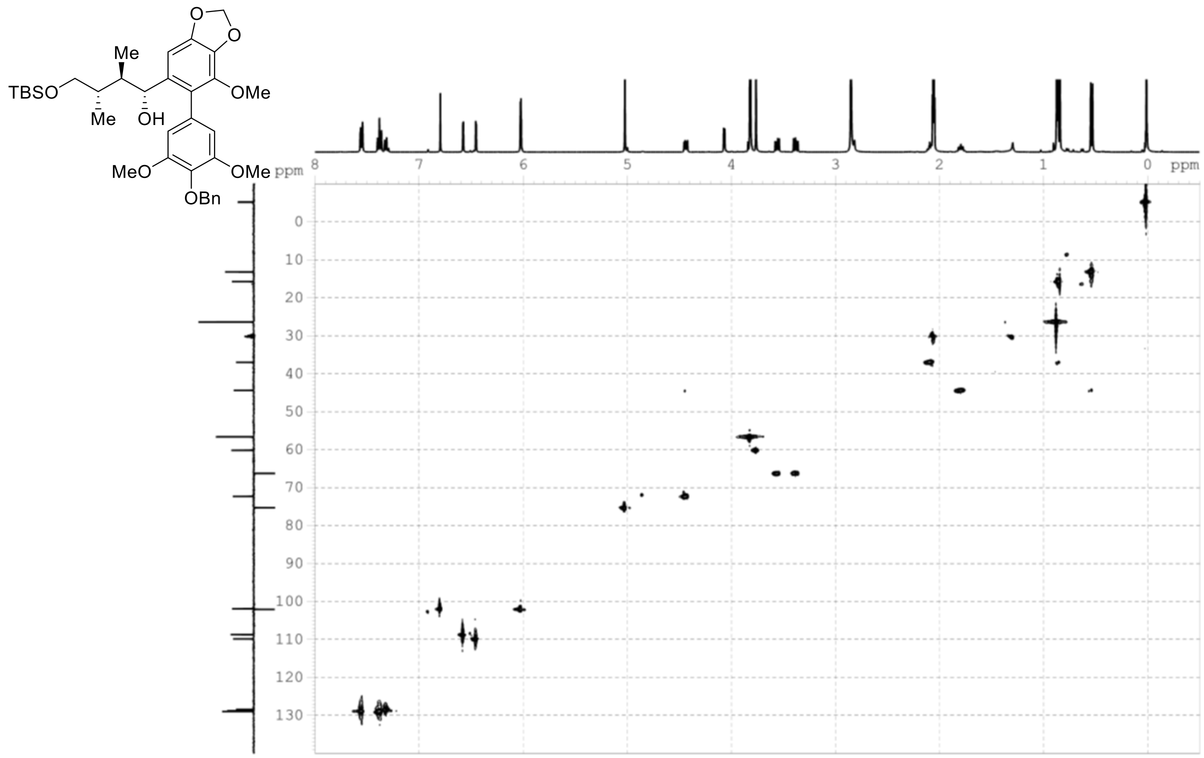

HMBC spectrum of **7a** (400 MHz for ^{1}H NMR and 100 MHz for $^{13}C\{^{1}H\}$ NMR, acetone-d_6)

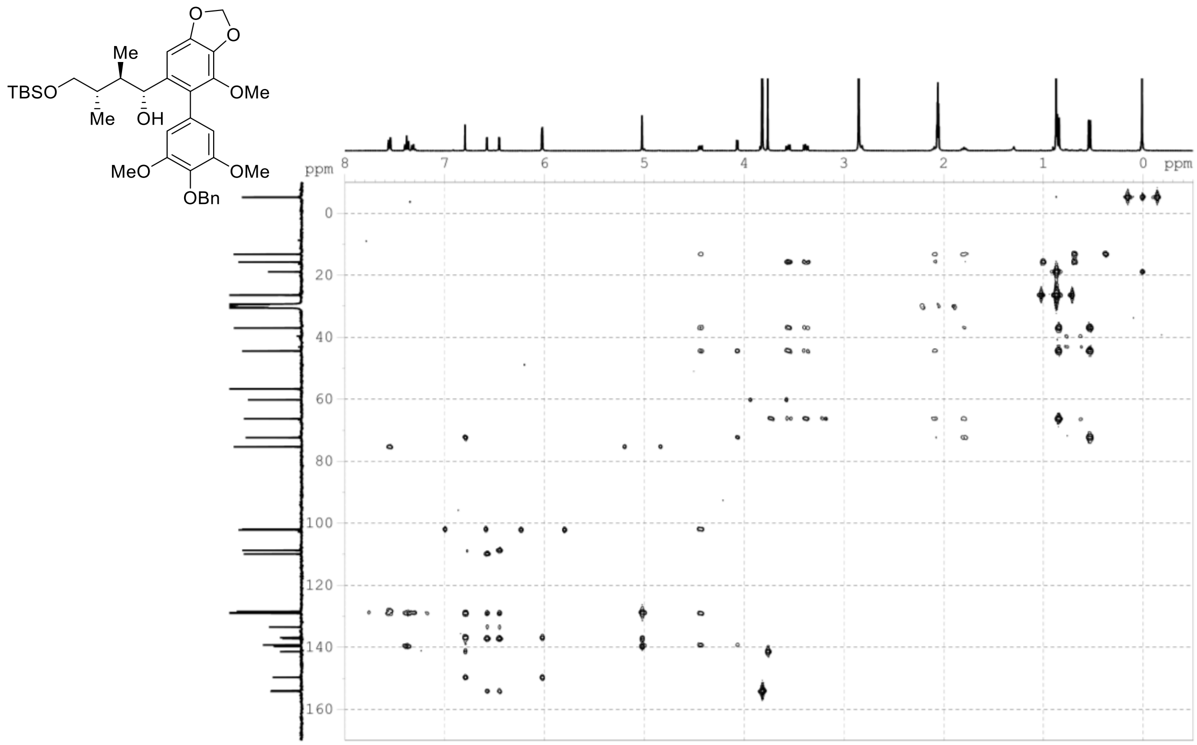

COSY spectrum of **7a** (400 MHz, acetone-d_6)

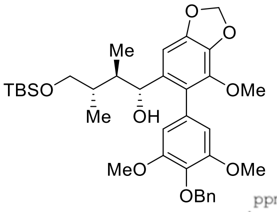

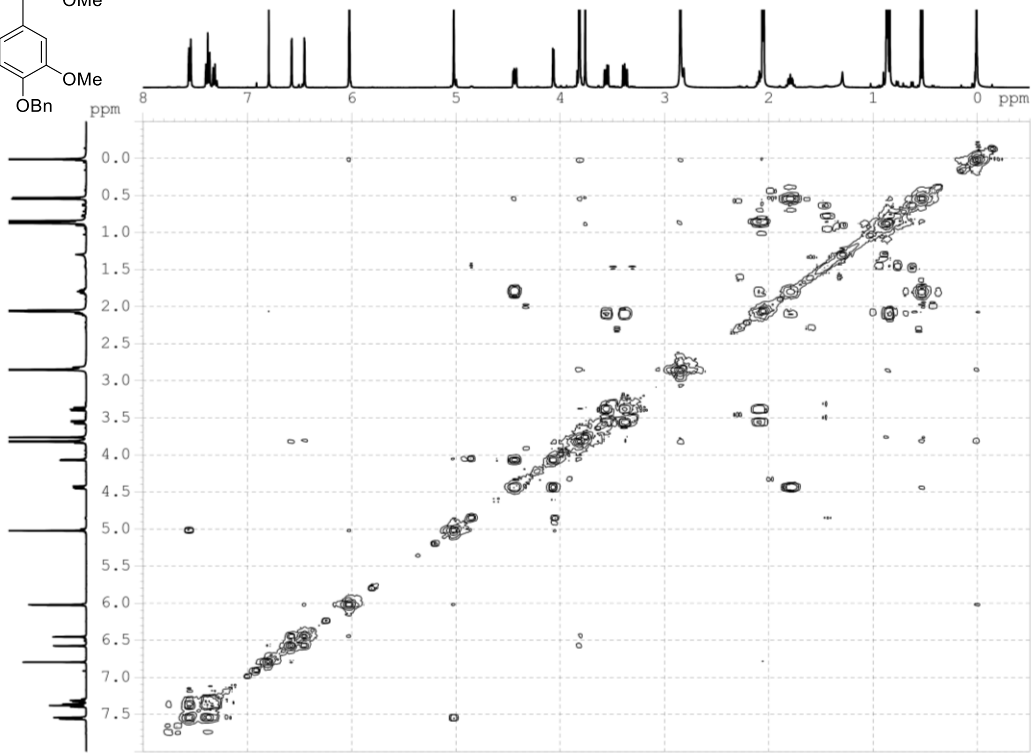

Optical rotation of **7a**

Light Source	Na
Monitor wavelength	589 nm
D.I.T.	5 sec
No. of cycle	5
Cycle interval	1 sec
Temp. Monitor	Holder
Temp. Corr. Factor	0 at 25 C
Correct	Blank
Aperture(S)	8.0mm
Aperture(L)	Auto
Mode	Specific O.R.
Path Length	10 mm
Concentration	1.92 w/v%
Water content of sample	0 %
Factor	1

		Sample No.	Mode	Specific O.R.	Temperature(C)	Blank	Measurement Date	Comment
1	*	398F2-1	Specific O.R.	-30.2813	26.03	-0.0001	11/7/2025 10:47 AM	
2	*	398F2-2	Specific O.R.	-30.3333	26.02	-0.0001	11/7/2025 10:47 AM	
3	*	398F2-3	Specific O.R.	-30.4896	26.01	-0.0001	11/7/2025 10:47 AM	
4	*	398F2-4	Specific O.R.	-30.5417	26.01	-0.0001	11/7/2025 10:47 AM	
5	*	398F2-5	Specific O.R.	-30.7500	26.00	-0.0001	11/7/2025 10:48 AM	
6	*	Avg.		-30.4792				
7		S.D		0.1856				
8		C.V		0.6090				

^{1}H NMR spectrum of **6a** (400 MHz, $CDCl_3$)

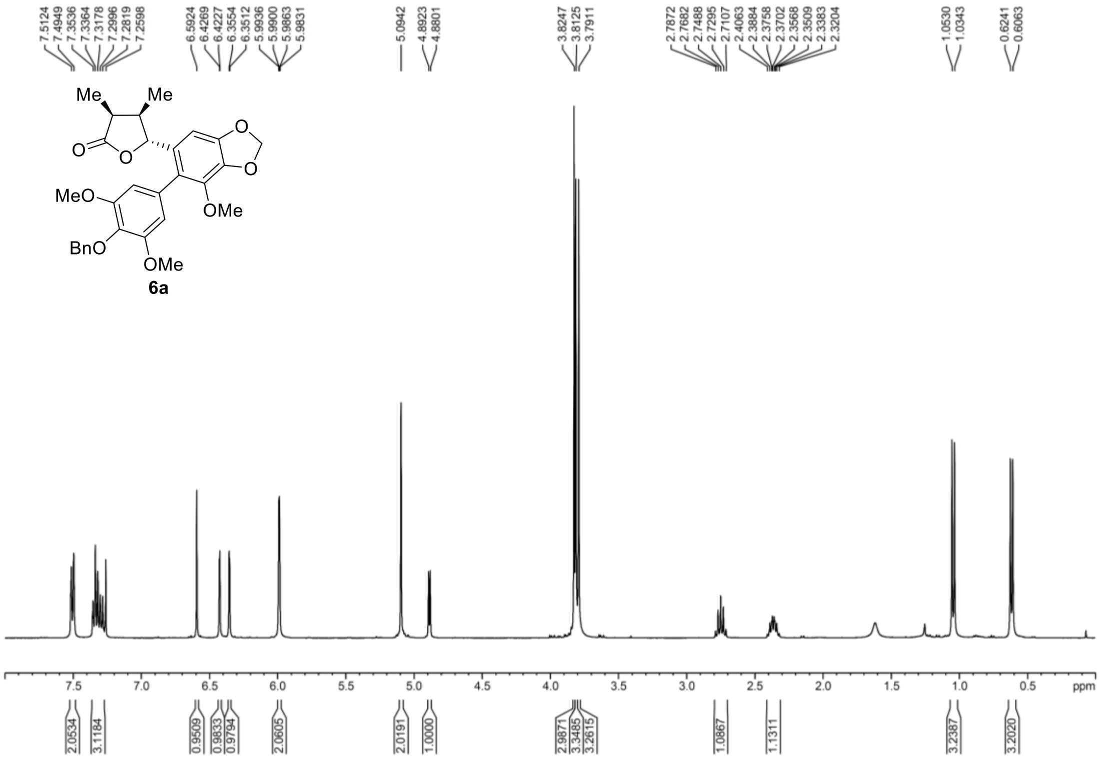

^{13}C{^{1}H} NMR spectrum of **6a** (100 MHz, $CDCl_3$)

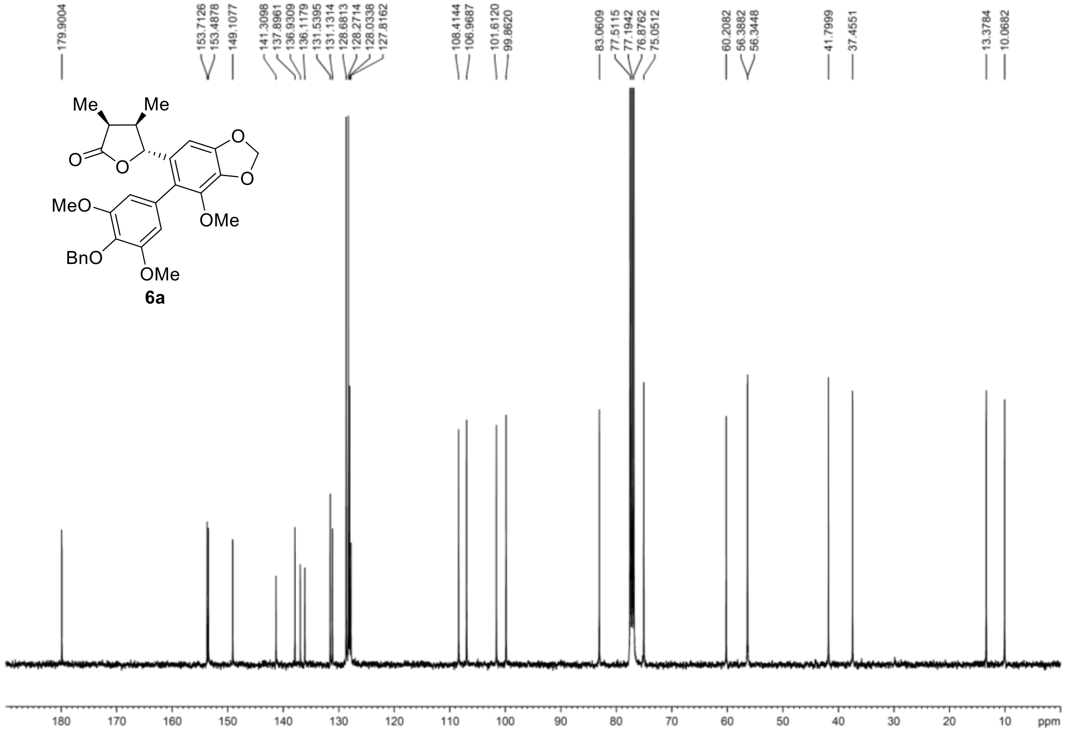

DEPT-135 NMR spectrum of **6a** (100 MHz, $CDCl_3$)

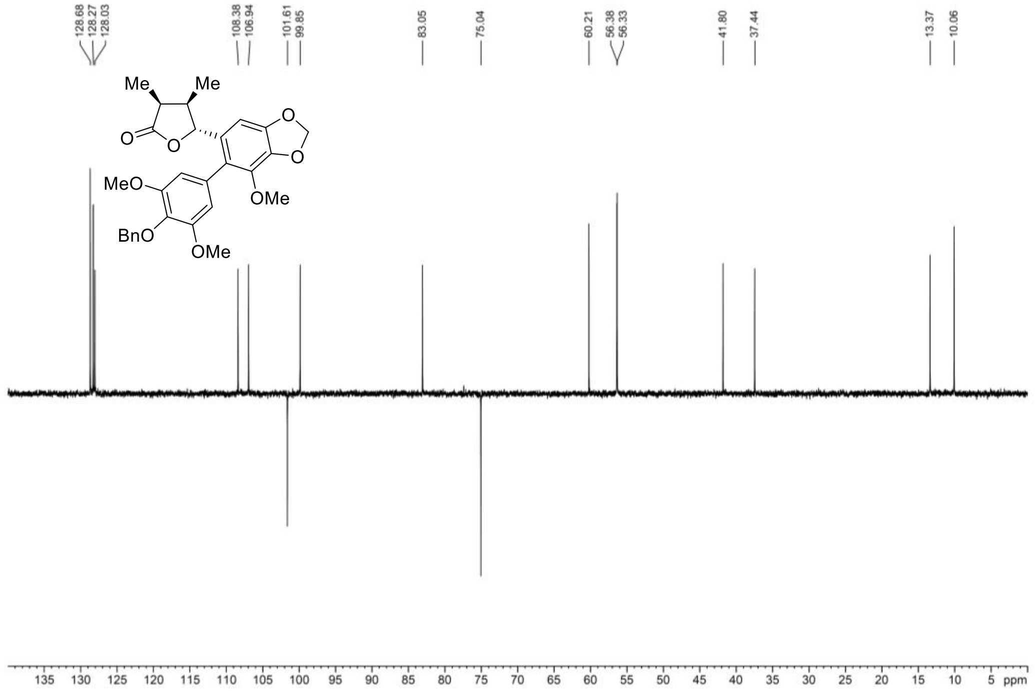

HSQC spectrum of **6a** (400 MHz for ^{1}H NMR and 100 MHz for $^{13}C\{^1H\}$ NMR, $CDCl_3$)

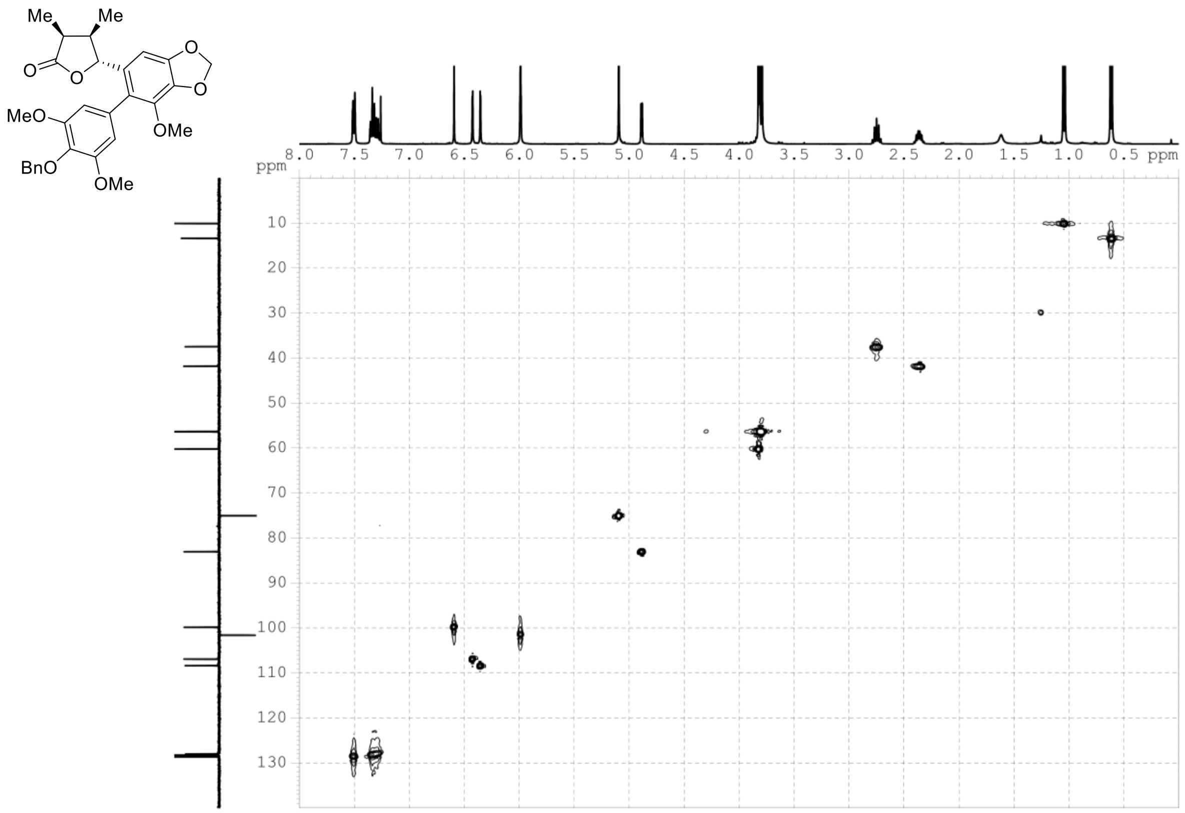

HMBC spectrum of **6a** (400 MHz for ^{1}H NMR and 100 MHz for $^{13}C\{^{1}H\}$ NMR, $CDCl_3$)

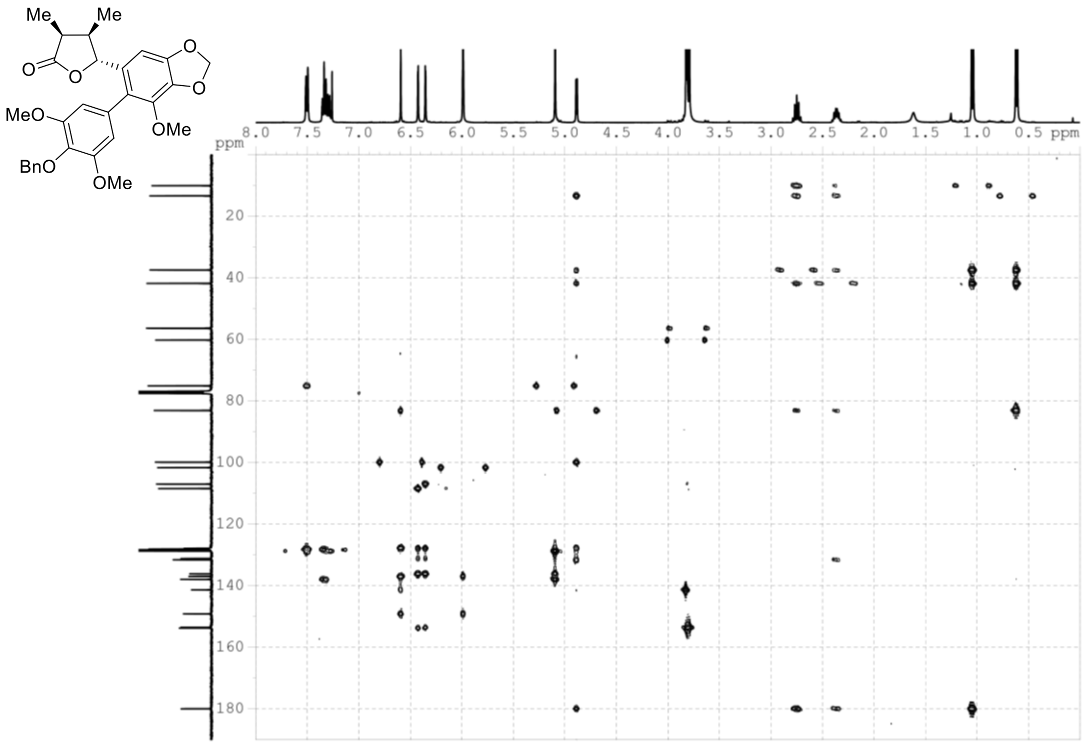

Key COSY correlations of **6a** (400 MHz, $CDCl_3$)

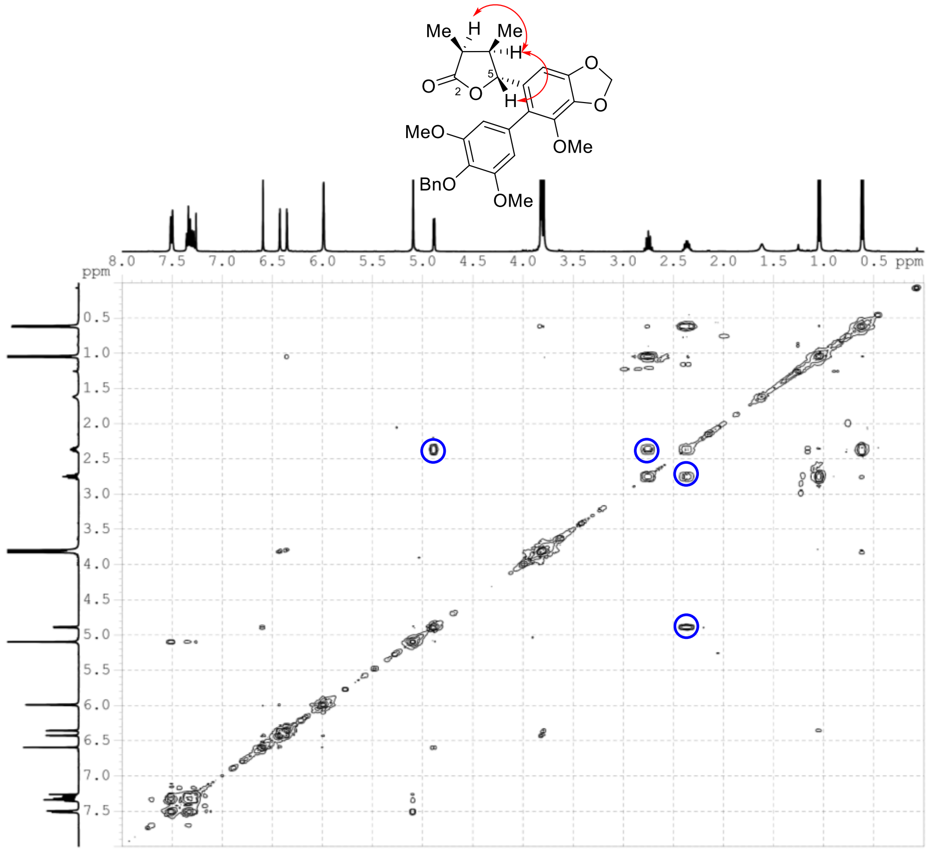

Key NOESY correlations of **6a** (400 MHz, $CDCl_3$)

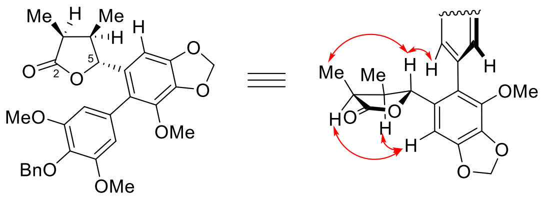

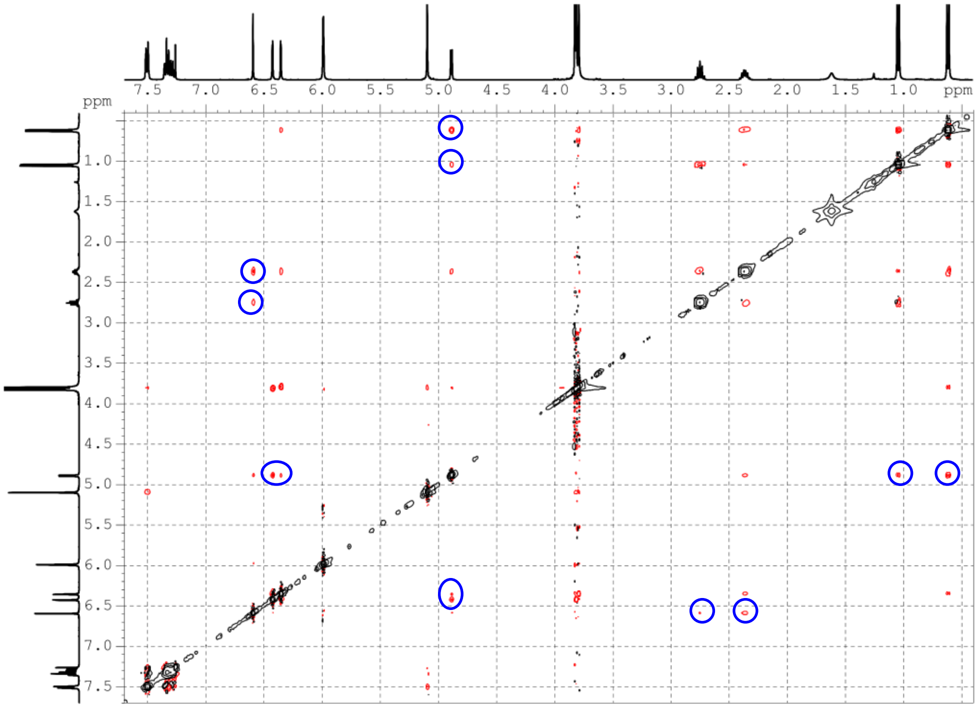

Optical rotation of **6a**

Light Source	Na
Monitor wavelength	589 nm
D.I.T.	5 sec
No. of cycle	5
Cycle interval	1 sec
Temp. Monitor	Holder
Temp. Corr. Factor	0 at 25 C
Correct	Blank
Aperture(S)	8.0mm
Aperture(L)	Auto
Mode	Specific O.R.
Path Length	10 mm
Concentration	0.52 w/v%
Water content of sample	0 %
Factor	1

		Sample No.	Mode	Specific O.R.	Temperature(C)	Blank	Measurement Date	Comment
1	*	378F9-1	Specific O.R.	-23.1154	23.11	0.0006	6/13/2025 11:08 AM	
2	*	378F9-2	Specific O.R.	-23.6923	23.11	0.0006	6/13/2025 11:08 AM	
3	*	378F9-3	Specific O.R.	-23.3077	23.11	0.0006	6/13/2025 11:08 AM	
4	*	378F9-4	Specific O.R.	-23.1154	23.11	0.0006	6/13/2025 11:08 AM	
5	*	378F9-5	Specific O.R.	-22.3462	23.11	0.0006	6/13/2025 11:08 AM	
6	*	Avg.		-23.1154				
7		S.D		0.4903				
8		C.V		2.1211				

^{1}H NMR spectrum of **6b** (400 MHz, $CDCl_3$)

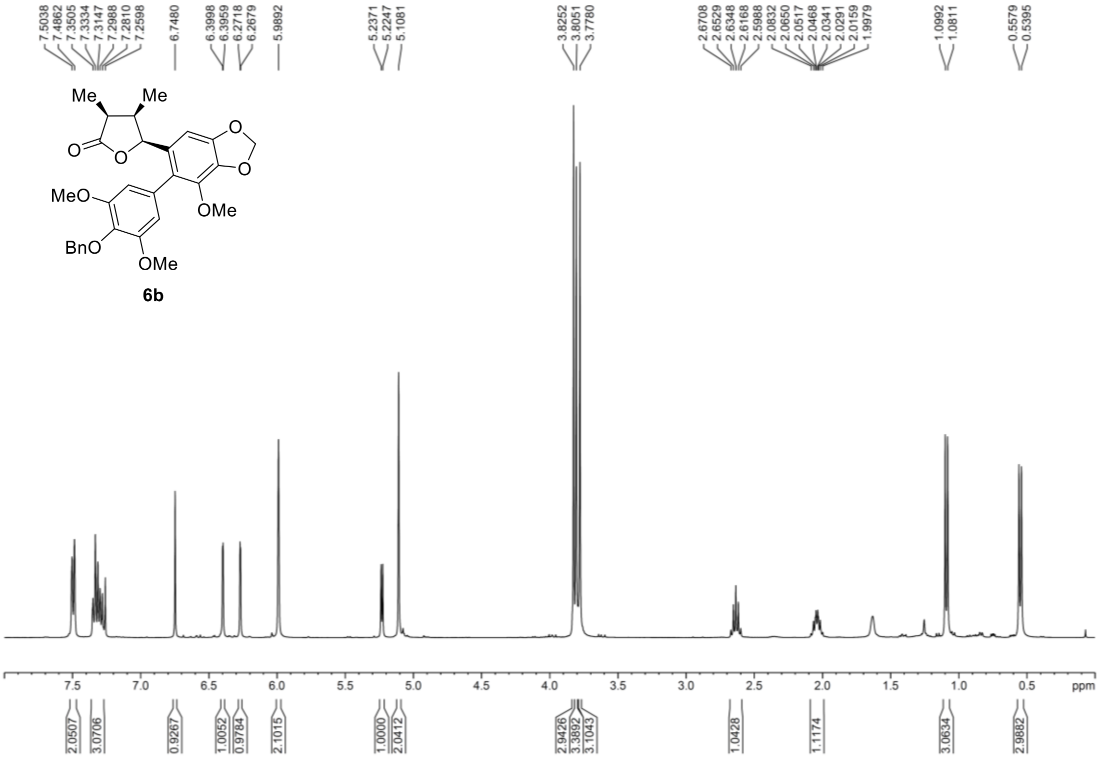

$^{13}C\{^{1}H\}$ NMR spectrum of **6b** (100 MHz, $CDCl_3$)

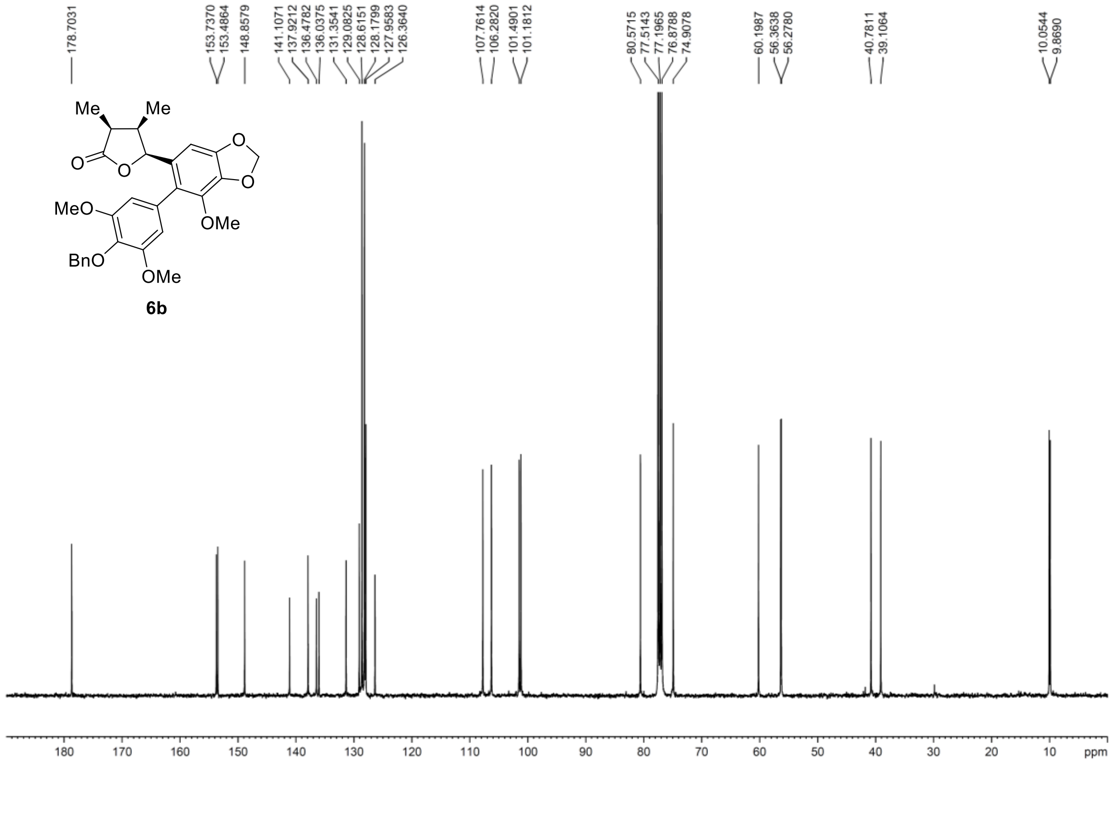

DEPT-135 NMR spectrum of **6b** (100 MHz, $CDCl_3$)

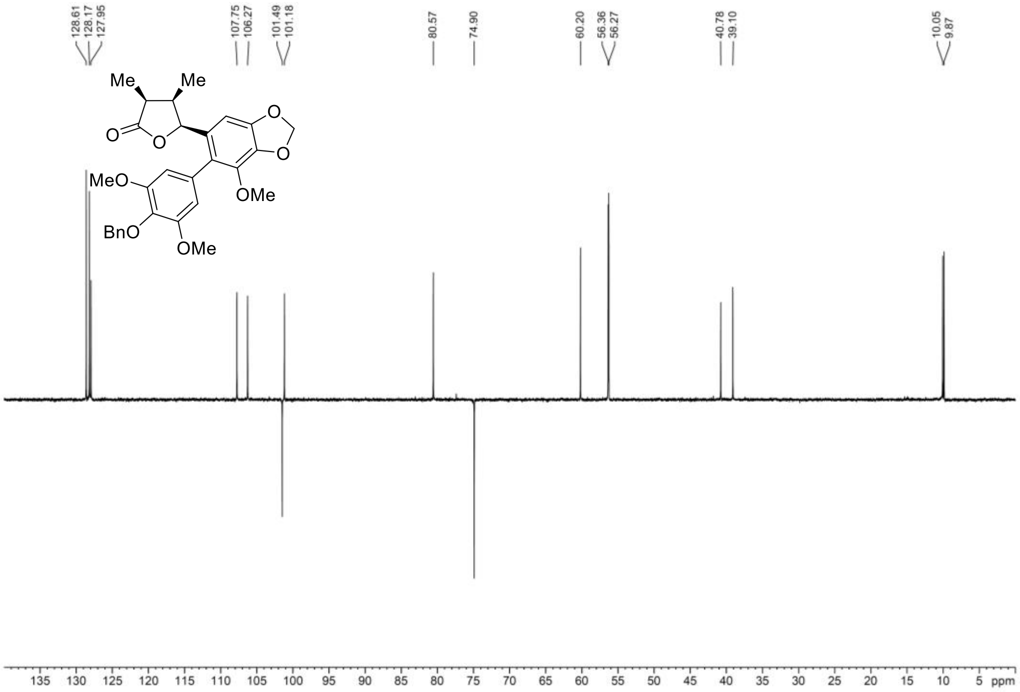

HSQC spectrum of **6b** (400 MHz for ^{1}H NMR and 100 MHz for $^{13}C\{^{1}H\}$ NMR, $CDCl_3$)

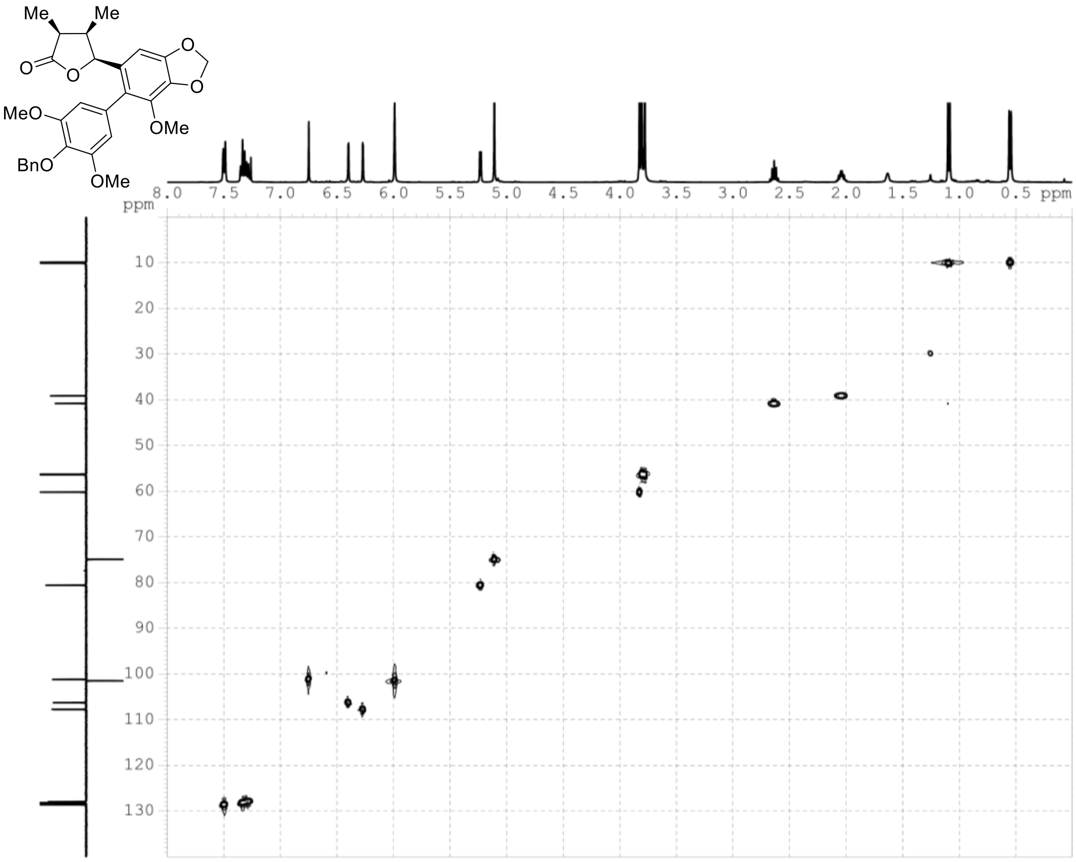

HMBC spectrum of **6b** (400 MHz for ^{1}H NMR and 100 MHz for $^{13}C\{^{1}H\}$ NMR, $CDCl_3$)

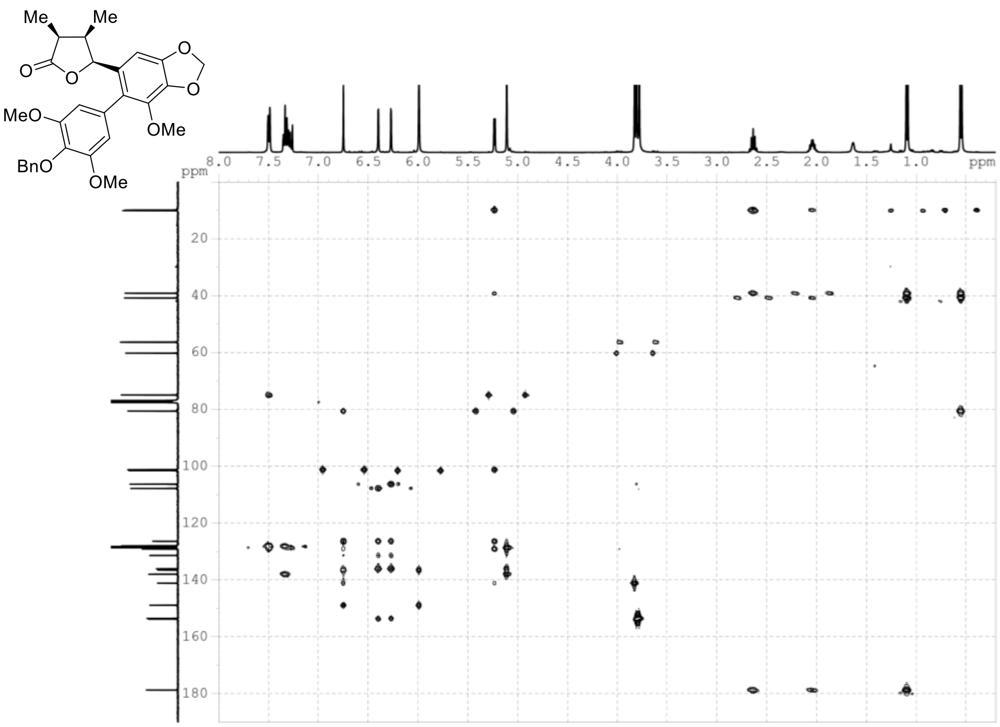

Key COSY correlations of **6b** (400 MHz, $CDCl_3$)

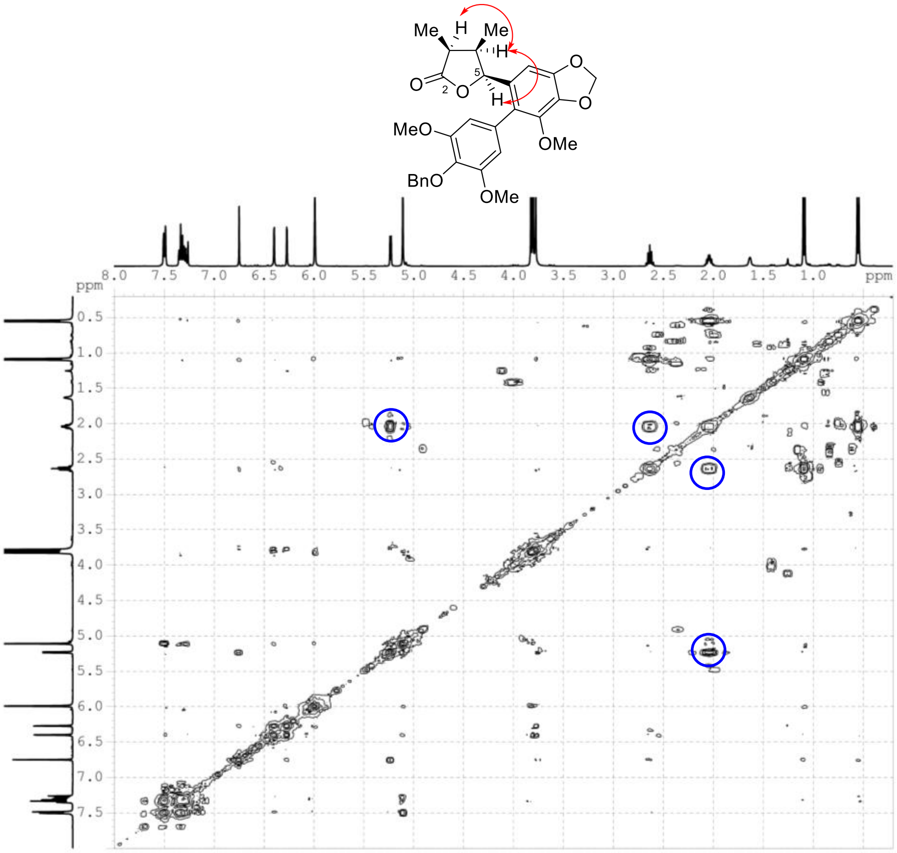

Key NOESY correlations of **6b** (400 MHz, $CDCl_3$)

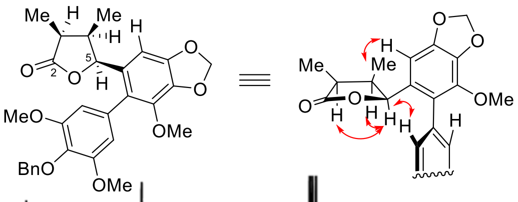

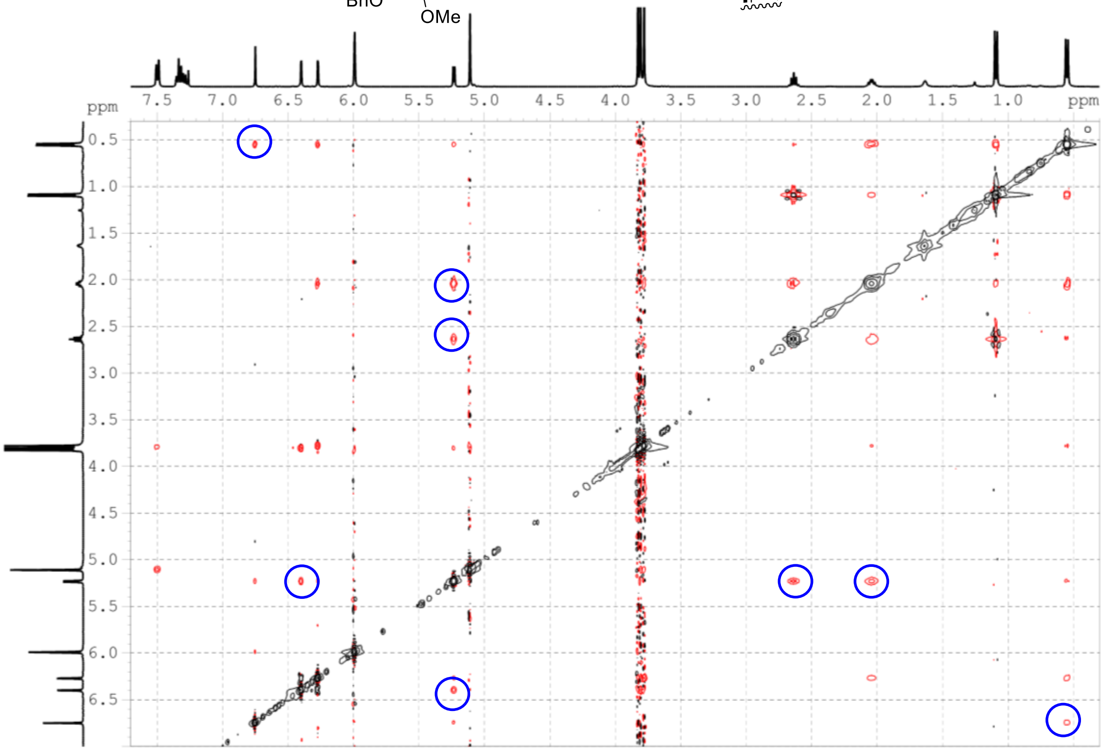

Optical rotation of **6b**

Light Source	Na
Monitor wavelength	589 nm
D.I.T.	5 sec
No. of cycle	5
Cycle interval	1 sec
Temp. Monitor	Holder
Temp. Corr. Factor	0 at 25 C
Correct	Blank
Aperture(S)	8.0mm
Aperture(L)	Auto
Mode	Specific O.R.
Path Length	10 mm
Concentration	1.18 w/v%
Water content of sample	0 %
Factor	1

		Sample No.	Mode	Specific O.R.	Temperature(C)	Blank	Measurement Date	Comment
1	*	378F6-1	Specific O.R.	-19.7627	23.02	0.0006	6/13/2025 10:40 AM	Acetone
2	*	378F6-2	Specific O.R.	-20.1017	23.02	0.0006	6/13/2025 10:40 AM	Acetone
3	*	378F6-3	Specific O.R.	-20.4407	23.02	0.0006	6/13/2025 10:40 AM	Acetone
4	*	378F6-4	Specific O.R.	-20.6949	23.02	0.0006	6/13/2025 10:40 AM	Acetone
5	*	378F6-5	Specific O.R.	-20.4407	23.02	0.0006	6/13/2025 10:40 AM	Acetone
6	*	Avg.		-20.2881				
7		S.D		0.3615				
8		C.V		1.7820				

^{1}H NMR spectrum of **1** (400 MHz, acetone-d_6)

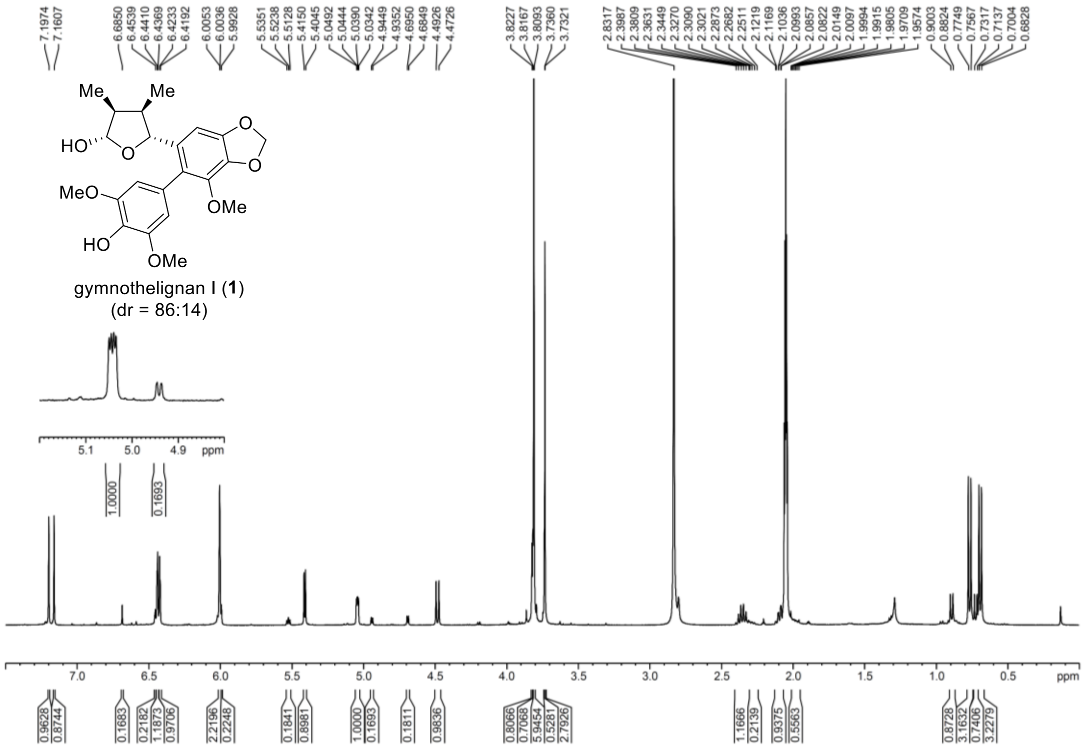

^{13}C{^{1}H} NMR spectrum of **1** (100 MHz, acetone-d_6)

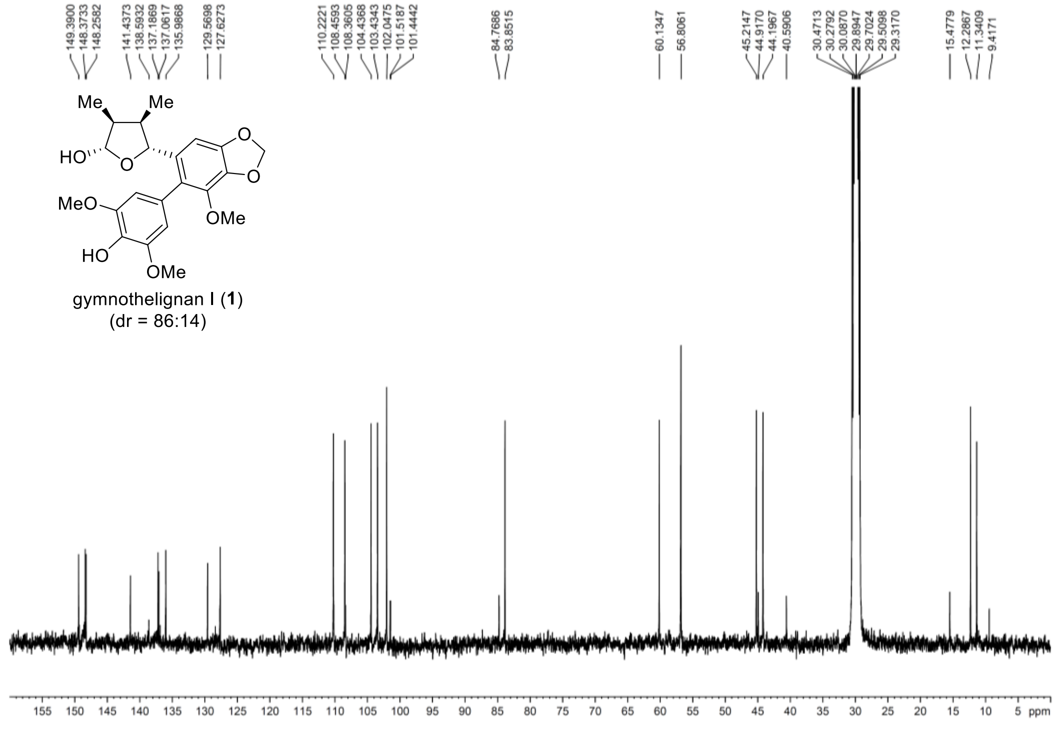

DEPT-135 NMR spectrum of **1** (100 MHz, acetone-d_6)

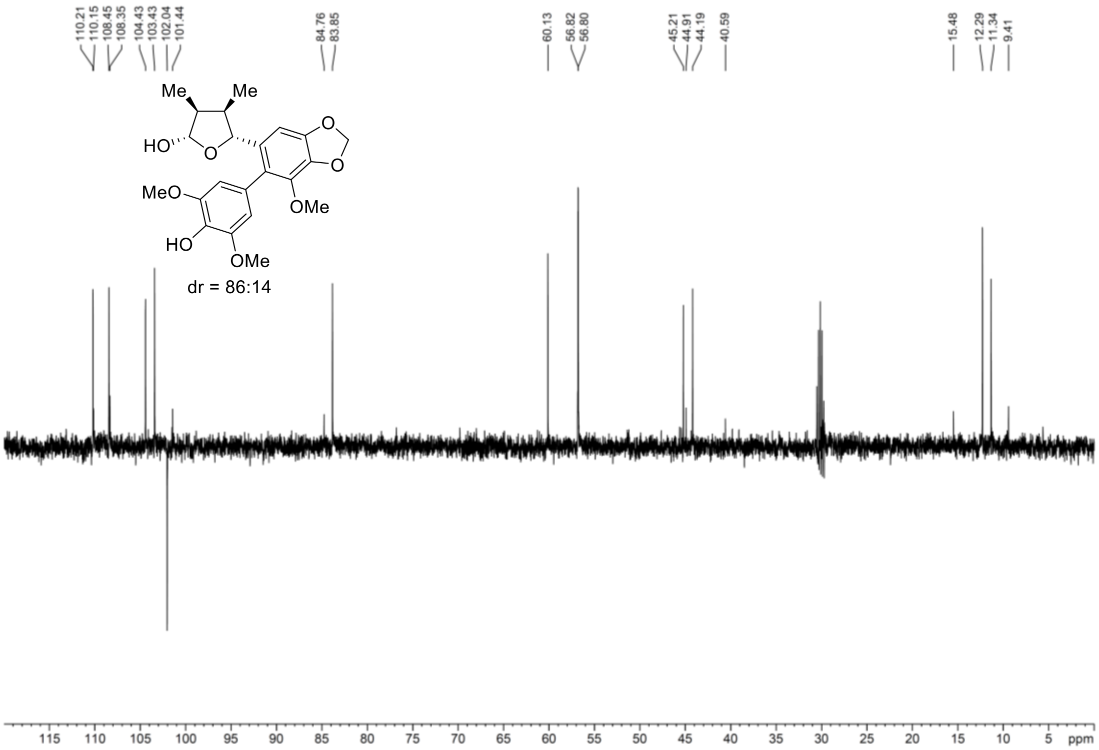

HSQC spectrum of **1** (400 MHz for ^{1}H NMR and 100 MHz for ^{13}C{^{1}H} NMR, acetone-d_6)

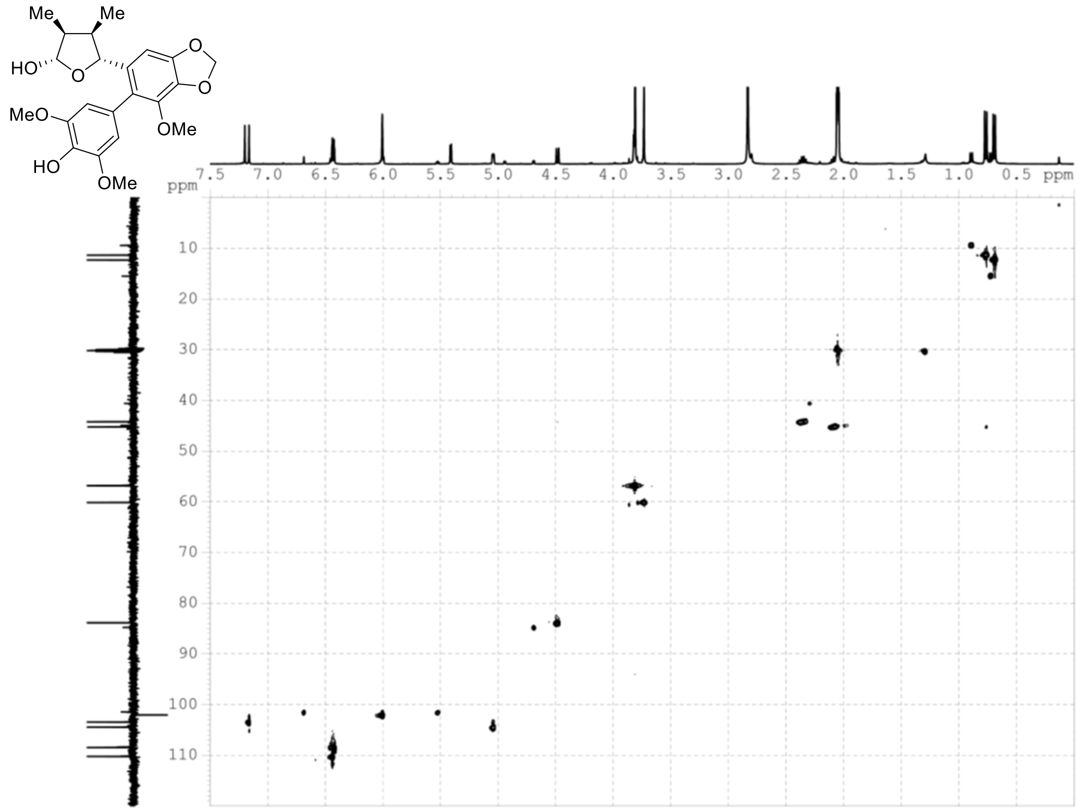

HMBC spectrum of **1** (400 MHz for ^{1}H NMR and 100 MHz for $^{13}C\{^{1}H\}$ NMR, acetone-d_6)

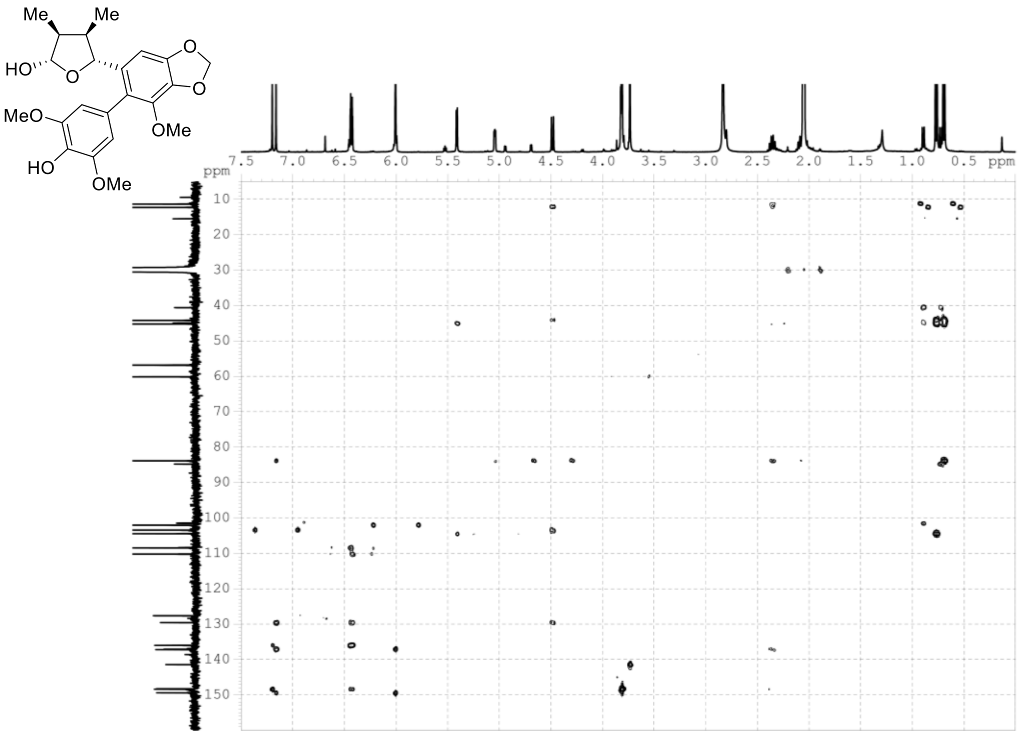

Key COSY correlations (400 MHz, acetone-d_6)

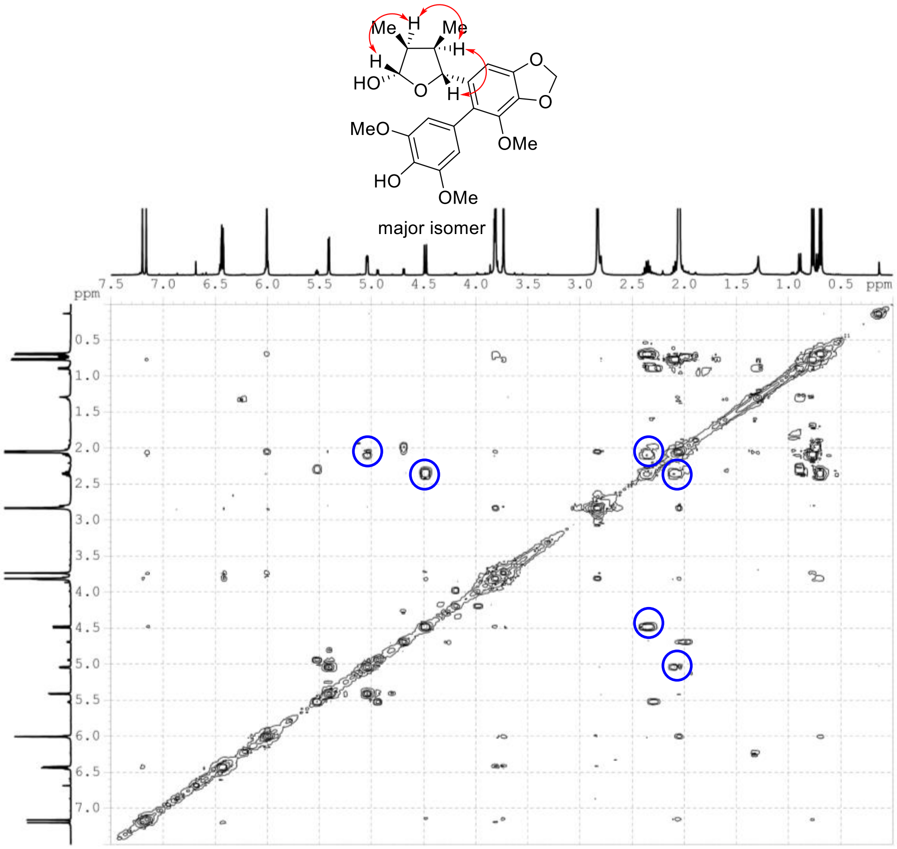

Key NOESY correlations of **1** (400 MHz, acetone-d_6)

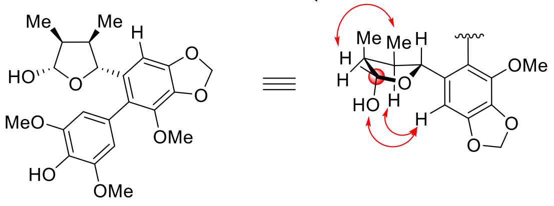

major isomer

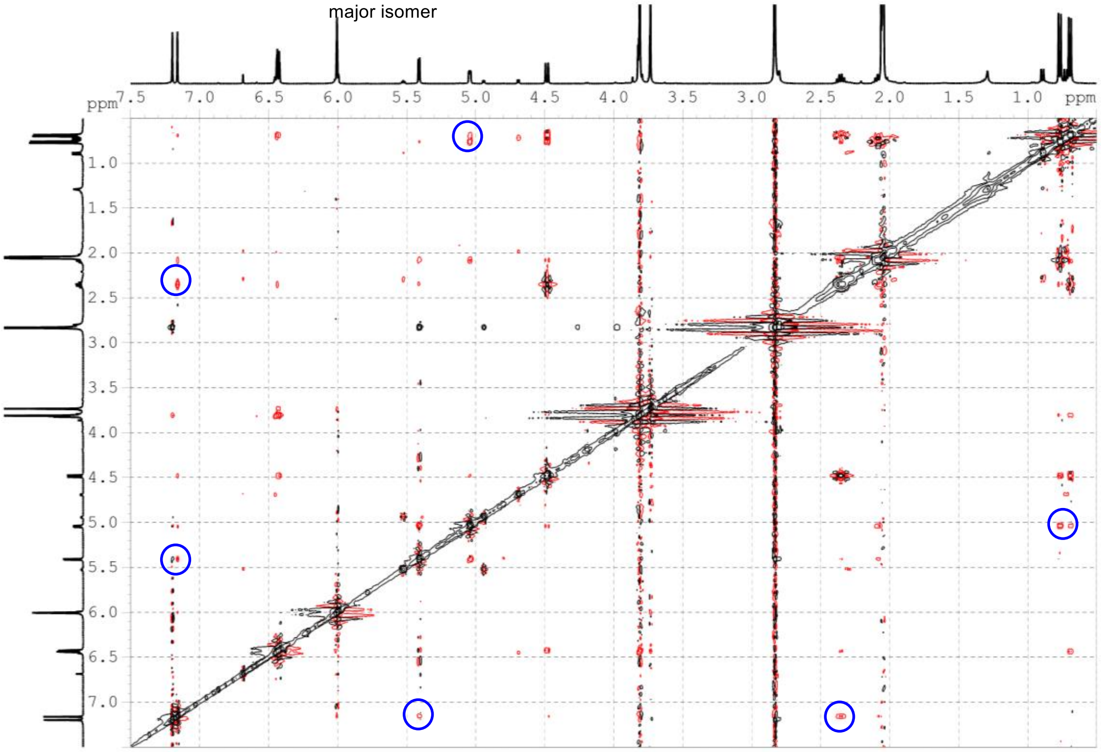

Optical rotation of **1**

Light Source	Na
Monitor wavelength	589 nm
D.I.T.	5 sec
No. of cycle	5
Cycle interval	1 sec
Temp. Monitor	Holder
Temp. Corr. Factor	0 at 25 C
Correct	Blank
Aperture(S)	8.0mm
Aperture(L)	Auto
Mode	Specific O.R.
Path Length	10 mm
Concentration	0.1 w/v%
Water content of sample	0 %
Factor	1

		Sample No.	Mode	Specific O.R.	Temperature(C)	Blank	Measurement Date	Comment
1	*	384-I1-1	Specific O.R.	-4.4000	24.95	0.0006	7/18/2025 10:59 AM	
2	*	384-I1-2	Specific O.R.	-4.4000	24.94	0.0006	7/18/2025 10:59 AM	
3	*	384-I1-3	Specific O.R.	-4.4000	24.93	0.0006	7/18/2025 10:59 AM	
4	*	384-I1-4	Specific O.R.	-4.4000	24.93	0.0006	7/18/2025 10:59 AM	
5	*	384-I1-5	Specific O.R.	-4.4000	24.92	0.0006	7/18/2025 10:59 AM	
6	*	Avg.		-4.4000				
7		S.D		0.0000				
8		C.V		0.0000				

^{1}H NMR spectrum of **2** (400 MHz, acetone-d_6)

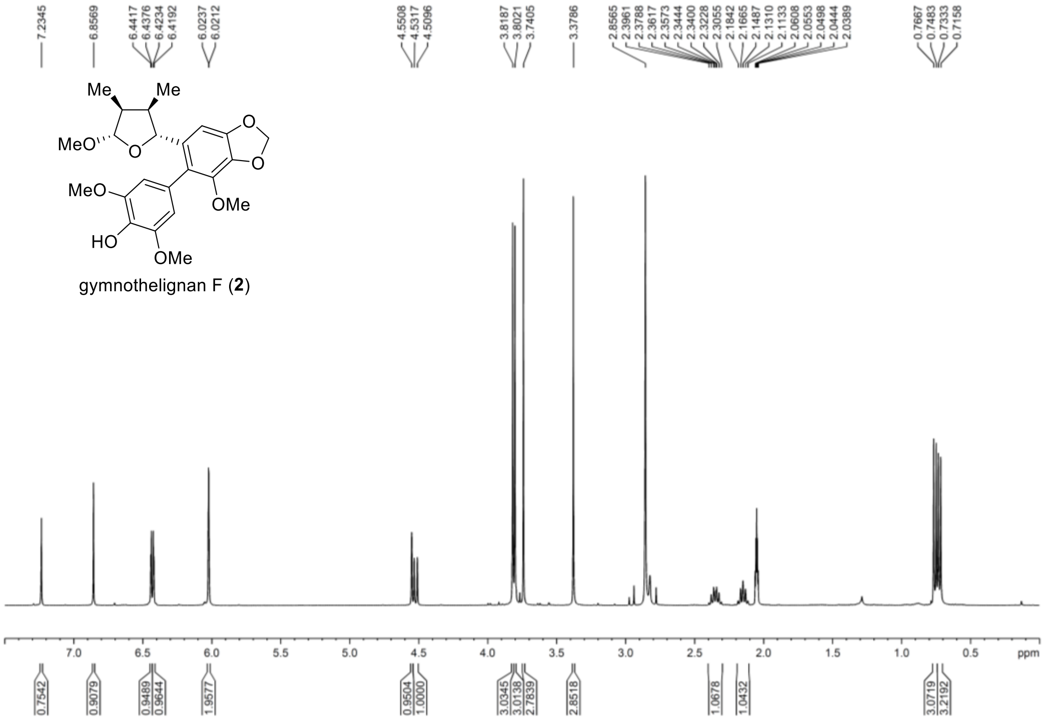

^{13}C{^{1}H} NMR spectrum of **2** (100 MHz, acetone-d_6)

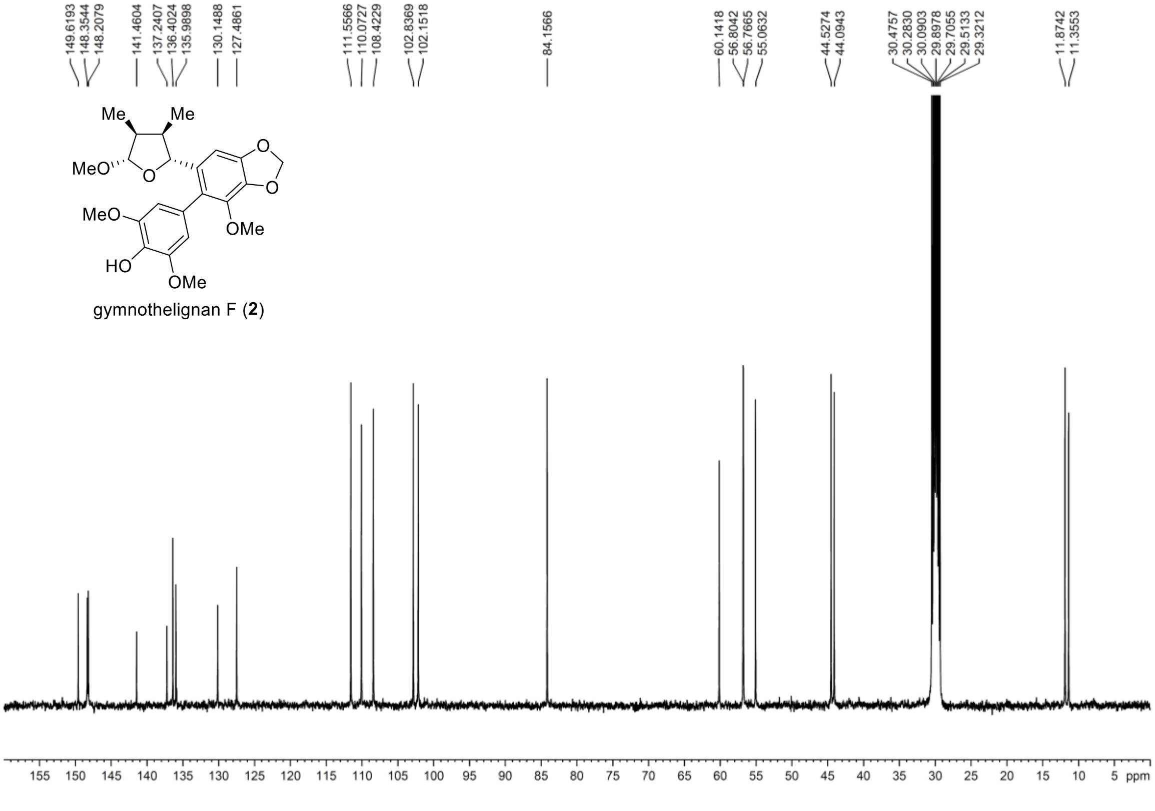

DEPT-135 NMR spectrum of **2** (100 MHz, acetone-d_6)

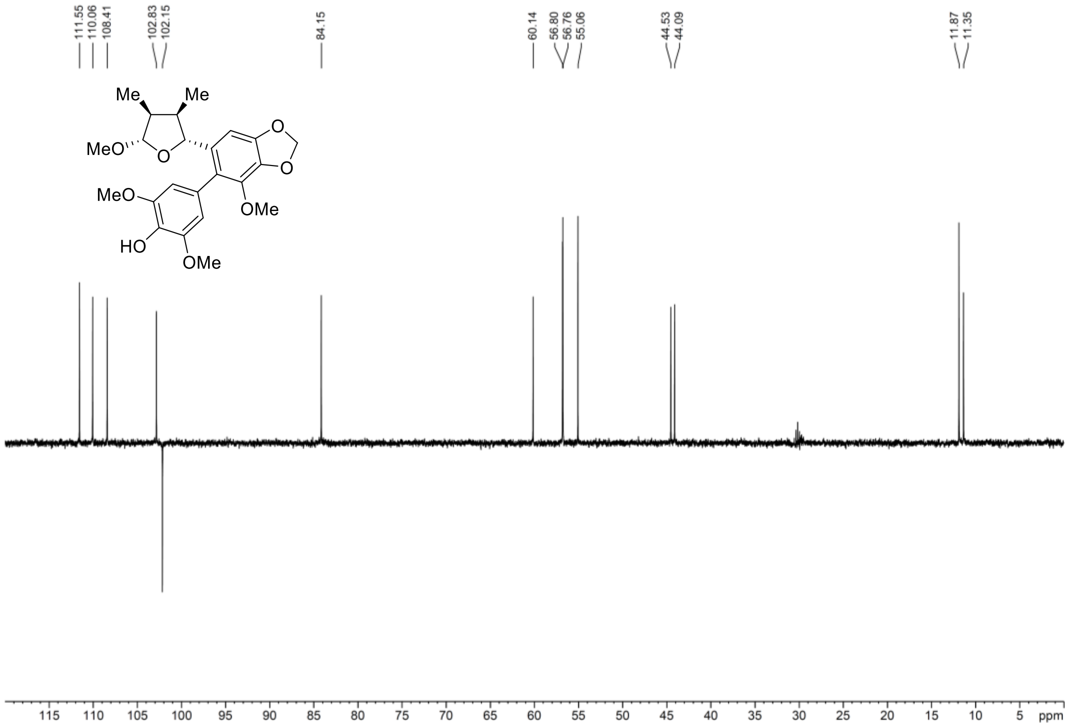

HSQC spectrum of **2** (400 MHz for ^{1}H NMR and 100 MHz for ^{13}C{^{1}H} NMR, acetone-d_6)

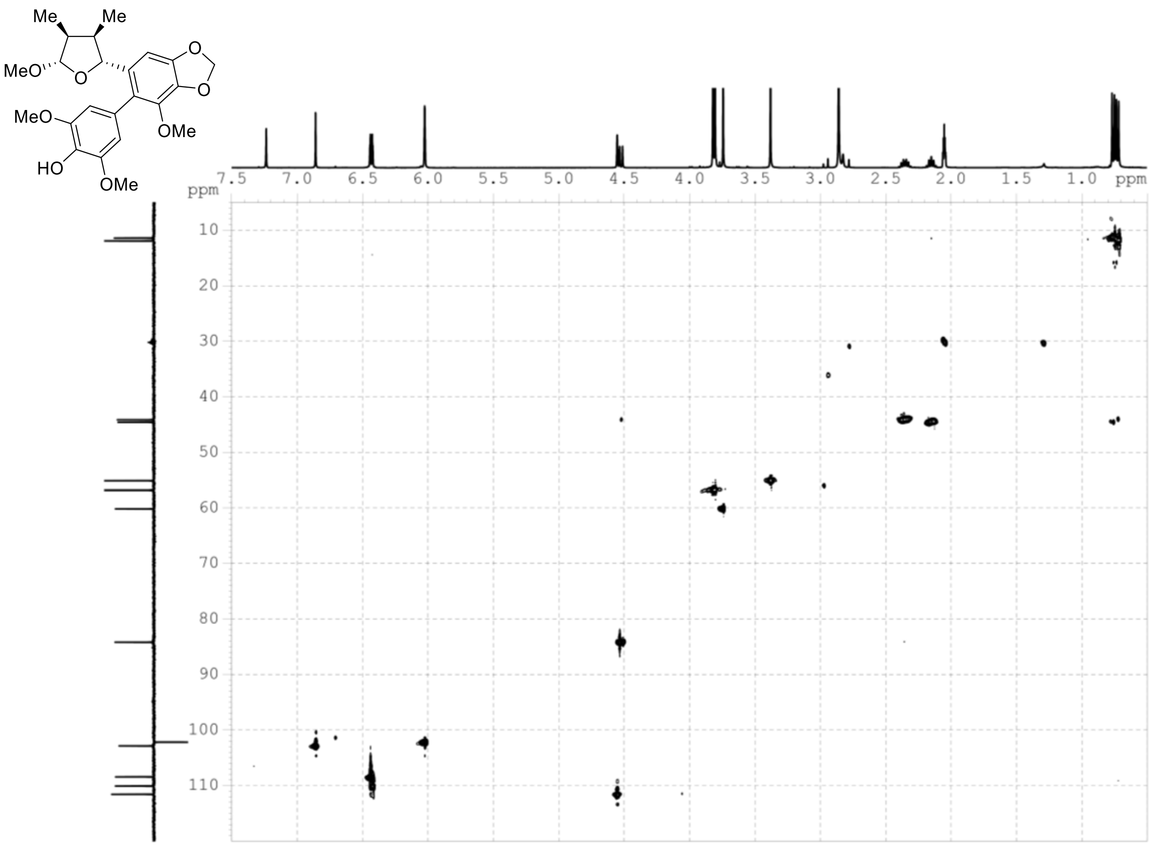

HMBC spectrum of **2** (400 MHz for ^{1}H NMR and 100 MHz for $^{13}C\{^{1}H\}$ NMR, acetone-d_6)

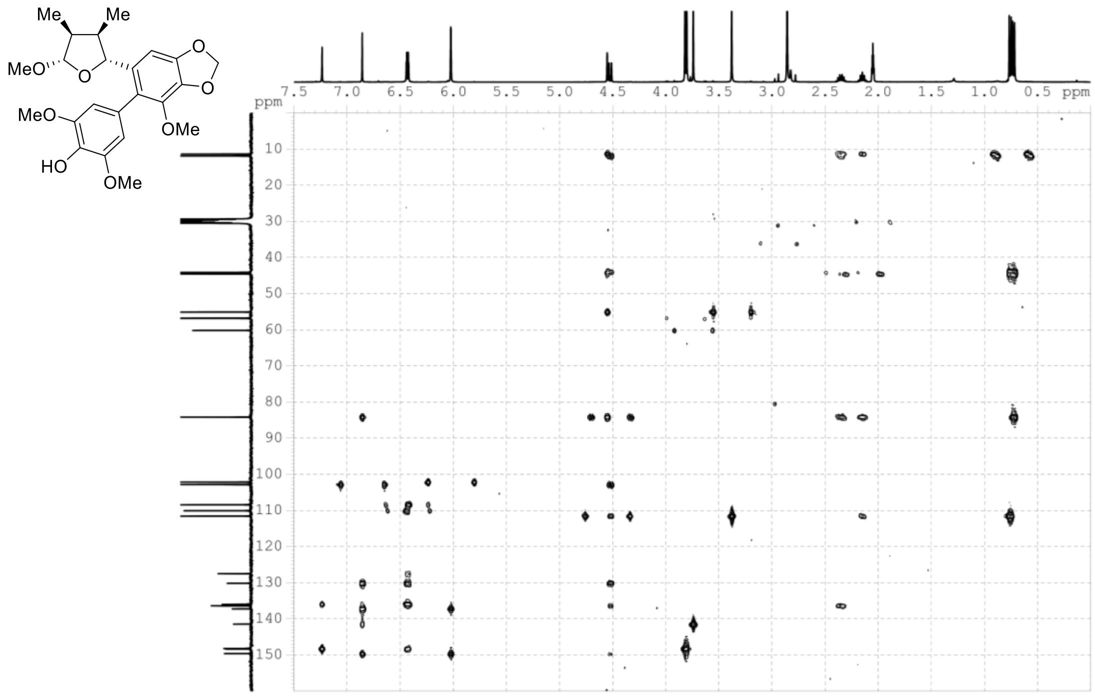

Key COSY correlations of **2** (400 MHz, acetone-d_6)

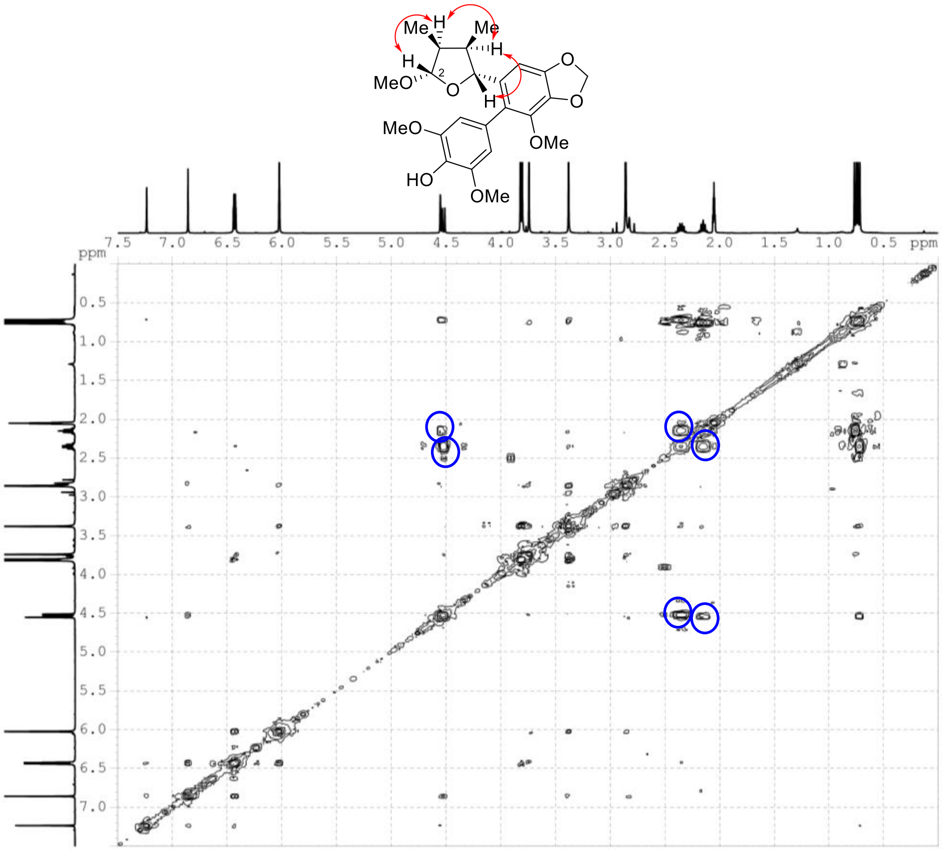

Key NOESY correlations of **2** (400 MHz, acetone-d_6)

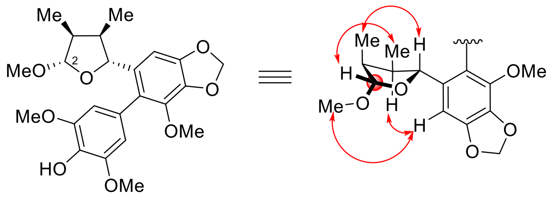

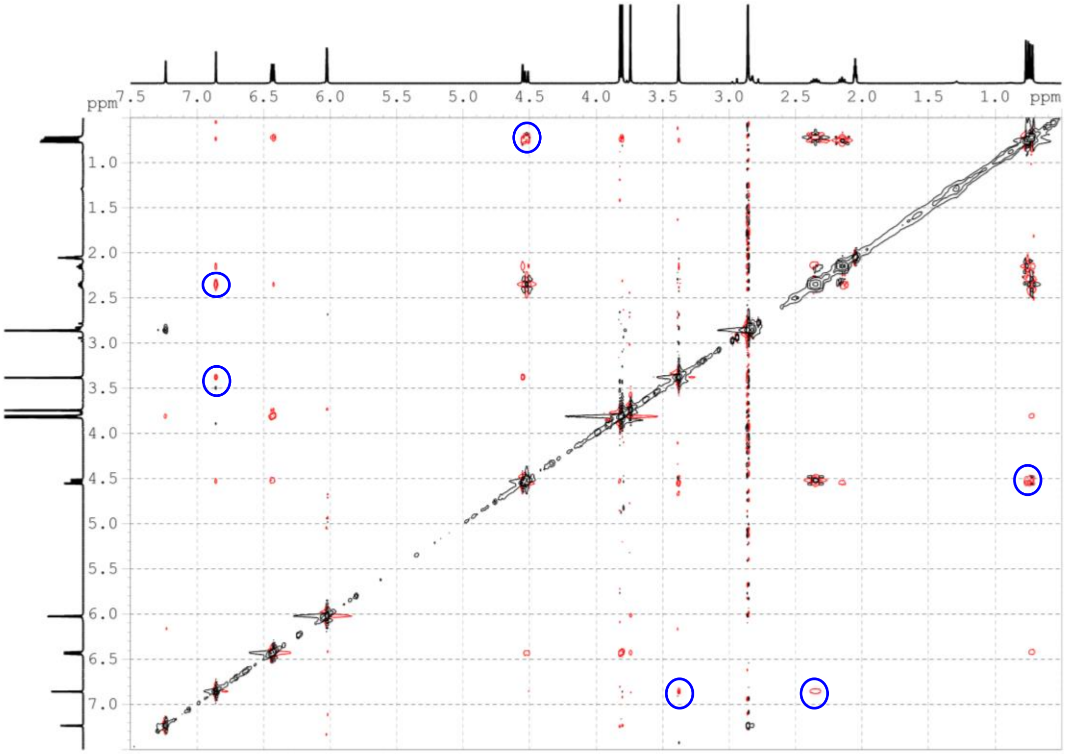

Optical rotation of **2**

Light Source	Na
Monitor wavelength	589 nm
D.I.T.	5 sec
No. of cycle	5
Cycle interval	1 sec
Temp. Monitor	Holder
Temp. Corr. Factor	0 at 24 C
Correct	Blank
Aperture(S)	8.0mm
Aperture(L)	Auto
Mode	Specific O.R.
Path Length	10 mm
Concentration	0.2 w/v%
Water content of sample	0 %
Factor	1

		Sample No.	Mode	Specific O.R.	Temperature(C)	Blank	Measurement Date	Comment
1	*	297F4-1	Specific O.R.	-0.2000	23.79	-0.0001	4/29/2025 11:53 AM	
2	*	297F4-2	Specific O.R.	-0.7000	23.79	-0.0001	4/29/2025 11:53 AM	
3	*	297F4-3	Specific O.R.	-4.7000	23.79	-0.0001	4/29/2025 11:53 AM	
4	*	297F4-4	Specific O.R.	-2.7000	23.79	-0.0001	4/29/2025 11:53 AM	
5	*	297F4-5	Specific O.R.	-1.2000	23.79	-0.0001	4/29/2025 11:53 AM	
6	*	Avg.		-1.9000				
7		S.D		1.8235				
8		C.V		95.9715				

^{1}H NMR spectrum of **15** (400 MHz, acetone-d_6)

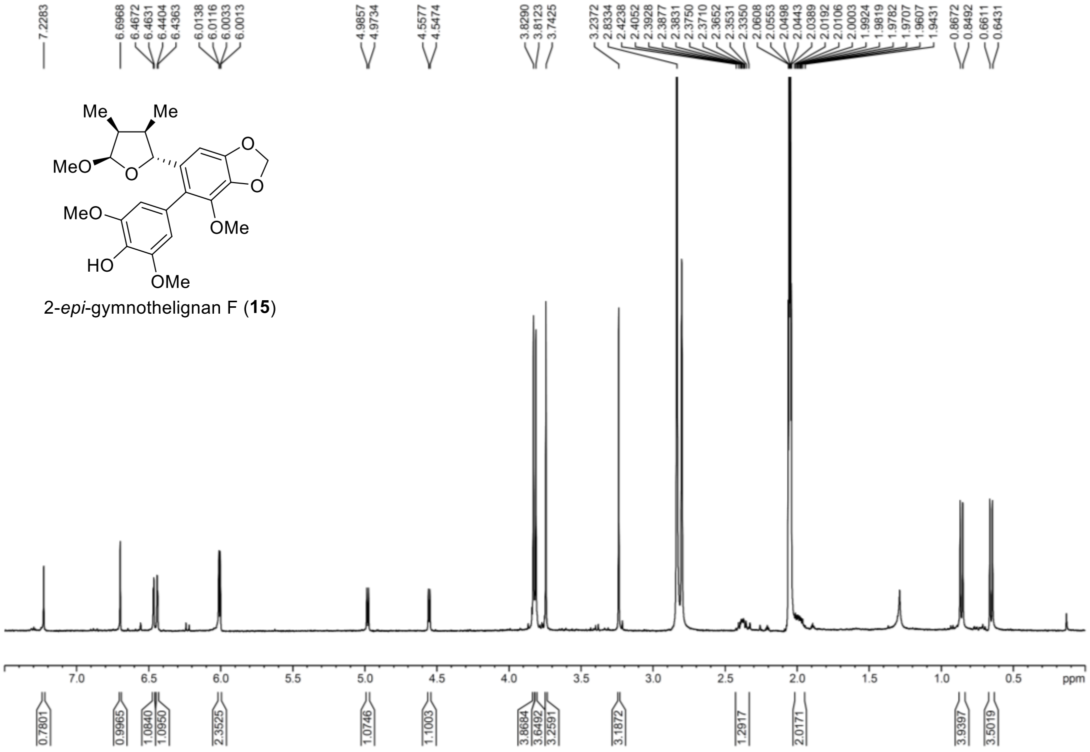

^{13}C{^{1}H} NMR spectrum of **15** (100 MHz, acetone-d_6)

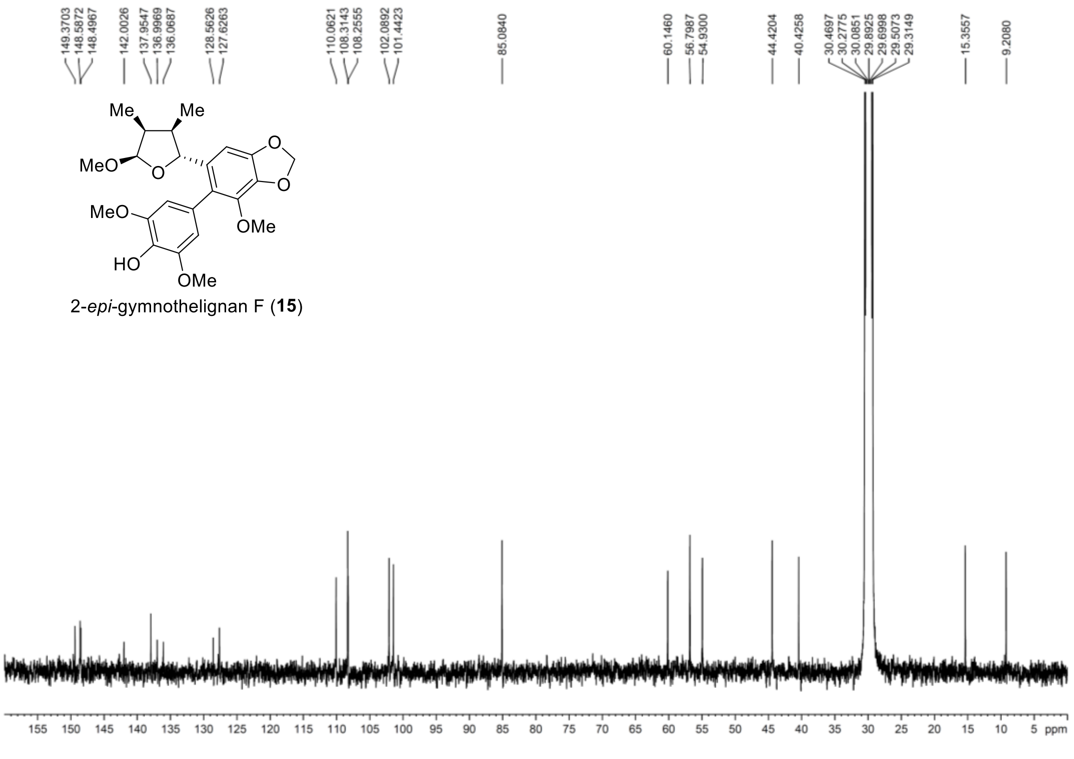

DEPT-135 NMR spectrum of **15** (100 MHz, acetone-d_6)

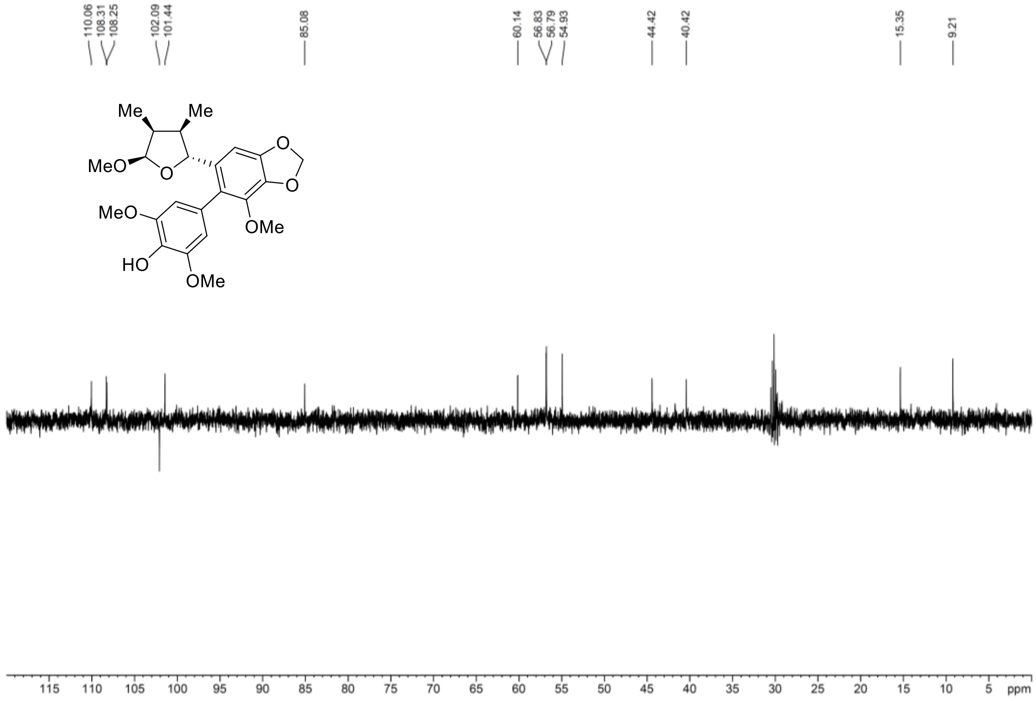

HSQC spectrum of **15** (400 MHz for ^1H NMR and 100 MHz for $^{13}C\{^1H\}$ NMR, acetone-d_6)

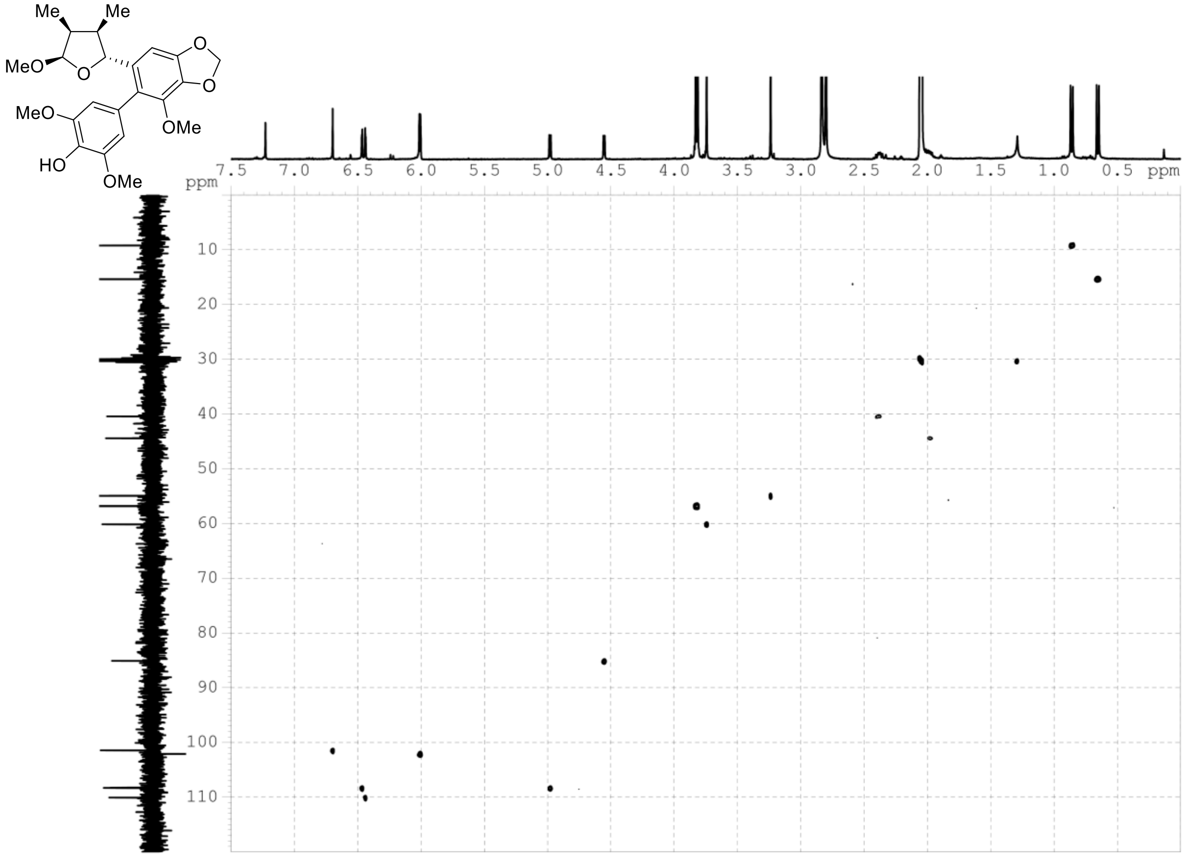

HMBC spectrum of **15** (400 MHz for ^{1}H NMR and 100 MHz for ^{13}C{^{1}H} NMR, acetone-d_6)

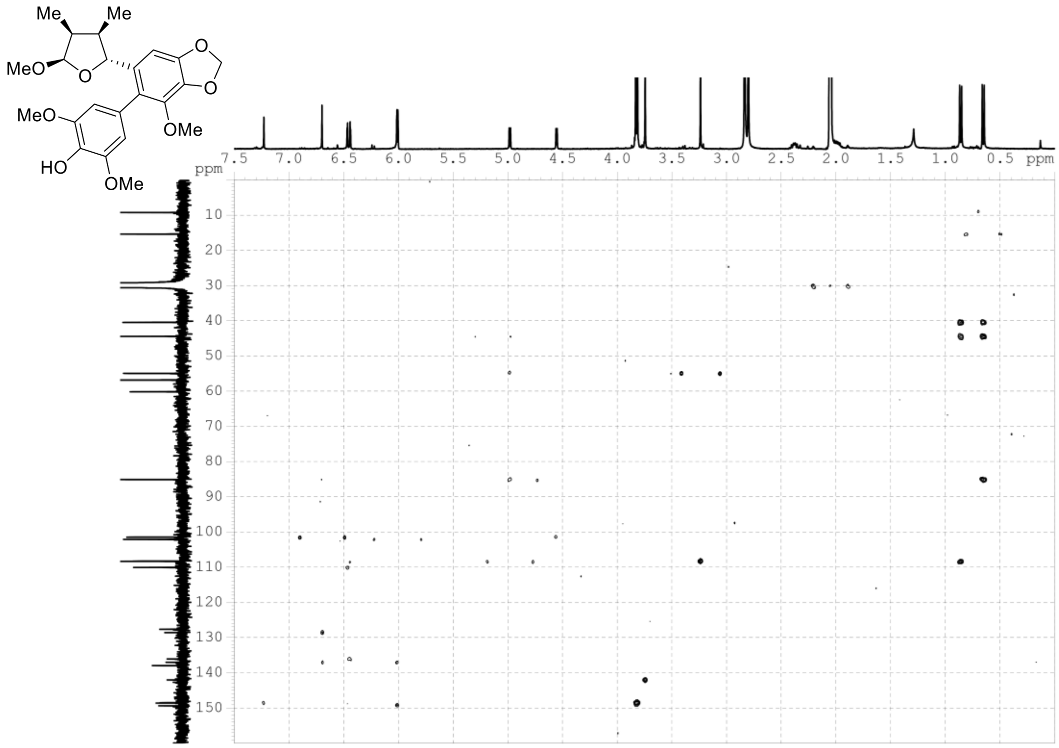

Key COSY correlations of **15** (400 MHz, acetone-d_6)

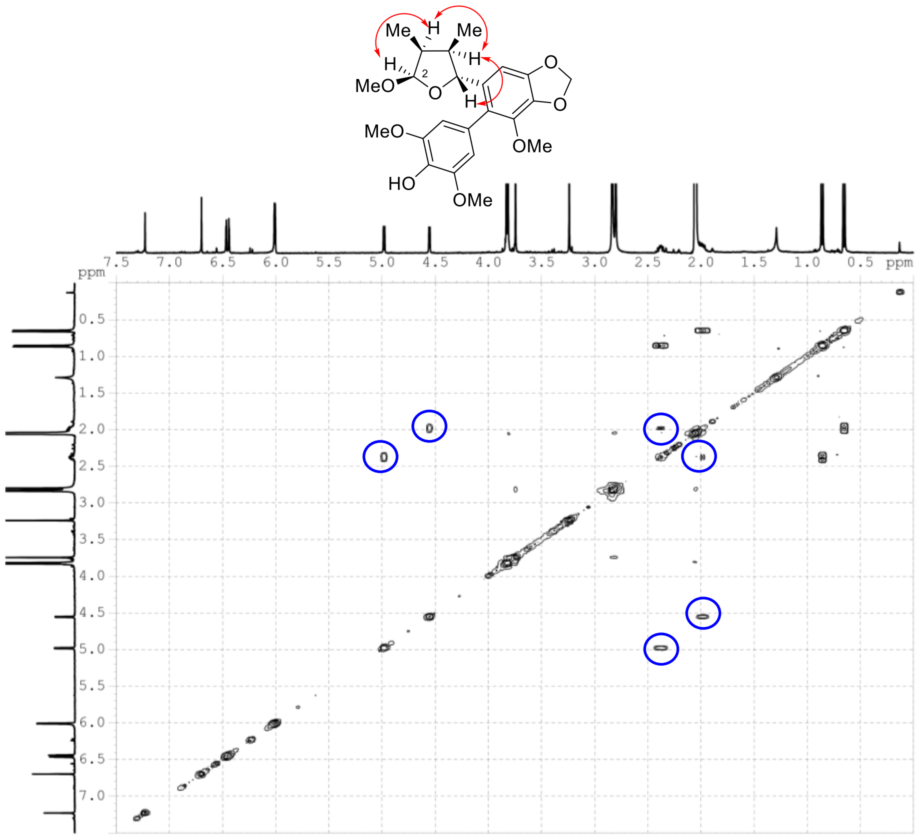

Key NOESY correlations of **15** (400 MHz, acetone-d_6)

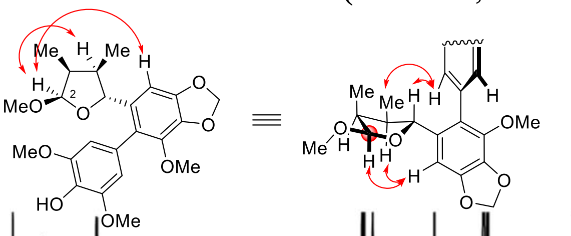

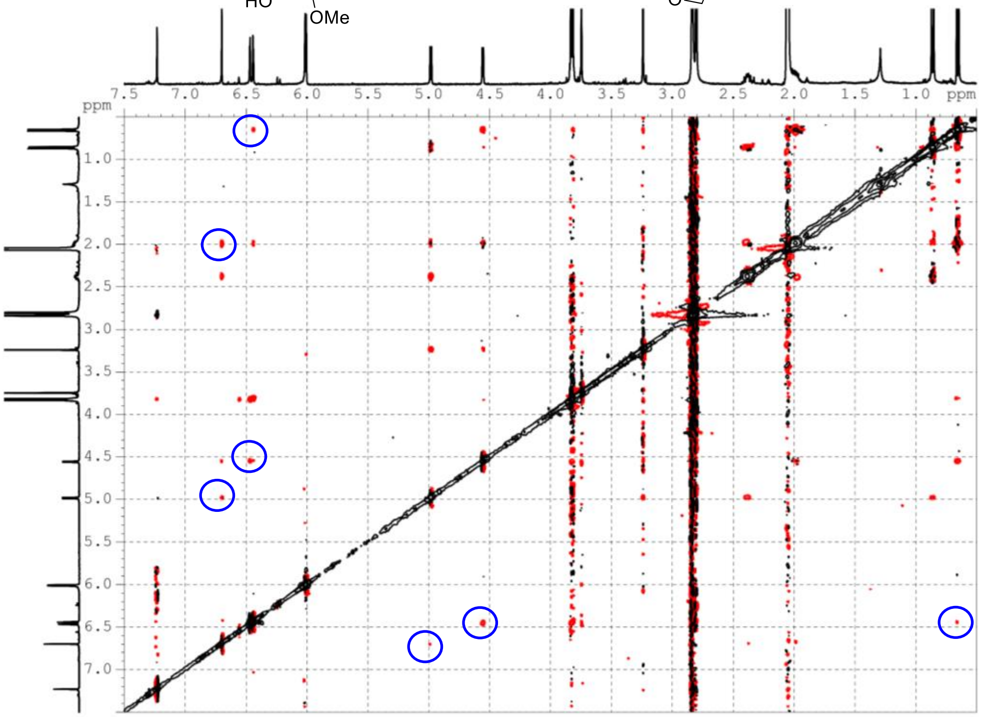

Optical rotation of **15**

Light Source	Na
Monitor wavelength	589 nm
D.I.T.	5 sec
No. of cycle	5
Cycle interval	1 sec
Temp. Monitor	Holder
Temp. Corr. Factor	0 at 25 C
Correct	Blank
Aperture(S)	8.0mm
Aperture(L)	Auto
Mode	Specific O.R.
Path Length	10 mm
Concentration	0.22 w/v%
Water content of sample	0 %
Factor	1

		Sample No.	Mode	Specific O.R.	Temperature(C)	Blank	Measurement Date	Comment
1	*	297F2-1	Specific O.R.	-46.0909	24.38	-0.0001	4/29/2025 10:59 AM	
2	*	297F2-2	Specific O.R.	-42.9091	24.38	-0.0001	4/29/2025 10:59 AM	
3	*	297F2-3	Specific O.R.	-45.6364	24.38	-0.0001	4/29/2025 10:59 AM	
4	*	297F2-4	Specific O.R.	-46.5455	24.38	-0.0001	4/29/2025 10:59 AM	
5	*	297F2-5	Specific O.R.	-44.7273	24.37	-0.0001	4/29/2025 10:59 AM	
6	*	Avg.		-45.1818				
7		S.D		1.4374				
8		C.V		3.1814				

^{1}H NMR spectrum of **17** (400 MHz, acetone-d_6)

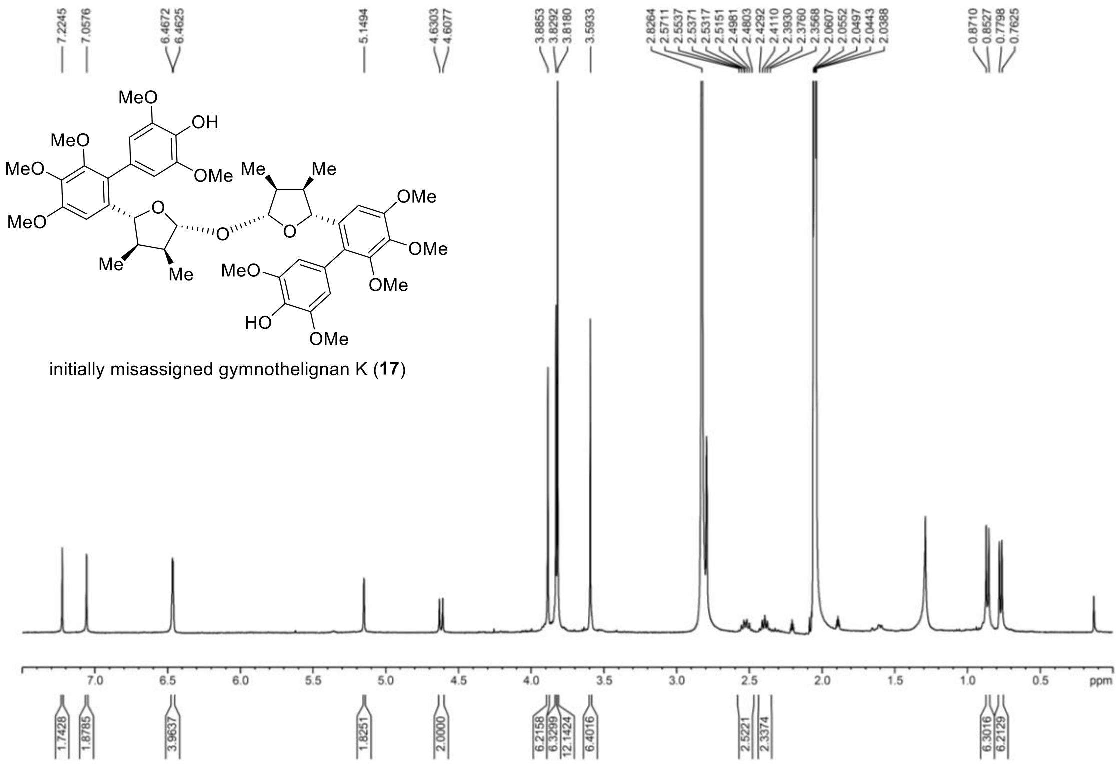

^{13}C{^{1}H} NMR spectrum of **17** (100 MHz, acetone-d_6)

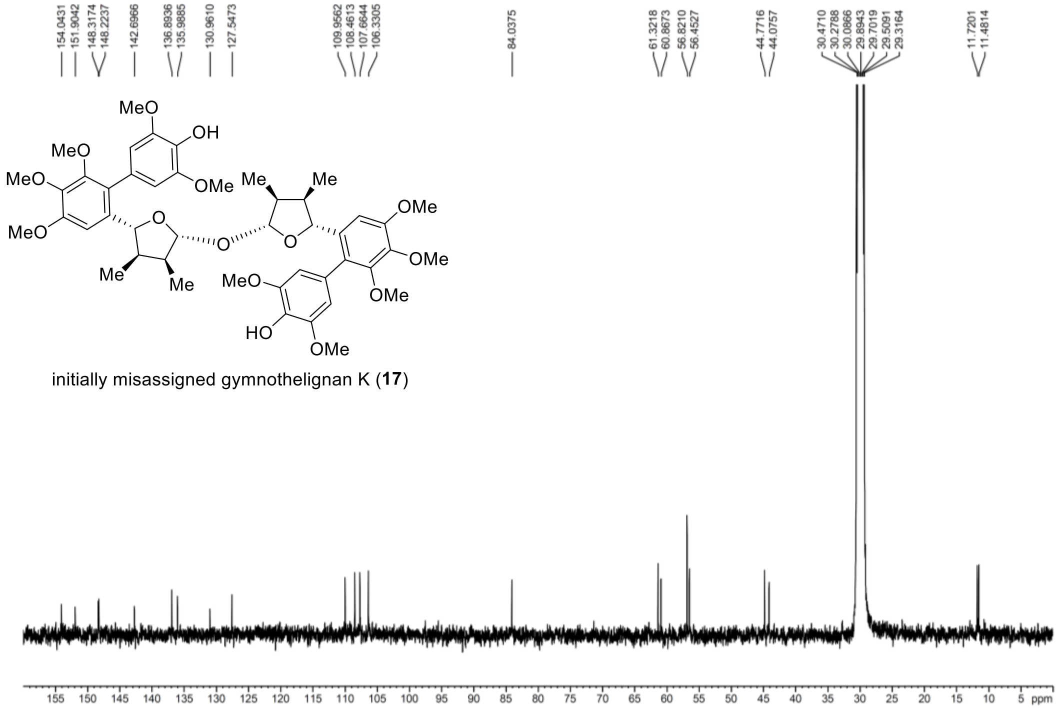

DEPT-135 NMR spectrum of **17** (100 MHz, acetone-d_6)

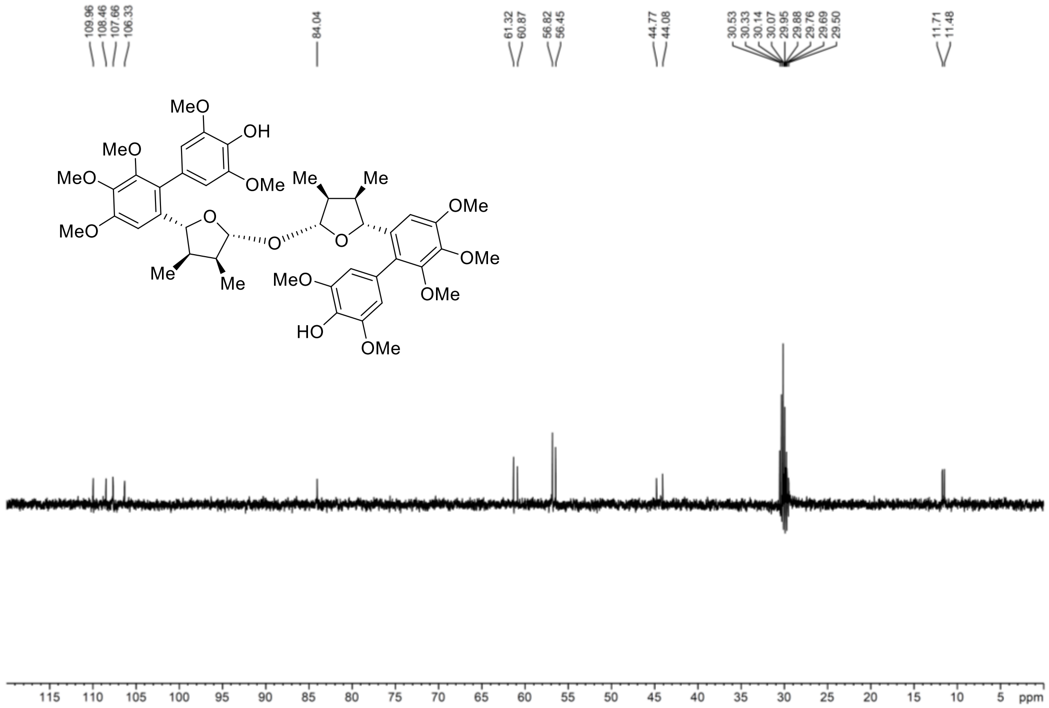

HSQC spectrum of **17** (400 MHz for ^{1}H NMR and 100 MHz for ^{13}C{^{1}H} NMR, acetone-d_6)

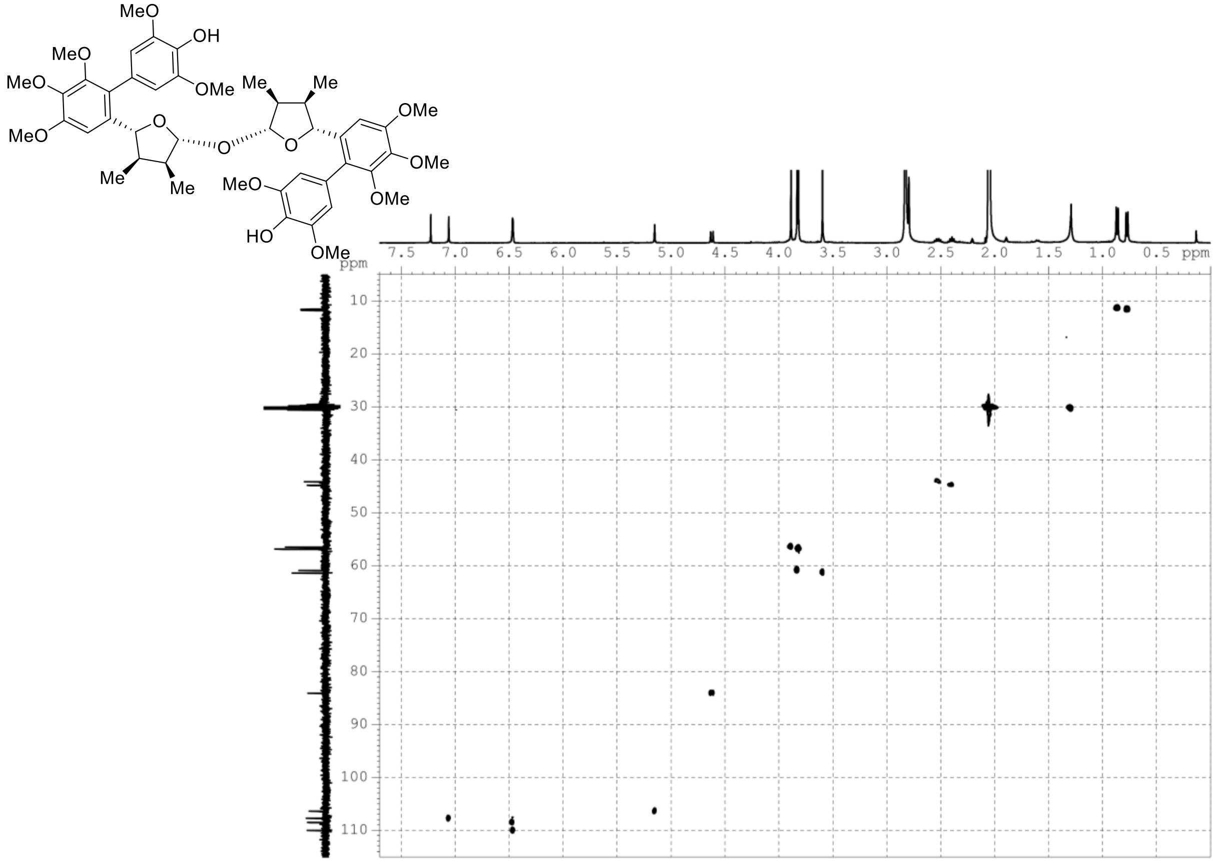

HMBC spectrum of **17** (400 MHz for ^{1}H NMR and 100 MHz for $^{13}C\{^{1}H\}$ NMR, acetone-d_6)

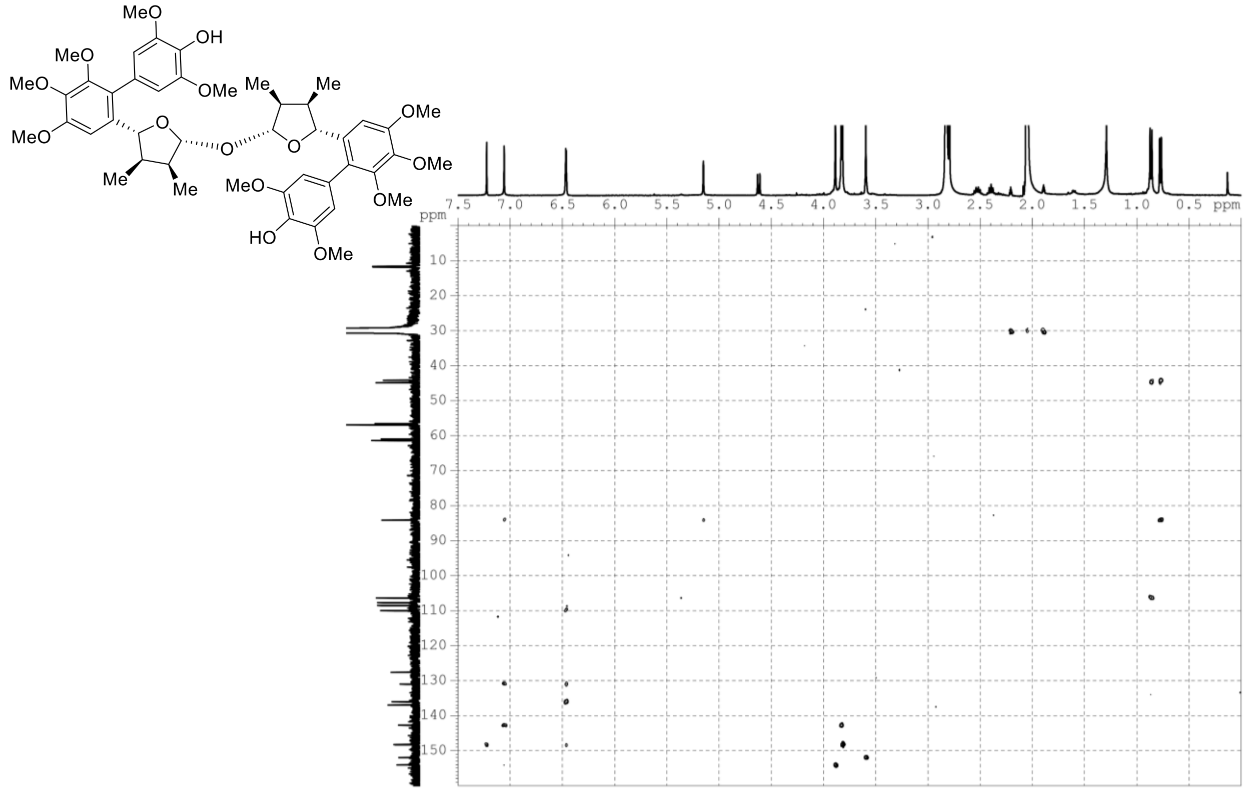

Key COSY correlations of **17** (400 MHz, acetone-d_6)

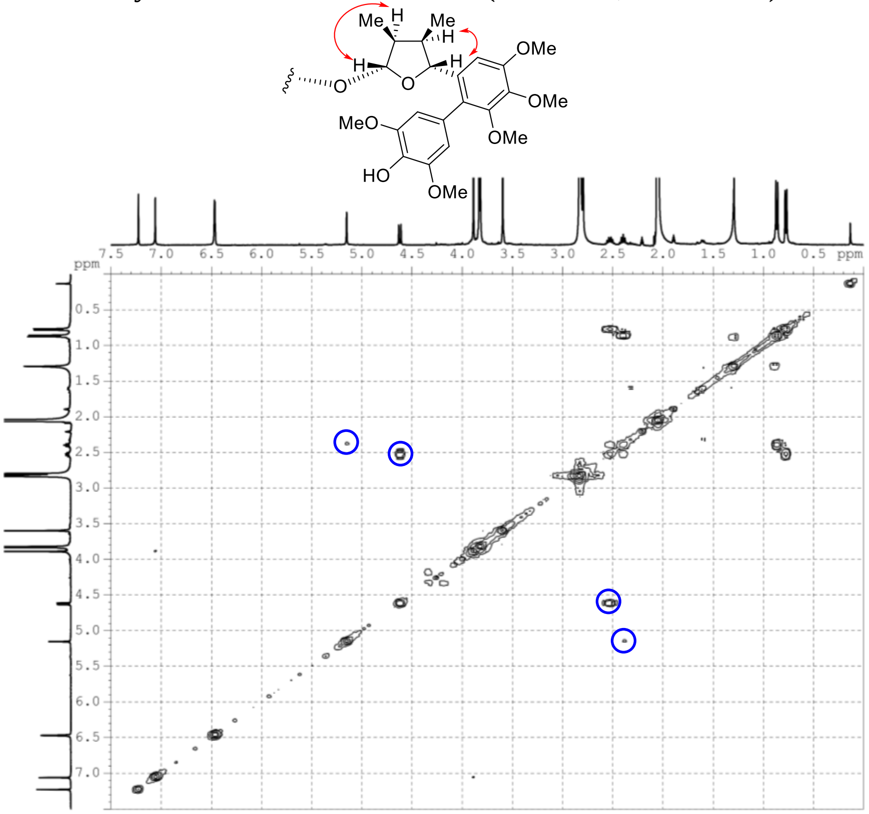

Key NOESY correlations of **17** (400 MHz, acetone-d_6)

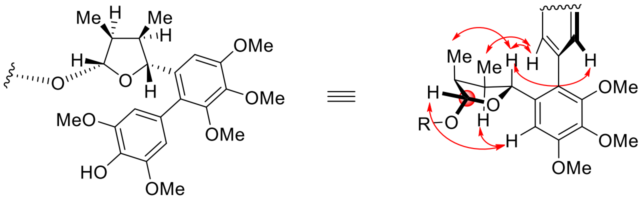

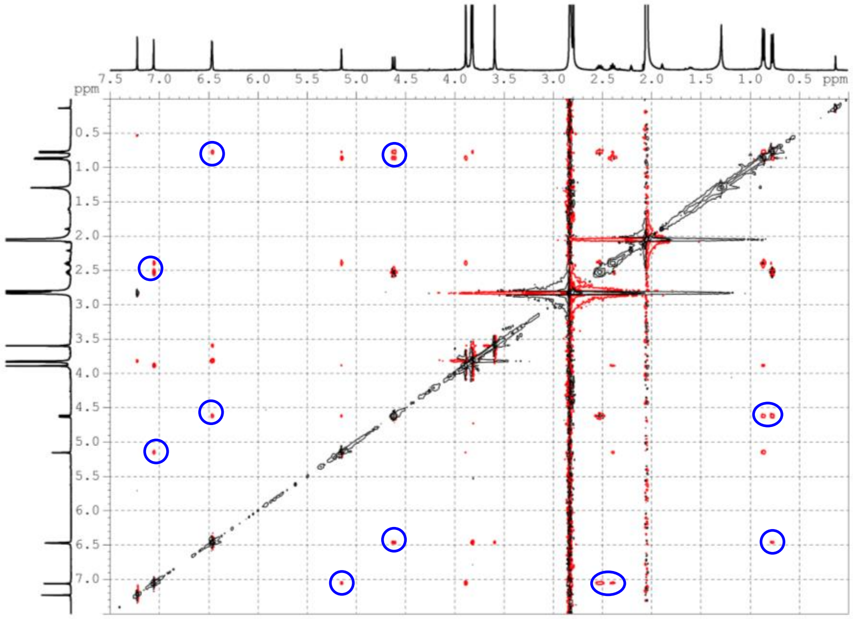

Optical rotation of **17**

Light Source	Na
Monitor wavelength	589 nm
D.I.T.	5 sec
No. of cycle	5
Cycle interval	1 sec
Temp. Monitor	Holder
Temp. Corr. Factor	0 at 25 C
Correct	Blank
Aperture(S)	8.0mm
Aperture(L)	Auto
Mode	Specific O.R.
Path Length	10 mm
Concentration	0.14 w/v%
Water content of sample	0 %
Factor	1

		Sample No.	Mode	Specific O.R.	Temperature(C)	Blank	Measurement Date	Comment
1	*	420P2-1	Specific O.R.	33.2857	23.94	-0.0001	11/7/2025 11:25 AM	
2	*	420P2-2	Specific O.R.	33.2857	23.94	-0.0001	11/7/2025 11:25 AM	
3	*	420P2-3	Specific O.R.	33.2857	23.93	-0.0001	11/7/2025 11:25 AM	
4	*	420P2-4	Specific O.R.	33.2857	23.93	-0.0001	11/7/2025 11:25 AM	
5	*	420P2-5	Specific O.R.	33.2857	23.92	-0.0001	11/7/2025 11:25 AM	
6	*	Avg.		33.2857				
7		S.D		0.0000				
8		C.V		0.0000				

^{1}H NMR spectrum of **3** (400 MHz, acetone-d_6)

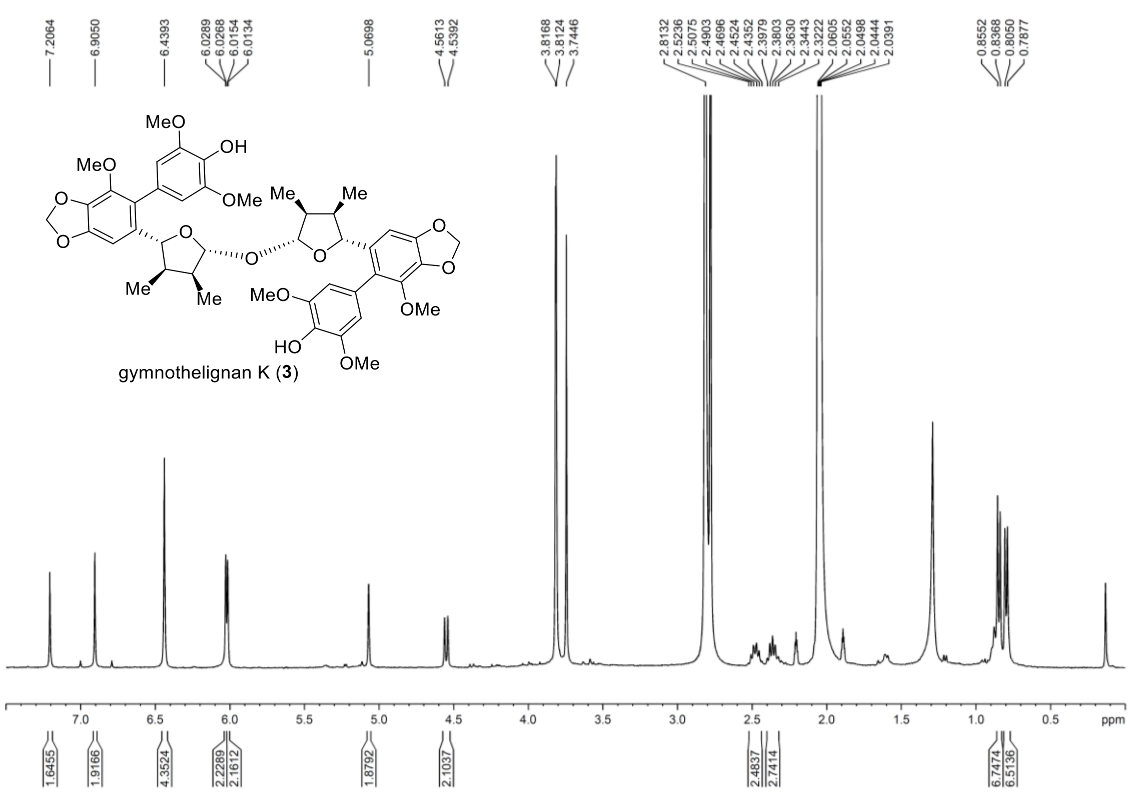

^{13}C{^{1}H} NMR spectrum of **3** (100 MHz, acetone-d_6)

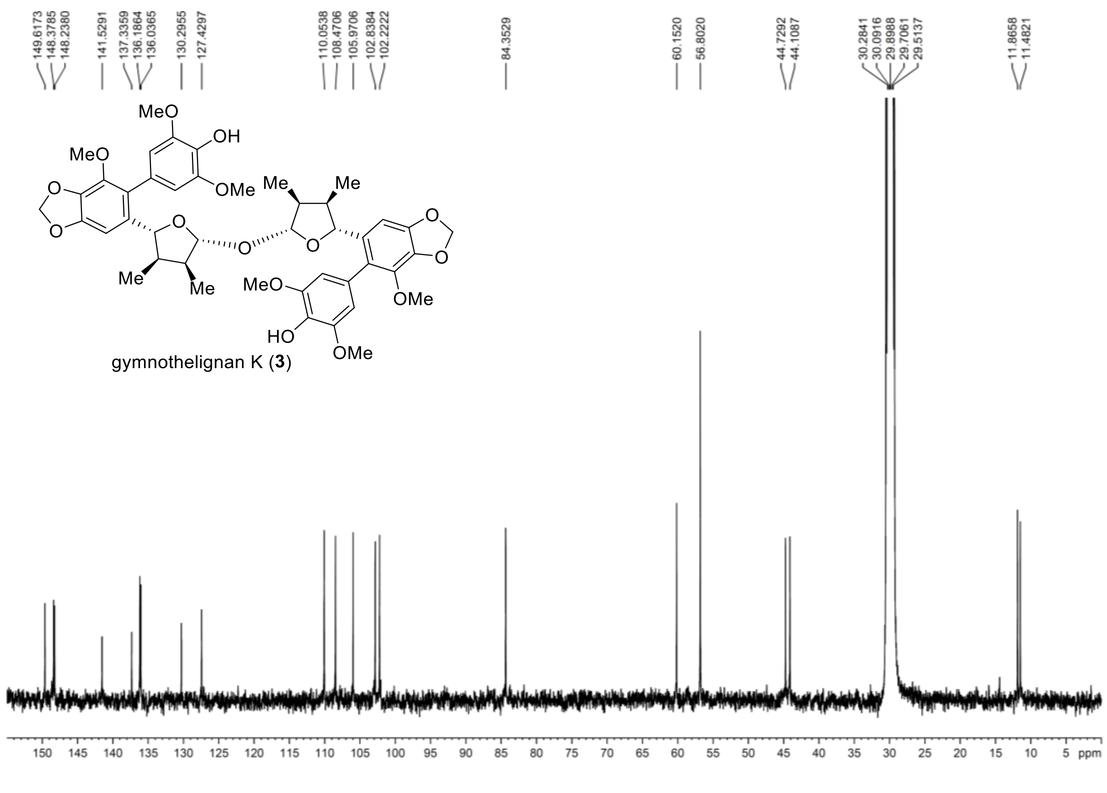

DEPT-135 NMR spectrum of **3** (100 MHz, acetone-d_6)

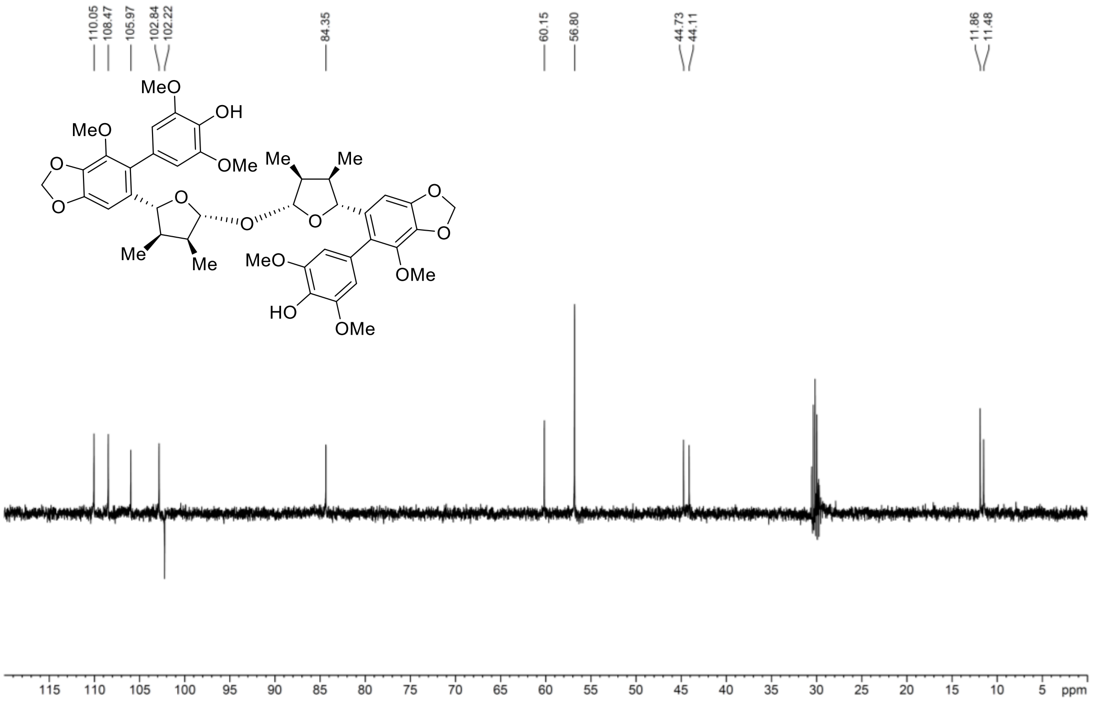

HSQC spectrum of **3** (400 MHz for ^{1}H NMR and 100 MHz for ^{13}C{^{1}H} NMR, acetone-d_6)

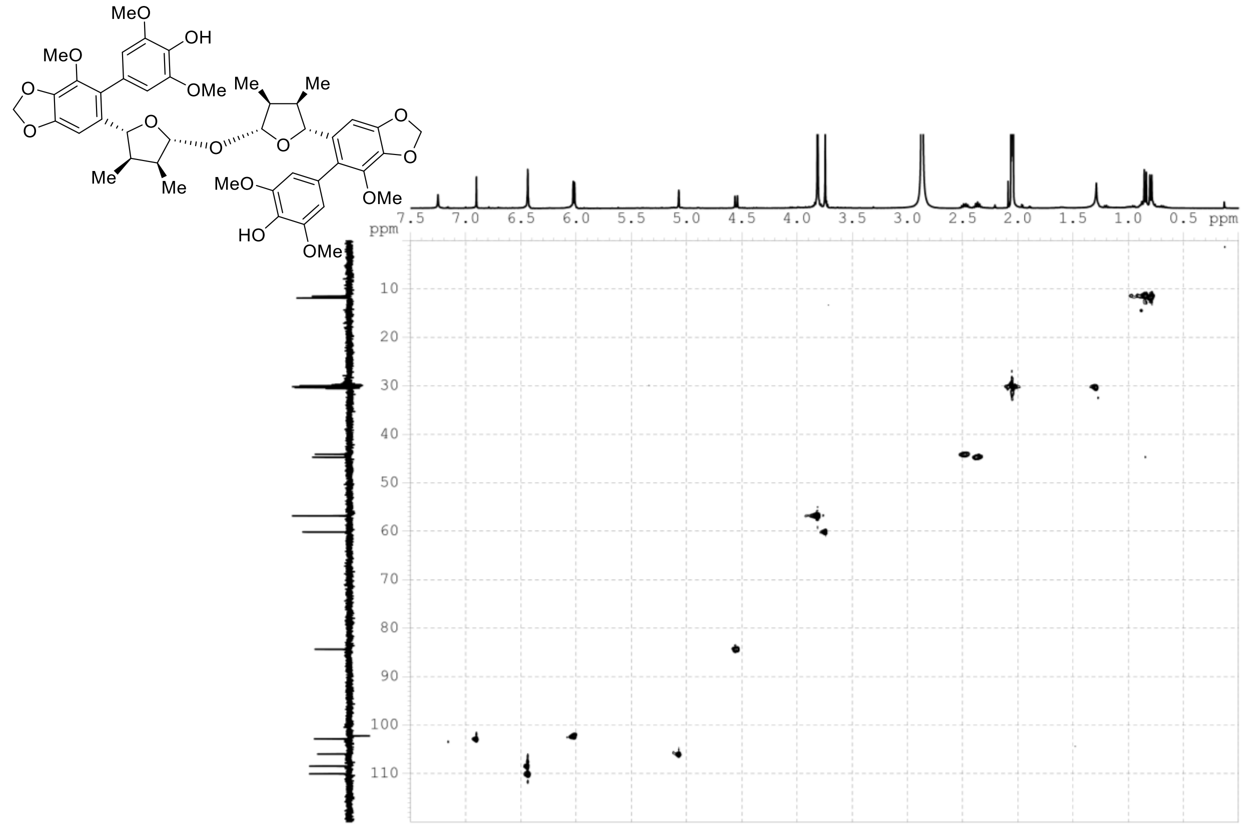

HMBC spectrum of **3** (400 MHz for ^{1}H NMR and 100 MHz for $^{13}C\{^{1}H\}$ NMR, acetone-d_6)

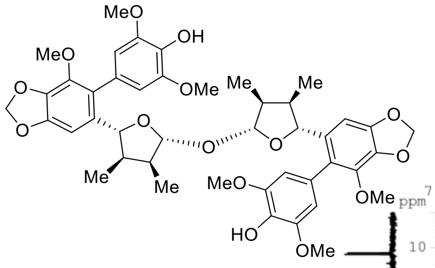

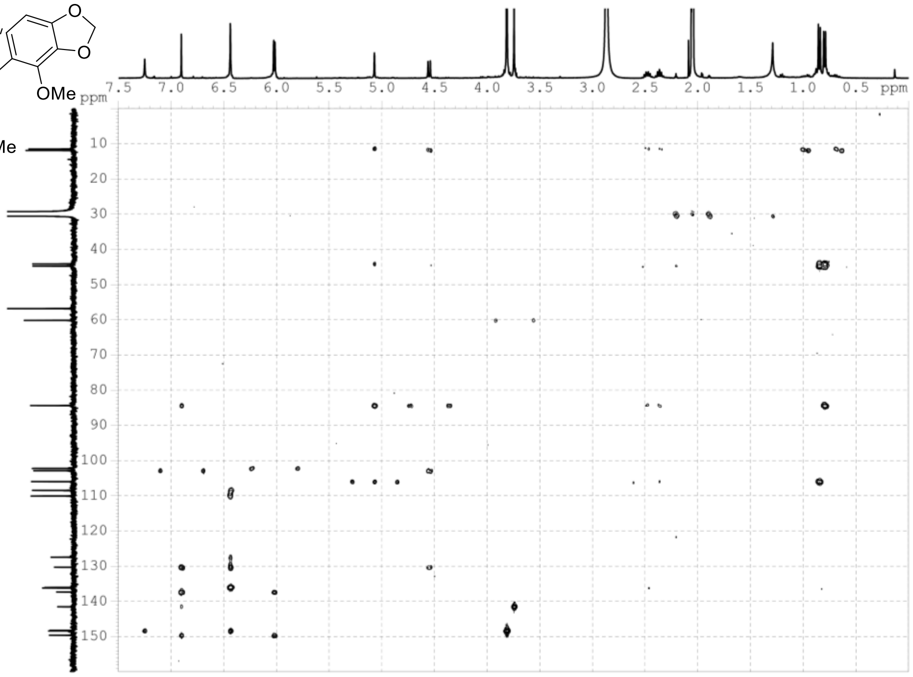

Key COSY correlations of **3** (400 MHz, acetone-d_6)

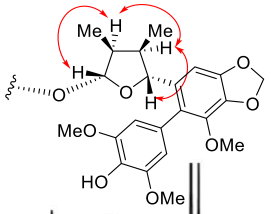

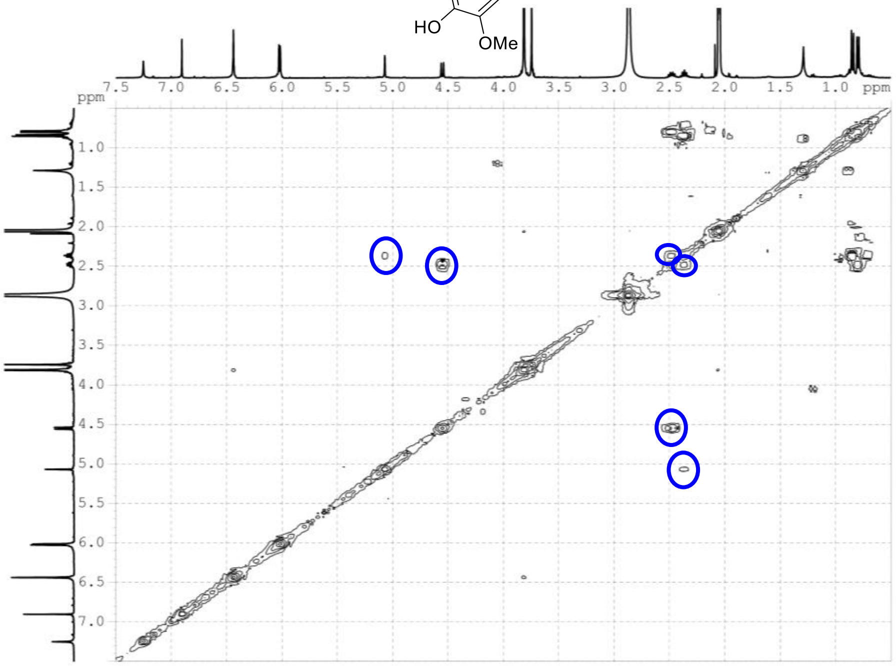

Key NOESY correlations of **3** (400 MHz, acetone-d_6)

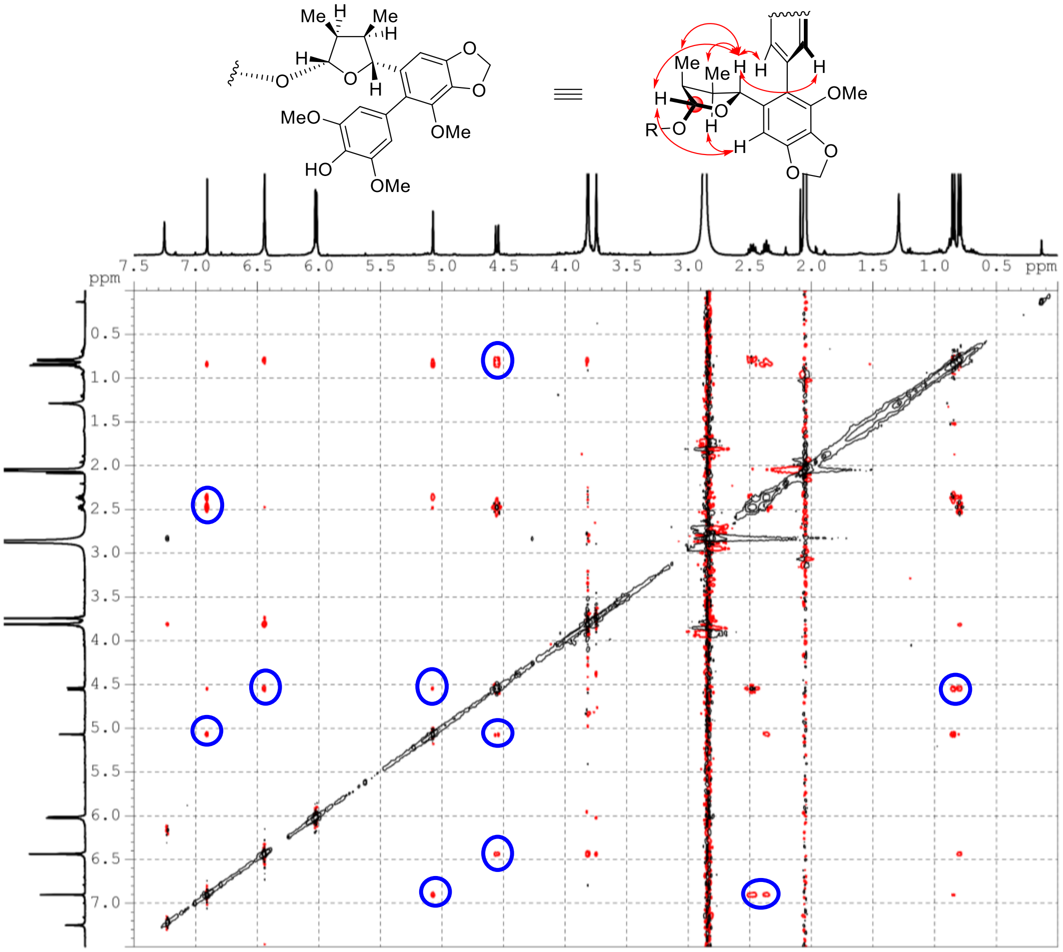

Optical rotation of **3**

Light Source	Na
Monitor wavelength	589 nm
D.I.T.	5 sec
No. of cycle	5
Cycle interval	1 sec
Temp. Monitor	Holder
Temp. Corr. Factor	0 at 25 C
Correct	Blank
Aperture(S)	8.0mm
Aperture(L)	Auto
Mode	Specific O.R.
Path Length	10 mm
Concentration	0.09 w/v%
Water content of sample	0 %
Factor	1

		Sample No.	Mode	Specific O.R.	Temperature(C)	Blank	Measurement Date	Comment
1	*	415-C1-1	Specific O.R.	-10.6667	21.16	0.0007	3/6/2026 2:24 PM	
2	*	415-C1-2	Specific O.R.	-9.5556	21.17	0.0007	3/6/2026 2:25 PM	
3	*	415-C1-3	Specific O.R.	-8.4444	21.17	0.0007	3/6/2026 2:25 PM	
4	*	415-C1-4	Specific O.R.	-12.8889	21.17	0.0007	3/6/2026 2:25 PM	
5	*	415-C1-5	Specific O.R.	-12.8889	21.16	0.0007	3/6/2026 2:25 PM	
6	*	Avg.		-10.8889				
7		S.D		1.9876				
8		C.V		18.2536				

^{1}H NMR spectrum of **4** (400 MHz, acetone-d_6)

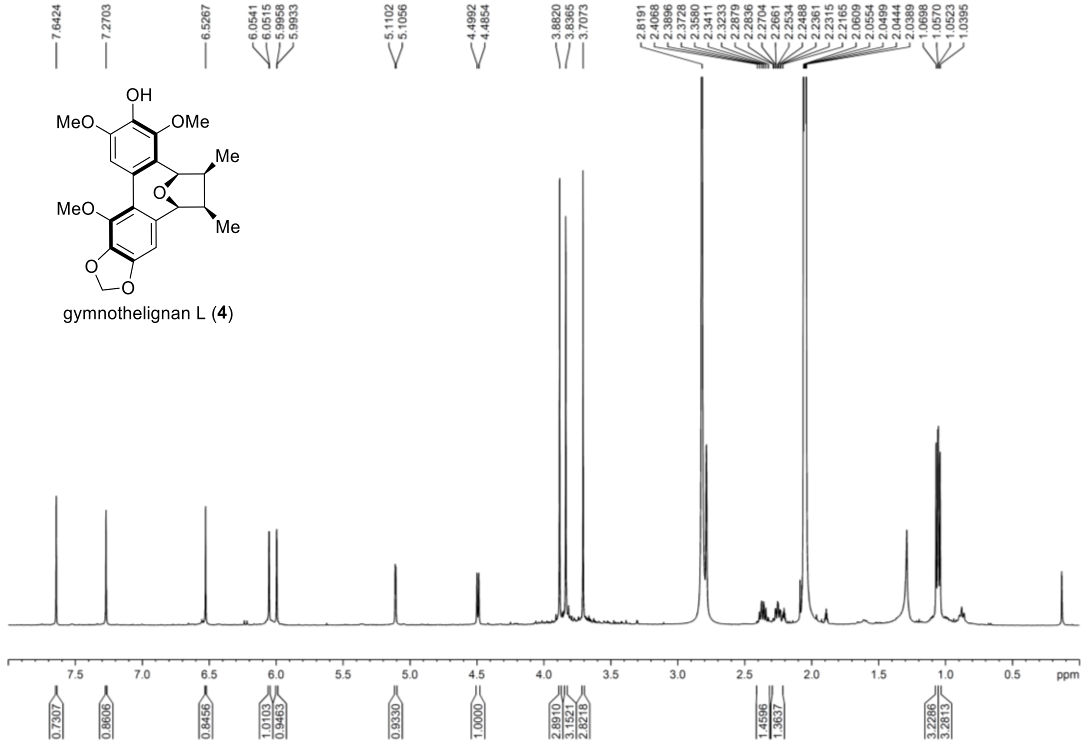

^{13}C{^{1}H} NMR spectrum of **4** (100 MHz, acetone-d_6)

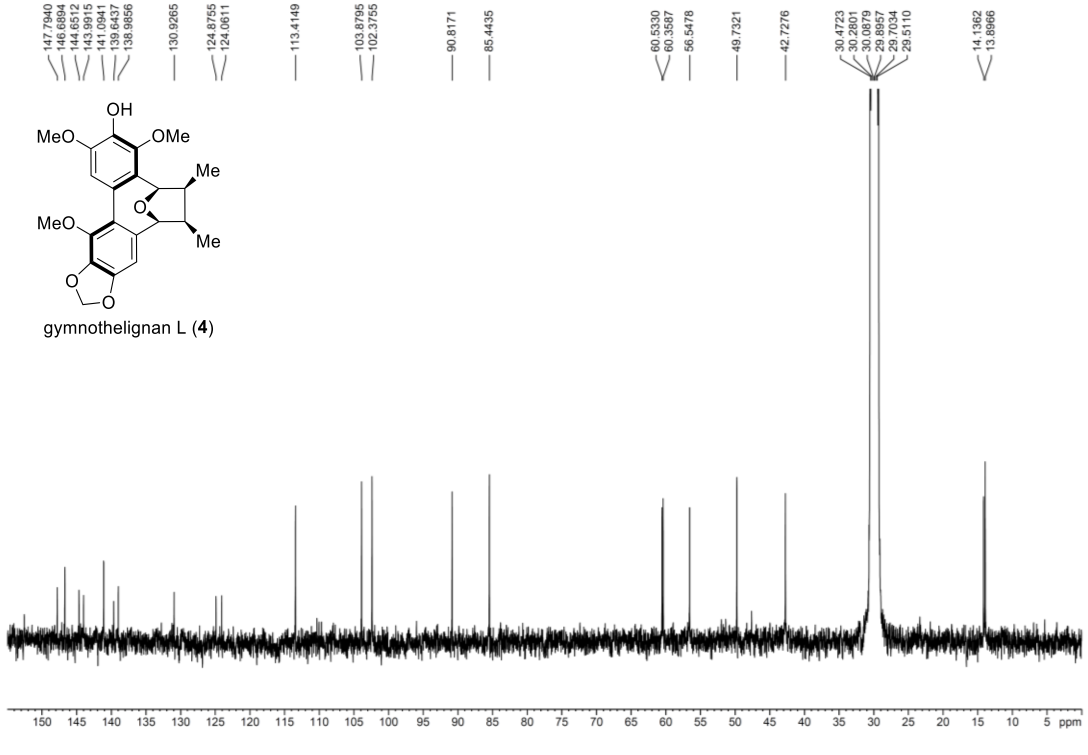

DEPT-135 NMR spectrum of **4** (100 MHz, acetone-d_6)

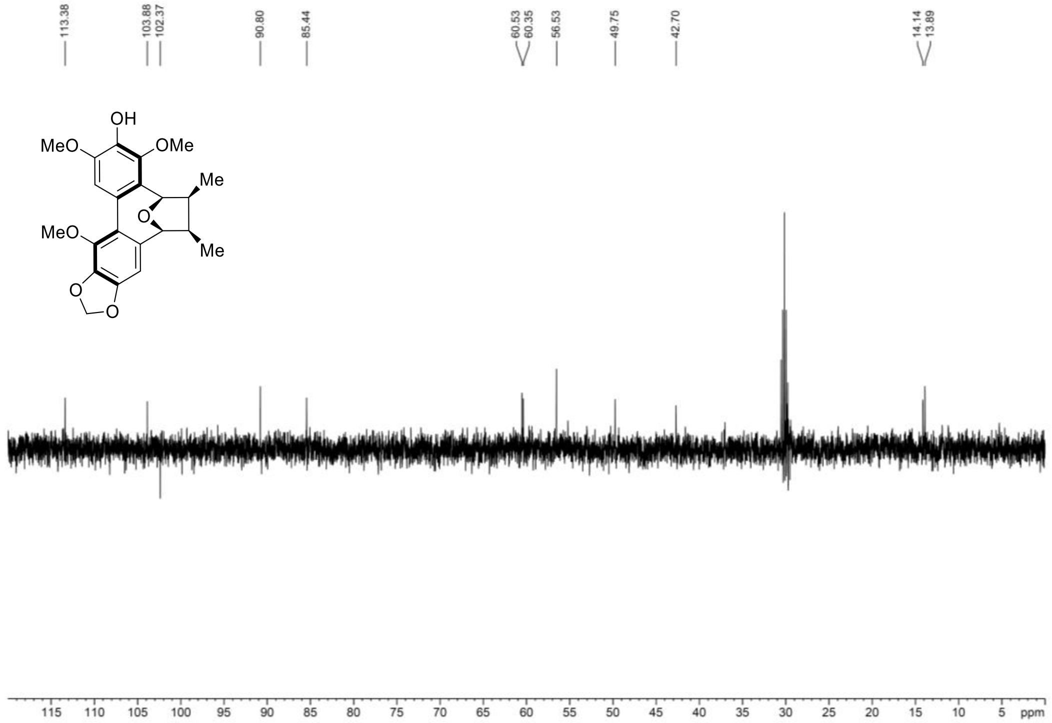

HSQC spectrum of **4** (400 MHz for ^{1}H NMR and 100 MHz for ^{13}C{^{1}H} NMR, acetone-d_6)

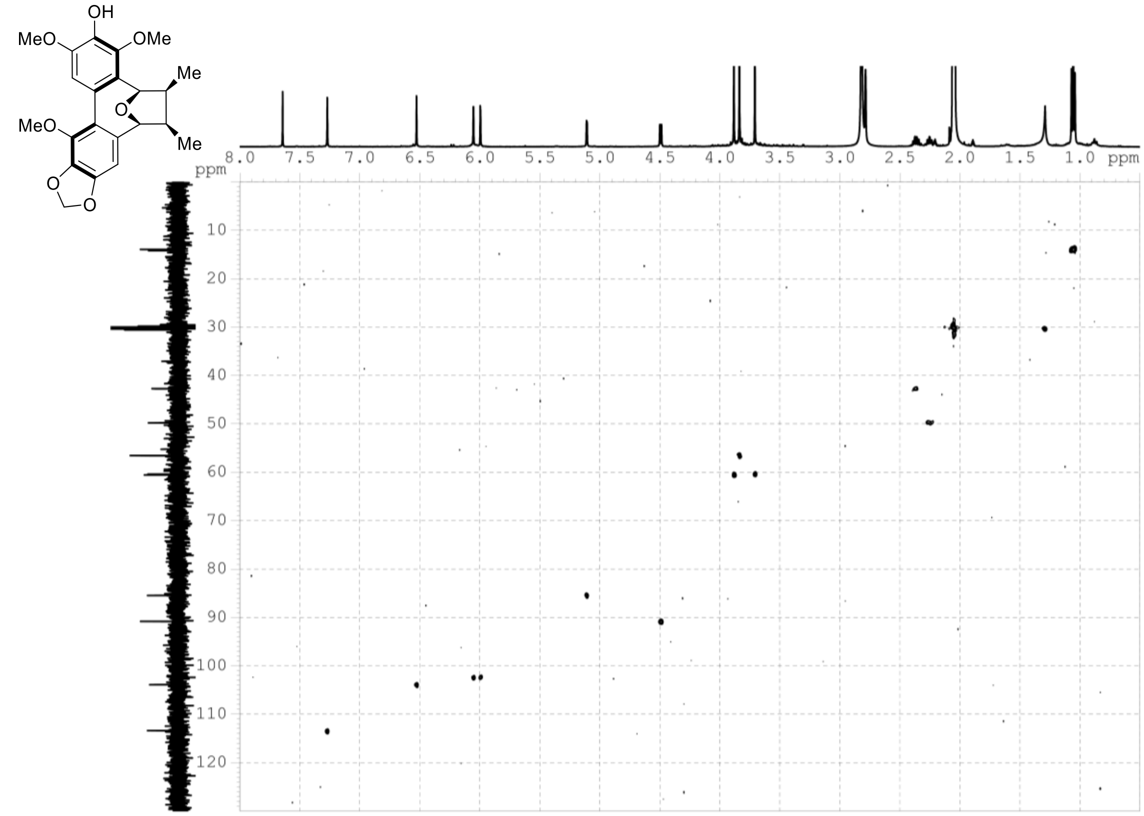

HMBC spectrum of **4** (400 MHz for ^{1}H NMR and 100 MHz for ^{13}C{^{1}H} NMR, acetone-d_6)

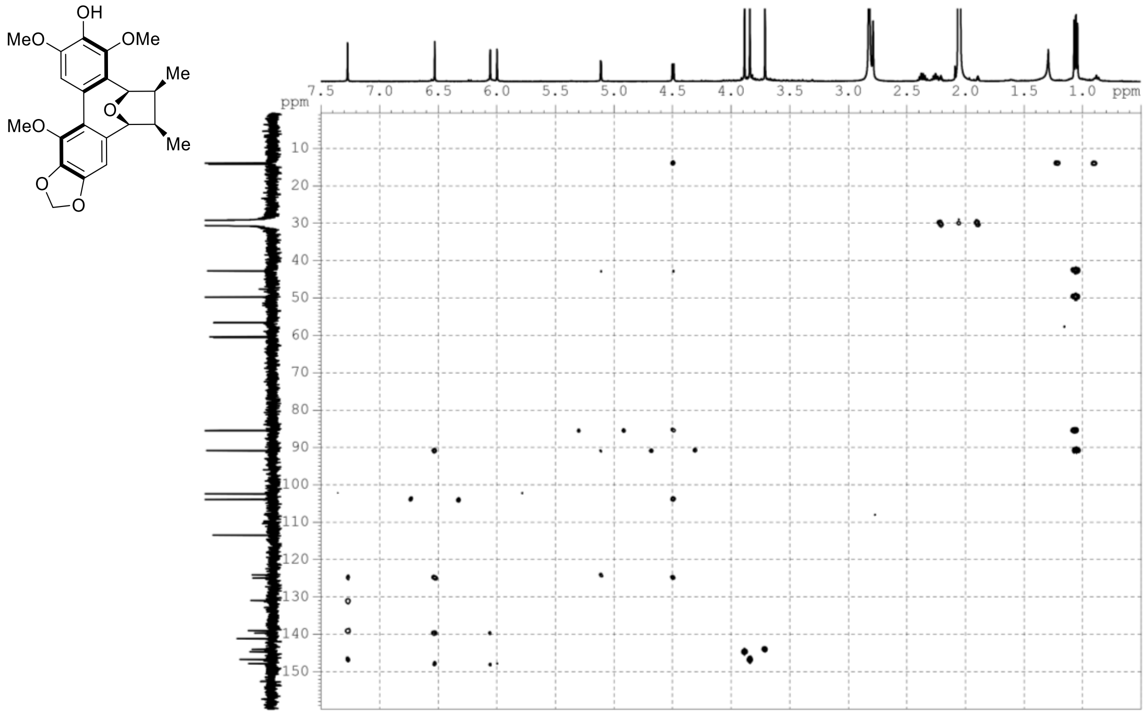

Key COSY correlations of **4** (400 MHz, acetone-d_6)

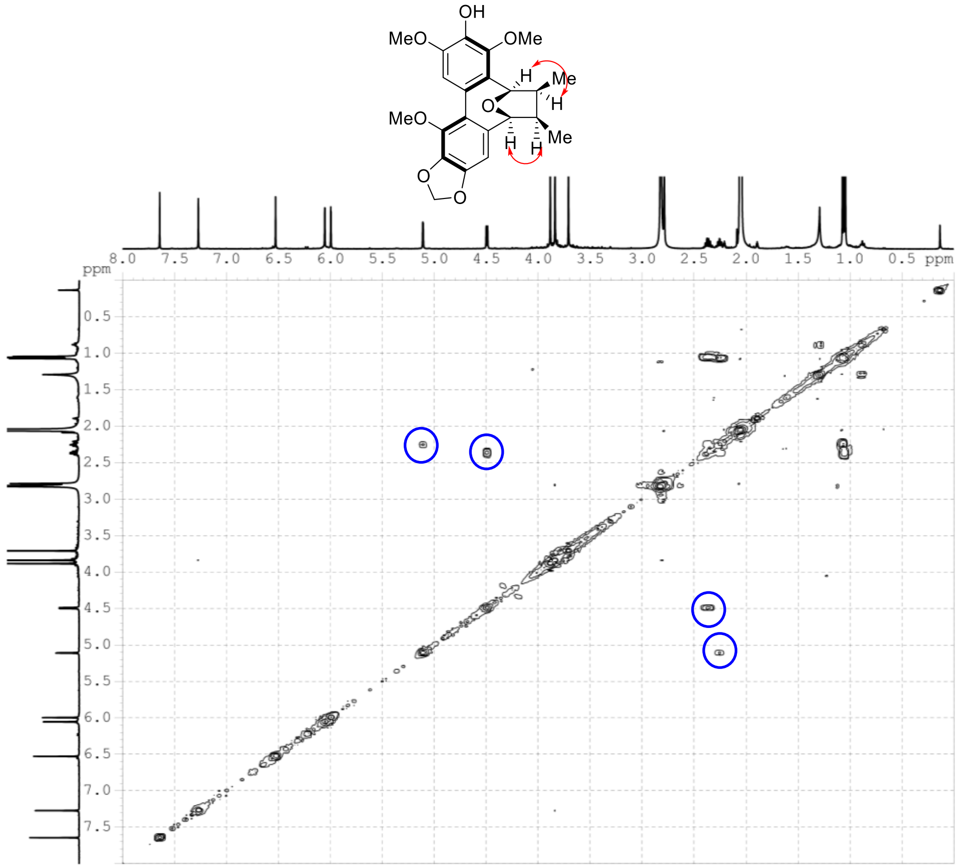

Key NOESY correlations of **4** (400 MHz, acetone-d_6)

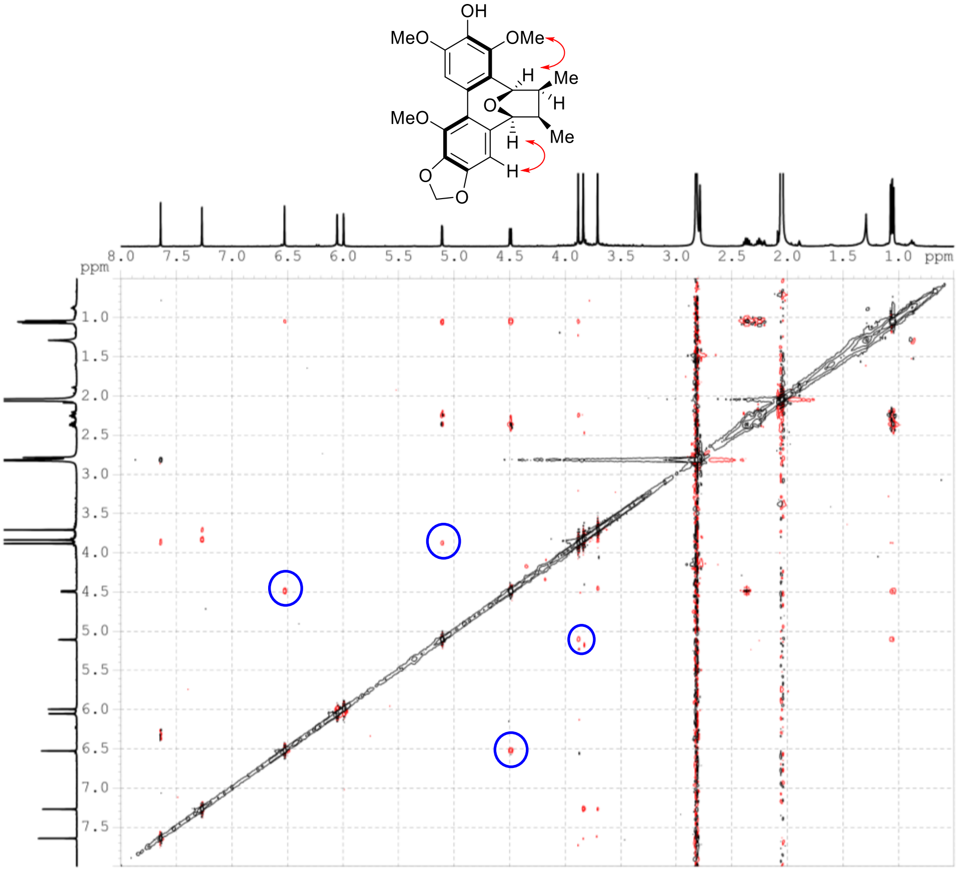

Optical rotation of **4**

Light Source	Na
Monitor wavelength	589 nm
D.I.T.	5 sec
No. of cycle	5
Cycle interval	1 sec
Temp. Monitor	Holder
Temp. Corr. Factor	0 at 25 C
Correct	Blank
Aperture(S)	8.0mm
Aperture(L)	Auto
Mode	Specific O.R.
Path Length	10 mm
Concentration	0.2 w/v%
Water content of sample	0 %
Factor	1

		Sample No.	Mode	Specific O.R.	Temperature(C)	Blank	Measurement Date	Comment
1	*	354F1-1	Specific O.R.	-1.9000	22.76	0.0006	8/1/2025 11:30 AM	
2	*	354F1-2	Specific O.R.	-1.9000	22.76	0.0006	8/1/2025 11:30 AM	
3	*	354F1-3	Specific O.R.	-1.9000	22.76	0.0006	8/1/2025 11:30 AM	
4	*	354F1-4	Specific O.R.	-1.9000	22.76	0.0006	8/1/2025 11:30 AM	
5	*	354F1-5	Specific O.R.	-1.9000	22.75	0.0006	8/1/2025 11:30 AM	
6	*	Avg.		-1.9000				
7		S.D		0.0000				
8		C.V		0.0000				

^{1}H NMR spectrum of **20** (400 MHz, acetone-d_6)

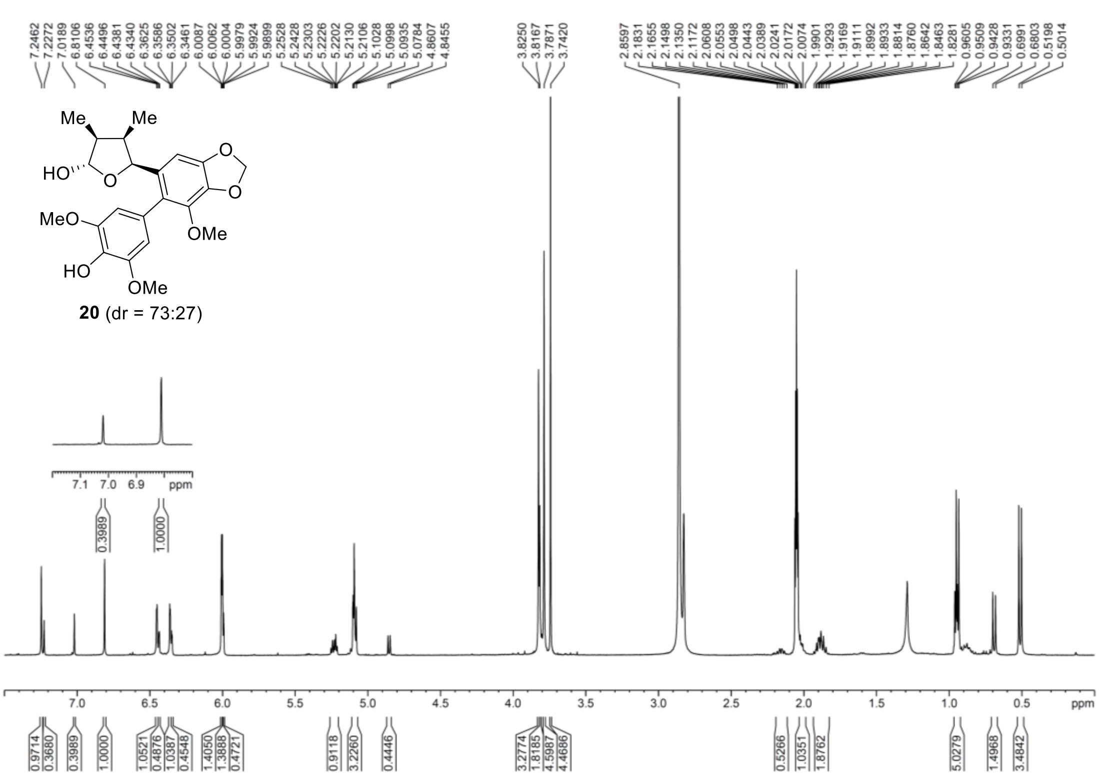

^{13}C{^{1}H} NMR spectrum of **20** (100 MHz, acetone-d_6)

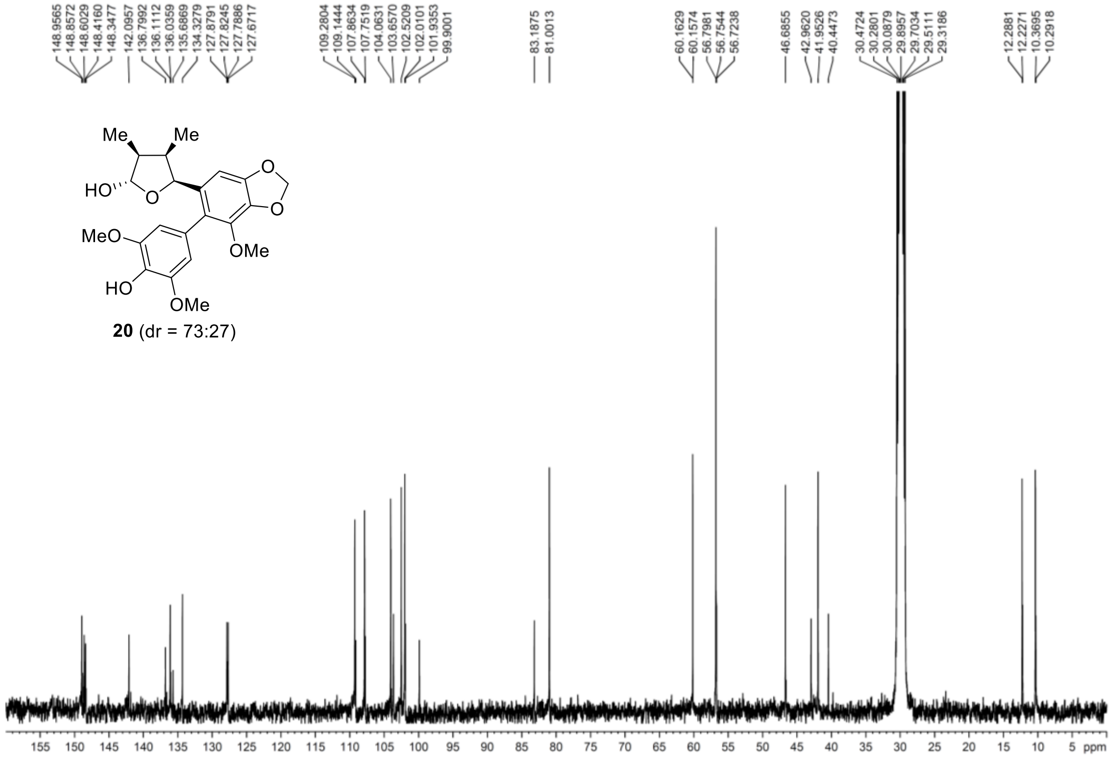

DEPT-135 NMR spectrum of **20** (100 MHz, acetone-d_6)

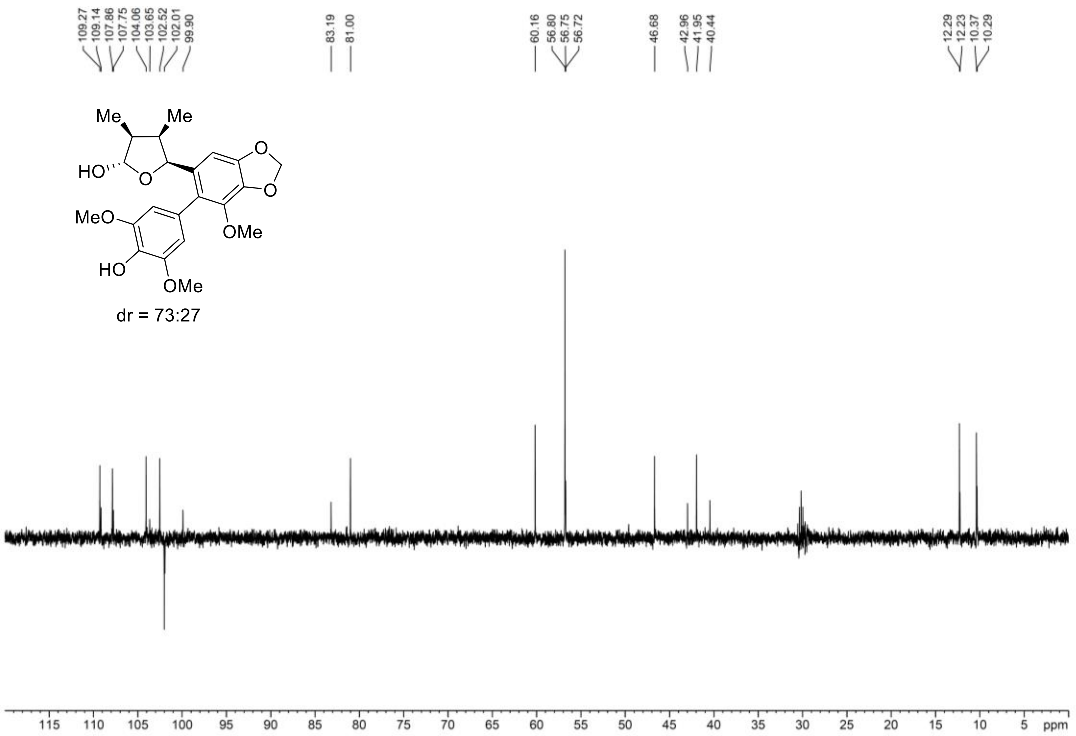

HSQC spectrum of **20** (400 MHz for ^{1}H NMR and 100 MHz for ^{13}C{^{1}H} NMR, acetone-d_6)

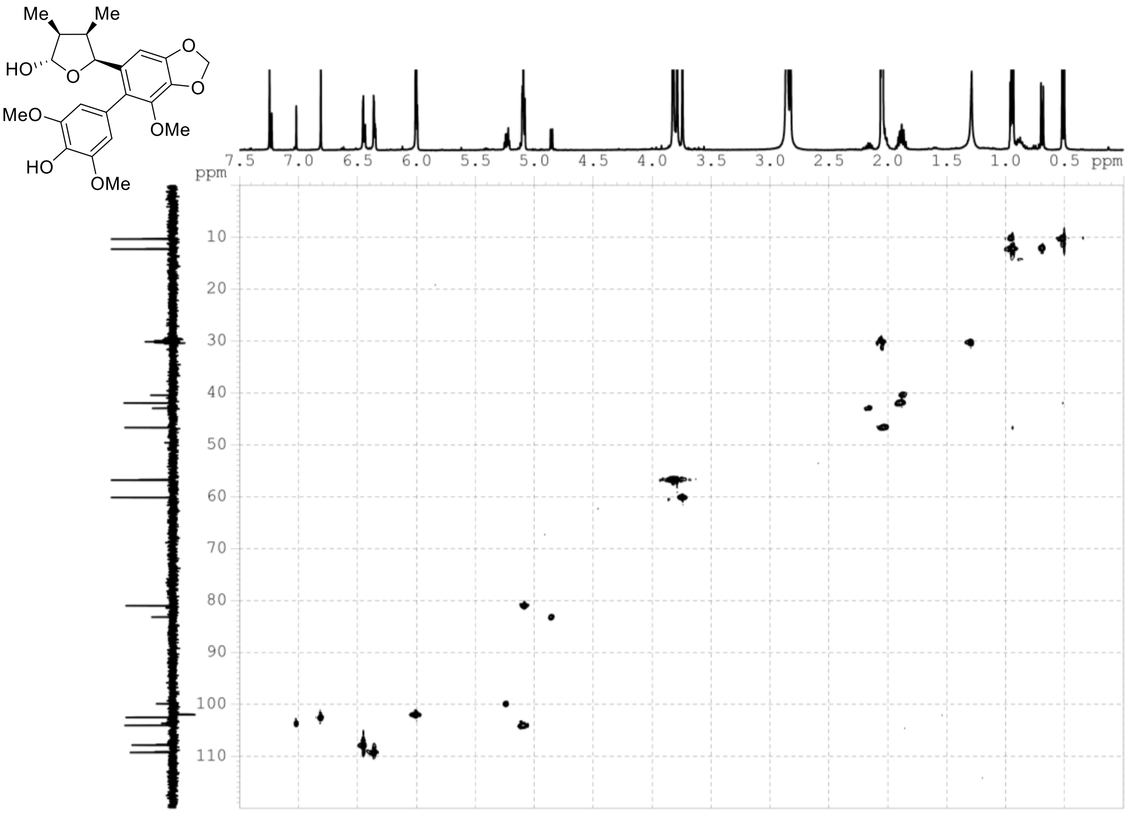

HMBC spectrum of **20** (400 MHz for ^{1}H NMR and 100 MHz for ^{13}C{^{1}H} NMR, acetone-d_6)

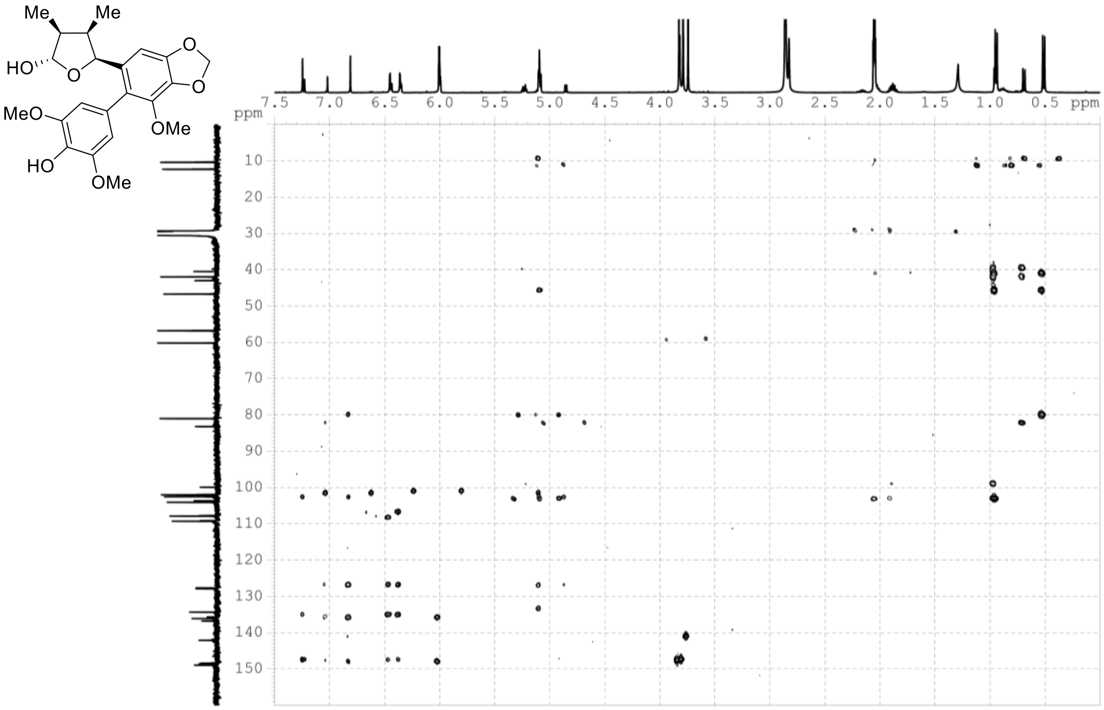

Key COSY correlations of **20** (400 MHz, acetone-d_6)

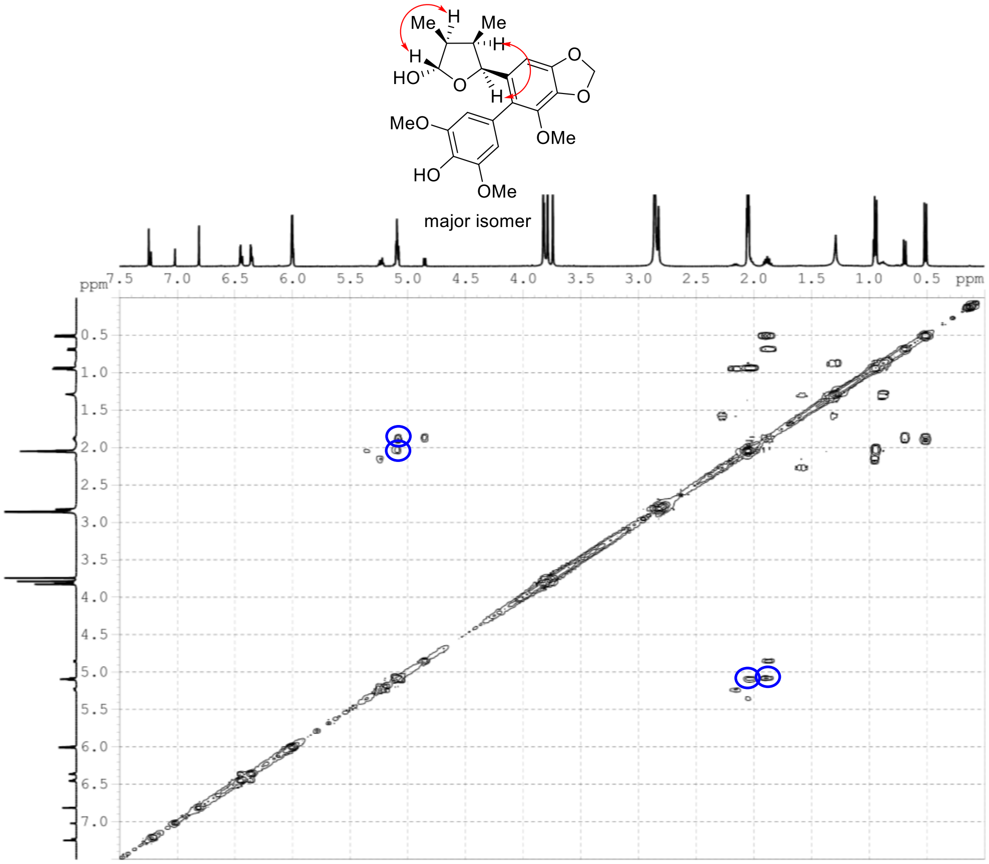

Key NOESY correlations of **20** (400 MHz, acetone-d_6)

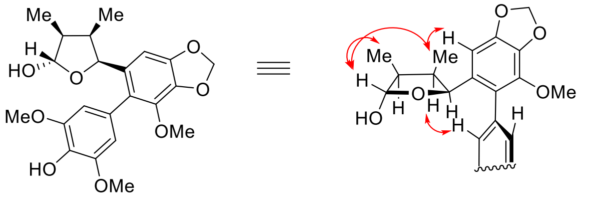

major isomer

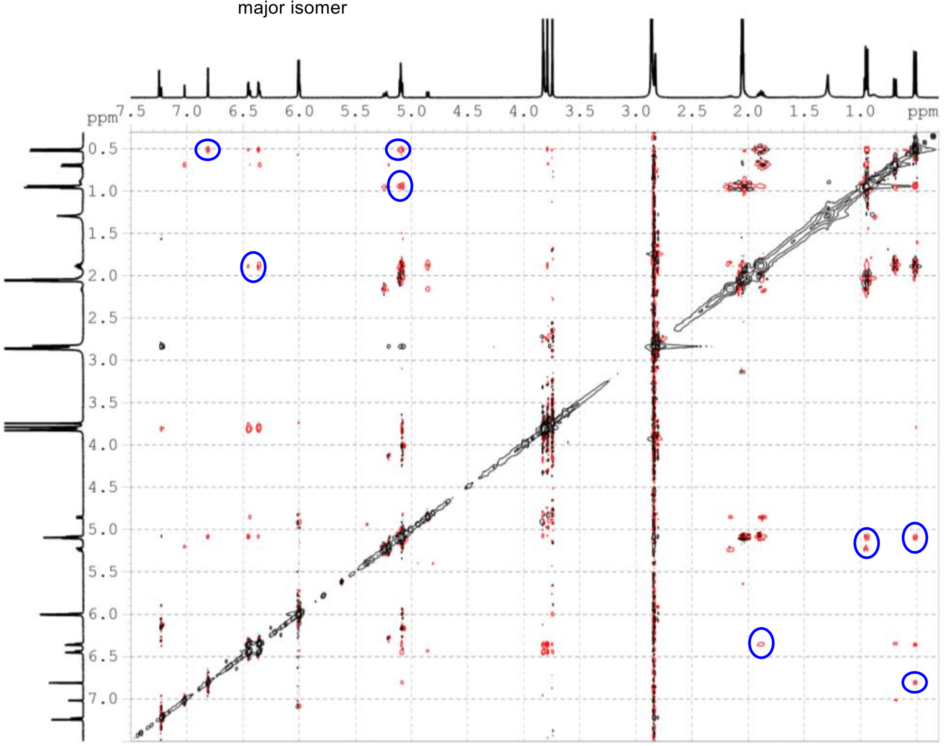

Optical rotation of **20** and its C-2 epimer (dr = 73:27)

Light Source	Na
Monitor wavelength	589 nm
D.I.T.	5 sec
No. of cycle	5
Cycle interval	1 sec
Temp. Monitor	Holder
Temp. Corr. Factor	0 at 25 C
Correct	Blank
Aperture(S)	8.0mm
Aperture(L)	Auto
Mode	Specific O.R.
Path Length	10 mm
Concentration	0.31 w/v%
Water content of sample	0 %
Factor	1

		Sample No.	Mode	Specific O.R.	Temperature(C)	Blank	Measurement Date	Comment
1	*	333F7-1	Specific O.R.	-9.4194	21.98	0.0004	8/19/2025 1:22 PM	
2	*	333F7-2	Specific O.R.	-10.3871	21.98	0.0004	8/19/2025 1:22 PM	
3	*	333F7-3	Specific O.R.	-9.4194	21.99	0.0004	8/19/2025 1:22 PM	
4	*	333F7-4	Specific O.R.	-10.7097	21.98	0.0004	8/19/2025 1:22 PM	
5	*	333F7-5	Specific O.R.	-10.7097	21.98	0.0004	8/19/2025 1:22 PM	
6	*	Avg.		-10.1290				
7		S.D		0.6611				
8		C.V		6.5267				

^{1}H NMR spectrum of **21** (400 MHz, acetone-d_6)

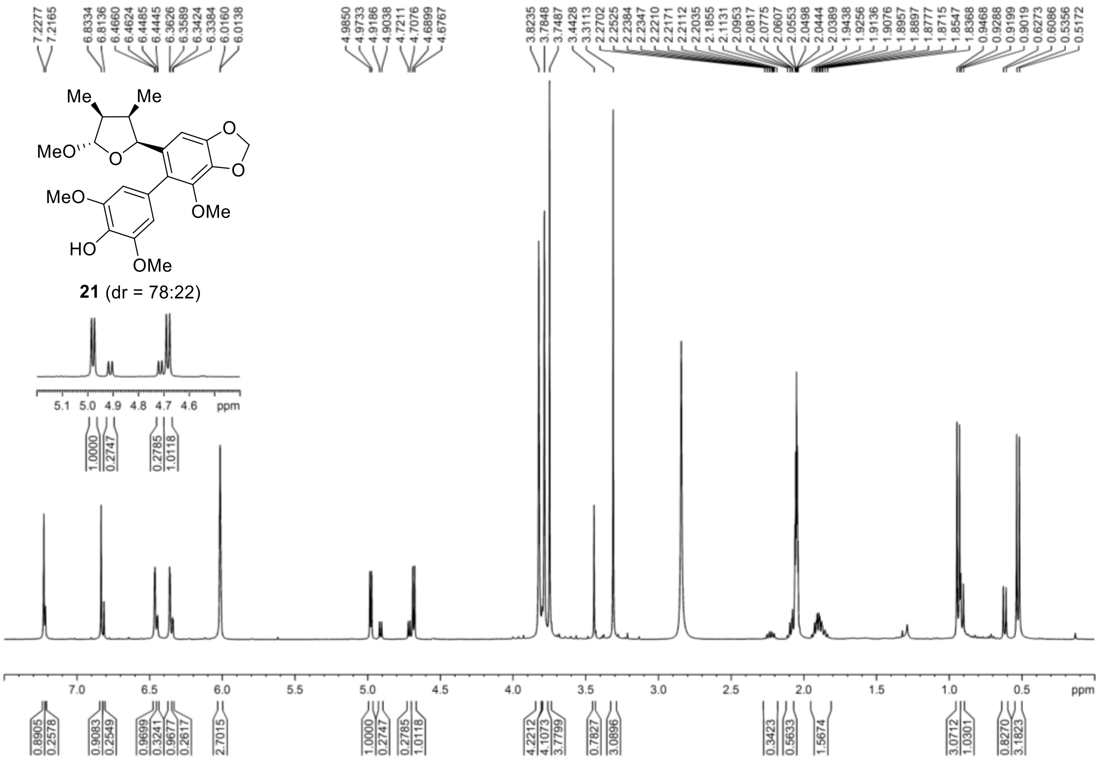

^{13}C{^{1}H} NMR spectrum of **21** (100 MHz, acetone-d_6)

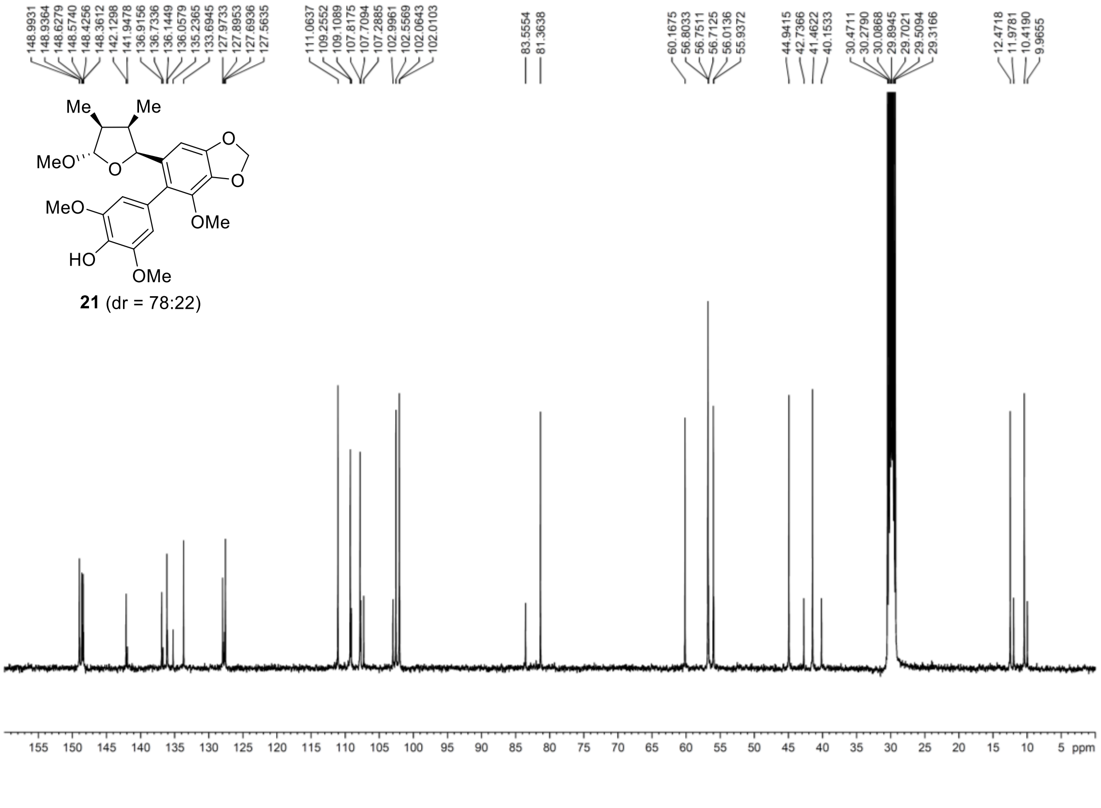

DEPT-135 NMR spectrum of **21** (100 MHz, acetone-d_6)

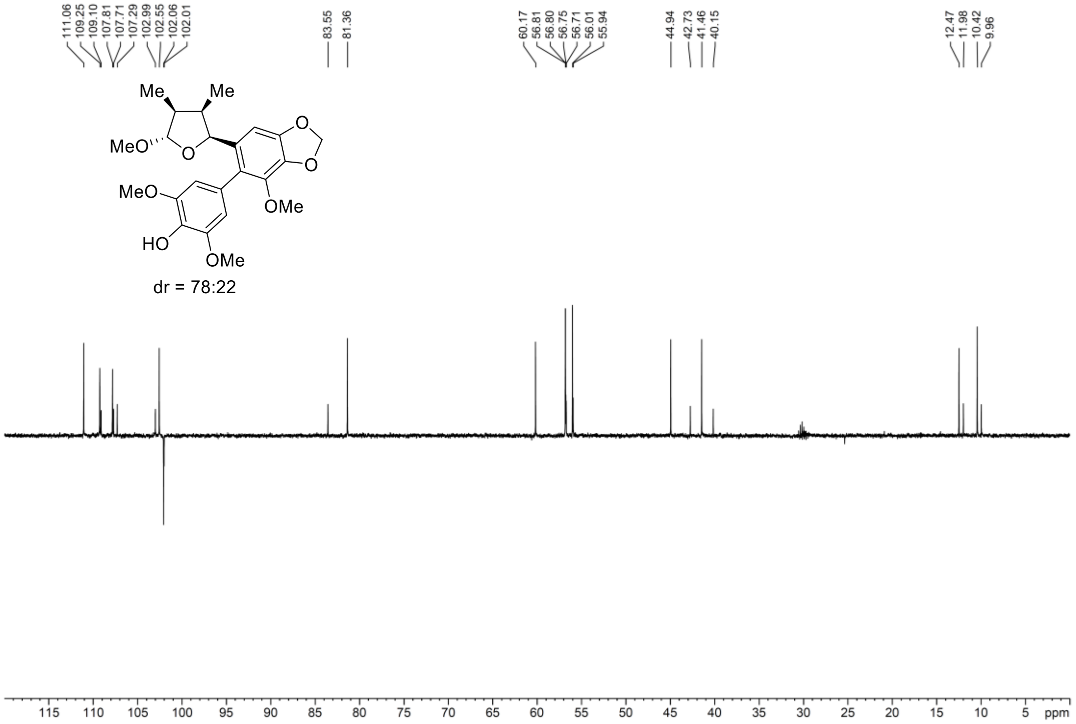

HSQC spectrum of **21** (400 MHz for ^{1}H NMR and 100 MHz for ^{13}C{^{1}H} NMR, acetone-d_6)

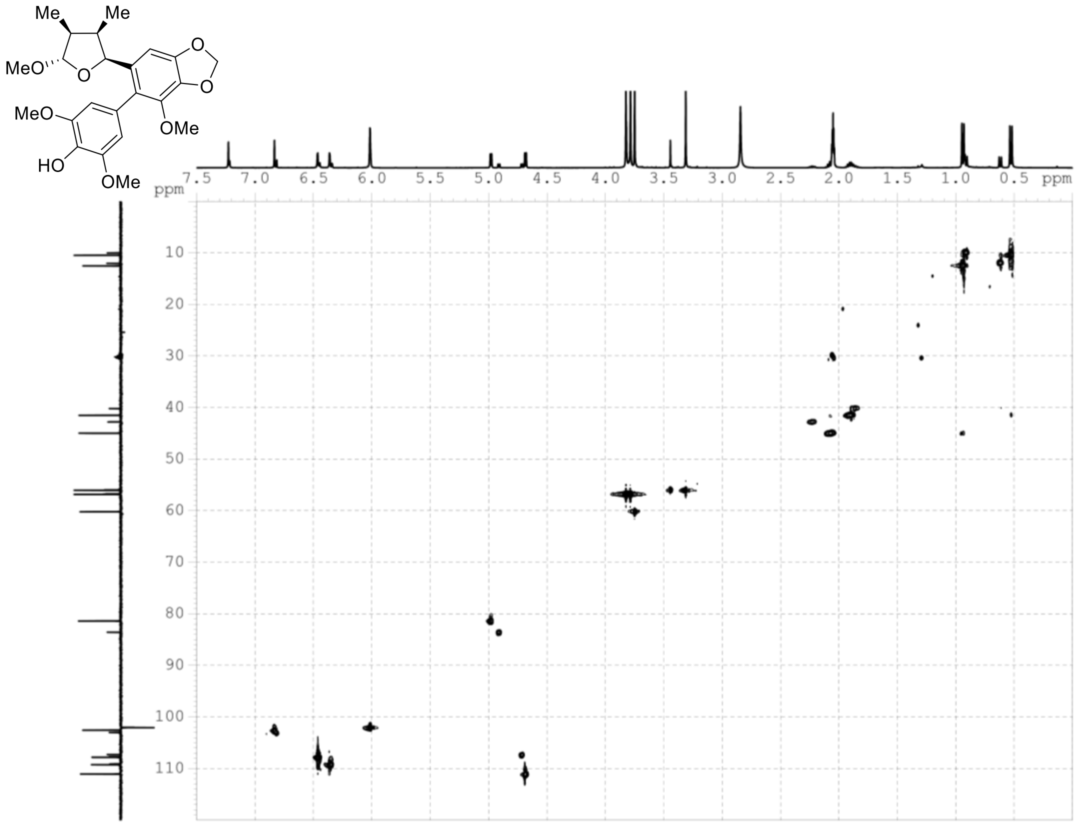

HMBC spectrum of **21** (400 MHz for ^{1}H NMR and 100 MHz for $^{13}C\{^{1}H\}$ NMR, acetone-d_6)

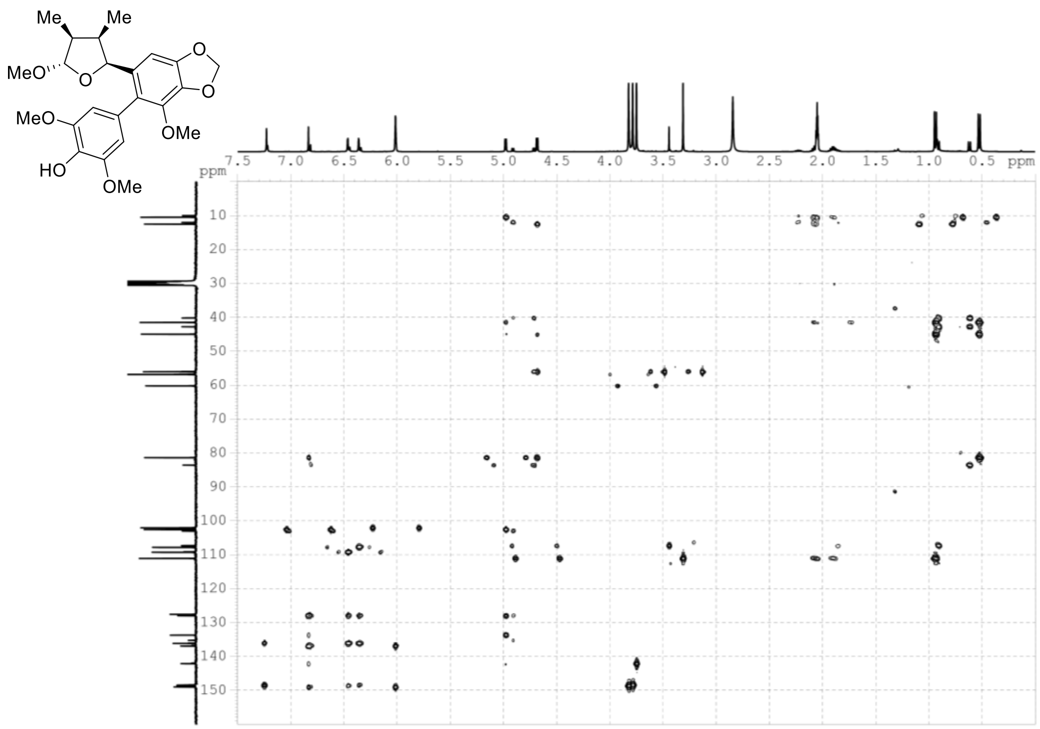

Key COSY correlations of **21** (400 MHz, acetone-d_6)

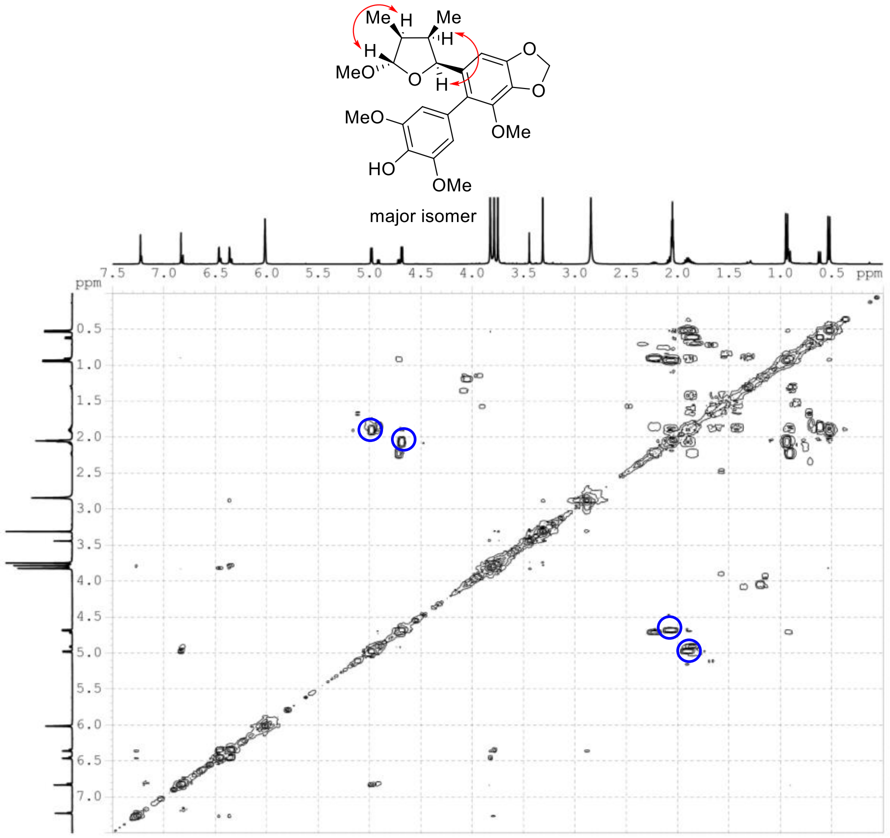

Key NOESY correlations of **21** (400 MHz, acetone-d_6)

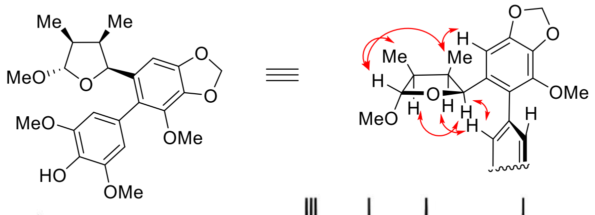

major isomer

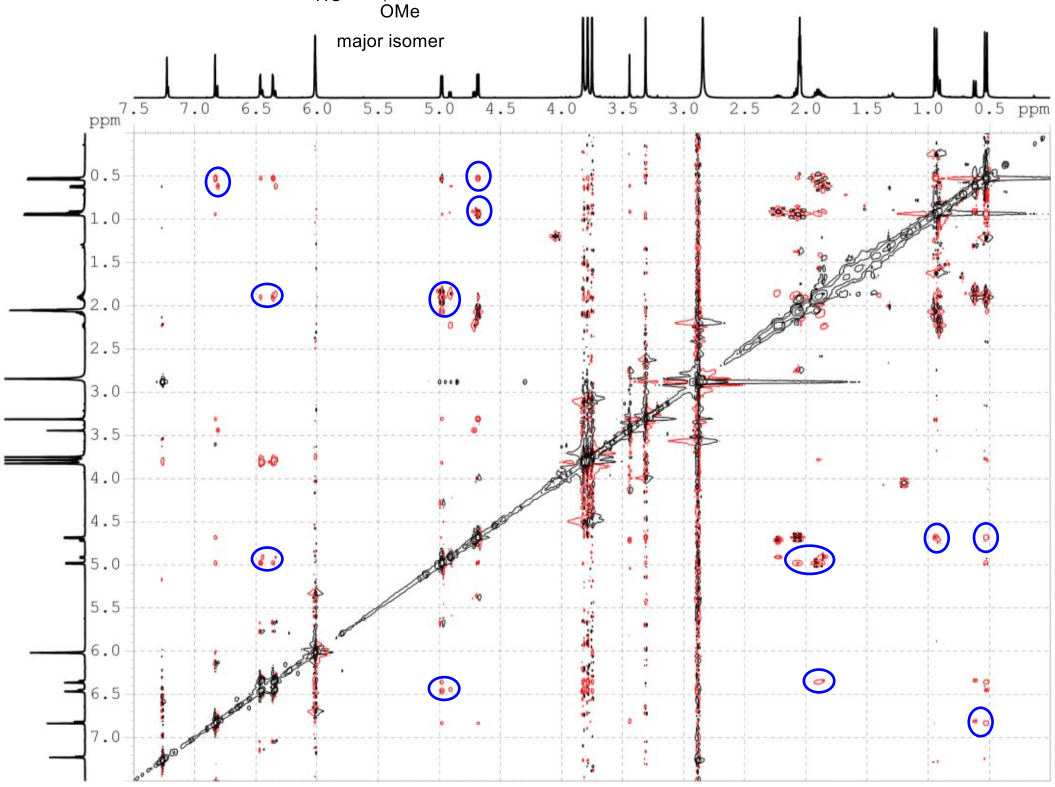

Optical rotation of **21** and its C-2 epimer (dr = 78:22)

Light Source	Na
Monitor wavelength	589 nm
D.I.T.	5 sec
No. of cycle	5
Cycle interval	1 sec
Temp. Monitor	Holder
Temp. Corr. Factor	0 at 25 C
Correct	Blank
Aperture(S)	8.0mm
Aperture(L)	Auto
Mode	Specific O.R.
Path Length	10 mm
Concentration	0.4 w/v%
Water content of sample	0 %
Factor	1

		Sample No.	Mode	Specific O.R.	Temperature(C)	Blank	Measurement Date	Comment
1	*	333F3-1	Specific O.R.	-5.7000	23.10	0.0006	8/1/2025 11:18 AM	
2	*	333F3-2	Specific O.R.	-5.7000	23.10	0.0006	8/1/2025 11:18 AM	
3	*	333F3-3	Specific O.R.	-5.7000	23.10	0.0006	8/1/2025 11:18 AM	
4	*	333F3-4	Specific O.R.	-5.7000	23.09	0.0006	8/1/2025 11:19 AM	
5	*	333F3-5	Specific O.R.	-5.7000	23.09	0.0006	8/1/2025 11:19 AM	
6	*	Avg.		-5.7000				
7		S.D		0.0000				
8		C.V		0.0000				

^{1}H NMR spectrum of **22** (500 MHz, acetone-d_6)

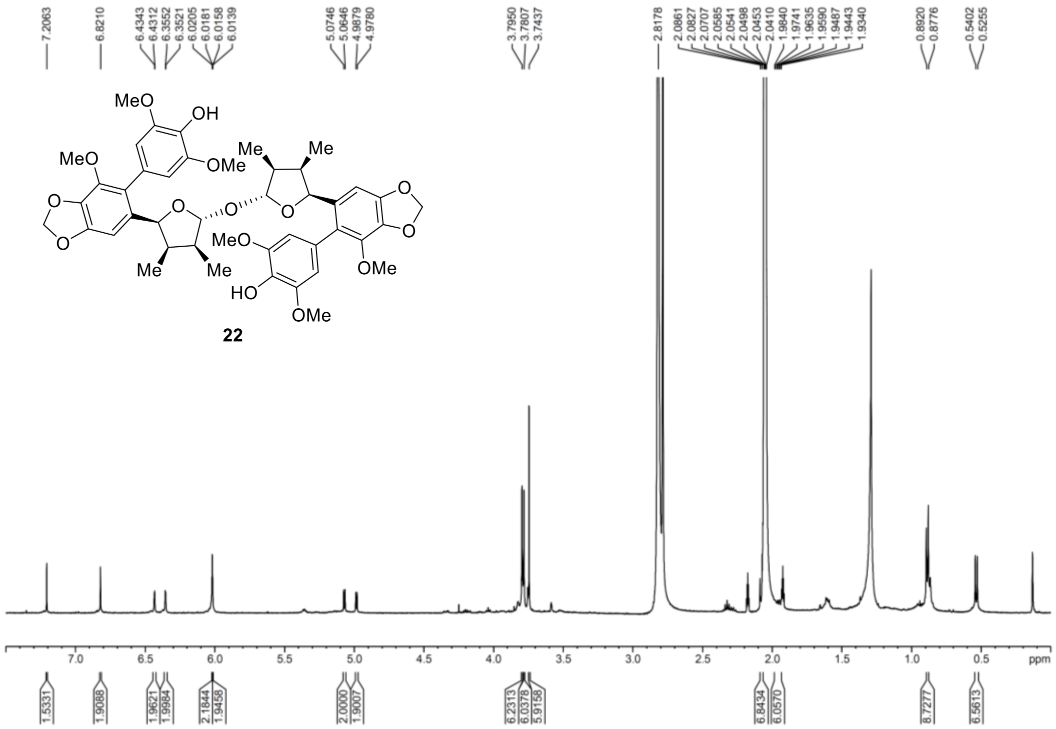

^{13}C{^{1}H} NMR spectrum of **22** (125 MHz, acetone-d_6)

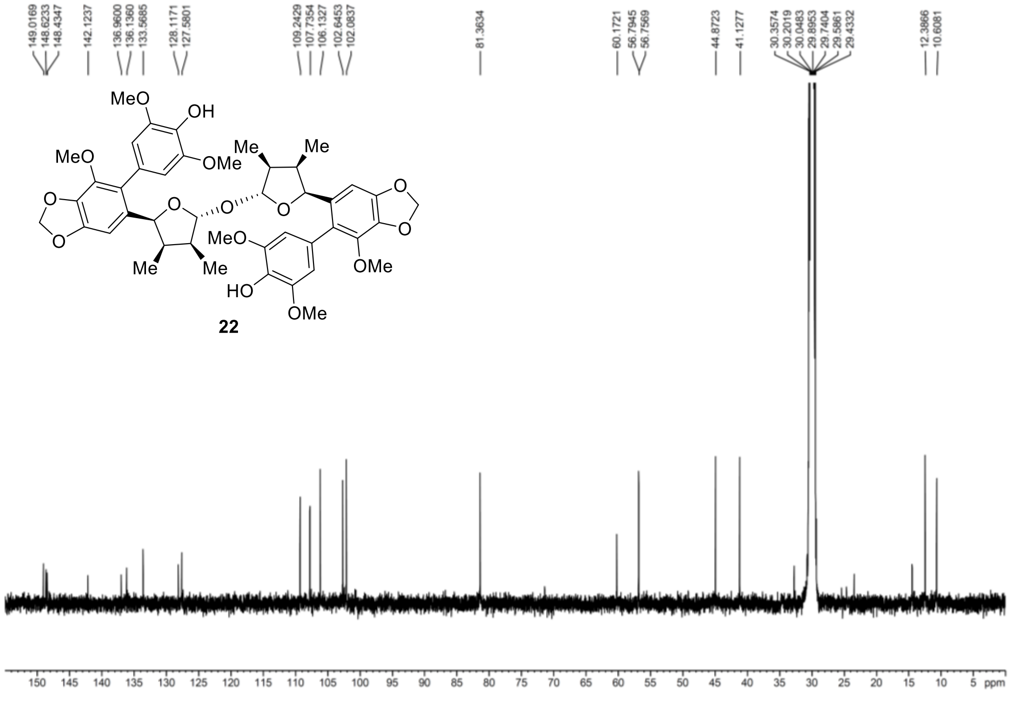

DEPT-135 NMR spectrum of **22** (125 MHz, acetone-d_6)

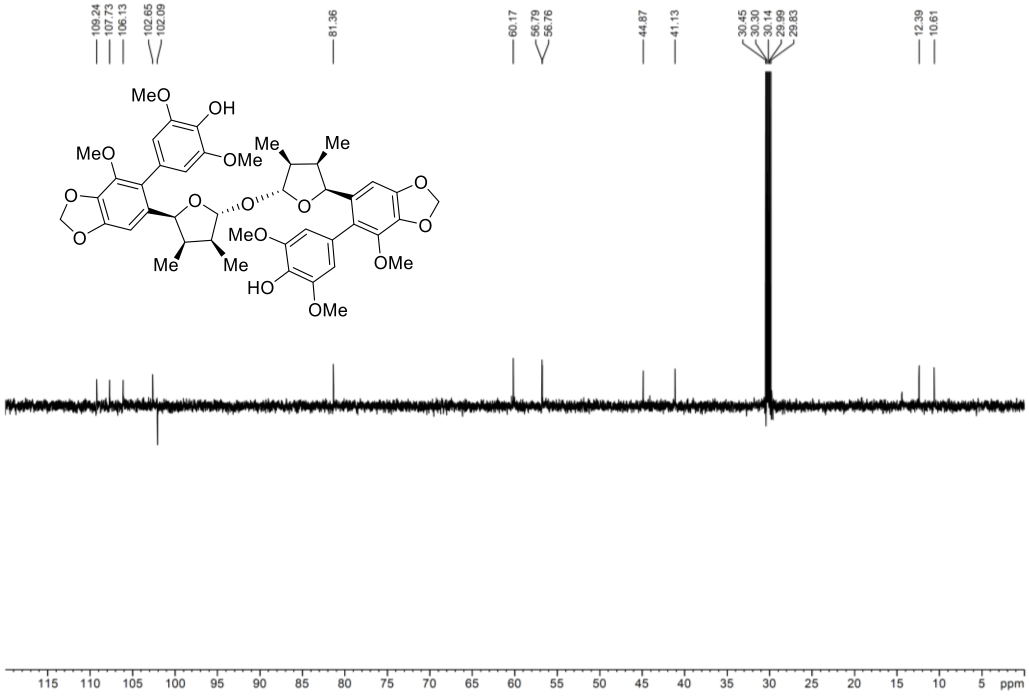

HSQC spectrum of **22** (500 MHz for ^{1}H NMR and 125 MHz for ^{13}C{^{1}H} NMR, acetone-d_6)

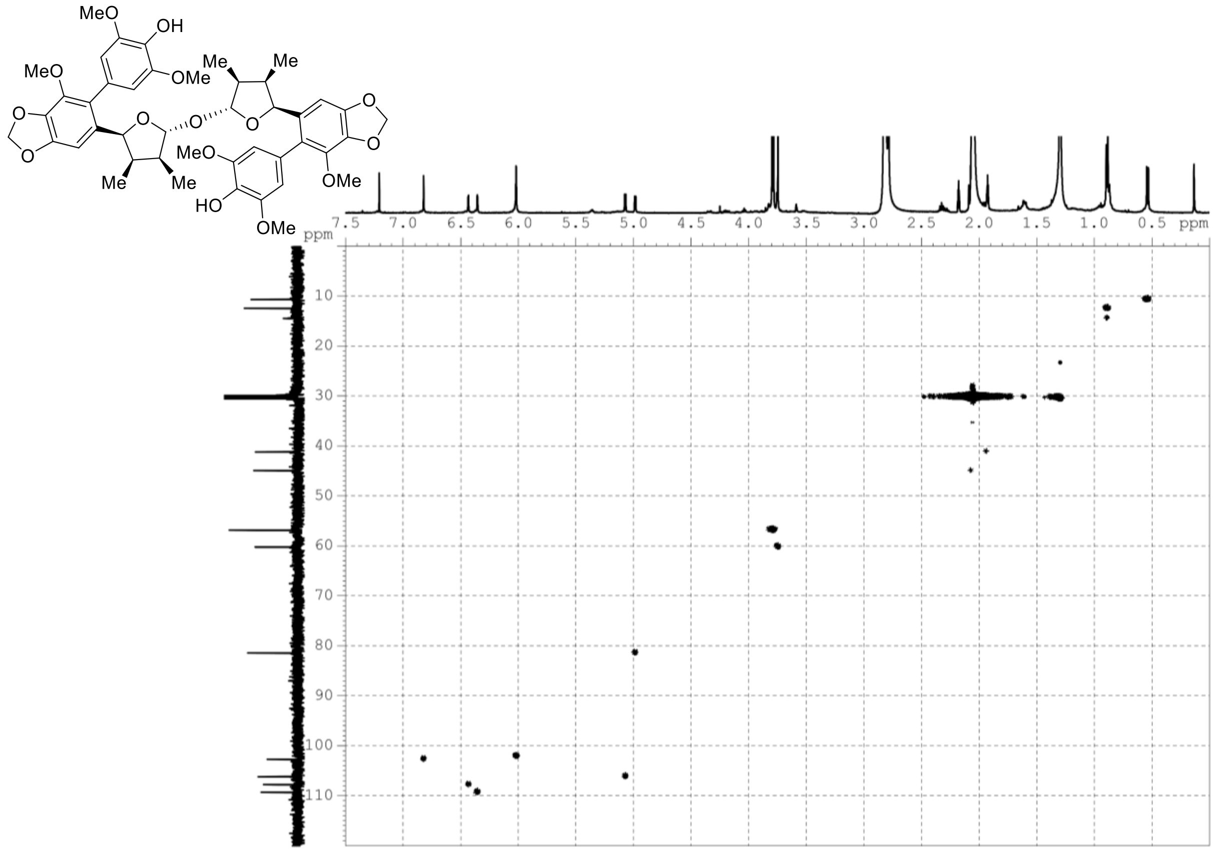

HMBC spectrum of **22** (500 MHz for ^{1}H NMR and 125 MHz for $^{13}C\{^{1}H\}$ NMR, acetone-d_6)

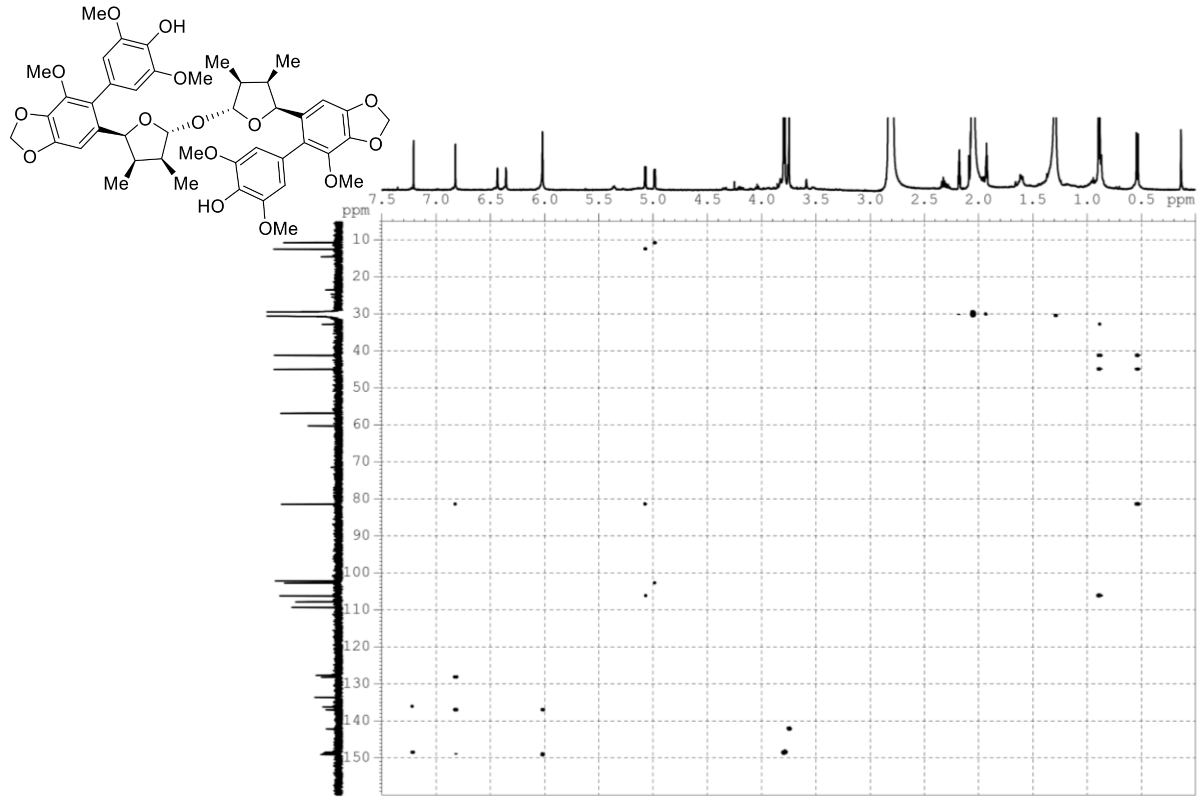

Key COSY correlations of **22** (500 MHz, acetone-d_6)

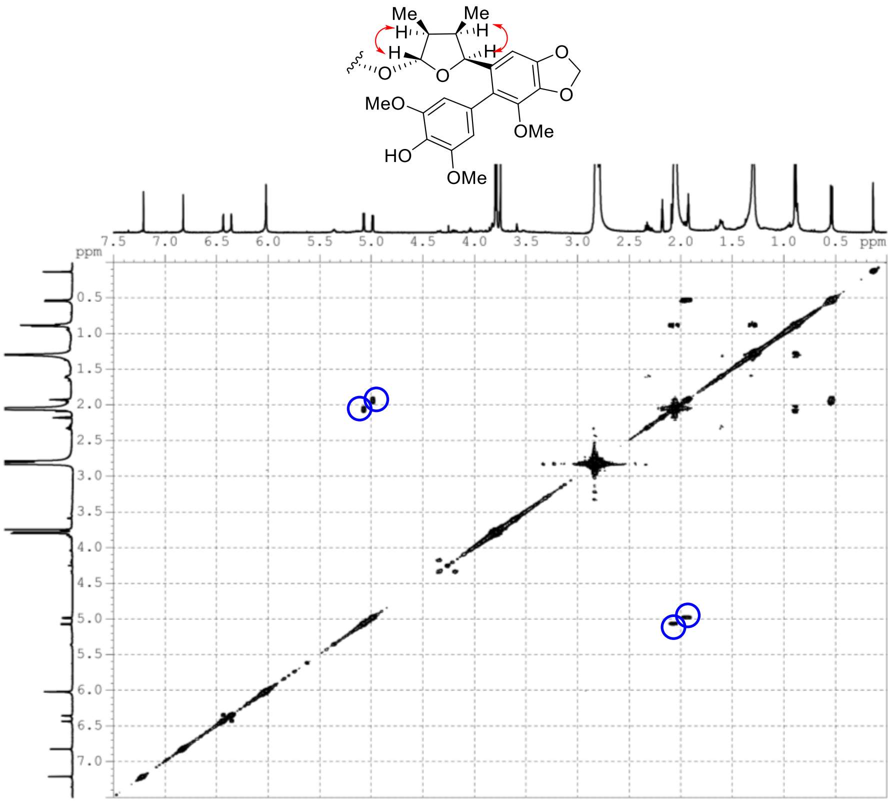

Key NOESY correlations of **22** (500 MHz, acetone-d_6)

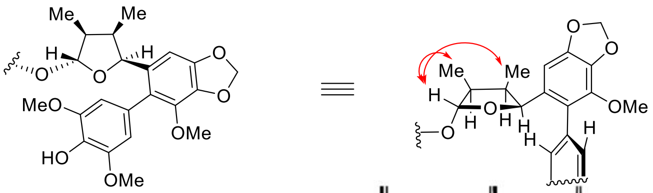

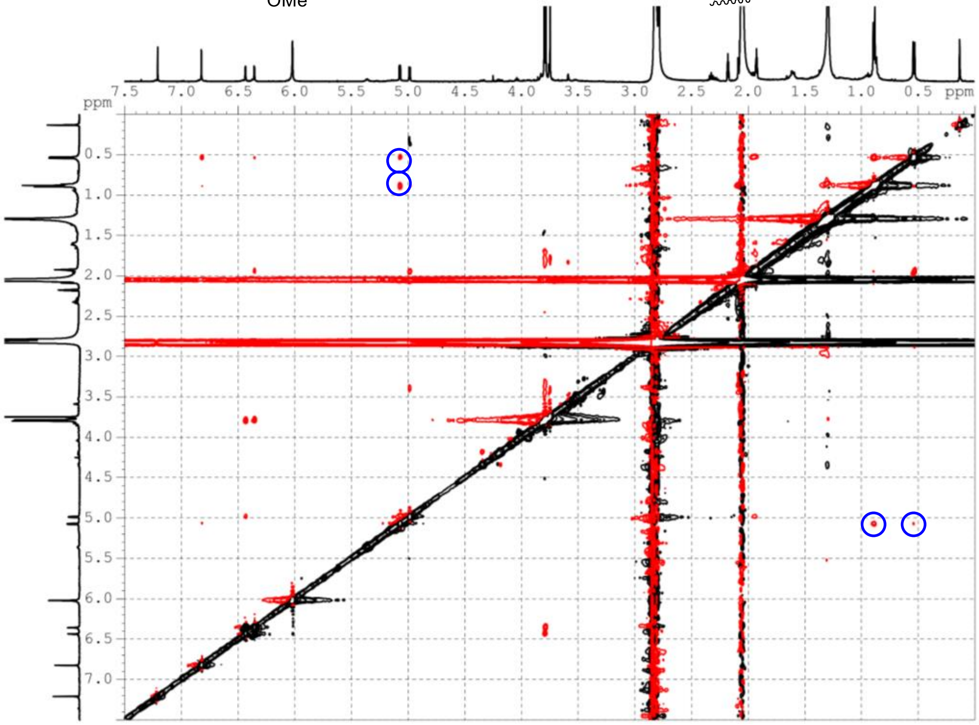

Optical rotation of **22**

Light Source	Na
Monitor wavelength	589 nm
D.I.T.	5 sec
No. of cycle	5
Cycle interval	1 sec
Temp. Monitor	Holder
Temp. Corr. Factor	0 at 25 C
Correct	Blank
Aperture(S)	8.0mm
Aperture(L)	Auto
Mode	Specific O.R.
Path Length	10 mm
Concentration	0.11 w/v%
Water content of sample	0 %
Factor	1

		Sample No.	Mode	Specific O.R.	Temperature(C)	Blank	Measurement Date	Comment
1	*	414P1-1	Specific O.R.	34.1818	24.77	-0.0001	11/7/2025 11:06 AM	
2	*	414P1-2	Specific O.R.	34.1818	24.77	-0.0001	11/7/2025 11:06 AM	
3	*	414P1-3	Specific O.R.	34.1818	24.76	-0.0001	11/7/2025 11:06 AM	
4	*	414P1-4	Specific O.R.	34.1818	24.76	-0.0001	11/7/2025 11:06 AM	
5	*	414P1-5	Specific O.R.	34.1818	24.76	-0.0001	11/7/2025 11:06 AM	
6	*	Avg.		34.1818				
7		S.D		0.0000				
8		C.V		0.0000				

Cytotoxic effects on cholangiocarcinoma (CCA) cell lines

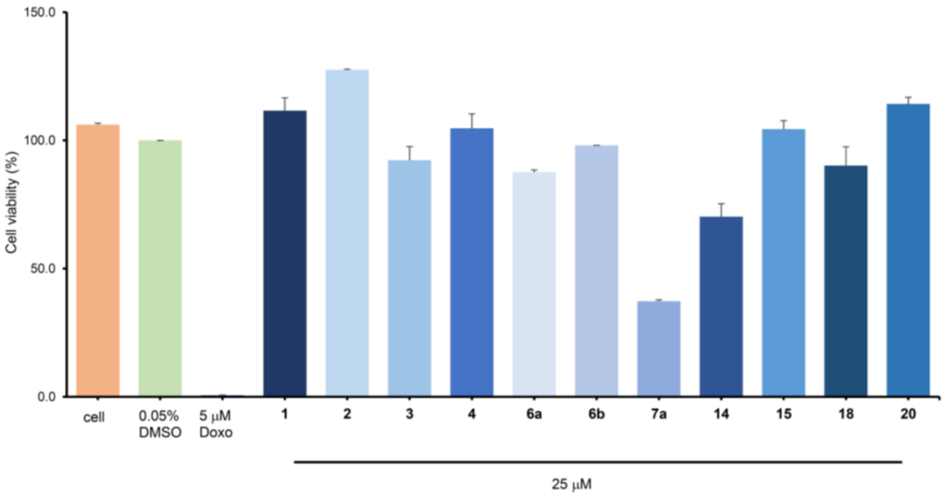

Figure SI-1. Cytotoxic effects of compounds **1**, **2**, **3**, **4**, **6a**, **6b**, **7a**, **14**, **15**, **18**, and **20** in cholangiocarcinoma cell line (KKU-M213).

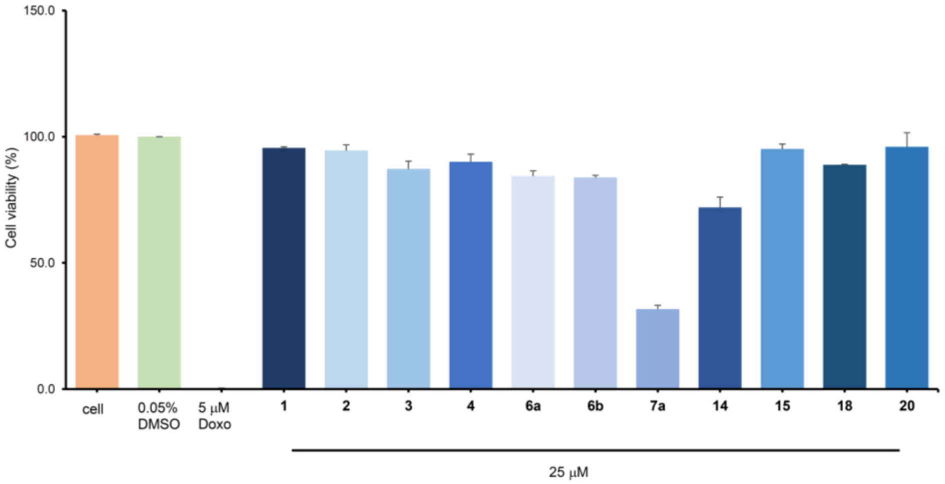

Figure SI-2. Cytotoxic effects of compounds **1**, **2**, **3**, **4**, **6a**, **6b**, **7a**, **14**, **15**, **18**, and **20** in cholangiocarcinoma cell line (KKU-M055).
